# Thiourea-derived coating enabled lithium-rich manganese oxide positive electrode in solid-state batteries

**DOI:** 10.1038/s41467-026-75215-1

**Published:** 2026-07-15

**Authors:** Feng Jin, Wenguang Zhao, Ingeborg Sellæg Ellingsen, Henrik Rotvær Bratlie, Quoc Hung Nguyen, Dragos Stoian, Kenneth Marshall, Wouter van der Beek, Per Erik Vullum, Manuel Dillenz, Jose Maria Castillo Robles, Juan Maria Garcia Lastra, Ivano Eligio Castelli, Feng Pan, Günther J. Redhammer, Daniel Rettenwander

**Affiliations:** 1 Department of Material Science and Engineering, NTNU Norwegian University of Science and Technology, Trondheim, Norway; 2 School of Advanced Materials Shenzhen Graduate School, Peking University, Shenzhen, PR China; 3 Swiss-Norwegian Beam Lines, European Synchrotron Radiation Facility, Grenoble, France; 4 Sintef Industry, Trondheim, Norway; 5 Department of Energy Conversion and Storage, Technical University of Denmark, Kongens Lyngby, Denmark; 6 Department of Chemistry and Physics of Materials, University of Salzburg, Salzburg, Austria; 7 Christian Doppler Laboratory for Solid-State Batteries, NTNU Norwegian University of Science and Technology, Trondheim, Norway; 8 AIT Austrian Institute of Technology GmbH, Center for Transport Technologies, Battery Technologies, Vienna, Austria; 9 Institute of Chemical Technologies and Analytics, Vienna University of Technology, Vienna, Austria

**Keywords:** Batteries, Batteries

## Abstract

Solid-state batteries employing lithium-rich manganese oxide positive electrodes are a highly promising candidate for next-generation high-energy-density energy storage systems. However, the practical deployment of lithium-rich manganese oxide positive electrodes is hindered by several critical challenges, including poor initial-cycle reversibility, rapid capacity decay, structural collapse due to oxygen release, and interfacial instability at high potentials. Here, we introduce a thiourea-derived surface modification strategy for lithium-rich manganese oxide positive electrodes, which significantly enhances the electrochemical performance of solid-state batteries (SSBs). The modified lithium-rich manganese oxide positive electrodes exhibit an initial discharge capacity of 220.2 mAh g^−1^, an initial Coulombic efficiency of 84.83 %, and capacity retention of 97 % after 600 cycles at 1 C under 4.6 V (vs. Li^+^/Li). The improved cycling performance is shown to be attributed to a dual modification of lithium-rich manganese oxide particles, i.e., the application of sub-nm-thick S-rich coating layer and formation of a spinel-like structure in the surface near proximity, which prevents oxygen-related degradation and accelerates Li^+^ transport, respectively. These findings present a scalable surface modification strategy that potentially addresses key limitations of lithium-rich manganese oxide-based SSBs, paving the way for the development of stable, high-energy-density batteries.

## Abstract (web summary)

Lithium-rich manganese oxide positive electrodes enable high-energy batteries but suffer from instability and rapid decay. Here, authors develop a thiourea-derived surface modification that stabilizes structure, improves reversibility, and enables long-lived, high-capacity solid-state battery performance.

## Introduction

Solid-state batteries (SSBs) with Li-rich manganese oxide (LRMO) positive electrodes represent a pivotal advancement toward lithium-ion batteries with energy densities exceeding 500 Wh/kg and 1000 Wh/l^1^. LRMO positive electrodes stand out due to their superior specific capacities (>250 mAh g^−1^), high operating potentials (≥4.6 V vs. Li^+^/Li), cost efficiency, sustainability, and thermal stability, outperforming traditional layered metal oxide positive electrodes such as LiCoO_2_ and LiNi_0.8_Mn_0.1_Co_0.1_O_2_^2^. These advantages originate from the combined contributions of oxygen anions and transition metal (TM) cations in charge compensation.

Active oxygen species, facilitated by the Li-O-Li superlattice configuration, participate in electrochemical reactions at potentials above 4.5 V^3^. However, this unique anion redox process generates reactive oxygen species (O^2−n^; 0 < *n* < 2), which can oxidize electrolytes, impede Li^+^ transport, and cause low initial Coulombic efficiency (ICE) alongside irreversible charge/discharge capacities^4–6^. Furthermore, the inherently low electronic conductivity of Li_2_MnO_3_, up to four orders of magnitude lower than that of conventional layered oxides, limits the charge-discharge kinetics^7^.

To address these challenges, surface modifications have shown promise in stabilizing oxygen redox reactions and improving electrochemical performance. For example, Li_3_PO_4_ coatings applied via atomic layer deposition or functionalization with ${{{\rm{S}}}}{{{{\rm{O}}}}}_{3}^{2-}$ groups have enhanced anion redox reversibility through reversible “sponge-like” reactions (e.g., ${{{{\rm{SO}}}}}_{3}^{2-}+{{{{\rm{O}}}}}^{2-}\rightleftharpoons {{{{\rm{SO}}}}}_{4}^{2-}$)^8,9^. Similarly, applying Li_2_WO_4_ coatings has demonstrated improvements in interfacial stability, resulting in enhanced cycling performance and oxygen redox reversibility. While the processing-induced W interdiffusion is modifying the near-surface LRMO structure, mitigating TM migration^10^. Additionally, processing-induced W interdiffusion is modifying the near-surface LRMO structure, mitigating TM migration.

Building upon these findings, we introduce an approach that integrates surface reconstruction in combination with a thin S-rich coating, addressing interfacial incompatibility caused by O-involving reactions and limited kinetics in LRMO-based SSBs. This strategy aims to overcome the persistent limitations of previous surface treatments and unlock LRMO’s potential. This is realized by immersing LRMO (Li_1.2_Mn_0.5__4_Ni_0.13_Co_0.13_O_2_) in a thiourea (SC(NH_2_)_2_)-based solution followed by annealing. The resulting modified LRMO (S@LRMO) delivers an enhanced initial discharge capacity (from 138 mAh g^−1^ to 220.2 mAh g^−1^) and improved ICE (from 75.46% to 84.83%). Moreover, it exhibits comparable cycling stability, retaining 97% capacity after 600 cycles at 1 C and 25 ± 2 °C. Comprehensive characterization techniques—including transmission electron microscopy (TEM), in situ galvanostatic electrochemical impedance spectroscopy (GEIS) in combination with a distribution of relaxation time analysis (DRT), galvanostatic intermittent titration technique (GITT), *operando* synchrotron X-ray diffraction (sXRD), X-ray photoelectron spectroscopy (XPS) and time-of-flight secondary ion mass spectrometry (ToF-SIMS)—reveal that the coating induces structural reconstruction of the LRMO surface, transitioning the topmost 1–2 nm to a spinel-type structure while forming a S-rich layer comprising ${{{\rm{S}}}}{{{{\rm{O}}}}}_{3}^{2-}$ and ${{{\rm{S}}}}{{{{\rm{O}}}}}_{4}^{2-}$. This dual modification mitigates oxygen-related degradation and accelerates Li^+^ transport through the surface-near spinel phase.

Our findings demonstrate that the synergistic effects of surface reconstruction and sulfur-based functionalization can effectively mitigate oxygen-related degradation while enhancing Li-ion transport in LRMO positive electrodes. By stabilizing the anion redox process and improving interfacial compatibility, this strategy addresses critical challenges that have limited the practical implementation of LRMO in SSBs. The structural and electrochemical insights gained from this work not only pave the way for more stable and high-capacity positive electrodes but also provide a broader design principle for engineering next-generation positive electrode materials.

## Results and discussions

### Coating and surface-reconstruction of LRMO particles

**Fig. 1:**
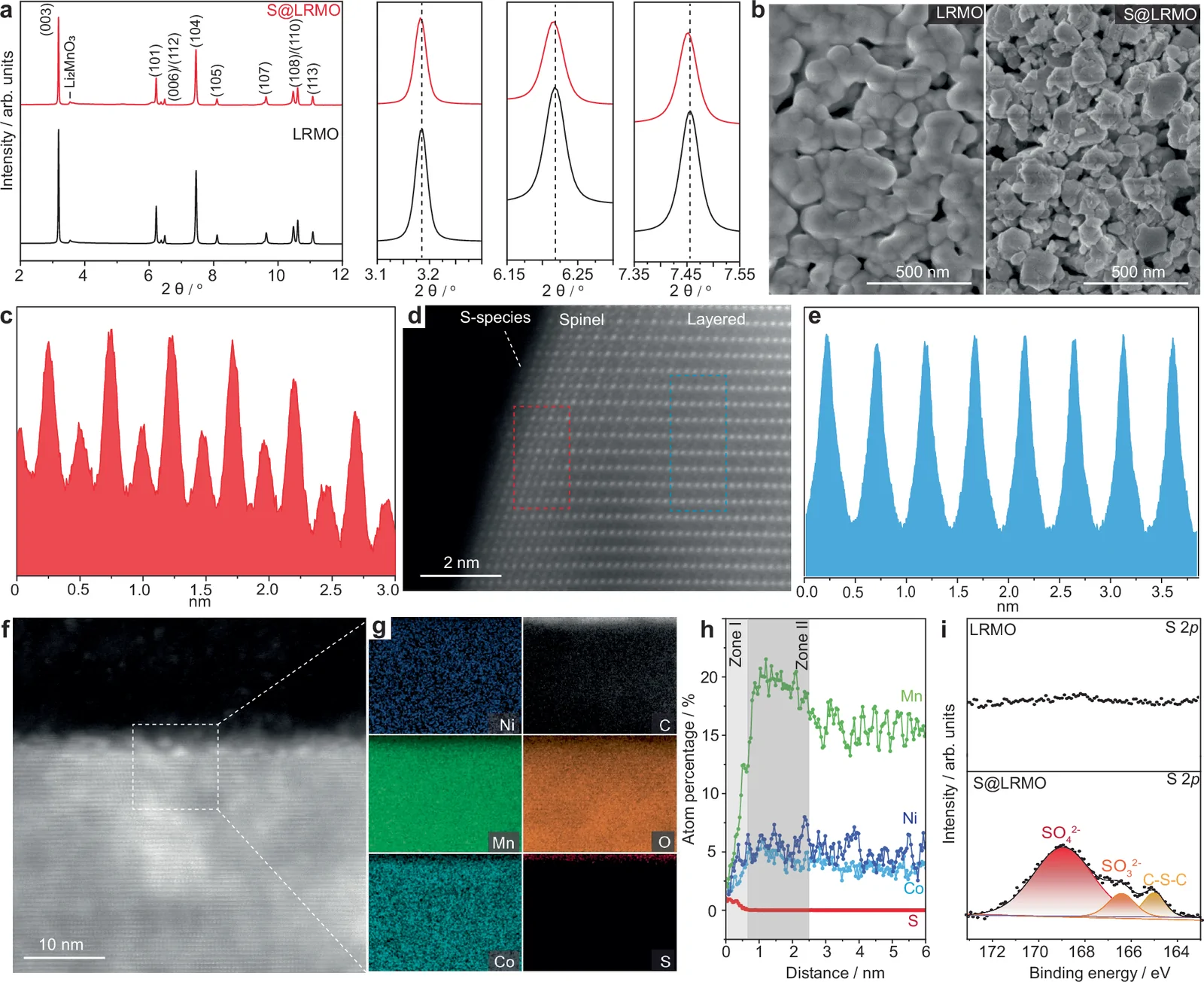
Positive electrode materials characterizations. **a** Synchrotron X-Ray Diffraction (sXRD) patterns collected from the bare lithium-rich manganese oxide (LRMO) and modified lithium-rich manganese oxide (S@LRMO) powders; (**b**) SEM images of bare LRMO (left) and S@LRMO (right) particles; (**d**) High-Angle Annular Dark-field Scanning Transmission Electron Microscopy (HAADF-STEM) image from a S@LRMO positive electrode particle and the lattice space (**c**, **e**) of selected areas from its surface and bulk; (**f**) HAADF-STEM image from a S@LRMO positive electrode particle and its corresponding element maps in (**g**) extracted from the electron energy loss spectroscopy (EELS) data after mapping (the region inside the white dashed box in the STEM image); (**h**) Element line profiles based on the maps in (**g**); (**i**) S 2*p* X-ray photoelectron spectroscopy (XPS) spectrum for bare LRMO and S@LRMO particles.

The LRMO particles were modified using a liquid-phase coating process followed by annealing (see Methods and Fig. S1). To examine potential changes in phase, structure, and crystallinity after the coating process, sXRD was conducted on pristine LRMO and S@LRMO. The diffraction patterns are shown in Fig. 1a. Rietveld refinement reveals that pristine LRMO exhibits a mixture of trigonal (space group *R*−3*m*) and monoclinic (space group *C*2/*m*) symmetries, with lattice parameters *a* = 2.85181(6) Å, *c* = 14.2440(2) Å and *V* = 100.324(2) Å^3^ (Fig. S2)^11,12^. The site occupation numbers suggest minor anti-site defects, with ~0.014(6) M^2+^ occupying Li1 sites and 0.194(6) Li^+^ present on M^2+^ sites. Minor amounts of spinel-type Mn-rich compounds (2.3(5) wt%) and disordered monoclinic Li_2_MnO_3_ (4.0(8) wt%) were detected as impurity phases. The coating process introduces minor structural changes to LRMO (see also in TEM analysis), with a slight increase in lattice parameters (*a* = 2.85315(6) Å, *c* = 14.2563(3) Å, and *V* = 100.504(2) Å^3^), accompanied by a modest rise in anti-site defects (0.028(6) M^2+^ on Li1 sites) (Supplementary Table 1). The particle interior remained predominantly intact, retaining its layered structure. However, a significant increase in the spinel phase (space group *Fd-*3*m*) from 2.3(5) wt% in pristine LRMO to 10.2 wt% in S@LRMO, as evidenced by the emergence of the (311) spinel reflection at ~6° of 2θ (*d* = 3.48 Å) was observed^13,14^. Notably, the broad Bragg peaks of the spinel phase indicate the presence of nanoscale crystallites. Details of the refinement process are provided in Method section.

To confirm the successful formation of a uniform and homogeneous coating on LRMO particles and to elucidate its chemical, compositional, and structural characteristics, electron microscopy techniques and XPS were employed.

Scanning electron microscopy (SEM) images reveal that the LRMO surface became rougher after coating (Fig. 1b and Fig. S3), hinting towards a successful surface modification. High-angle annular dark-field  scanning transmission electron microscopy (HAADFSTEM) image of S@LRMO showed two-layered coating, and the inert layer was a spinel phase (Fig. 1c-e). And TEM combined with electron energy loss spectroscopy (EELS) along the cross-section of S@LRMO further proved the existence of two-layered coating, which contained 0.7 nm thick S-containing species (shown in EELS mapping of Sulfur in Fig. 1g and line scan spectra highlighted in Zone I in Fig. 1h) and Mn-rich layer (see Zone II in Fig. 1h) on LRMO particles. The Mn-enriched layer hinting towards the formation of a spinel phase LiMn_2_O_4_, agreeing with the lattice space shown in Fig. 1c and sXRD observations where an enhancement of the amount of the spinel phase was detected. Besides, the existence of spinel phase was also evidenced by Raman spectrum of S@LRMO. Specifically, a small shoulder peak at near 652 cm^−1^ emerged, which is related to the Mn-O stretching vibration in the spinel structure (Fig. S9)^15^.

XPS spectra for both pristine LRMO and S@LRMO (Fig. 1i and Supplementary Figs. S10–13) further corroborate these findings. Notably, no N-containing species are detected in the N 1 s spectrum (Fig. S10). In contrast, the S 2*p* spectrum revealed peaks corresponding to ${{{{\rm{SO}}}}}_{4}^{2-}$ (168.9 eV), ${{{{\rm{SO}}}}}_{3}^{2-}$ (166.4 eV), and C-S-C species (165.1 eV) ^8,16^. Additionally, O 1*s* spectra (Fig. S11) show peaks at 530.9 eV and 532.6 eV, further confirming the presence of these sulfur species^17–19^. Notably, TM ion signals exhibit shifts toward higher oxidation states, likely resulting from oxidation during the annealing process (Fig. S13)^16^.

Based on the above results and analysis, we propose that the formation of sulfur-containing species and the spinel phase proceeds via the following reactions. When LRMO particles with an alkaline surface are introduced into the solvent, thiourea decomposes according to Reaction (1)^20^.

1 $${{{\rm{SC}}}}{({{{{\rm{NH}}}}}_{2})}_{2}+2{{{{\rm{OH}}}}}^{-}\to {{{{\rm{S}}}}}^{2-}+{{{\rm{CO}}}}{({{{{\rm{NH}}}}}_{2})}_{2}+{{{{\rm{H}}}}}_{2}{{{\rm{O}}}}$$

Subsequently, the generated CO(NH_2_)_2_ further decomposes to produce NH_3_, as described by Reaction (2)^21^. The absence of a discernible N 1 s signal in the XPS spectra (Fig. S13) is consistent with the volatilization of nitrogen-containing species during this process.

2 $$2{{{\rm{CO}}}}{({{{{\rm{NH}}}}}_{2})}_{2}\to {{{{\rm{NH}}}}}_{3}+2{{{\rm{HCNO}}}}$$

During the annealing step, the produced S^2-^ species and/or NH_3_ act as reducing agents, promoting the partial reduction of Li_2_MnO_3_ and inducing its transformation into the spinel LiMn_2_O_4_ phase^22^. Concurrently, S^2−^ is oxidized to sulfite and sulfate species, as summarized in Reaction (3).

3 $${{{{\rm{O}}}}}_{{{{\rm{Lattice}}}}}+{{{{\rm{O}}}}}_{2}+{{{{\rm{S}}}}}^{2-}+{{{{\rm{Li}}}}}_{2}{{{\rm{Mn}}}}{{{{\rm{O}}}}}_{3}\to {{{{\rm{SO}}}}}_{3}^{2-}+{{{{\rm{SO}}}}}_{4}^{2-}+{{{{\rm{LiMn}}}}}_{2}{{{{\rm{O}}}}}_{4}$$

### Electrochemical performances

**Fig. 2:**
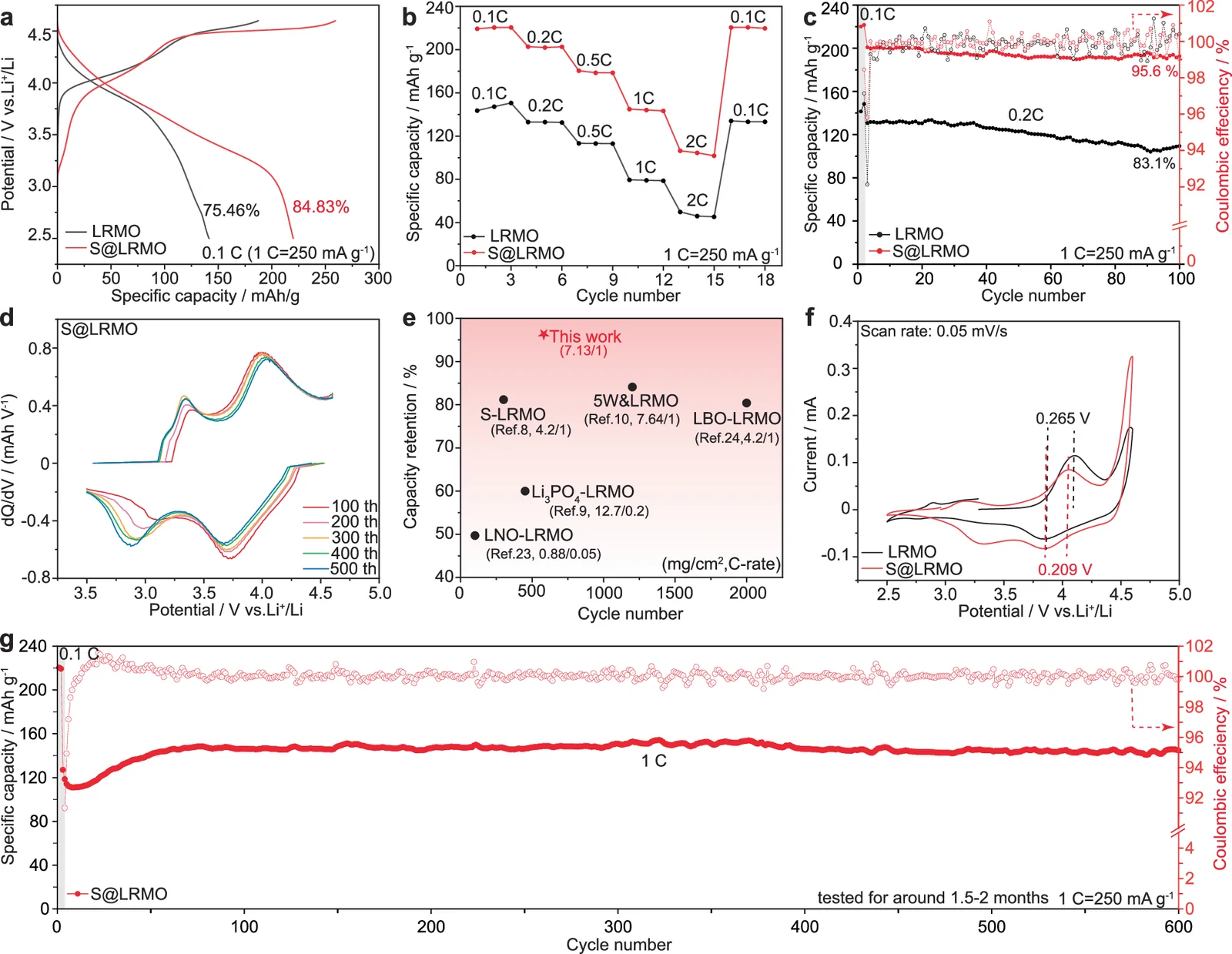
Electrochemical performances of LRMO and S@LRMO. **a** Galvanostatic charge-discharge curves for LRMO and S-LRMO-based solid state batteries (SSBs) in the initial cycle at 0.1 C; (**b**) Charge/discharge-specific capacity at different current densities for LRMO and S-LRMO-based SSBs; (**c**) Cycling performance comparison of LRMO and S-LRMO SSBs at 0.2 C; (**d**) dQ/dV curves of S-LRMO SSBs at various cycles; (**e**) Comparison of the cycling performance between previously reported LRMO-based SSBs based on various coatings^8–10,23,24^; (**f**) Comparison of the Cyclic Voltammetry (CV) curves of LRMO and S@LRMO positive electrode with a scan rate of 0.05 mV/s from 2.5 to 4.6 V for initial cycle; (**g**) The long-term cycling of S-LRMO SSBs at the current density of 1.0 C. And 1 C = 250 mA g^−1^. All the cells are measured at 25 ± 2 °C.

To assess the effect of the LRMO coating on SSB performance, both S@LRMO and pristine LRMO positive electrodes were tested in cells with the configuration LiIn|Li_6_PSCl_5_ (LPSCl)|Li_3_InCl_5.4_F_0.6_ (LICF)/positive electrode/C (Fig. S16). LICF was chosen as the catholyte owing to its high oxidative stability compared to LPSCl and comparable high Li-ion conductivity (0.9 mS/cm) (Fig. S17). Initial galvanostatic charge-discharge curves (Fig. 2a) were recorded at 0.1 C (1 C = 250 mA/g; 0.089 mA/cm^2^) with voltage cutoffs of 1.88–3.98 V (vs. LiIn, equivalent to 2.5–4.6 V vs. Li/Li^+^). SSBs employing S@LRMO (SSB-S@LRMO) achieved a specific discharge capacity of 220.2 mAh g^−1^ with an ICE of 84.83% (Fig. 2a), showing improved electrochemical performance compared with SSB-LRMO, delivering a specific discharge capacity of 138 mAh g^−1^ and an initial Coulombic efficiency of 75.46%. Moreover, SSB-S@LRMO exhibited comparable rate performance, achieving reversible capacities of 219.38, 202.74, 180.56, 144.96, and 106.39 mAh g^−1^ at 0.1, 0.2, 0.5, 1, and 2 C, respectively, consistently exceeding SSB-LRMO by approximately 70 mAh g^−1^ across all C-rates (Fig. 2b, Fig. S18). Long-term cycling tests further demonstrated the advantages of S@LRMO. At 0.2 C, SSB-S@LRMO retained 95.6% of its initial capacity after 100 cycles, compared to 83.1% for SSB-LRMO (Fig. 2c and Fig. S18). Even under prolonged cycling at 1 C (Fig. 2g), SSB-S@LRMO maintained a high specific capacity (~147 mAh g^−1^) and exhibited comparable cycling stability, retaining >97% of its initial capacity after 600 cycles, which shows competitive performance compared with previous reported results for SSBs utilizing LRMO coated with materials such as Li_3_PO_4_^9^, Li_2_NbO_3_^23^, Li_2_SO_3_^8^, Li_2_WO_4_^10^ and Li_2_B_4_O_7_^24^ (Fig. 2e and Table S4).

In contrast, pristine LRMO positive electrodes experience significant voltage decay during extended cycling due to structural and microstructural degradation^25^. Mid-point potential is a crucial parameter for assessing redox reaction reversibility. After 500 cycles at 1 C, SSB-S@LRMO retained 86.8% of its initial mid-point potentials (Fig. 2d and Fig. S19), likely caused by increased resistance after initial cycles.

To further assess the practical applicability of S@LRMO, cells with a high areal mass loading of 15.28 mg cm^−2^ were evaluated. At 0.1 C, these cells exhibited a reduced discharge-specific capacity of approximately 174 mAh g^−1^, which is likely attributable to kinetic limitations of the LRMO positive electrode and its secondary-particle morphology (Fig. S21). Despite the lower capacity, the cells demonstrated stable long-term cycling performance, indicating that the engineered electrode–electrolyte interface remains stable during prolonged cycling even at high mass loading. And evaluation of the proposed strategy in practical pouch-cell architecture will be addressed in future work.

To investigate the origins of enhanced electrochemical performance, Cyclic voltammetry (CV) was conducted (Fig. 2f). Oxidation peaks below ~4.4 V (vs. Li^+^/Li) were attributed to the oxidation of TM ions, including Co^3+/4+^ and Ni^2+/3+/4+,^ corresponding to Li^+^ removal from octahedral sites in the LiTMO_2_ structure, with the reverse process occurring during discharge^26^. The SSB-S@LRMO cell displayed reduced polarization, with the voltage difference decreasing from 0.265 V to 0.209 V (vs. Li^+^/Li) during charge and discharge cycles. Additionally, a significant increase in the intensity of the oxidation peak near 4.5 V (vs. Li^+^/Li) was observed, indicating higher capacity utilization compared to SSB-LRMO. This aligns with the extended potentials plateau near 4.5 V (vs. Li^+^/Li) in the charge-discharge profiles (Fig. 2a). The combination of reduced polarization and enhanced capacity utilization suggests improved cationic redox reversibility, attributed to the S-containing species coating on LRMO.

### Interfacial stability and transport

**Fig. 3:**
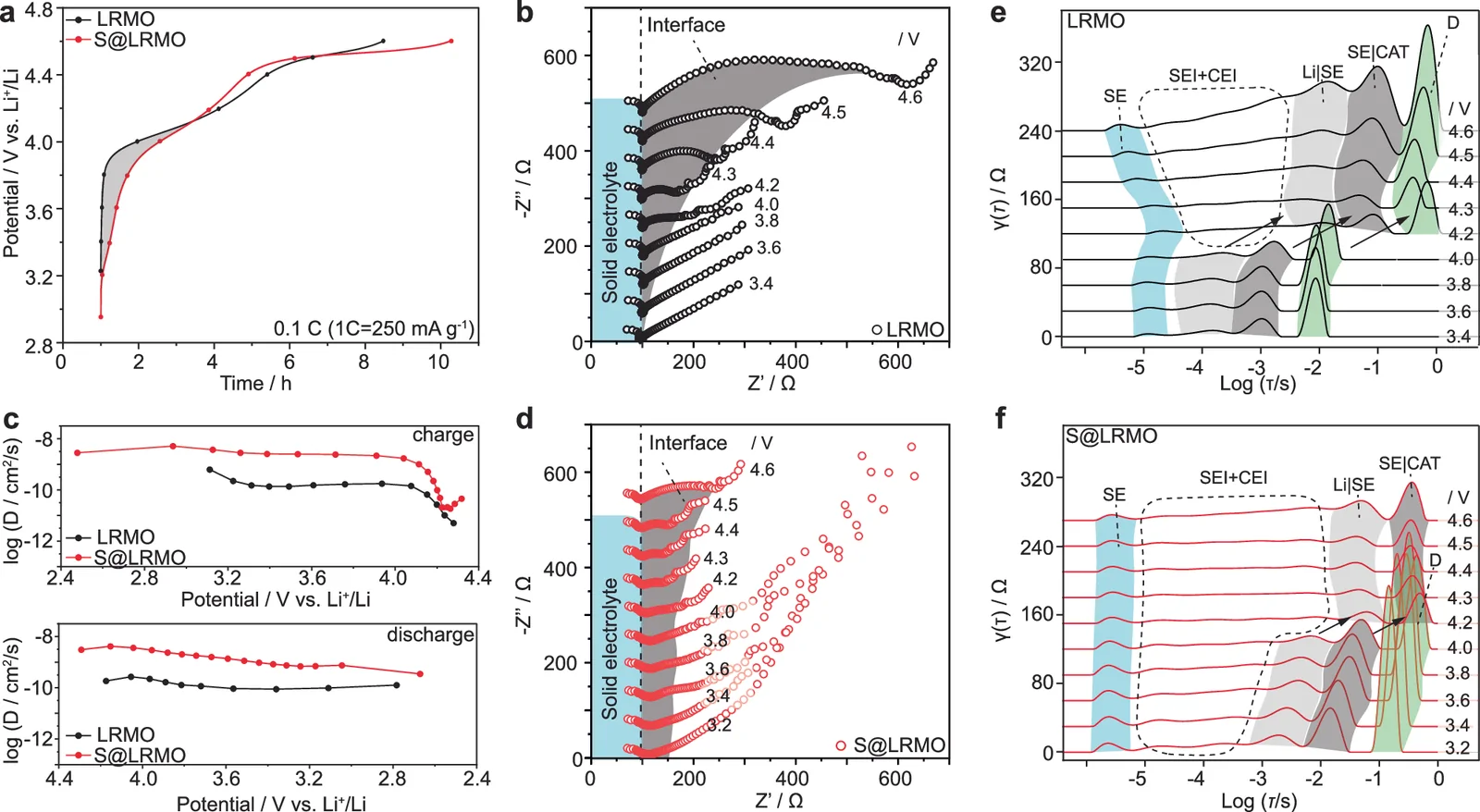
Interfacial Li^+^ kinetics evolution between positive electrode SE during the initial charging process. **a** Initial charge potential curves of the SSB-LRMO and SSB-S@LRMO cells at 0.1 C at 25 ± 2 °C;  In-situ GEIS spectra collected during the initial charging process for SSB-LRMO (**b**) and SSB-S@LRMO (**d**) cells and their corresponding DRT profiles (**e**, **f**) based on the transformation of galvanostatic electrochemical impedance spectroscopy (GEIS) results; (**c**) Calculated lithium ion (Li^+^) diffusion coefficients obtained via galvanostatic intermittent titration technique (GITT) for LRMO-based and S@LRMO-based cells during charging and discharging at 0.05 C. (1 C = 250 mA g^−1^).

To assess the impact of surface coating on transport kinetics at the SE|LRMO interface during initial charging, in-situ GEIS was performed. The impedance spectra for SSB-LRMO and SSB-S@LRMO at characteristic potentials (Fig. 3a) are presented in complex plane plots (Fig. 3b, d). The spectra reveal a high-frequency semicircle, an increasingly prominent depressed semicircle in the mid-frequency region with rising potentials, and a low-frequency tail. The high-frequency feature corresponds to the total resistance of the solid electrolytes (LICF and LPSCl), while the low-frequency tail is associated with diffusion processes within the electrode. Interpretation of the mid-frequency region is more complex due to the presence of multiple overlapping processes which result in a depressed semicircle.

To deconvolute and quantify these overlapping processes, we employed a model-independent DRT analysis, which resolves impedance spectra into discrete relaxation processes without relying on predefined equivalent circuit models^27^. The calculated peaks in the DRT spectrum (Fig. 3e, f) correspond to distinct transport and interfacial phenomena within the solid-state battery. Specifically, the high- frequency peak is attributed to ionic transport within the solid electrolyte, while the subsequent peak corresponds to ionic transport across the heteroionic LICF | LPSCl interface. In the mid-frequency region, two distinct peaks emerge, corresponding to charge transfer resistance at the SE | negative electrodes and SE | positive electrode interfaces. The low-frequency peak is assigned to Warburg-type resistance, indicative of diffusion-limited transport within the electrode materials^27,28^.

During the initial charging of the SSB-LRMO cell, the solid electrolyte resistance increases slightly (30–35 Ω at 4.6 V, Fig. S22), with no detectable contributions from cathode electrolyte interphase (CEI) or solid electrolyte interphase (SEI) formation below 4.3 V (vs. Li^+^/Li) (Fig. 3e). At higher potentials, CEI/SEI-related peaks emerge, accompanied by a pronounced increase in charge transfer impedance, leading to interfacial resistance reaches to 203.4 Ω at 4.6 V (vs. Li^+^/Li) (Fig. S22). This suggests the formation of Li-ion blocking species at the LICF|LRMO interface due to interfacial reactions.

By contrast, the SSB-S@LRMO cell exhibits additional minor peaks at a low state of charge (SOC) in the 10^−4^ to 10^−3 ^s range, which we attribute to the formed coating on LRMO particles that introduces an additional resistive contribution. However, the resistance decreases to 28.6 Ω at 4.3 V (vs. Li^+^/Li) and then increases to 67.2 Ω at 4.6 V (vs. Li^+^/Li), lower than in the uncoated system (203.4 Ω) (Fig. S22).

Additionally, impedance data recorded after 200 cycles revealed a substantial total cell resistance increase for the SSB-LRMO cell, reaching to 1258 Ω, while the cycled SSB-S@LRMO cell exhibited a much smaller increase to 382 Ω (Fig. S23). These results underscore the efficacy of the coating layer in preserving interfacial stability and enhancing Li- ion transport over extended cycling.

Moreover, to evaluate if the cross-diffusion and phase transformation in the near- approximate to the LRMO particle surface and its potential implication on Li-ion mobility and kinetics, GITT measurements were conducted at 0.05 C with a relaxation time of 1 h during the initial cycle. The corresponding Li^+^ diffusion coefficient (*D*(Li^+^)) values for both, LRMO and S@LRMO as a function of the SOC are shown in Fig. 3c. At low SOC, a slight decrease in *D*(Li^+^) is observed. However, as the voltage increases to the high-SOC region (above 4.4 V (vs. Li^+^/Li)), a pronounced decline in D(Li^+^) occurs, indicating limited kinetics attributed to structural rearrangement during the activation of the Li_2_Mn_2_O_3_ phase. In contrast, S@LRMO exhibits higher D(Li^+^) values at high SOC compared to LRMO, likely due to eliminated interfacial reactions at the LICF|LRMO interface and reduced irreversible phase transitions induced by oxygen release at high potential. Additionally, the presence of spinel-type Mn-rich species provides three-dimensional Li^+^ pathways at the interface, bypassing the restricted two-dimensional diffusion in the bulk layered structure, which facilitates faster interfacial Li^+^ transport and enhances overall electrochemical performance.

### Origin of improved cell performance

To elucidate the origin of the improved performance of S@LRMO, we employed a comprehensive suite of *operando/* in situ and ex situ characterization techniques, including ex situ XPS, *operando* sXRD, and ToF-SIMS.

The higher specific capacity of LRMO positive electrodes compared with conventional layered oxide positive electrodes (e.g., LiCoO_2_ and LiNi_x_Mn_y_Co_z_O_2_) originates from the additional contribution of oxygen redox reactions. However, the formation of O⁻/O_2_^2^⁻ species and, at high potentials, the possible evolution of O_2_ can induce structural changes in LRMO, which may oxidize solid electrolytes at the positive electrode–electrolyte interface, generate Li^+^-blocking species, and ultimately lead to sluggish reaction kinetics. Consequently, the reversibility of oxygen redox has generally been considered detrimental to the structural stability and Li^+^ transport properties of LRMO positive electrodes^10^.

To investigate the stability of oxygen redox reactions in LRMO and S@LRMO positive electrodes, O 1 *s* XPS spectra were collected for both materials in the delithiated and lithiated states. Quantitative analysis reveals that the fraction of O⁻/O_2_^2^⁻ species is significantly higher in S@LRMO, reaching 37.3% at 4.6 V and 26.5% at 2.5 V, compared with 24.3% and 15.9%, respectively, for pristine LRMO (Fig. S25). These results indicate enhanced oxygen redox stability in the S@LRMO positive electrode. This improvement is likely associated with the presence of sulfur-containing surface species and the spinel phase, which stabilize lattice oxygen through a surface phase transformation.

**Fig. 4:**
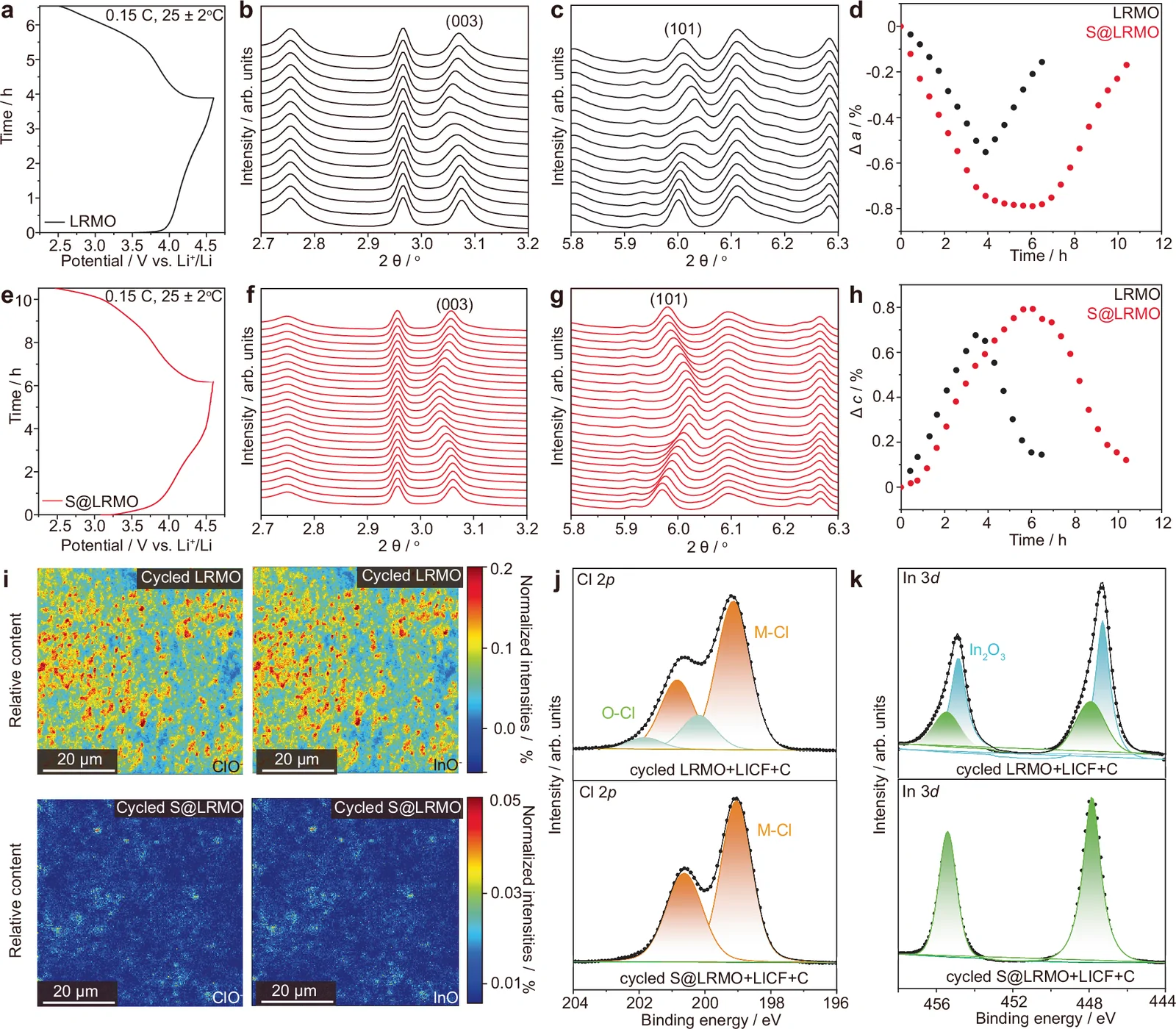
*Operando* sXRD characterization during the first charge/discharge and post-mortem physicochemical characterizations. *Operando* sXRD data was collected from SSB-LRMO and SSB-S@LRMO cells in the initial cycle at 0.15 C at 25 ± 2 °C. Galvanostatic charge-discharge curve for LRMO (**a**) and S-LRMO (**e**) based SSBs in the initial cycle. Selected areas *operando* sXRD patterns of corresponding to (003) and (101) peaks for LRMO (**b**, **c**) and S-LRMO (**f**, **g**) based SSBs; Relative changes of lattice parameters containing *a* (**d**) and *c* (**h**) of SSB-LRMO and SSB-S@LRMO during the initial cycle. Note that values are the statistically averaged results of many particles inside the *operando* cells; (**i**) Time-of-flight secondary ion mass spectrometry (ToF-SIMS) graph of ClO^-^ and InO^-^ for LRMO composites and S@LRMO composites after 200 cycles at 0.2 C (1 C = 250 mA g^−1^); (** j, k**) XPS spectra of Cl 2*p* and In 3*d* for LRMO composites and S@LRMO composites after 200 cycles at 0.2 C (1 C = 250 mA g^−1^) (positive electrode composites are collected at fully discharged state at Ar-filled glovebox).

To directly probe the associated structural evolution, *operando* sXRD patterns were collected during the initial cycles in SSB-LRMO and SSB-S@LRMO. Notable shifts in the (003) and (101) reflections were observed for both cells during the first cycle. Specifically, the (003) peak shifted to lower 2θ values, while the (101) peak shifted to higher values during charging, reflecting an increase in the *c*-lattice parameter (Fig. 4d, h). A shoulder emerged in both (003) and (101) reflections at higher potentials (>4.4 V (vs. Li^+^/Li)) in the SSB-LRMO cell, indicating oxygen loss and Mn migration from the TM layers, accompanied by spinel-like phase formation^29,30^. This irreversible oxygen loss likely contributed to the oxidation of LICF, forming non-conducting species and reducing reversible capacity. In contrast, the shoulders were absent in the (003) and (101) reflections of the S@LRMO cell, indicating that the coating effectively stabilized the structure under high potentials (>4.4 V (vs. Li^+^/Li)). Furthermore, the lattice parameters (*a* and *c*) of S@LRMO evolved linearly during charging up to 4.4 V (vs. Li^+^/Li), maintaining stability beyond this potential—a behavior absent in LRMO cells, where non-linear changes occurred (Fig. 4d, h). These observations align with the initial charge behavior shown in Fig. 2a and further confirm the coating’s role in suppressing irreversible structural changes.

Ex situ XPS and TOF-SIMS analyses provided further insights into interfacial stability. The Cl 2 *p* spectra of LICF in cycled SSB-LRMO revealed two new peaks at 200.1 eV and 201.8 eV, attributed to Cl-O bond formation (Fig. 4j) ^31^. In addition, new features at 453.5 eV and 446.0 eV correspond to In_2_O_3_^32^, arising from oxidation of the solid electrolyte induced by reactive oxygen species (Fig. 4k). In contrast, these degradation-related signals are absent in the cycled SSB-S@LRMO samples, confirming the effective protective role of the surface coating against oxygen-driven interfacial degradation. Post-mortem ToF-SIMS analysis corroborated these findings, showing InO⁻ and ClO⁻ fragments in SSB-LRMO after 200 cycles, indicative of LICF oxidative decomposition triggered by LRMO phase transitions. In contrast, SSB-S@LRMO exhibited minimal InO⁻ and ClO⁻ signals, reflecting superior interfacial stability conferred by the coating (Fig. 4i).

To verify the persistence of the sulfur-containing surface coating and the spinel phase on LRMO particles after prolonged cycling, post-mortem analysis based on XPS and TEM was conducted for S@LRMO positive electrode. XPS measurements detect sulfur-containing species on the cycled electrodes, with binding energies consistent with SO_4_^2−^ (Fig. S29a). This observation is further corroborated by TEM analysis with spinel remaining on the surface of LRMO particle (Fig. S29b). Collectively, these results demonstrate that the thin coating and surface spinel phase maintained their structural integrity during long-term electrochemical cycling.

**Fig. 5:**
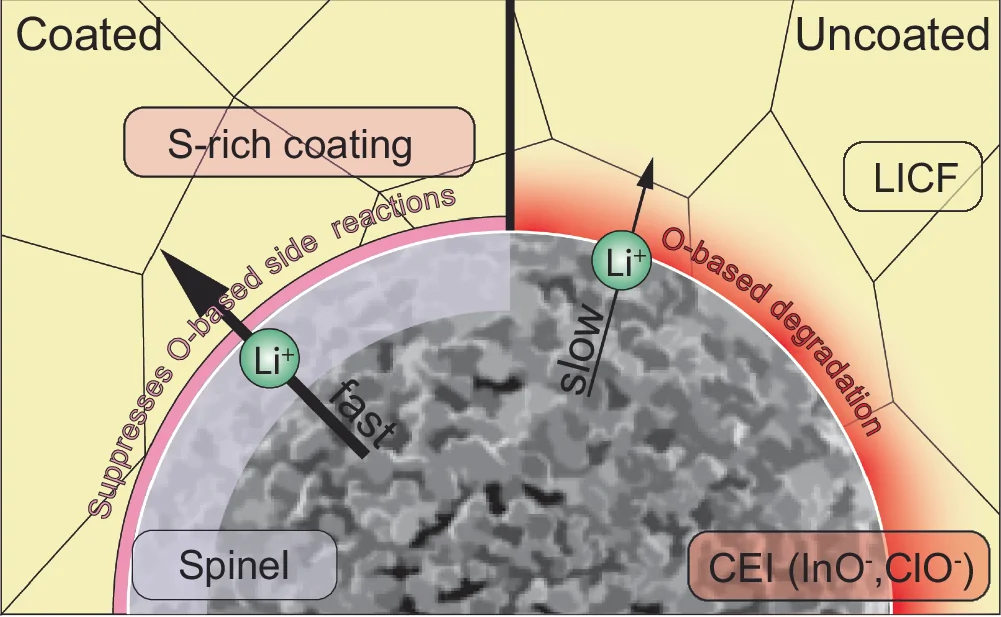
Schematic illustration of the thiourea-derived coating strategy. The thiourea-derived coating suppresses oxygen-related interfacial degradation and facilitates Li^+^ transport through the surface spinel layer, resulting in improved cycling stability and rate capability of the LRMO positive electrode.

To further substantiate the hypothesis that the spinel LiMn_2_O_4_ surface layer mitigated degradation by suppressing oxygen loss, density functional theory (DFT) calculations were performed to compare the energetics of oxidized anionic species formation in LRMO and spinel LiMn_2_O_4_ (Supplementary Data 1). An interface model comprising LRMO in the (104) orientation and spinel LiMn_2_O_4_ in the (111) orientation was constructed to enable a direct comparison of peroxide bond formation energetics (Fig. S30). This interface provides a common thermodynamic reference for delithiation-driven anionic redox processes.

Peroxide bonds were introduced at multiple sites within the bulk-like regions of both the LRMO and spinel slabs, accompanied by the removal of two Li atoms from the LRMO side to initiate anionic redox. Importantly, even when peroxide bonds were introduced in the spinel layer, the delithiation origin remained on the LRMO side, ensuring consistent comparison of total energies between the two phases.

Because peroxide bonds were introduced in bulk-like layers rather than at the immediate interface, the calculated relative energetics are not expected to be sensitive to the precise interface structure or surface facet selection. Although Li/vacancy configurations can influence peroxide stability, this effect was isolated by using a common Li/vacancy arrangement derived from a reference LRMO structure containing a peroxide bond. This configuration served as the initial state for peroxide formation in the spinel LiMn_2_O_4_ layer, while allowing full ionic relaxation.

As summarized in Table S5, peroxide formation is significantly less favorable in spinel LiMn_2_O_4_ than in LRMO. The most stable peroxide configuration in the spinel layer is 2.59 eV higher in energy than the lowest-energy configuration in the layered structure. Moreover, peroxide stability exhibits strong dependence on the local chemical environment, with energy spreads of approximately 2.3 eV and 3.9 eV for LRMO and spinel LiMn_2_O_4_, respectively.

These results demonstrate that the formation of oxidized anionic species in the form of peroxides is intrinsically unfavorable in spinel LiMn_2_O_4_, even when interfaced with LRMO and when anionic redox is initiated by over-delithiation of the layered phase. Consequently, the migration of oxidized anionic species through the spinel surface layer would require energetically prohibitive stabilization within the spinel lattice. This energetic barrier effectively suppresses oxygen migration to the surface and prevents oxygen loss, in strong agreement with the experimental observations.

To summarize, in this study we demonstrated a thiourea-derived surface modification strategy for LRMO positive electrodes, enabling significant improvements in electrochemical performance for SSBs. By integrating a thiourea-derived coating, we achieved a dual effect: the formation of a stable interphase that mitigates interfacial degradation through avoiding the physical contact between LRMO and SE and a surface lattice reconstruction into spinel phase that dominantly weakens oxygen loss and TM ion migration through its stable surface lattice as well as enhances Li-ion transport via its three-dimensional Li⁺ pathways (the functionality of the coating is summarized in Fig. 5). The modified S@LRMO positive electrode exhibited a high initial discharge capacity of 220.2 mAh g^−1^, a Coulombic efficiency of 84.83%, and improved cycling stability, retaining 97% capacity after 600 cycles at 1C under a high cutoff voltage of 4.6 V (vs. Li⁺/Li). *Operando* synchrotron X-ray diffraction and ex-situ spectroscopic analyses confirmed that the sulfur modification effectively blocked oxygen-related degradation and stabilized the LRMO structure during high-voltage cycling. Furthermore, the sulfur-induced formation of a spinel-like surface phase created a stable cathode-electrolyte interface and facilitated lithium-ion diffusion, improving both rate capability and long-term cycling stability. These findings establish a scalable and effective approach to overcoming the intrinsic challenges of LRMO positive electrodes in solid-state batteries. By suppressing oxygen involving reactions, stabilizing interfacial kinetics, and enhancing lithium-ion transport, this work provides fundamental insights into cathode material design and offers a promising route toward high-energy-density, long-cycle-life solid-state batteries. 

## Methods

### Materials synthesis

#### S@LRMO

2 g commercial LRMO (Ningbo Lanli Energy Technology Co., Ltd.) was dispersed in Thiourea (99%, ThermoFisher)-containing deionized water (0.04 g in 30 mL) in a 100 mL conical flask. Then the mixture was stirred under 500 rpm for 20 h. Next, the mixture was agitated at 80 °C until converted into a dry slurry with evaporation of water and then transferred into a muffle furnace for annealing under 450 °C for 4 h with 2 °C/min temperature increasing/decreasing rate. In the end, the Thiourea coated LRMO sample was obtained and further labeled as S@LRMO.

#### LICF synthesis

Li_3_InCl_5.4_F_0.6_ was prepared via a two-step synthesis process: 1 > lithium chloride (LiCl, Sigma-Aldrich, 99.9%), indium chloride (InCl_3_, Sigma-Aldrich, 99.99%) and indium fluoride (InF_3_, Sigma-Aldrich, 99.5%) were weighed to the stoichiometric molar ratio (15:4:1) and mechanically mixed in a ZrO_2_ container with ZrO_2_ balls (diameter = 5 and 10 mm, 1:1, wt %) (m (balls): m(powder) = 30:1) in a planetary ball mill, sealing at Ar-filled glovebox (H_2_O < 0.1 ppm, O_2_ < 0.1 ppm), (Fritsch, Pulverisette 7) at 500 rpm for 24 h (10 min running + 10 mins rest, repeating for 24 times); 2 > the ball milled Li_3_InCl_5.4_F_0.6_ powder was pelletized at 300 MPa in a 10 mm stainless steel die set, further annealed at 300 °C with a temperature increasing/decreasing rate of 2 °C/min for 5 h in Ar atmosphere and the annealed pellet are grinded with mortar and pestle to get LICF powder.

#### LiIn alloy fabrication

In foil and Li foil are pressed together with a weight ratio of 98: 2 for 2 h. and the pressed foil are further calendared several times to get homogeneous alloy with 200 μm thick. Then the obtained foil is cut into several 10 mm round disks. The whole process is conducted at Ar-filled glovebox.

### Materials characterization

#### XRD

The XRD patterns were collected in a transition mode at beamline SNBL-BM31 at the European Synchrotron Radiation Facility (ESRF) (Swiss-Norwegian Beamline, European Synchrotron Radiation Facility, Grenoble, France) using a monochromatic high brilliant X-ray beam with *λ* = 0.254480 Å. The diffractometer is based on a Pilatus2M detector and data processing, and integration was done using the BUBBLES software^33^. Detector position calibration was achieved by measuring a standard silicon reference powder and employing the calibration module provided by the pyFAI software package. Powder samples of cell components (LRMO, S@LRMO, LPSCl, LICF) were loaded into 0.3 mm glass capillaries for pre-characterization and for refining the crystal structures for subsequent multi-component *operando* measurements^33^. Synchrotron X-ray diffraction data were evaluated using the Rietveld technique as implemented in the TOPAS V6 program. In all refinements, the background was modelled using a Chebychev fifth-order polynomal function. The amorphous components arising from the electrochemical cell (or the capillary, see below) were modelled using pseudo-Voigt-shaped single peaks. Number, position and shape of these were extracted from measurement on the empty capillaries (for the measurements on the individual cell components) or from the calibration measurement of the empty cell with the silicon standard inside. For the crystalline phases, the peak shape was modeled with a Thompson-Cox-Hastings modified pseudo-Voigt function (PV-TCHZ) with six refinable parameters. For getting good starting models and checking for any structural changes during coating, LRMO and S@LRMO were investigated in detail using sXRD data obtained from capillary measurements with high q-values. In the as-purchased LRMO sample, the dominating phase is LRMO, which shows trigonal R3 ®mH symmetry. In structure refinement, the *B*-values of all symmetry non-equivalent atoms were refined to reliable values. For both, the Li1 (3a) and the M2 (3b) sites, full site occupation was assumed. For the 3a site, the site occupancy was refined as Li + Mn = 1, while for the 3b site the Ni and Co contents were fixed to the values, provided by the supplier (0.13 apfu each) and the site occupancy was refined thus as Li + Mn = 0.74. It is not possible to determine the content of Mn, Co and Ni independently from XRD data. The atomic displacement parameters *B* were refined isotropically and were constrained to be the same for all elements on the same non-equivalent sites, i.e. Li + Mn on the Li1 site have the same *B*-value, the same accounts for the cations on the 3b (TM) site, but they were allowed to refine freely for the distinct site. Results of the structure refinement are given in Fig. S2 and Tables S1 and S2. Besides domination LRMO (93.2(8) wt %), two additional phases were identified, namely spinel type LiMn_2_O_4_ (cubic, Fm3 ®mS with 2.8(9) wt %) and Li_2_MnO_3_ (monoclinic, C2/m, 4.0(9) wt %). The later phase shows some asymmetric broadening at the high angle side of some Bragg peaks, which can be interpreted as resulting from distinct stacking faults; due to the low amount of this phase no further information is extractable. For the S@LRMO sample the same refinement strategies were applied. The structural parameters for the main phase show only very minor changes, which are a result of the thermal treatment (slight increase in anti-site defects and thus a small change in lattice parameters), rather than due to the coating itself. Results of structure refinements are given in Fig. S2 and Tables S1 and S3. An interesting observation, however, is the increase in the amount of the spinel phase, which can be clearly seen now in the diffraction pattern as *e.g.* broad bumps at d-values of 2.91 Å, (2 2 0) lattice plane, and 2.48 Å, (3 1 1) lattice plane. Evaluation of the peak width following procedures implemented in TOPAS yield crystallite sizes for the spinel phase of <5(1) nm, based in the integral breath of the peaks. While the amount of the spinel phase increased during coating to ~10(2) wt %, the amount of the monoclinic Li_2_MnO_3_ phase remained constant within estimated standard deviation (4.6(8) wt % in SLRMO). Evaluation of *operando* XRD data was done in a sequential mode making use of the so-called launch-mode in TOPAS. Due to their complexity and multiphase nature of XRD pattern in the *operando* measurements, the peak shape parameters of individual phases were fixed to the values obtained from the capillary measurements of individual components (LRMO, S@LRMO, LICF, LPSCl). To account for any possible changes in peak width, a Lorentzian (crystallite size) broadening was allowed in sequential refinements. However, there was no significant change in peak shape and width within the resolution of the present measurements which would point to additional size and micro strain broadening during *operando* cycling. The structural parameters (*B*-values, site occupation numbers) of LRMO/S@LRMO were constrained with only the lattice parameters and the scaling factors of the cell components being allowed to vary freely. Due to the low overall content of the positive electrode material (~4 wt %) for both series (LRMO and S@LRMO), it was not possible to extract any reliable dependence of Li-contents during cycling.

#### SEM and TEM

The morphologies of LRMO and S@LRMO were characterized by secondary electron SEM images taken at 2 kV acceleration voltage and 100 pA beam current, using a Helios G4 UX from Thermo Fisher Scientific. Lamella for transmission electron microscopy (TEM) were prepared by the same Helios G4 UX. Representative particles were first coated by carbon using e-beam assisted carbon deposition. A thicker protection layer was further deposited by ion-beam assisted deposition. The lamellae were transferred and mounted as flags on a Cu TEM grid by standard lift-out technique. All coarse thinning were performed with 30 kV acceleration voltage for the Ga^+^ ions. Final thinning was first done at 5 kV and then 2 kV on either side of the lamellae to minimize surface damage. TEM was performed with a double spherical aberration corrected, cold field emission gun JEOL ARM 200FC, operated at 200 kV. This instrument is equipped with a large solid angle (covering a solid angle of 0.98 sr) Centurio detector for energy dispersive X-ray spectroscopy (EDS) and a GIF Quantum ER spectrometer for dual EELS. Element mapping was done in STEM mode by simultaneous use of EDS and dual EELS. The Li *K*-edge and TM *M*_4,5_-edge signals were extracted from the low-loss spectra, while the O *K*-edge and TM *L*_2,3_-edges were quantified in the core-loss spectra in every pixel of the maps.

#### XPS and ToF-SIMS

Chemical characterizations of the samples and the state of the surface were obtained with X-ray photoelectron spectroscopy (XPS). Core level spectra were measured using a monochromatized Al *K*_α_ (1486.6 eV) line with a hemispherical analyzer at NTNU XPS facilities. Measurements were performed at room temperature and with chamber pressures below 10^−9^ mbar. The calibration of the binding energy was done using C 1*s* and Au 4*f* reference peaks. The peaks fitting was optimized using CasaXPS software. ToF-SIMS analysis was performed in a dual-beam plasma-FIB system (Helios 5 PFIB CXe DualBeam) with a xenon (Xe) ion source. Measurements were conducted on a 60 × 60 µm^2^ area with an acceleration voltage of 30 kV and an ion beam current of 4 nA. Data acquisition was carried out in negative ion mode at a resolution of 512 × 442 pixels with a dwell time of 10 µs per pixel and 2 × 2 binning across 100 depth frames. ToF-SIMS explorer (version 1.12.2.0) was employed for data processing, which included a mass calibration against ^115^In and ^23^Na with mass-to-charge (*m*/*Q*) ratios of 114.90 and 22.99, respectively. Cycled positive electrodes for these two ex situ tests are collected at 2.5 V (vs. Li^+^/Li) in glovebox after 200 cycles at 0.2 C at 25 ± 2 °C. Transfer chambers are used during experiments.

#### *Operando* sXRD tests

*Operando* sXRD tests of cells in through-plane direction were conducted at SNBL-BM31. The XRD experiments were done in transmission mode with 50 KeV photon energy and a beam size of 300 µm × 50 µm.

### Electrochemical measurements

#### Impedance spectroscopy

Following the assembly of the LiIn||LRMO solid-state batteries (SSBs), electrochemical impedance spectroscopy (EIS) measurements were performed over a frequency range from 7 MHz to 10 mHz. An applied alternating current (AC) amplitude of 5 mV was used, and the tests were conducted utilizing a Biologic VMP-300 electrochemical workstation. For in situ GEIS test, cells were assembled, performed over a frequency range of 7 MHz to 10 mHz with an applied amplitude of 50 μA and charged at 0.1 C via Galvanostatic Cycling with Potential Limitation (GCPL) technique. 10 points per decade were collected during measurements. Prior to each measurement, the cell was allowed to equilibrate at open-circuit voltage for 10 s to ensure quasi-steady-state conditions. The DRT analysis based on EIS spectra with mathematical transformation was conducted on RelaxIS online DRT. EIS spectra fittings were conducted on EC-Lab software.

#### Half-cell characterization

Solid-state half cell: Firstly, 50 mg LPSCl (~300 μm thick in 10 mm PEEK cell) was added in the polyaryletheretherketone (PEEK) cell with diameter of 10 mm and pressed at 3 tons; Secondly, 50 mg LICF (~300 μm thick in 10 mm PEEK cell) was added on the top of LPSCl (NEI corporation) and further pressed at 3 tons; 10 mg of a composite powder—consisting of the active material (LRMO or S@LRMO), Ketjenblack (KB, EC-600JD, Sigma-Aldrich), and LICF in a mass ratio of 60:5:40 (active materials mass loading: 7.28 mg/cm^2^ for LRMO and 7.13 mg/cm^2^ for S@LRMO, respectively)—was homogeneously distributed onto one side of the solid electrolyte pellet and pressed at 3 tons. This resulted in an active material mass loading of 7.28 mg/cm^2^ for the LRMO cells and 7.13 mg/cm^2^ for the S@LRMO cells. Subsequently, a 200 μm thick LiIn disk was attached to the opposite side of the LPSCl pellet and pressed at 1.2 tons. All SSBs were fabricated inside an argon-filled glovebox to prevent atmospheric contamination. ^Galvanostatic^ cycling tests of the cells were conducted using Neware battery test system in the potential range from 2.5–4.6 V (vs Li/Li^+^) at 150 MPa. For long-term cycling tests, cells are measured under 0.1 C for 2 cycles (activation process) and 0.2 C or 1 C for hundreds of cycles; and for rate capabilities evaluation, cells are tested under 0.1 C, 0.2 C, 0.5 C, 1 C and 2 C for 3 cycles, respectively. Besides, cells for in situ GEIS test are measured under 0.1 C during its initial charge process in a typical PEEK cell while cells for operando sXRD tests are tested under 0.15 C during its initial cycle at operando cell. The C-rate of 1 C corresponds with 250 mA/g. Coulombic efficiency=discharge capacity/charge capacity.

#### Cyclic voltammetry

CV tests were performed at a scan rate of 0.05 mV/s in the potential range of 2.5 to 4.6 V (vs. Li^+^/Li).

#### GITT analysis

GITT tests were conducted using Neware battery test system in the potential range from 1.88 to 3.98 V (vs. LiIn), which corresponds to approximately 2.5–4.6 V (vs Li/Li^+^). During both the charge and discharge processes, a constant current pulse of 0.05 C was applied for 1 h, followed by a 1 h relaxation period. The cell potential was recorded at 30 s intervals during each current pulse and relaxation step. The lithium-ion diffusion coefficient D(Li^+^) was calculated using the following standard equation:

4 $$D=\frac{4}{\pi \tau }{\left(\frac{{m}_{{{{\rm{B}}}}}{V}_{{{{\rm{M}}}}}}{{M}_{{{{\rm{B}}}}}S}\right)}^{2}{\left(\frac{\varDelta {E}_{{{{\rm{s}}}}}}{\varDelta {E}_{{{{\rm{\tau }}}}}}\right)}^{2}$$

Here, τ specifies the duration of the relaxation step. The variables ${m}_{B},{V}_{M}$ and ${M}_{B}$ correspond to the mass, molar volume, and molar mass of the active component, respectively. Additionally, *S* designates the effective contact area at the cathode/electrolyte interface. The *ΔE*_τ_ describes the transient potential shift during the current pulse (corrected for the IR drop), whereas *ΔE*_s_ represents the overall steady-state potential difference observed upon completion of the relaxation interval.

All the SSB cells are tested at room temperature (25 ± 2 °C) without using environmental chamber.

### DFT simulations

#### Computational details

Periodic first-principles calculations based on DFT were performed to compare the formation of peroxide bonds as the early stages of anionic redox in LRMO and spinel LiMn_2_O_4_^34,35^. The projector augmented wave method (PAW) was used in conjunction with the Perdew–Burke–Ernzerhof (PBE) exchange-correlation functional as implemented in the Vienna Ab initio Simulation Package (VASP)^36–40^. Hubbard U corrections of U(Ni) = 5.6 eV, U(Mn) = 3.9 eV, and U(Co) = 5.0 eV were applied to treat the strongly correlated 3*d*-electrons of Ni, Mn, and Co^41–43^. The plane-wave cutoff was set to 520 eV and the Brillouin zone sampling was restricted to the Gamma point due to the large size of the interface model. The electronic convergence criterion was 1 × 10⁻^5^ eV. Bulk structures were relaxed until all forces converged below 0.01 eV Å⁻^1^, while a threshold of 0.05 eV Å⁻^1^ was used for the interface structures. The interface model was constructed by joining LRMO in the (104) orientation with spinel LiMn_2_O_4_ in the (111) orientation. These surface facets are among the lowest in energy and are thus expected to be present under experimental conditions^43–45^. The model was created as follows: First, the structure obtained from XRD refinement was relaxed, with Ni, Mn, Co, and Li atoms randomly distributed on the transition metal and Li sites to match the experimental composition. Second, surface slabs for LRMO (104) and spinel (111) were generated with 7 and 11 layers, respectively, and optimized by keeping three layers fixed while allowing the remaining layers to relax. A vacuum layer of 20 Å was introduced to avoid spurious interactions between periodic images. Finally, the interface was assembled by pre-relaxation to determine the optimal separation between the two slabs.

## Supplementary information

Supplementary Information

Description of Additional Supplementary Files

Supplementary Data 1

Transparent Peer Review file

## Source data

Source Data - Supplementary Information

Source Data - Main Manuscript

## Data Availability

The data generated and analyzed in this study are provided within the paper and its Supplementary Information. All additional information is available from the corresponding authors. Source data are provided with this paper. DFT input and output files are provided as Supplementary Data 1. Source data are provided with this paper.
