# Supplementary material for: Thiourea-derived coating enabled lithium-rich manganese oxide positive electrode in solid-state batteries
Source: Nat Commun. 2026 Jul 15;17:8706. doi: 10.1038/s41467-026-75215-1 (PMC13490549; doi:10.1038/s41467-026-75215-1)
Supplement: Supplementary file 1 [file 41467_2026_75215_MOESM1_ESM.pdf]

Supplementary Information for

Thiourea-Derived Coating Enabled Lithium-Rich Manganese Oxide Positive Electrode in Solid-State Batteries

Feng Jin[1,2], Wenguang Zhao[2], Ingeborg Sellæg Ellingsen[1], Henrik Rotvær Bratlie[1], Quoc Hung Nguyen[1], Dragos Stoian[3], Kenneth Marshall[3], Wouter van der Beek[3], Per Erik Vullum[4], Manuel Dillenz[5], Jose Maria Castillo Robles[5], Juan Maria Garcia Lastra[5], Ivano Eligio Castelli[5], Feng Pan[2], Günther J. Redhammer[6], Daniel Rettenwander[1,7,8,9*]

[1]Department of Material Science and Engineering, NTNU Norwegian University of Science and Technology, Trondheim, Norway

[2]School of Advanced Materials Shenzhen Graduate School, Peking University, Shenzhen 518060, PR China

[3]Swiss-Norwegian Beam Lines at European Synchrotron Radiation Facility, 38043 Grenoble, France

[4]Sintef Industry, Trondheim 7034, Norway

[5]Department of Energy Conversion and Storage, Technical University of Denmark, Agnes Nielsens Vej 301, DK-2800 Kongens Lyngby, Denmark

[6]Department of Chemistry and Physics of Materials, University of Salzburg, Salzburg 5020, Austria

[7]Christian Doppler Laboratory for Solid-State Batteries, NTNU Norwegian University of Science and Technology, Trondheim 7491, Norway.

[8]AIT Austrian Institute of Technology GmbH, Center for Transport Technologies, Battery Technologies, Vienna 1210, Austria

[9]Institute of Chemical Technologies and Analytics, Vienna University of Technology, Getreidemarkt 9/164-EC, 1060 Vienna, Austria

*Corresponding Author: daniel.rettenwander@ntnu.no

Contents

Supplementary Figures

Supplementary Figure 1. Schematic illustration of coating process.

Supplementary Figure 2. High-resolution sXRD pattern and Rietveld refinement result of LRMO and S@LRMO.

Supplementary Figure 3. SEM image of a single LRMO secondary particle.

Supplementary Figure 4. SEM images for LRMO and S@LRMO with lamella for further TEM tests.

Supplementary Figure 5. Positions of HR-TEM for LRMO and S@LRMO samples.

Supplementary Figure 6. TEM images of LRMO and S@LRMO samples.

Supplementary Figure 7. HAADF-STEM image of LRMO.

Supplementary Figure 8. HAADF-STEM image of 1% S@LRMO and 3% S@LRMO.

Supplementary Figure 9. Raman spectroscopy results for both LRMO and S@LRMO.

Supplementary Figure 10. XPS N 1*s* spectrum for LRMO (a) and S@LRMO (b).

Supplementary Figure 11. XPS O 1*s* spectrum for LRMO and S@LRMO.

Supplementary Figure 12. The XPS spectra of the Co, Ni for LRMO and S@LRMO.

Supplementary Figure 13. O 1*s*, S 2*p*, Co 2*p*, Ni 2*p* and Mn 2*p* XPS spectra for S@LRMO with / without etching.

Supplementary Figure 14. XRD patterns for coated LRMO without and with annealing process.

Supplementary Figure 15. XRD patterns of Li_3InCl_6 (LIC) and $Li_3InCl_{5.4}F_{0.6}$ (LICF).

Supplementary Figure 16. The schematic illustration of the ASSLBs.

Supplementary Figure 17. Nyquist plot of LICF at room temperature.

Supplementary Figure 18. Electrochemical performances of LRMO and coated LRMO with various coating contents.

Supplementary Figure 19. The average potential change curve for S@LRMO-based cell during 1C long-term cycling.

Supplementary Figure 20. Charge/discharge curves of different cycles for SSB-S@LRMO with current density of 1C.

Supplementary Figure 21. Rate capabilities and long-term cycling performance of S@LRMO with high mass loading.

Supplementary Figure 22. Solid electrolyte resistance (R_{SE}), SEI/CEI resistance (R_{SEI}) and charge transfer (R_{CT}) resistance changes during initial charging process of SSB-LRMO and SSB-S@LRMO.

Supplementary Figure 23. The Nyquist plots of SSB-LRMO and SSB-S@LRMO after 1 and 200 cycles with 0.2 C.

Supplementary Figure 24. Zoomed-in areas of potentia-time curves for LRMO and S@LRMO cells during GITT tests under 0.05 C and 1 h rest.

Supplementary Figure 25. The XPS spectra of O 1s in the LRMO and S@LRMO positive electrode composite at initial charging state of 4.6 V and discharging state of 2.5 V.

Supplementary Figure 26. Optical images of the operando cell and its components, including plungers, sleeve, insulator, load cell, piezo and screw.

Supplementary Figure 27. Galvanostatic charge-discharge curves for LRMO and S-LRMO based operando cells in the initial cycle.

Supplementary Figure 28. Cl 2*p* XPS and In 3*d* spectra of LICF.

Supplementary Figure 29. S 2*p* XPS spectrum of cycled S@LRMO positive electrode components and TEM image of cycled S@LRMO positive electrode particle.

Supplementary Figure 30. Initial LRMO and spinel $LiMn_2O_4$ interface model and peroxide structure.

Supplementary Figure 31. The long-term cycling of S-LRMO SSB.

Supplementary Tables

Supplementary Table S1. Crystallographic parameters of the LRMO and S@LRMO acquired from Rietveld refinement.

Supplementary Table S2. Rietveld analysis results of LRMO.

Supplementary Table S3. Rietveld analysis results of S@LRMO.

Supplementary Table S4. Comparison advanced coatings on LRMO and corresponding electrochemical performance of all-solid state lithium batteries.

Supplementary Table S5. Relative energetics of peroxides formed in LRMO and spinel $LiMn_2O_4$ layers of the interface model.

Supplementary Figures

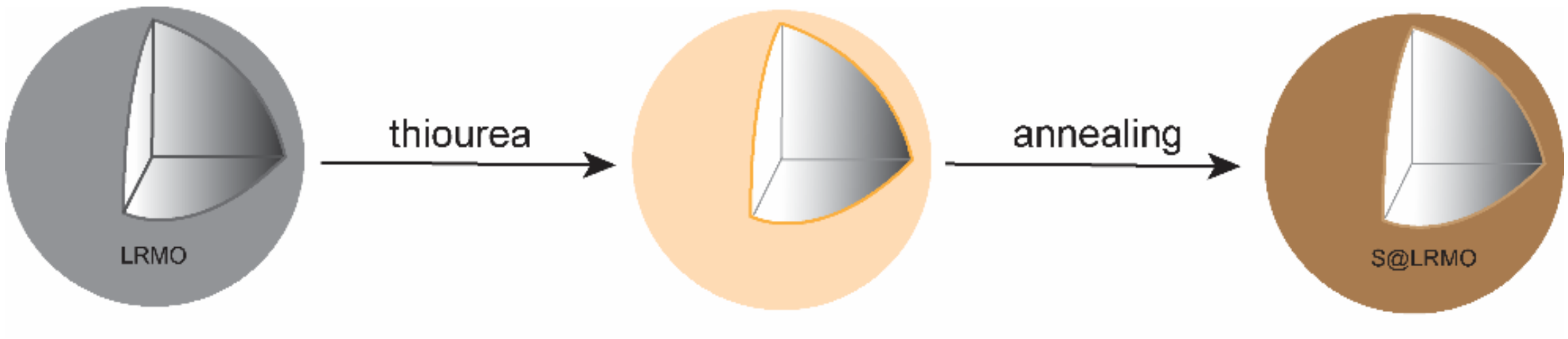

Supplementary Figure 1. Schematic illustration of coating process. The whole process contains two procedures, including liquid phase coating and subsequent annealing step.

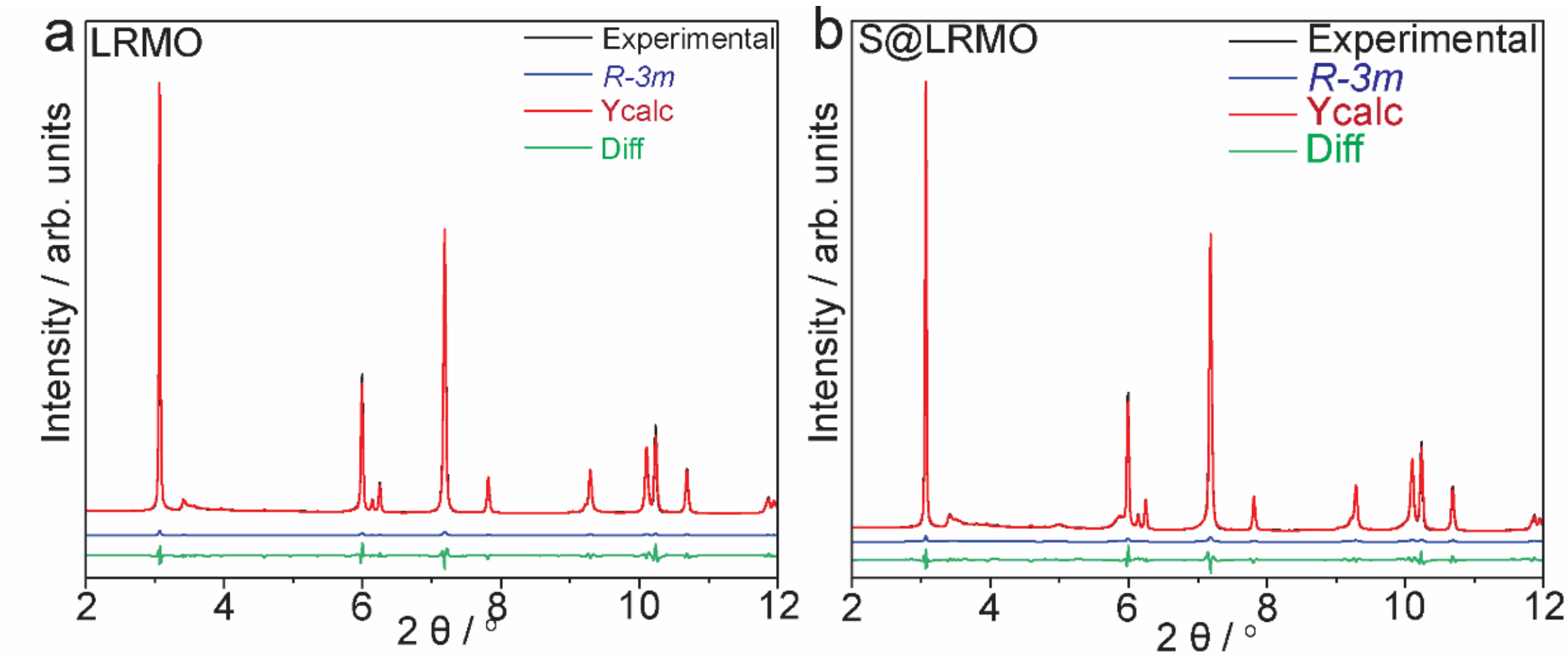

Supplementary Figure 2. High-resolution sXRD patterns and corresponding Rietveld refinement results of LRMO (a) and S@LRMO (b). Data collected at BM31@ESRF with λ = 0.54480 Å.

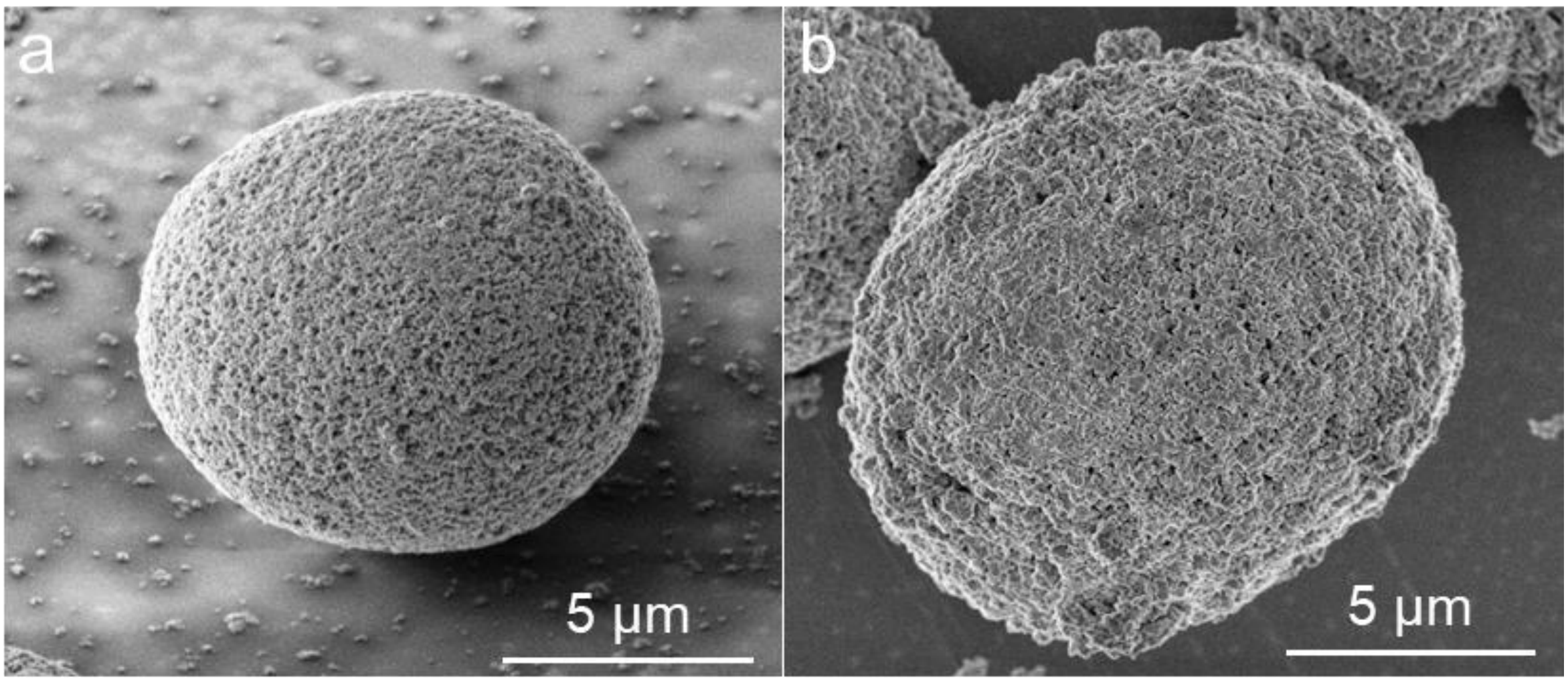

Supplementary Figure 3. SEM image of LRMO (a) and S@LRMO (b).

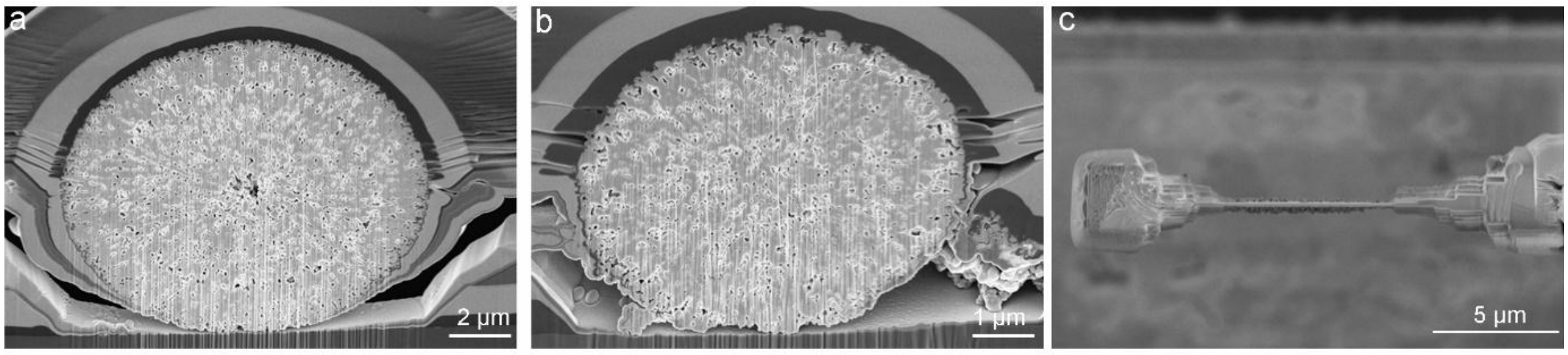

Supplementary Figure 4. SEM images for LRMO and S@LRMO with lamella for further TEM tests. Top-view SEM images for ion-beam cut LRMO (a) and S@LRMO (b). (c) cross-section SEM image of lamella LRMO (similar with S@LRMO) with a thickness at ~ 50-100 nm after cutting and cleaning for several time with Focus ionic beam.

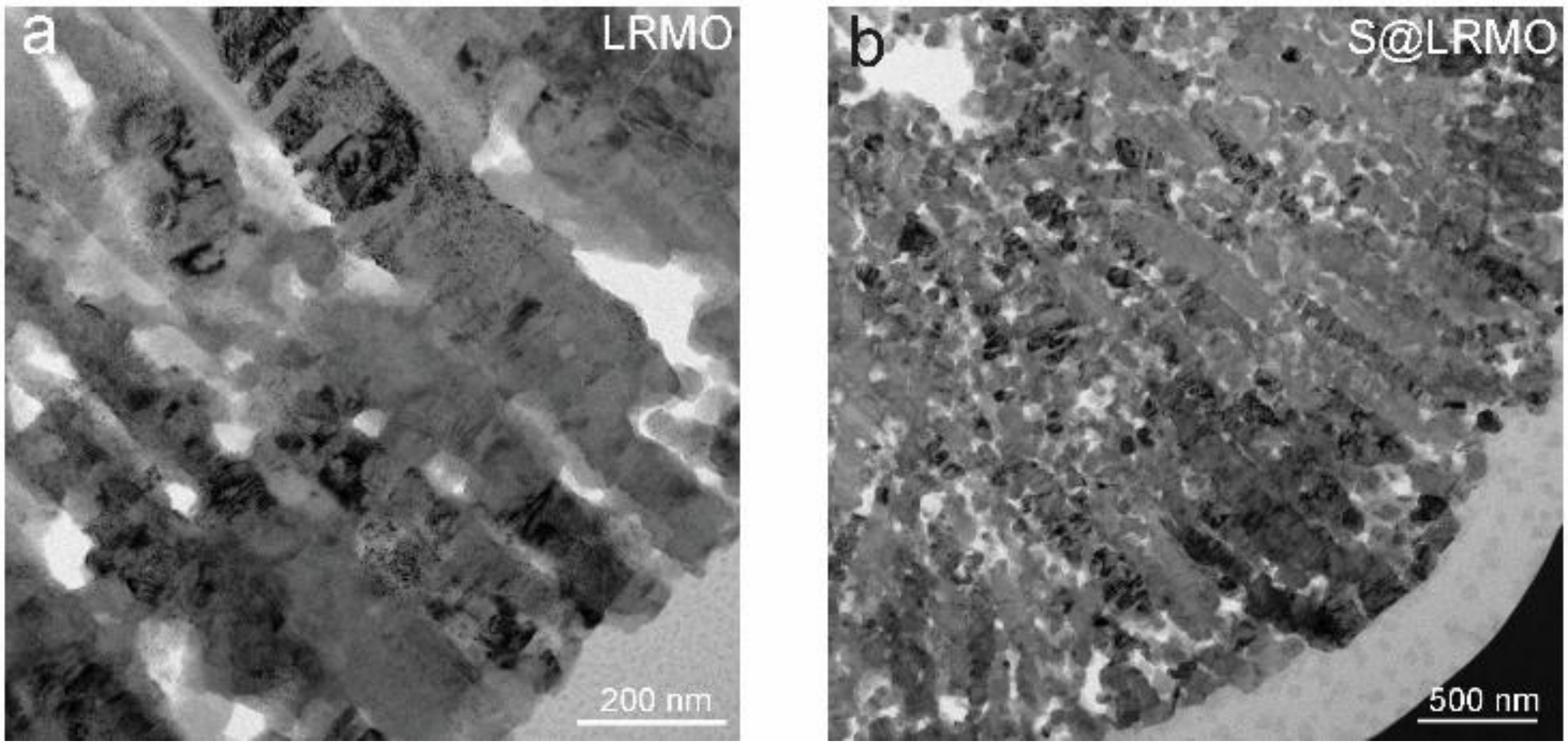

Supplementary Figure 5. Positions of HR-TEM for LRMO (a) and S@LRMO (b) samples.

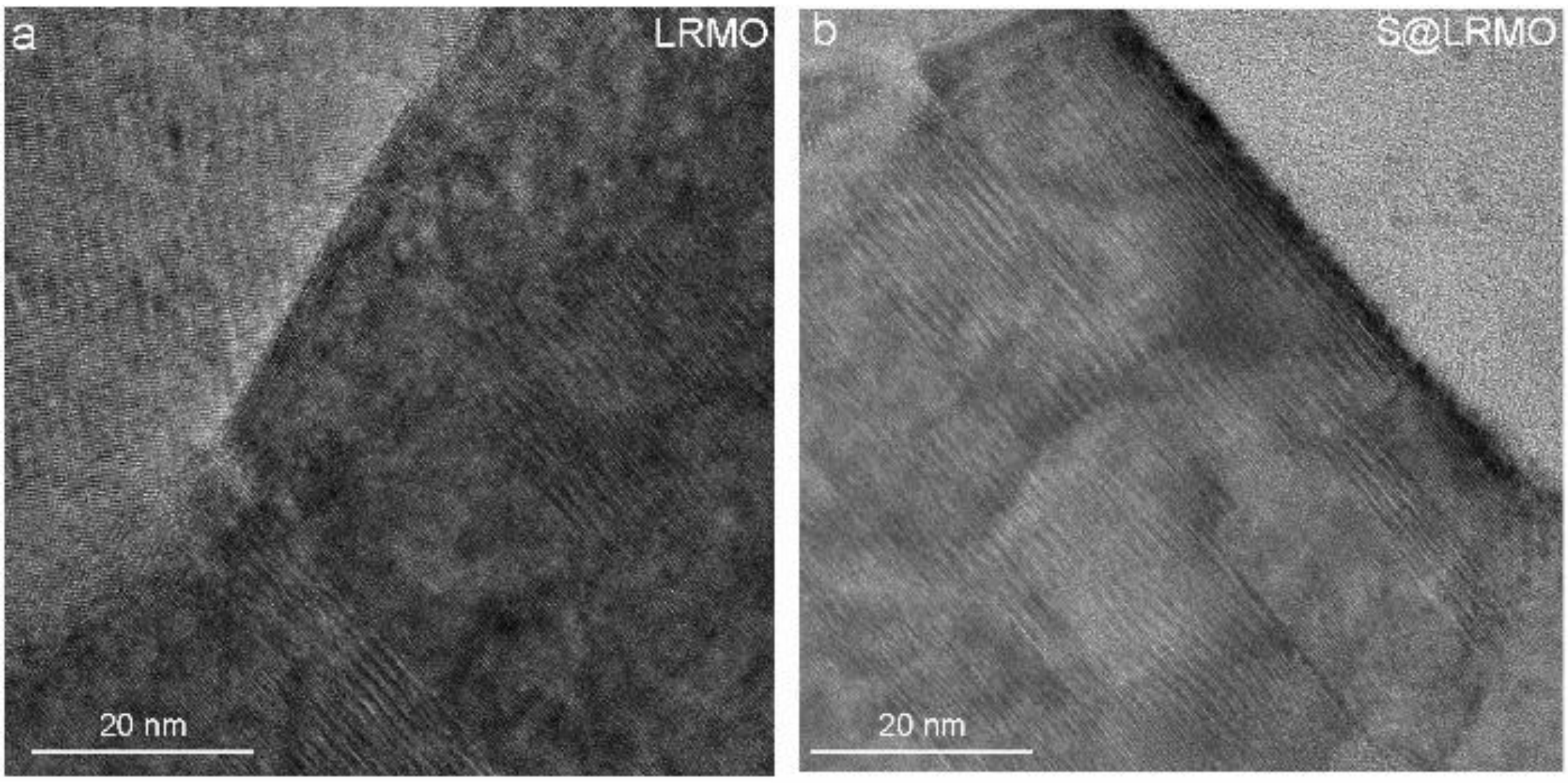

Supplementary Figure 6. TEM images of LRMO (a) and S@LRMO (b) samples.

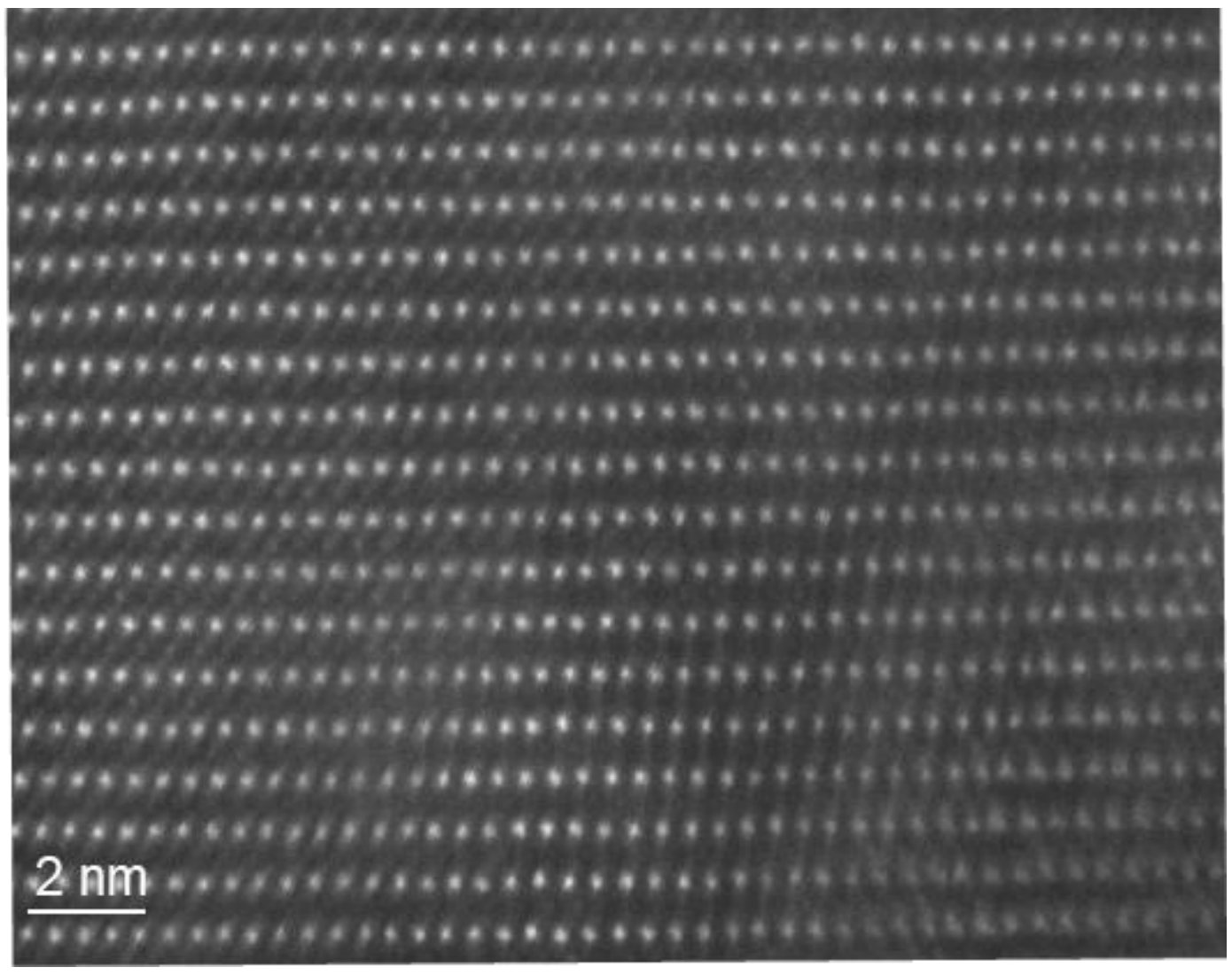

Supplementary Figure 7. HAADF-STEM image of LRMO.

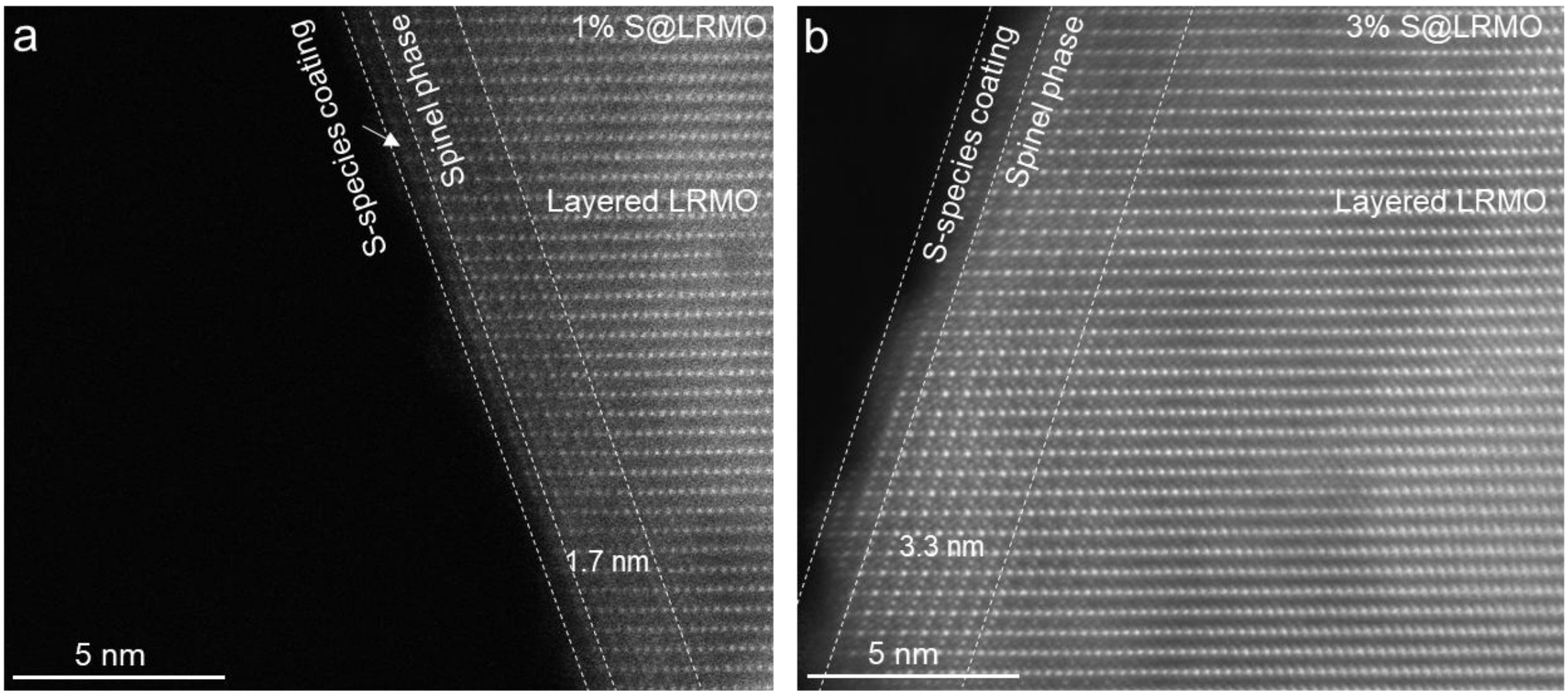

Supplementary Figure 8. HAADF-STEM image of 1% S@LRMO (a) and 3% S@LRMO (b).

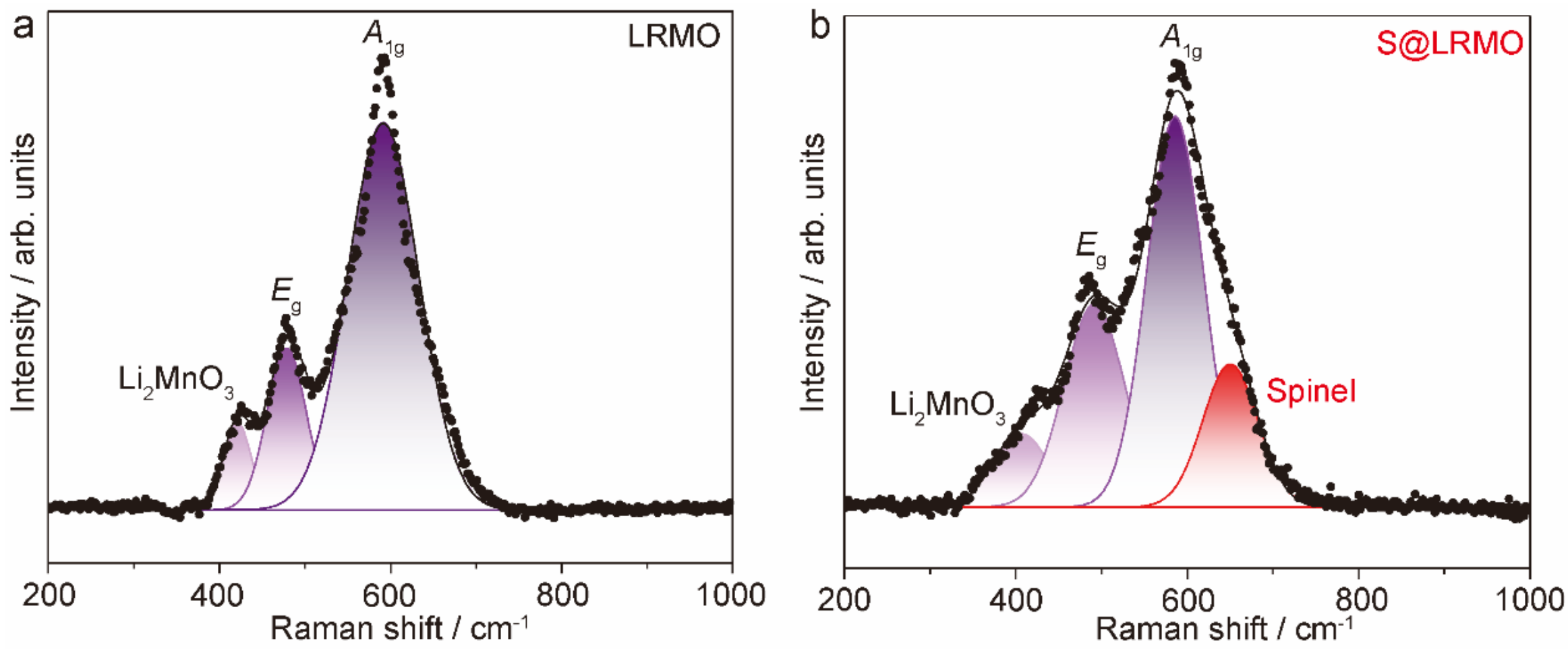

Supplementary Figure 9. Raman spectroscopy results for both LRMO (a) and S@LRMO (b).

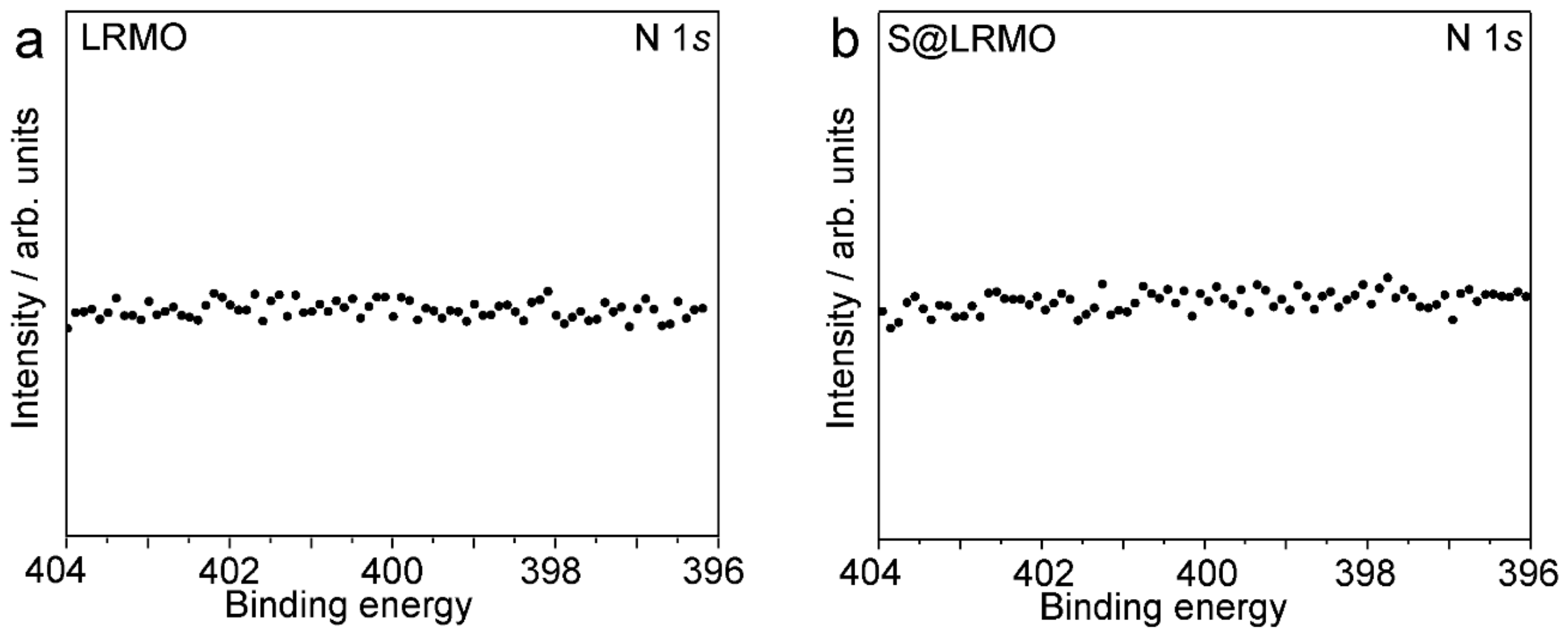

Supplementary Figure 10. XPS N 1*s* spectrum for LRMO (a) and S@LRMO (b). No obvious peaks observed in N 1*s* spectrum in S@LRMO, indicating that N element disappeared with the form of NH_3 during annealing process.

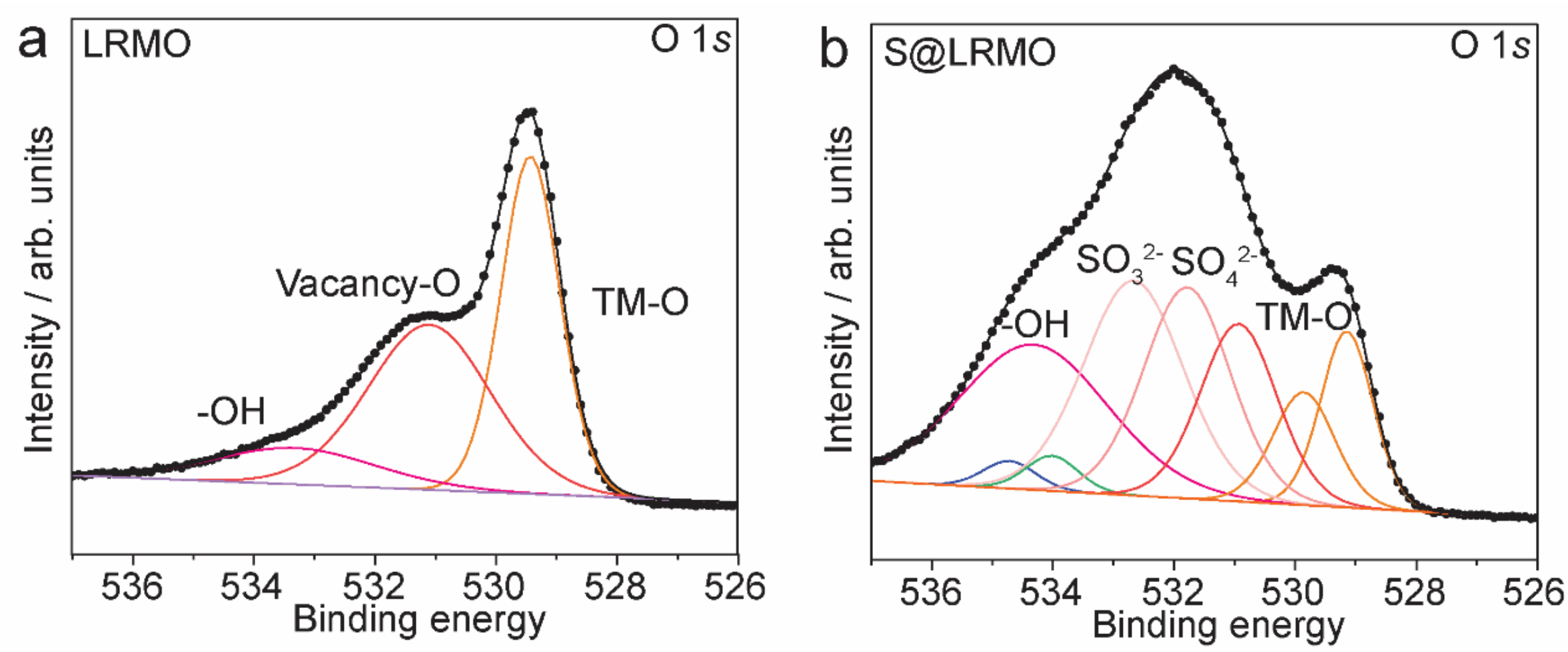

Supplementary Figure 11. XPS O 1*s* spectrum for LRMO (a) and S@LRMO (b).

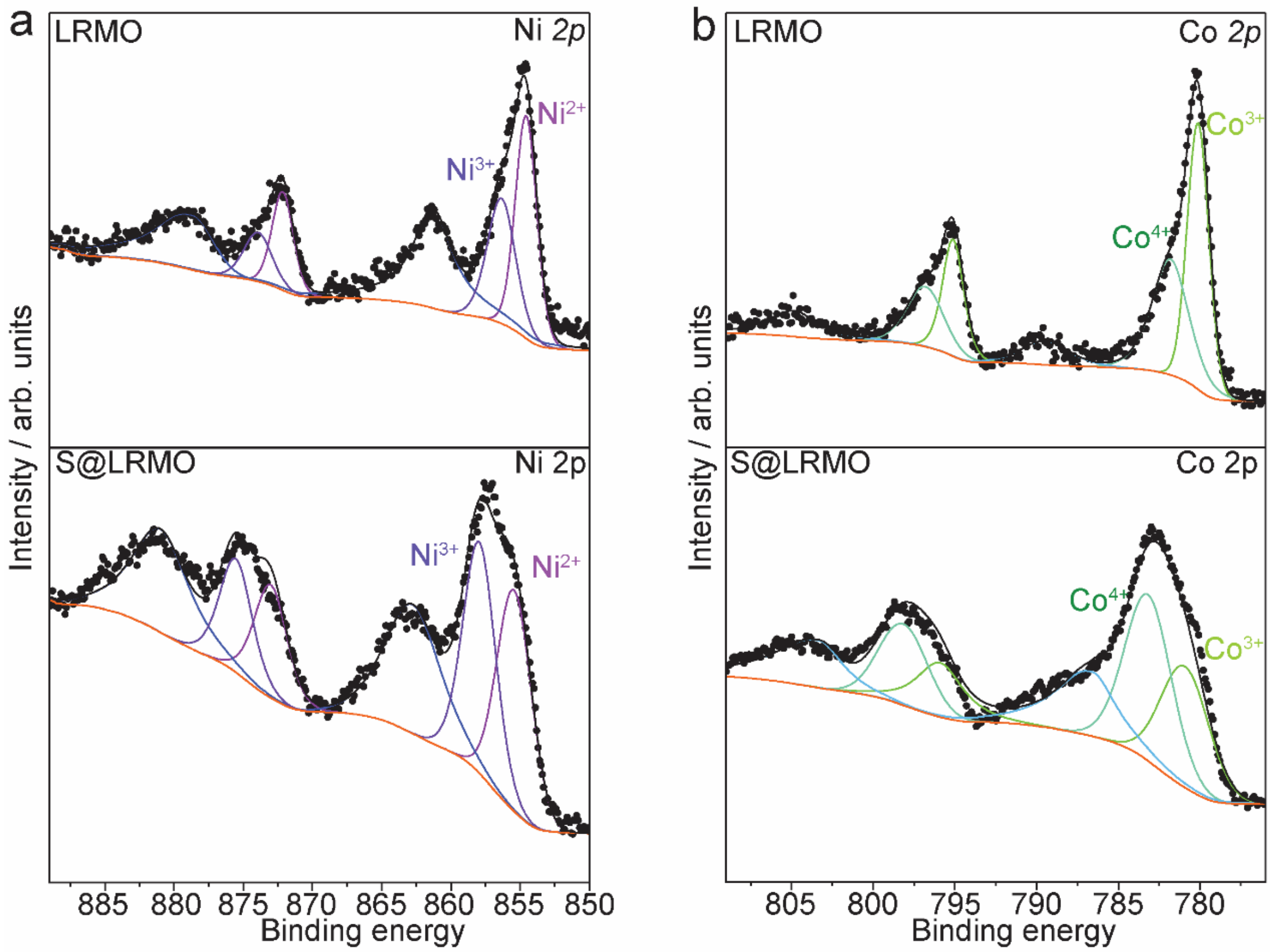

Supplementary Figure 12. The XPS spectra of the Co (a), Ni (b) for LRMO and S@LRMO.

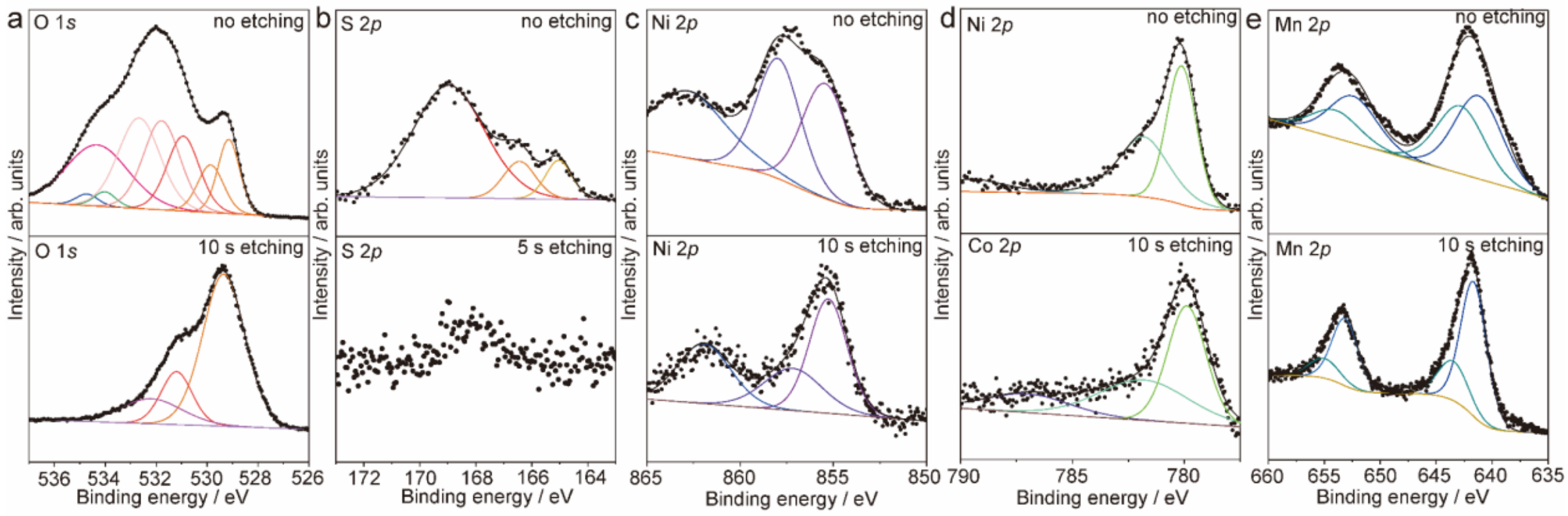

Supplementary Figure 13. O 1*s* (a), S 2*p* (b), Co 2*p* (c), Ni 2*p* (d) and Mn 2*p* (e) XPS spectra for S@LRMO with / without etching, respectively.

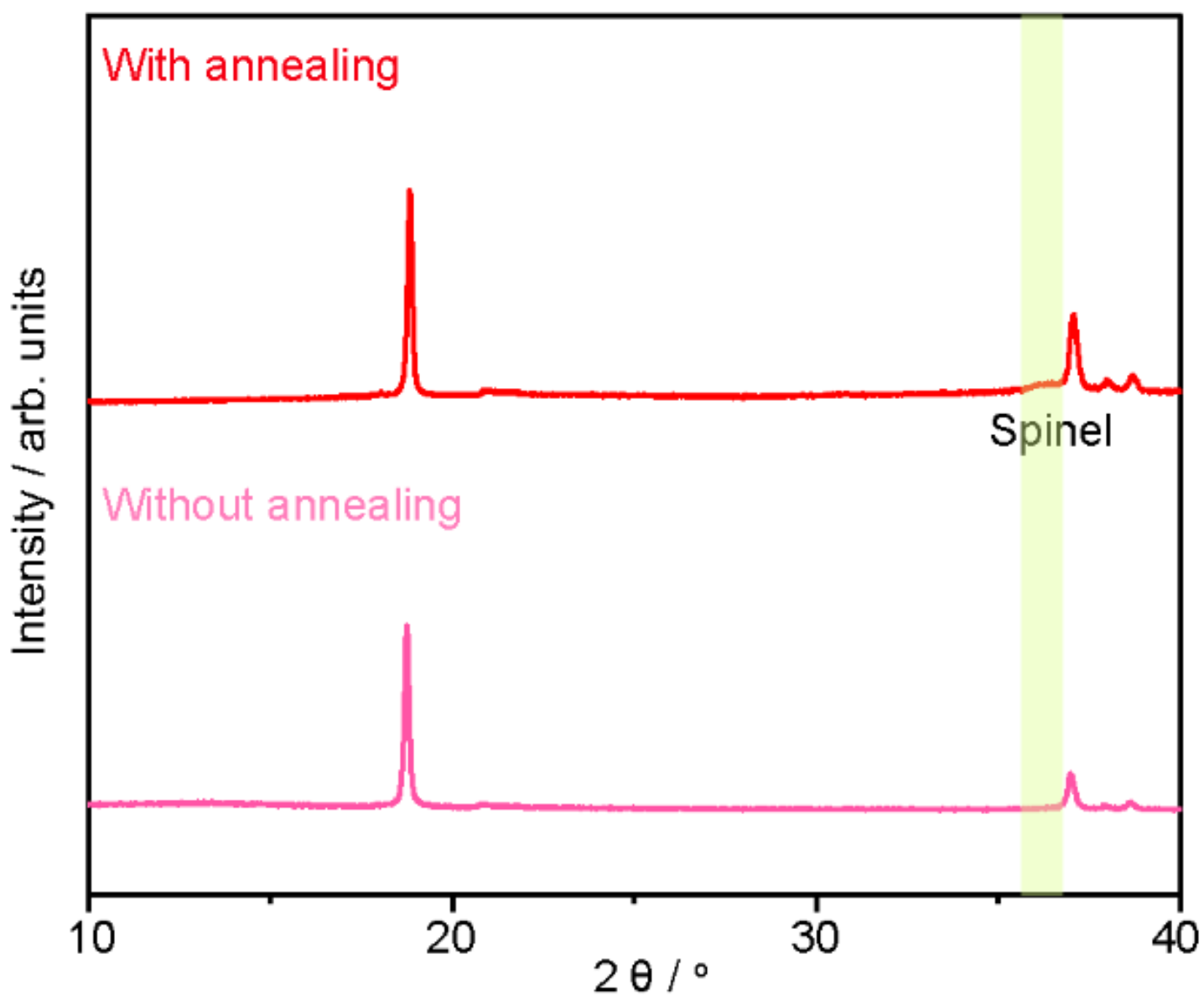

Supplementary Figure 14. XRD patterns for coated LRMO without and with annealing process.

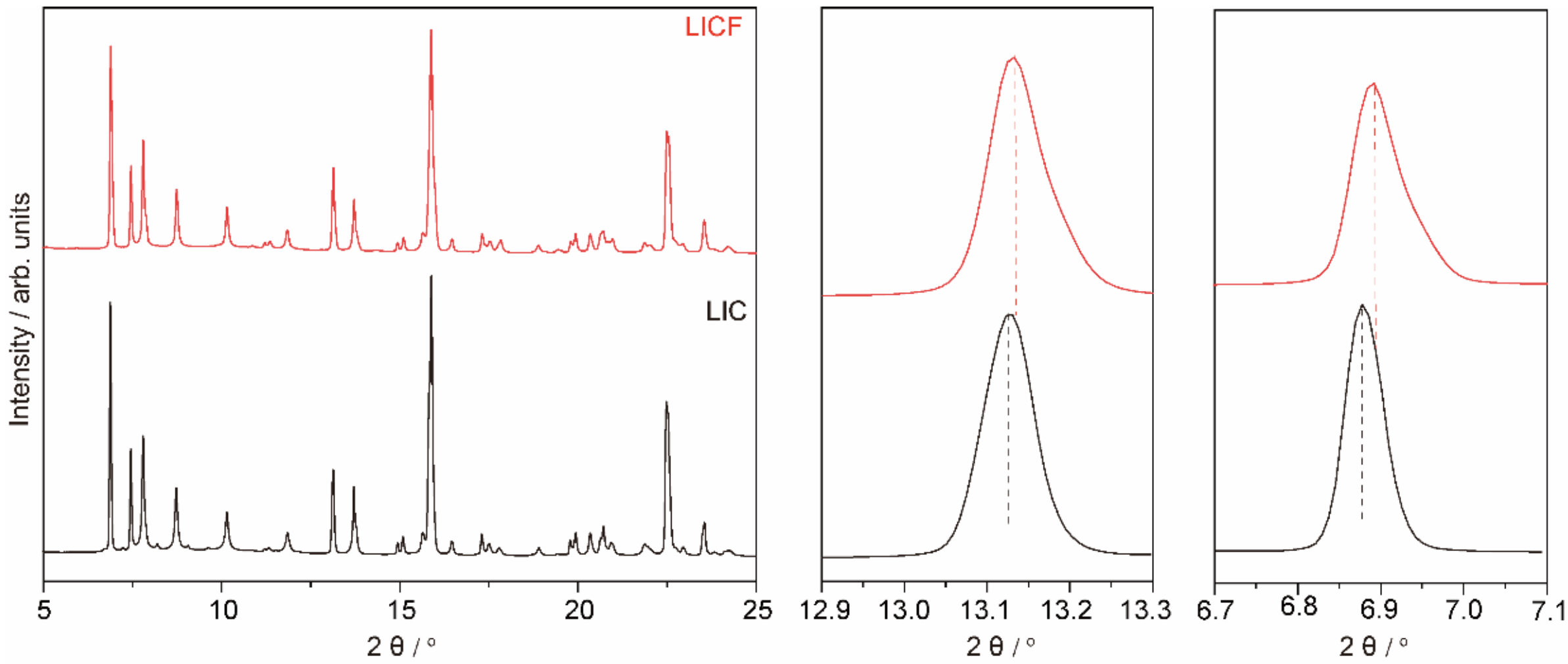

Supplementary Figure 15. sXRD patterns of Li_3InCl_6 (LIC) and $Li_3InCl_{5.4}F_{0.6}$ (LICF) collected from Bruker D8 focus. Both patterns can be indexed with monoclinic rock-salt structure with space group *C2/m* and no other phases are observed.

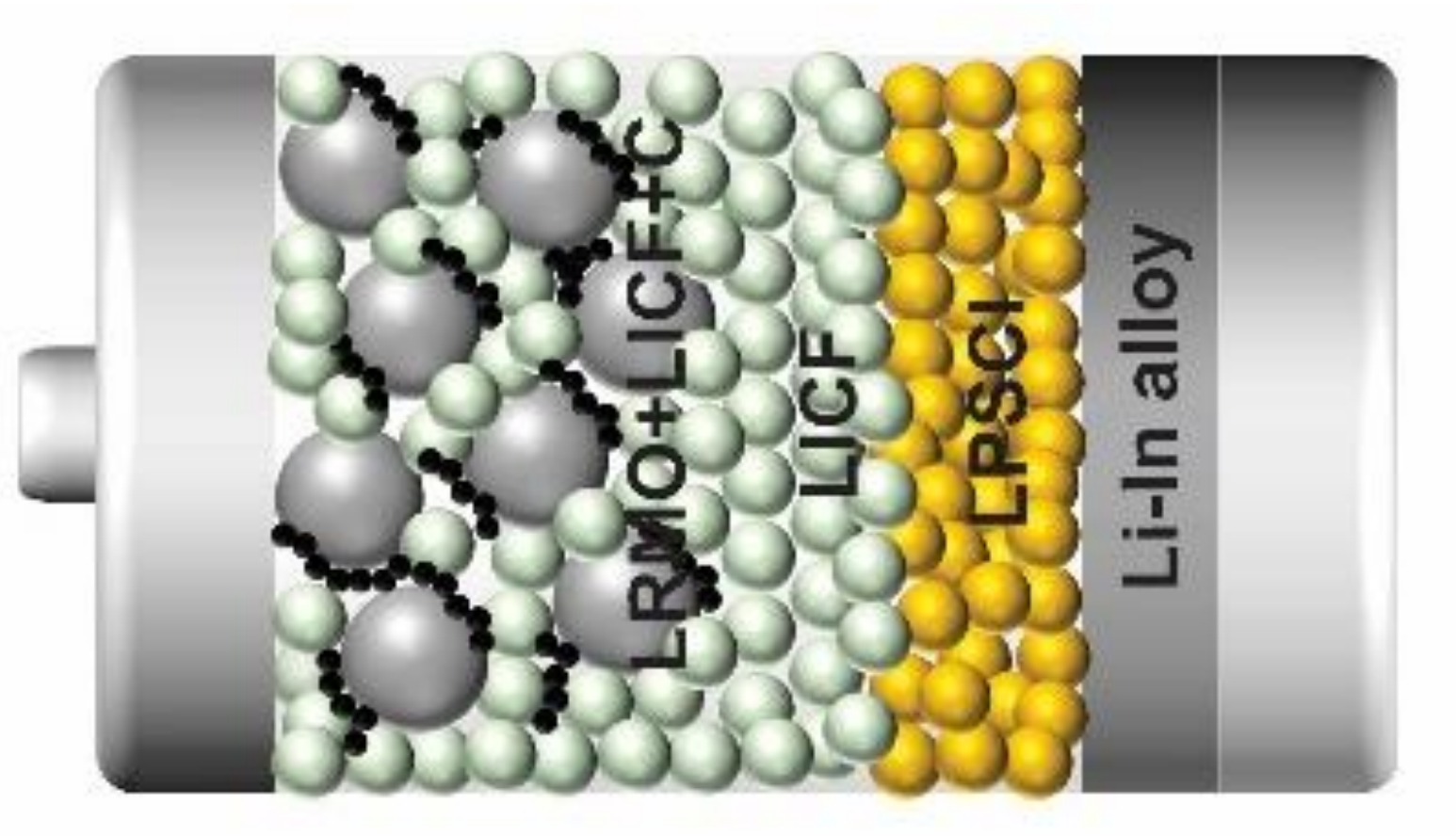

Supplementary Figure 16. The schematic illustration of the ASSLBs. The ASSLBs is assembled with LiIn as anode, LICF as the solid electrolyte and the mixed LRMO / S@LRMO positive electrode material, LICF and C as the positive electrode. To avoid the reduction reaction occurring between In^{3+} in LICF and In^{0} in the anode, an interlayer with LPSCl is used.

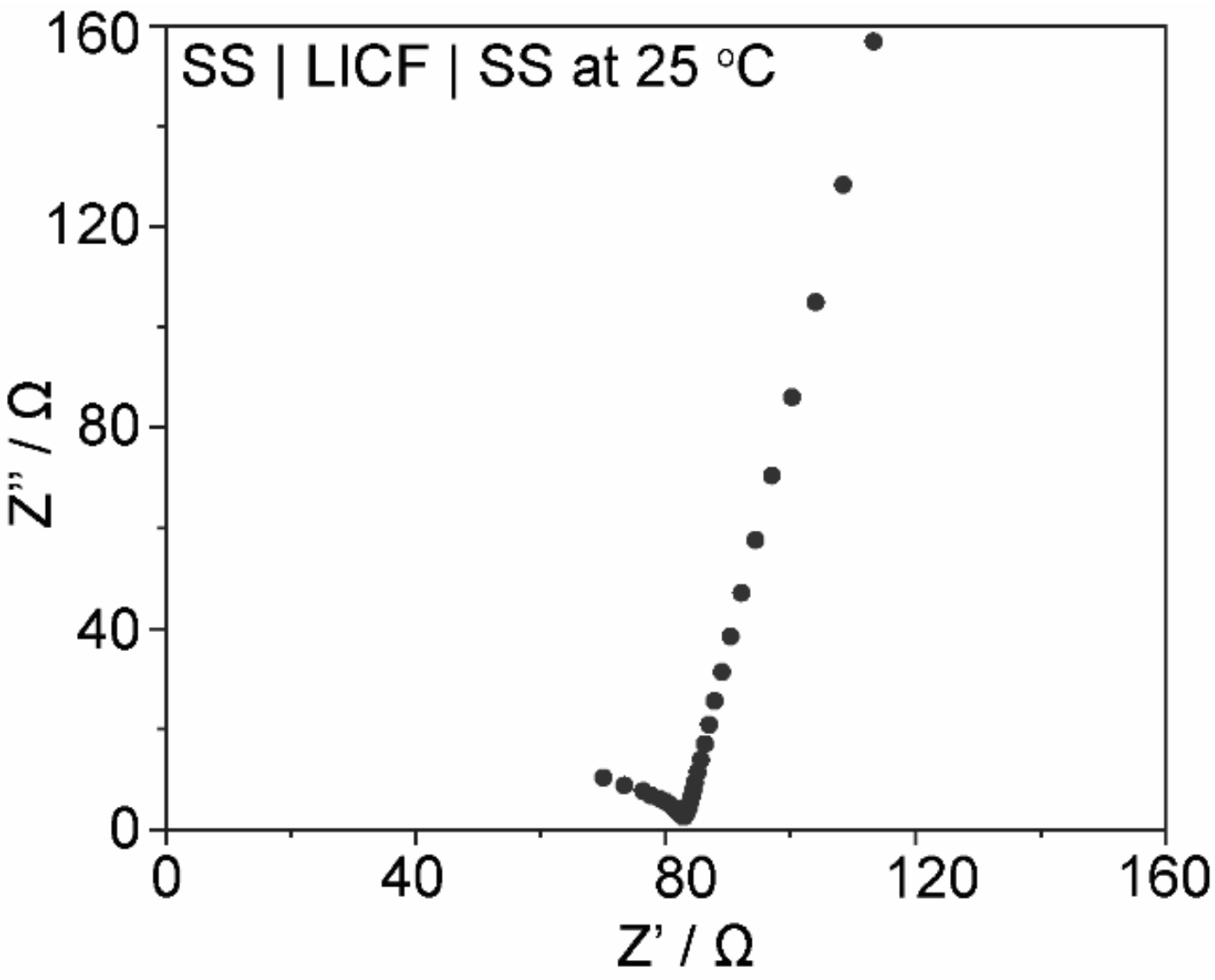

Supplementary Figure 17. Nyquist plot of LICF at room temperature. The EIS test was performed based on SS | LICF | SS (stainless steel) cell in PEEK cell with a diameter of 10 mm at room temperature and LICF exhibits high ionic conductivity of ~ 0.9 mS/cm.

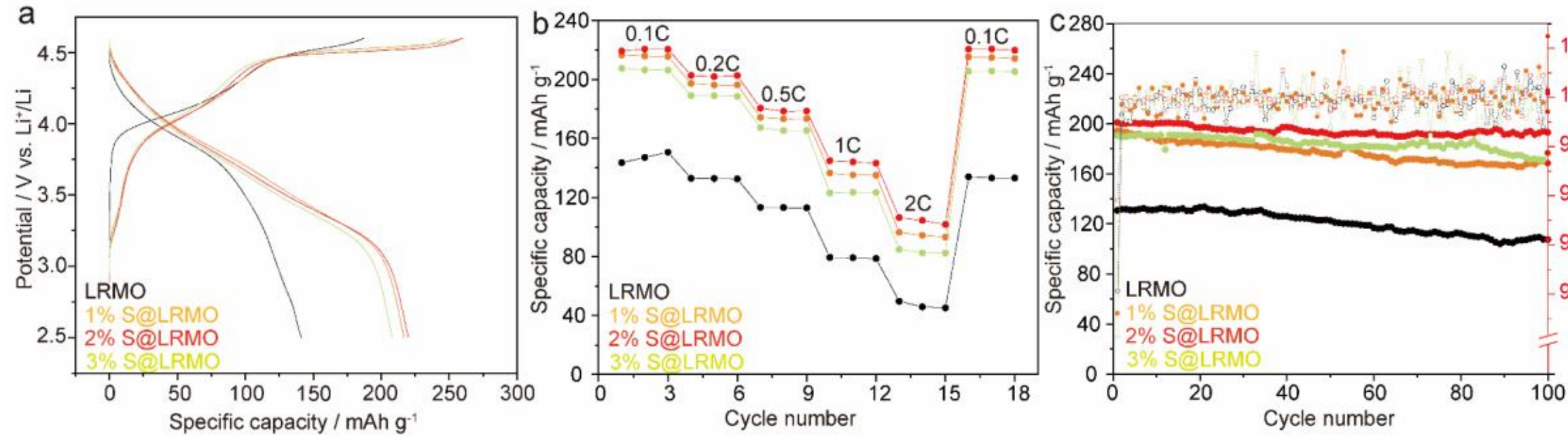

Supplementary Figure 18. Electrochemical performances of LRMO and coated LRMO with various coating contents (1 %, 2 % and 3 %). (a) charge and discharge curves of initial cycle; rate capabilities (b) and long-term cycling stabilities (c). Based on electrochemical data, we can conclude that 2 % is the optimized ratio.

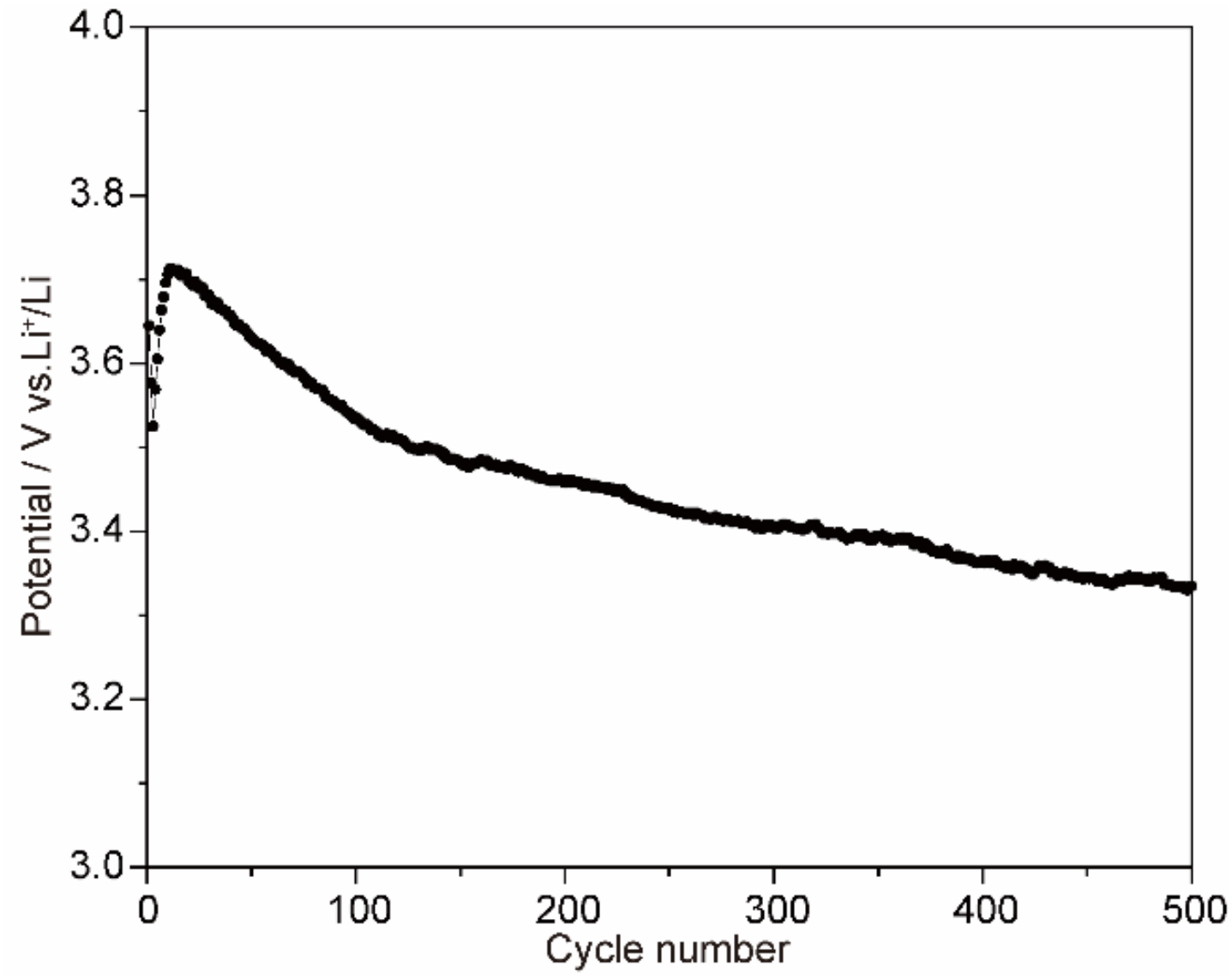

Supplementary Figure 19. The average potential change curve for S@LRMO-based cell during 1C long-term cycling.

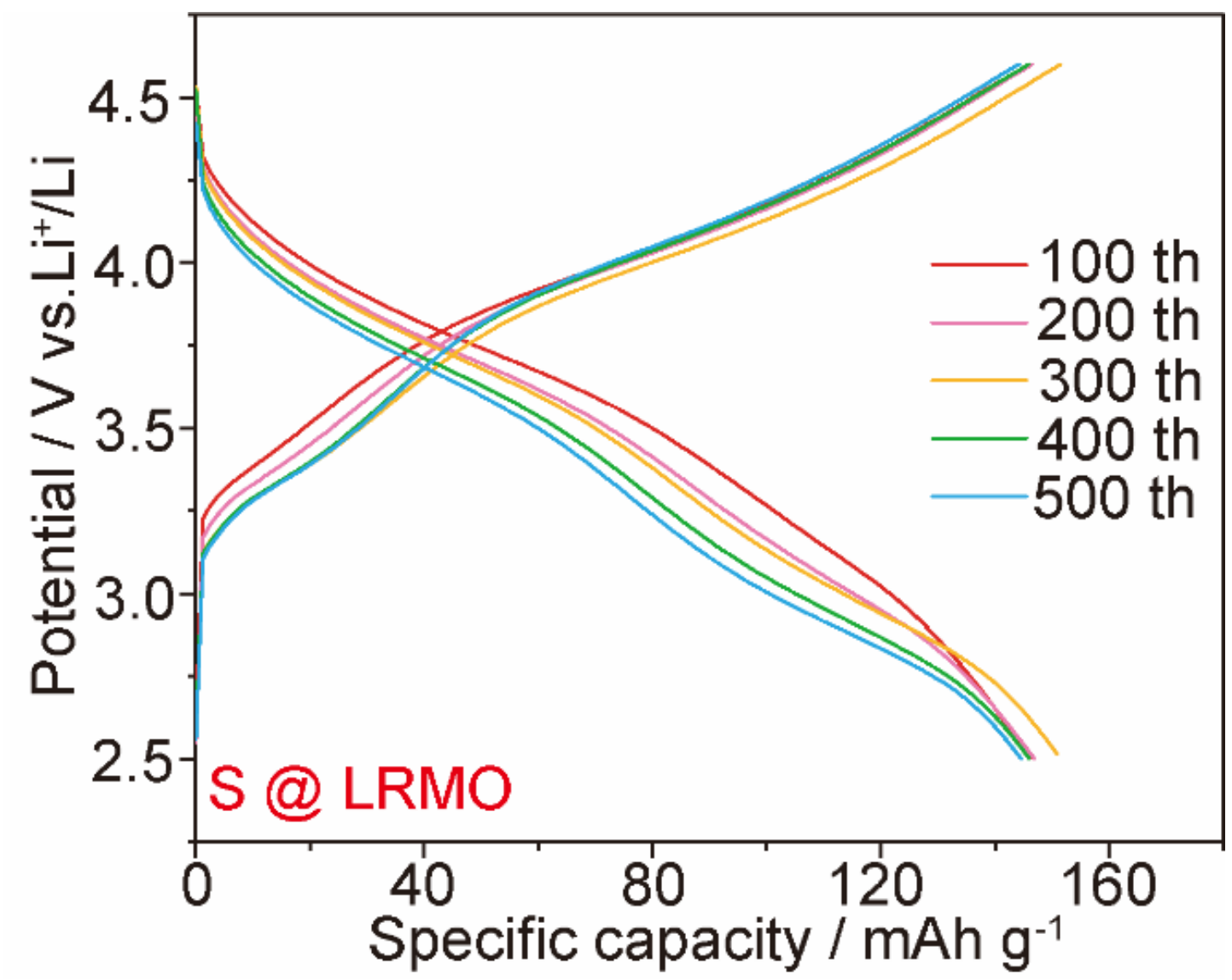

Supplementary Figure 20. Charge/discharge curves of different cycles for SSB-S@LRMO with current density of 1C.

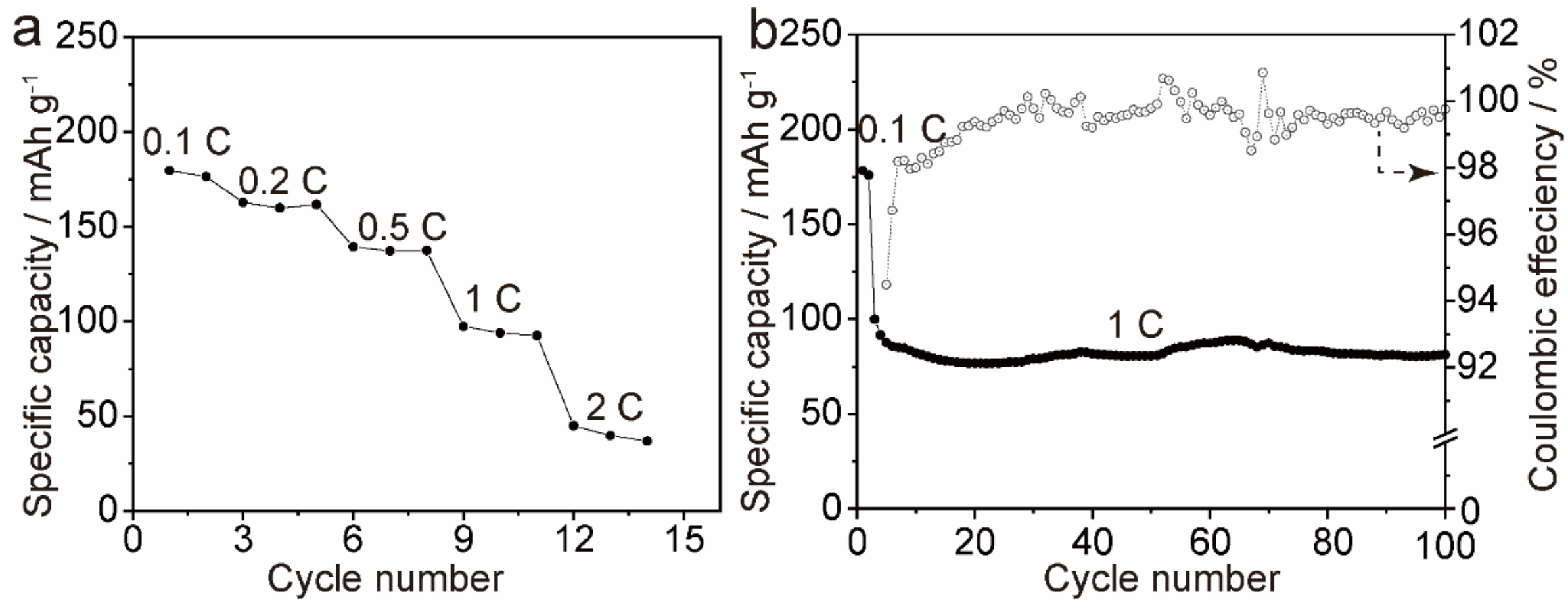

Supplementary Figure 21. Rate capabilities (a) and long-term cycling performance (b) of S@LRMO with high mass loading of 15.28 mg cm^{-2} at 25 ± 2 ºC (1 C = 250mA g^{-1}).

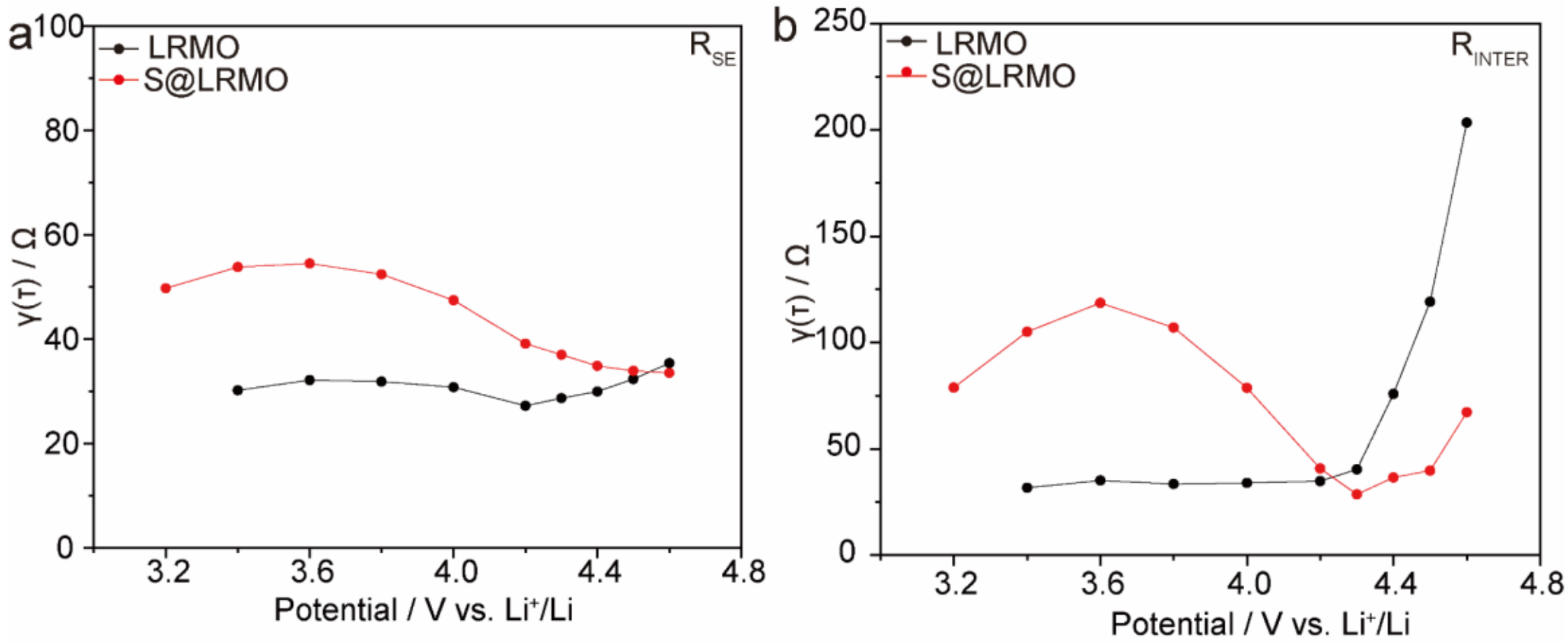

Supplementary Figure 22. (a) Solid electrolyte resistance (R_{SE}) and (b) Interfacial resistance (R_{INTER}) changes during initial charging process of SSB-LRMO and SSB-S@LRMO.

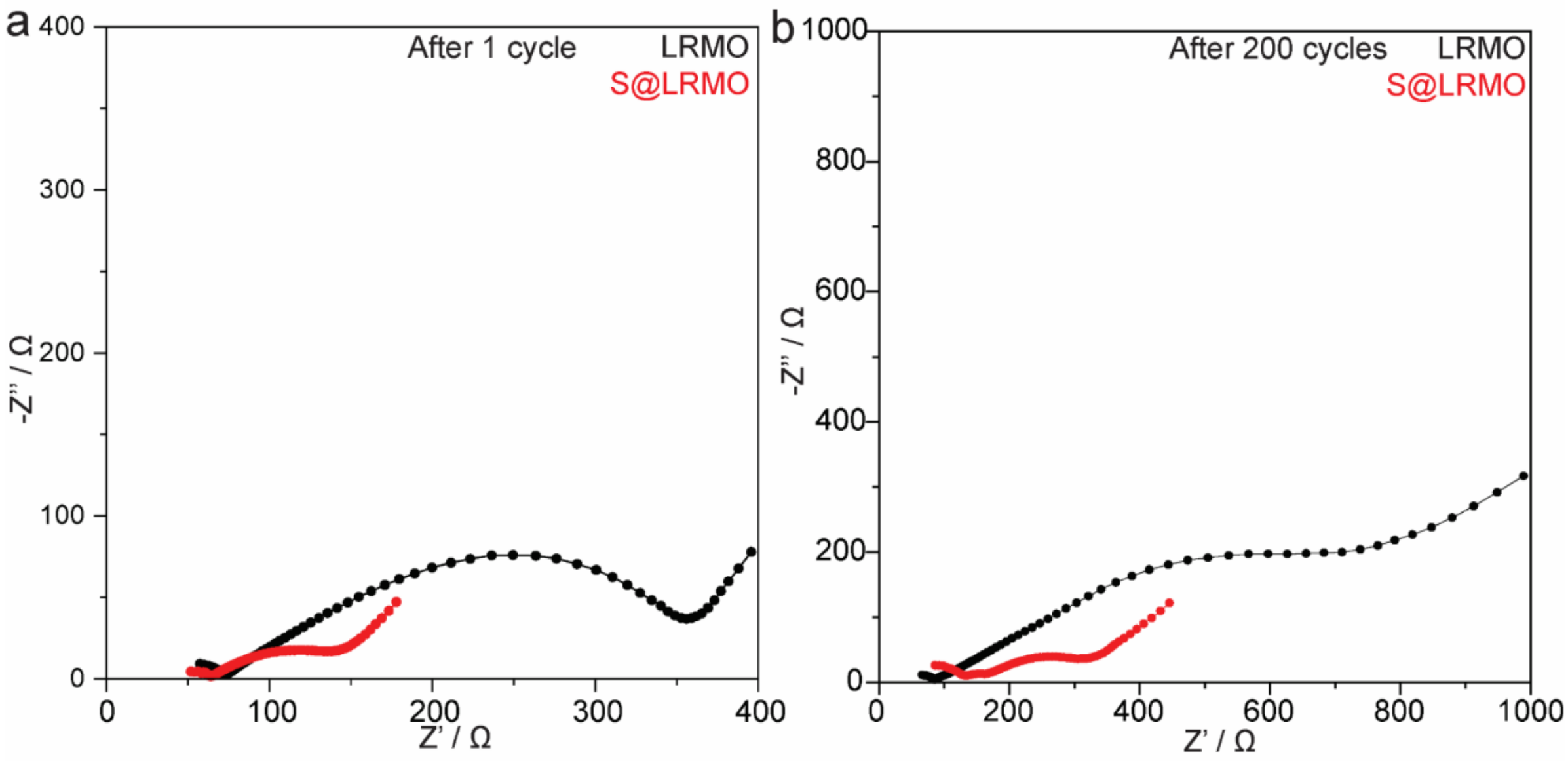

Supplementary Figure 23. The Nyquist plots of SSB-LRMO and SSB-S@LRMO after 1 (a) and 200 (b) cycles with 0.2 C.

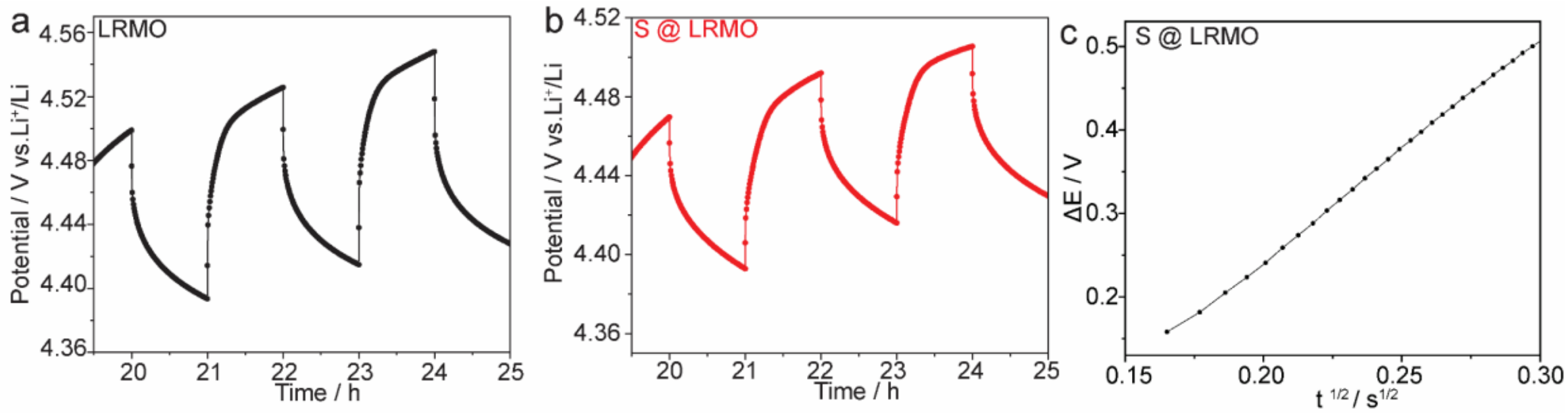

Supplementary Figure 24. Zoomed-in areas of potential-time curves for LRMO (a) and S@LRMO (b) cells during GITT tests under 0.05 C and 1 h rest. (c) $\Delta E \sim t^{1/2}$ curve during initial pulse for S@LRMO cell.

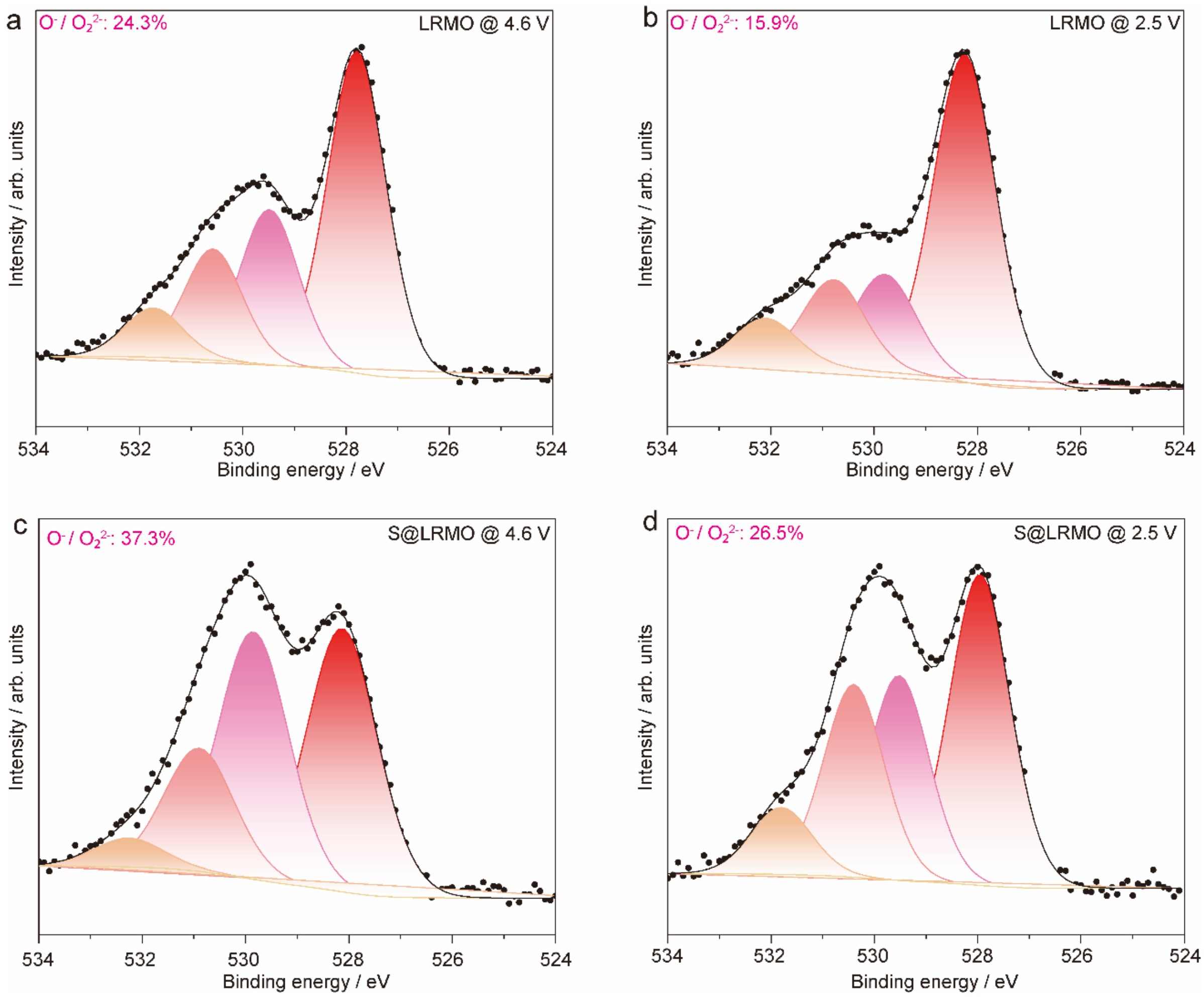

Supplementary Figure 25. O 1*s* XPS spectra of LRMO and S@LRMO composite electrodes at different electrochemical states. (a) O 1*s* XPS spectra of the LRMO composite electrode at the initial charged state (4.6 V). (b) O 1*s* XPS spectra of the LRMO composite electrode at the initial discharged state (2.5 V), after cycling at 0.1 C. (c) O 1*s* XPS spectra of the S@LRMO composite electrode at the initial charged state (4.6 V). (d) O 1*s* XPS spectra of the S@LRMO composite electrode at the initial discharged state (2.5 V). samples are collected in an Ar-filled glovebox.

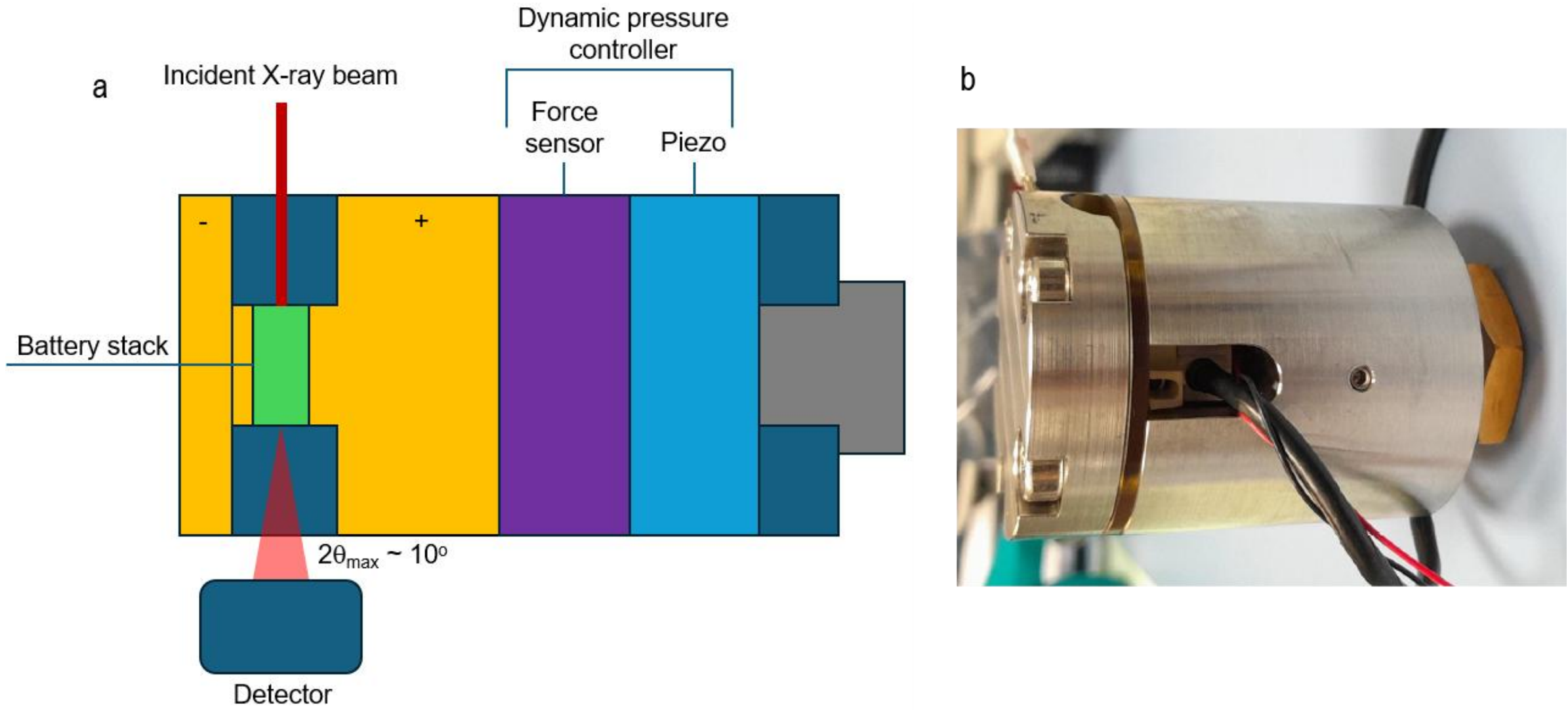

Supplementary Figure 26. Operando XRD setup. (a) Schematic of the setup, which contains dynamic pressure control using a piezo. (b) Photo of the actual device.

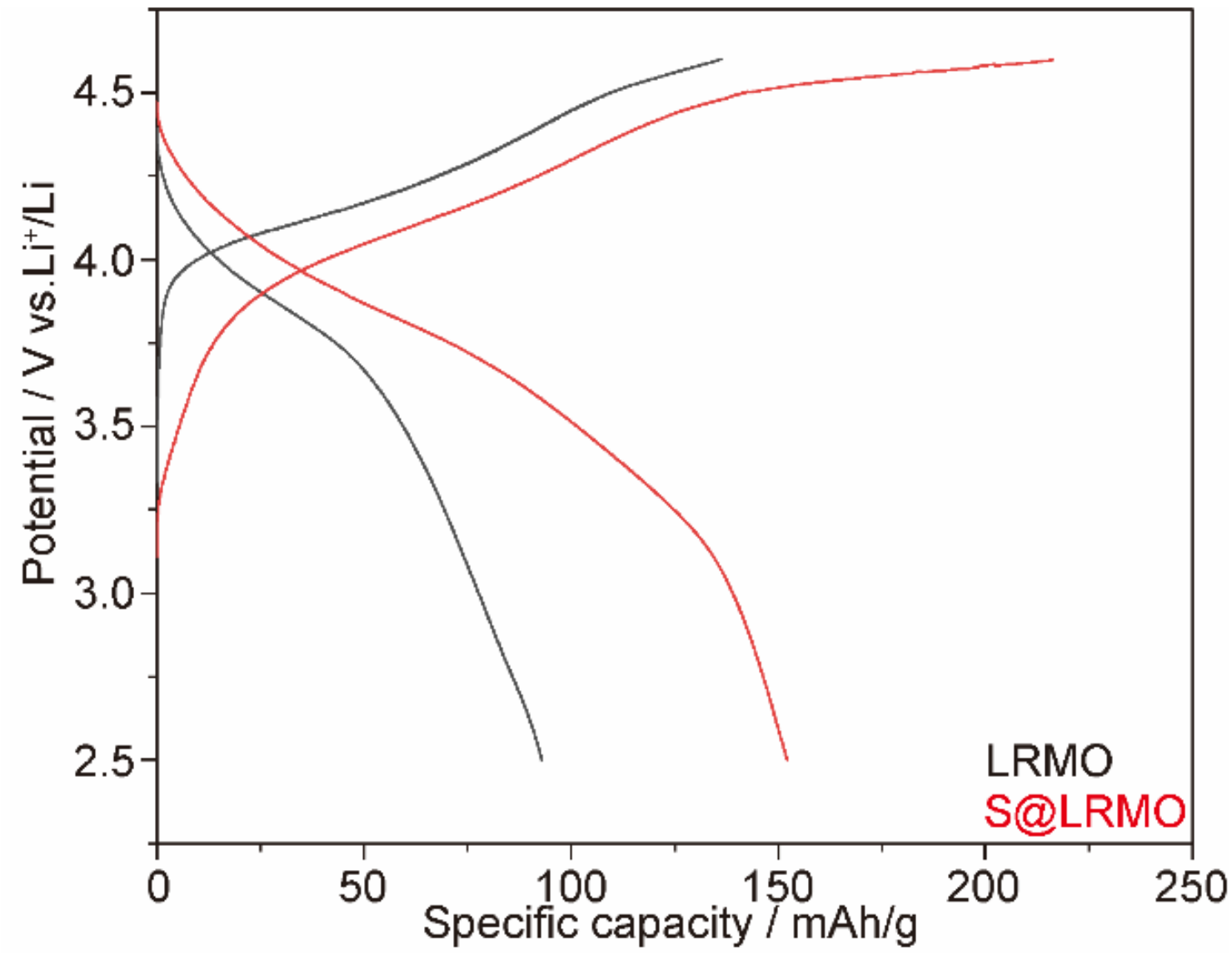

Supplementary Figure 27. Galvanostatic charge-discharge curves for LRMO and S-LRMO based operando cells in the initial cycle.

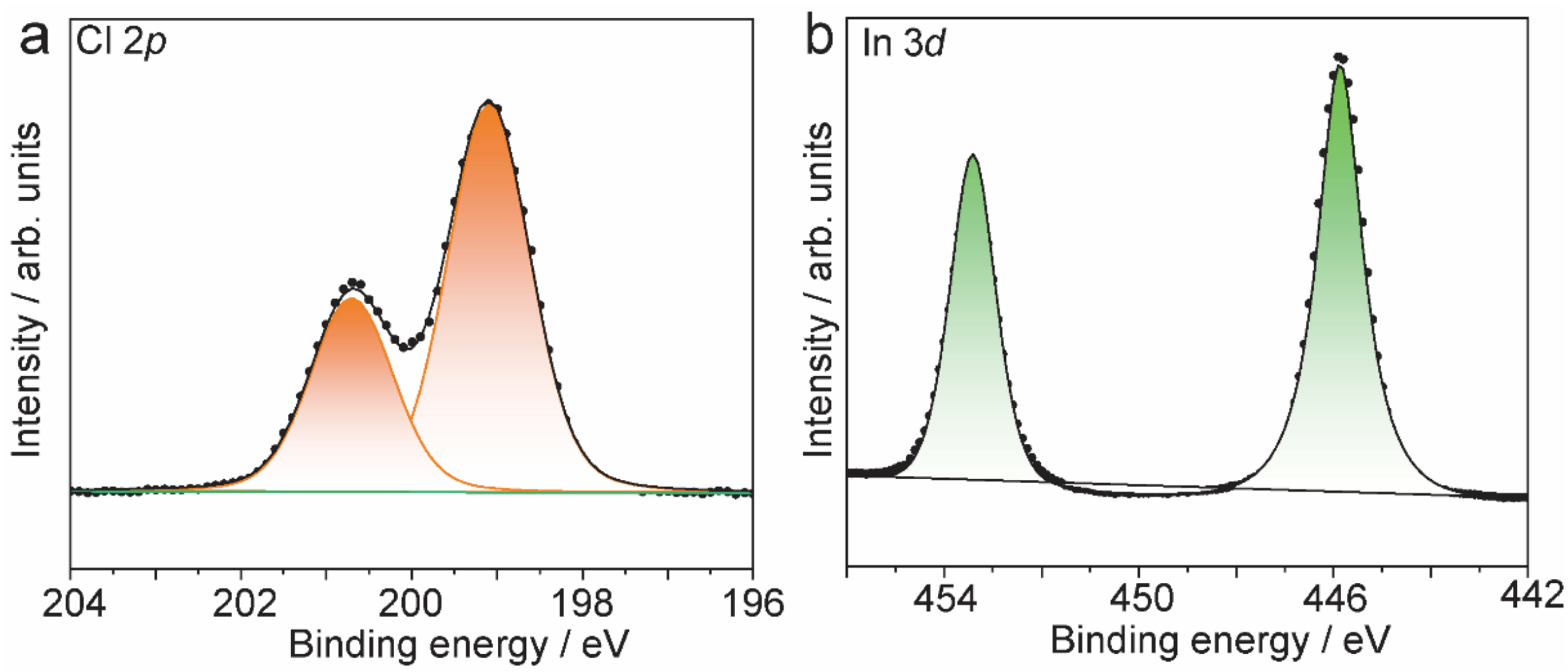

Supplementary Figure 28. Cl 2*p* (a) and In 3*d* (b) XPS spectra of LICF.

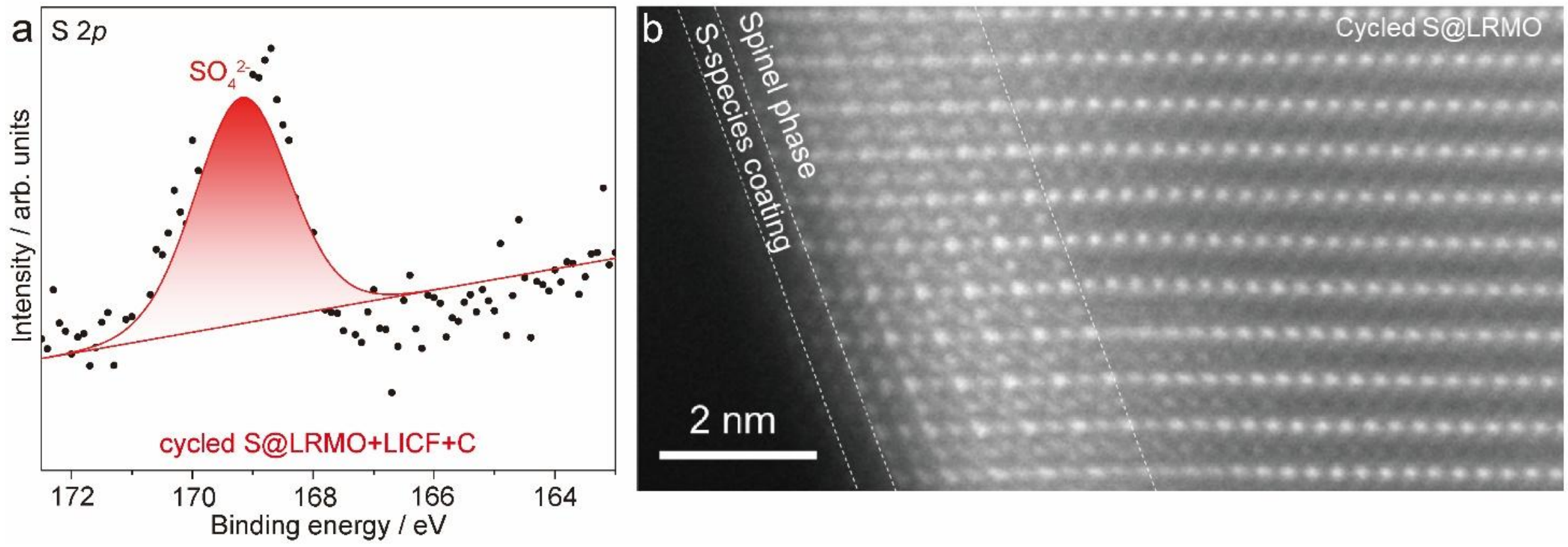

Supplementary Figure 29. S 2*p* XPS spectrum of S@LRMO positive electrode components (a) and TEM image of S@LRMO positive electrode particle (b) after 200 cycles at 0.2 C (1 C =250 mA g^{-1}) (positive electrode composites are collected at fully discharged state at Ar-filled golvebox).

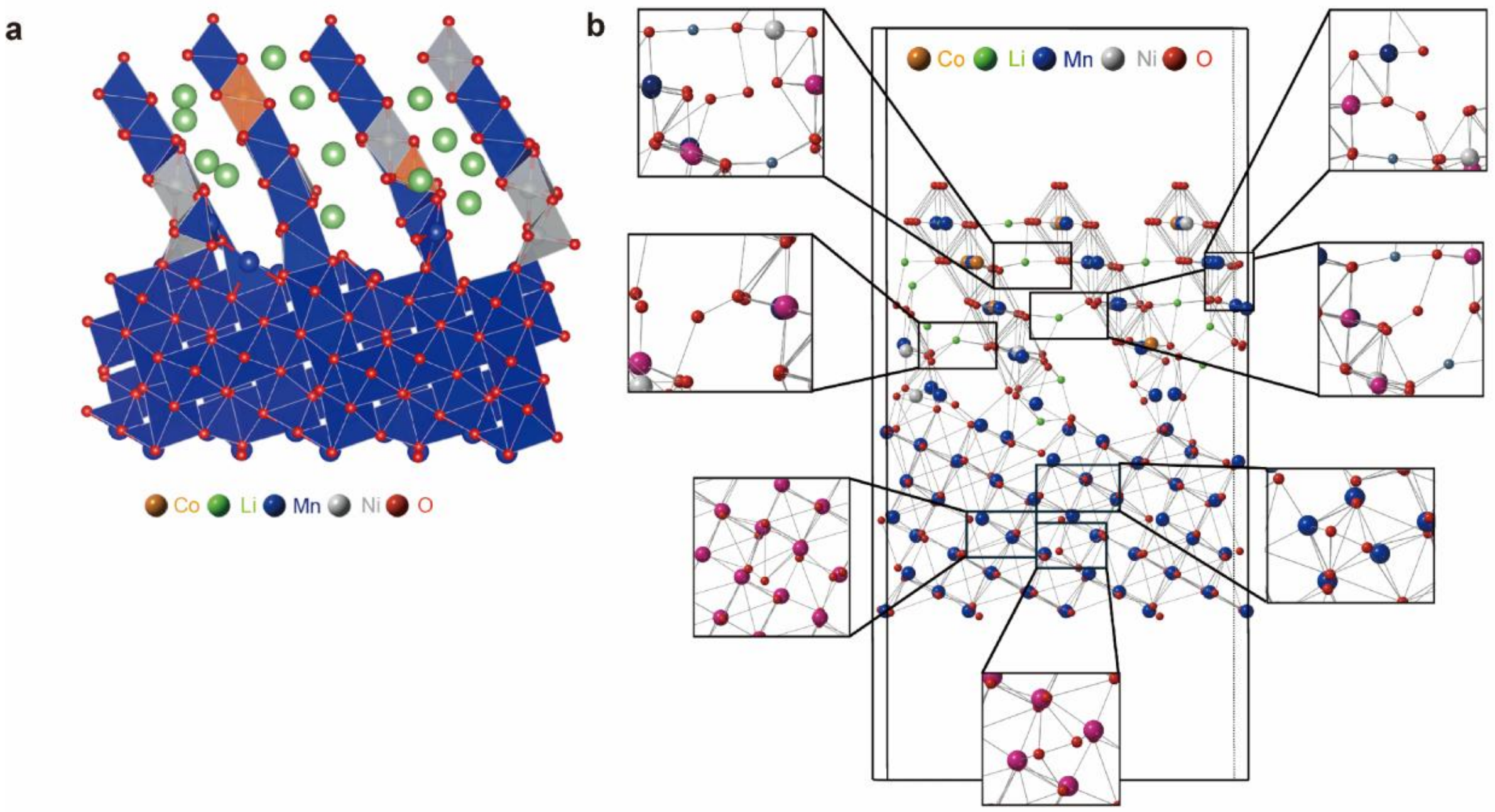

Supplementary Figure 30. Initial LRMO and spinel $LiMn_2O_4$ interface model (a) and peroxide structure (b).

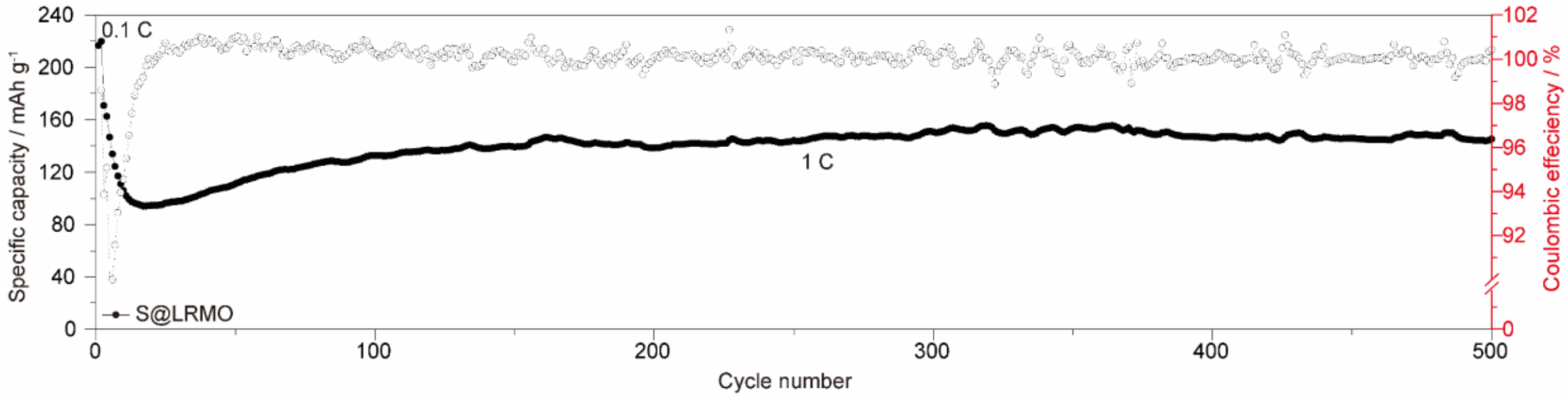

Supplementary Figure 31. The long-term cycling of S-LRMO SSB at the current density of 1.0 C (1 C = 250mA g $^{-1}$) at 25 ± 2 ºC.

Supplementary Tables

Supplementary Table S1 Crystallographic parameters of the LRMO and S@LRMO acquired from Rietveld refinement using synchrotron XRD data, λ = 0.254480 Å.

Sample	a / Å	c / Å	V / Å^3	R_{wp} / %
LRMO	2.85181(6)	14.2440(2)	100.324(2)	6.02
S@LRMO	2.85315(6)	14.2563(3)	100.504(2)	5.47

Supplementary Table S2 Rietveld analysis results of LRMO, trigonal symmetry, $R\bar{3}mH$.

Atom	Site	x	y	z	B	Occ
Li1	3*a*	0	0	0	1.24(12)	0.986(2)
Mn1	3*a*	0	0	0	1.24(12)	0.014(2)
Li2	3*b*	0	0	½	0.22(2)	0.194(8)
Mn2	3*b*	0	0	½	0.22(2)	0.546(8)
Co2	3*b*	0	0	½	0.22(2)	0.13*
Ni2	3*b*	0	0	½	0.22(2)	0.13*
O	6*c*	0	0	0.24160(10)	0.67(4)	1.00*

* Value fixed during refinement.

Supplementary Table S3 Rietveld analysis results of S@LRMO, trigonal symmetry, $R\bar{3}mH$

Atom	Site	x	y	z	B	Occ
Li1	$3a$	0	0	0	1.24(12)	0.971(2)
Mn1	$3a$	0	0	0	1.24(12)	0.029(2)
Li2	$3b$	0	0	½	0.17(2)	0.195(8)
Mn2	$3b$	0	0	½	0.17(2)	0.545(8)
Co2	$3b$	0	0	½	0.17(2)	0.13*
Ni2	$3b$	0	0	½	0.17(2)	0.13*
O	$6c$	0	0	0.24159(11)	0.56(4)	1.00*

* Values fixed during refinement.

Supplementary Table S4 Comparison advanced coatings on LRMO and corresponding electrochemical performance of all-solid state lithium batteries.

LRMO	Coating	Voltage /V	IDC@0.1 C /mAh/g	ICE	Mass loading/ mg/cm^2	C-rate/C	Long-term cycling performances	Test T/ ºC	Ref.
$Li_{1.2}Mn_{0.54}Co_{0.13}Ni_{0.13}O_2$	Li_2WO_4	2.2-4.6	218	74.83	7.64	1	81% retention after 1200 cycles	25	1
$Li_{1.2}Mn_{0.54}Co_{0.13}Ni_{0.13}O_2$	Li_2SO_4	2.3-4.6	248	94	4.2	1	81% retention after 300 cycles	25	2
$Li_{1.17}Mn_{0.55}Co_{0.05}Ni_{0.24}O_2$	Li_3PO_4	2.0-4.8	230	83.0	12.7	0.2	60 % retention after 431cycles	25	3
$Li_{1.2}Mn_{0.54}Co_{0.13}Ni_{0.13}O_2$	$LiNbO_3$	4.8	221	73.21	0.88	0.05	67 % retention after 100 cycles	25	4
$Li_{1.2}Mn_{0.54}Co_{0.13}Ni_{0.13}O_2$	$Li_2B_4O_7$	2.2-4.7	231	90	4.2	1	80% retention after 2000 cycles	25	5
**$Li_{1.2}Mn_{0.54}Co_{0.13}Ni_{0.13}O_2$**	**$SC(NH_2)_2$**	**2.5-4.6**	**220**	**84.83**	**7.13**	**1**	**97 % retention after 600 cycles**	**25**	**This work**

Supplementary Table S5 Relative energetics of peroxides formed in LRMO and spinel $LiMn_2O_4$ layers of the interface model. The peroxide bond distance was set to 1.4 Å and ΔE was determined by the difference in total energy between the given structure and the structure of peroxide 1.

Peroxide	ΔE	Structure type	Oxygen indices
4	0 eV	LRMO	227, 261
3	1.5 eV	LRMO	257, 259
1	1.5 eV	LRMO	185, 201
2	2.3 eV	LRMO	192, 200
5	2.6 eV	spinel	136, 164
6	3,6 eV	spinel	101, 130
7	6.4 eV	spinel	110, 136

Supplementary references

1. Kong, W.-J. *et al.* Bulk/Interfacial Structure Design of Li-Rich Mn-Based cathode for All-Solid-State Lithium Batteries. *J. Am. Chem. Soc.* 28190−28200 (2024).
2. Sun, S. *et al.* Eliminating interfacial O-involving degradation in Li-rich Mn-based p cathode for all-solid-state lithium batteries. *Sci. Adv.* **8**, eadd5189 (2022).
3. Yu, R. *et al.* Manipulating Charge-Transfer Kinetics of Lithium-Rich Layered Oxide cathode in Halide All-Solid-State Batteries. *Adv. Mater.* **35**, 2207234 (2023).
4. Li, X. *et al.* High-voltage all-solid-state lithium batteries with Li3InCl6 electrolyte and LiNbO3 coated lithium-rich manganese oxide cathode. *Electrochimica Acta* **453**, 142361 (2023).
5. Sun, S. *et al.* Boosting Anionic Redox Reactions of Li-Rich cathode through Lattice Oxygen and Li-Ion Kinetics Modulation in Working All-Solid-State Batteries. *Adv. Mater.* **37**, 2414195 (2025).
