# Supplementary material for: Thiourea-derived coating enabled lithium-rich manganese oxide positive electrode in solid-state batteries
Source: Nat Commun. 2026 Jul 15;17:8706. doi: 10.1038/s41467-026-75215-1 (PMC13490549; doi:10.1038/s41467-026-75215-1)
Supplement: Supplementary file 2 [file 41467_2026_75215_MOESM2_ESM.pdf]

Description of Additional Supplementary Files

Title: Supplementary Data 1

Description: DFT calculation datasets used in this work, including CONTCAR, INCAR and KPOINTS files for all reported calculations, as well as optimized structural models used for the peroxide formation energy analysis.
