# Supplementary material for: Thiourea-derived coating enabled lithium-rich manganese oxide positive electrode in solid-state batteries
Source: Nat Commun. 2026 Jul 15;17:8706. doi: 10.1038/s41467-026-75215-1 (PMC13490549; doi:10.1038/s41467-026-75215-1)
Supplement: Supplementary file 3 [file 41467_2026_75215_MOESM4_ESM.pdf]

nature portfolio

Peer Review File

Thiourea-Derived Coating Enabled Lithium-Rich Manganese Oxide Positive Electrode in Solid-State Batteries

Corresponding Author: Professor Daniel Rettenwander

This file contains all reviewer reports in order by version, followed by all author rebuttals in order by version.

Version 0:

Reviewer comments:

Reviewer #1

(Remarks to the Author)
The manuscript reports a thiourea-derived surface modification strategy for LRMO cathodes, resulting in the formation of a sub-nanometer-thick sulfur-rich coating and a spinel-like structure near the surface. This dual modification is claimed to suppress oxygen-related degradation and facilitate Li^+ transport, leading to enhanced electrochemical performance in solid-state batteries (SSBs). However, the correlation between the surface modification and the observed electrochemical improvements is not convincingly demonstrated. Furthermore, the concept of surface regulation through sulfur-based coatings is not particularly novel. Given these limitations, the manuscript is not suitable for publication in Nature Communications.

1. Incomplete Synthesis Details:
The manuscript lacks a description of the synthesis procedure for the bare LRMO powders.

2. Inconsistencies in Electrolyte Usage:
On page 4, the authors state that "both S@LRMO and pristine LRMO cathodes were tested in cells with the configuration LiIn | Li_6PSCl_5 (LPSCl) | $Li_3InCl_{5.4}F_{0.6}$ (LICF)/Cathode/C," with LICF chosen for its high oxidative stability. However, in the experimental section, it is mentioned that the cathode composite uses LPSCl, not LICF. This discrepancy must be clarified: Which solid electrolyte was used in the cathode composite—LICF or LPSCl?

3. Insufficient Coating Layer Characterization:
Although the abstract claims a "sub-nm-thick S-rich coating," the only characterization provided includes S 2p and O 1s XPS spectra indicating SO_4^{2-}, SO_3^{2-}, and C–S–C species. This evidence is insufficient to confirm the coating's structure and composition. Additional surface analyses are strongly recommended to support the claim that the coating is responsible for the enhanced electrochemical performance.

4. Unexplained Spinel Phase Formation:
In Figure 2, the manuscript claims that Mn is enriched near the particle surface, suggesting the formation of a $LiMn_2O_4$-like spinel phase. However, there is no explanation of how the thiourea-derived coating induces this phase transition. A mechanistic discussion is needed to substantiate this claim.

5. Unclear Impact on Bulk Diffusivity:
Figure 3c compares Li^+ diffusivity in LRMO and S@LRMO. However, the explanation lacks clarity and depth. Please provide a more rigorous discussion of how surface modification can influence bulk properties like diffusivity.

6. Oxygen Redox Reversibility Not Addressed:
Reversibility of the oxygen redox reaction is a critical parameter for LRMO performance but is not discussed in the manuscript. Techniques such as XAS, DEMS, or XPS could help clarify whether the thiourea coating improves oxygen redox reversibility. Additionally, the voltage decay during cycling for LRMO and S@LRMO should be discussed to evaluate oxygen redox reversibility.

7. Unsupported Claims in Conclusion:
The conclusion asserts that the coating provides a "dual effect": formation of a stable interphase and surface lattice

reconstruction, which jointly mitigate degradation and enhance Li-ion transport. However, the manuscript does not provide sufficient experimental evidence to support these claims. It is also unclear which of the two mechanisms plays a dominant role in the improved electrochemical behavior. A more evidence-based, quantitatively supported conclusion is necessary.

Reviewer #2

(Remarks to the Author)

This is an important study demonstrates a thiourea-derived surface modification strategy to enhance the performances of lithium-rich manganese oxide (LRMO) cathodes in solid-state batteries (SSBs). The work addresses critical challenges such as oxygen-related degradation and sluggish Li-ion kinetics, offering a dual modification approach that combines sulfur-rich coating and spinel-like surface reconstruction. The electrochemical results are impressive and highlight the practical potential of this strategy, particularly the exceptional capacity retention of 97% after 600 cycles at 1 C. The use of advanced characterization/analysis techniques such as operando SXRD and DRT analysis provides evidences for the proposed mechanisms. While the manuscript is well-structured and the conclusions are largely supported by the data, the following points should be addressed to strengthen the work further.

1. While the SXRD and TEM data suggest the formation of a spinel-like surface structure (LiMn2O4), the mechanism driving this reconstruction remains unclear. Is the spinel phase formed through Mn migration from the bulk during annealing, or is it a result of sulfur species interacting with the LRMO surface? A deeper discussion is necessary.

2. The GITT-derived Li+ diffusion coefficients (Figure 3c) show improved kinetics for S@LRMO. Including numerical data (e.g., DLi+ values at different voltages) and comparing them with previous literature reports would quantify the enhancement attributable to this phase. Moreover, could sulfur species react with LICF during cycling, leading to interfacial degradation?

3. The cycling results at 1 C and 25oC are good, but practical SSBs often operate under a wide range of temperatures or higher current densities. Testing the S@LRMO cathode at different temperatures (e.g., –20 to 60oC) or extreme rates (e.g., 3–5 C) would provide insights into the robustness of the coating under realistic conditions. Additionally, post-cycling structural analysis could confirm whether the spinel phase and sulfur-rich layer remain intact after prolonged operation. Also, scalability considerations, such as particle aggregation during annealing, solvent choice for large-scale processing, should be addressed.

4. The authors briefly compare S@LRMO with prior coatings (e.g., Li3PO4, Li2WO4) in Figure 2e but do not discuss the relative advantages of sulfur-based modification in depth. A table summarizing key metrics (e.g., ICE, capacity retention, coating thickness) for these systems would help contextualize the novelty and superiority of the thiourea-derived approach.

5. Some expressions are not very rigorous. For example, I do not believe that this strategy will “unlock LRMO’s full potential” (introduction part). The phrase "suppressed oxygen-related degradation" is repeated excessively.

Reviewer #3

(Remarks to the Author)
The manuscript “NCOMMS-25-19137-T” reported a modified LRMO cathode (S@LRMO), which exhibits an exceptional initial discharge capacity and outstanding capacity retention. And the origin of the improved electrochemical performance is discussed. It can be accepted in nature communication before a major revision, answering the following questions.

1. During the XRD refinement, for the 3a site, why the site occupancy was refined as Li + Mn = 1, but not Li+Ni=1? As the cation mixing is usually suggested to occur between Li+ and Ni2+.
2. Please present the in-situ XRD cell design. Is there a high pressure offered during the in-situ XRD test? Please provide the rate of the operando cell, and the related capacities of the cells.
3. Are there any errors in Figure 3c? Please check it.
4. The active process are clearly seen in Figure 2g, please explain it.
5. In Figure S2, the bragg peaks are suggested to be presented. What is the percentage of the monoclinic phase (Li2MnO3)? Why are spinel phase not observed in the TEM or HAADF-STEM images?
6. The HAADF-STEM image of LMRO is suggested to be offered.
7. In Figure S6, why does the peak belonging to -OH increase much after the treatment?
8. In Figure S11, the midpoint voltage is suggested to be replaced by the average. In Figure S11, the midpoint voltage is suggested to be replaced by the average voltage, and the charge discharge curves for the 4th, 100th, 200th, 300th, and 500th cycles are suggested to be presented.
9. In Figure S12, is the relaxation time of 1h during the GITT test not enough to get a quasi-equilibrium state?
10. Why the SEI/CEI resistance (RSEI) and charge transfer (RCT) resistance of SSB-S@LRMO decrease in the high voltage, it is unnormal. Please explain it.

Reviewer #4

(Remarks to the Author)

This work presents a thiourea-derived surface modification strategy for LRMO cathodes, aiming to create a sulfur-rich sub-nanometer coating and induce spinel-like surface reconstruction to enhance solid-state battery performance. The work failed to meet the requirements of this journal for innovation and mechanism depth. The specific reasons are as follows:

1. The proposed sulfur-rich coating modification for LRMO cathodes lacks sufficient novelty, as similar approaches utilizing sulfate/sulfite-based coatings (e.g., SO_4^{2-}/SO_3^{2-}) to stabilize the interface, suppress oxygen loss/TM migration, and enhance Li^+ transport have been previously demonstrated by Zhang et al. (Sci. Adv. 2022, DOI: 10.1126/sciadv.add5189). While the authors employ thiourea-derived coatings in this work, the fundamental chemistry and functional mechanisms appear analogous to prior article, significantly diminishing the conceptual advancement. To justify publication, the authors must explicitly clarify how their method differs in (1) coating composition or formation mechanism, (2) interfacial stabilization efficacy, or (3) electrochemical performance metrics compared to existing strategies. Without such comparative analysis, the study's incremental contribution remains unclear.

2. The manuscript presents a surface coating modification for cathode materials but fails to provide systematic experimental evidence demonstrating how the optimal coating content was determined. To establish scientific rigor and reproducibility, the authors should include: (1) electrochemical performance comparisons (charge/discharge curves, long-term cycling stability, and rate capability tests) across different coating contents (e.g., 1%, 3%, 5%), clearly illustrating the impact on capacity, voltage stability, and kinetics; (2) microstructural characterization (TEM images with thickness measurements and EDS/elemental mapping) to verify coating uniformity and layer integrity at varying coating ratios. Without these critical datasets, the justification for the selected coating content remains unsubstantiated, significantly limiting the study's practical applicability. Providing this information would strengthen the mechanistic understanding and enable readers to evaluate the optimization process objectively.

3. The authors' claim of achieving a 0.7-nm-thick uniform coating via conventional liquid-phase methods raises significant concerns regarding both methodological feasibility and experimental validation. At this thickness (equivalent to merely 2-3 atomic layers), the coating approaches single-atom precision - a feat typically only achievable through atomic layer deposition (ALD) techniques, as extensively documented in the literature. For liquid-phase approaches like sol-gel or coprecipitation, such extreme thinness would be exceptionally vulnerable to nanoparticle agglomeration and heterogeneous growth. To substantiate this extraordinary claim, the authors must: (1) provide comprehensive full-particle HRTEM images demonstrating cross-scale uniformity, supplemented by statistically rigorous thickness measurements (≥3 independent points per coating condition); (2) offer a detailed mechanistic explanation of how their liquid-phase process overcomes the fundamental limitations of nanoparticle self-assembly; and (3) include post-cycling TEM or TOF-SIMS analysis to verify whether such an ultrathin coating maintains structural integrity under electrochemical cycling, particularly at high-curvature surface features where coverage would be most challenging. Without this critical evidence, the reported coating thickness and its purported uniformity remain scientifically unsubstantiated.

4. The TEM data presented in this study are insufficient to support the structural analysis conclusions. Specifically: (1) The HRTEM images lack critical lattice spacing measurements (e.g., the (003) plane of layered structures), which are essential for direct verification of the crystal structure; (2) The SAED patterns are not indexed with zone axes or diffraction spot assignments, making accurate phase identification impossible; and (3) No comparative HRTEM analysis of cycled vs. uncycled samples is provided to demonstrate the coating's role in suppressing phase transitions. To address these deficiencies, the authors must: (i) clearly label key lattice spacings (e.g., (003), (104), and any spinel/rock-salt phase signatures) in HRTEM images; (ii) index all SAED patterns with zone axes and diffraction spots, including calibration references; and (iii) perform detailed structural evolution analysis of pre- and post-cycling samples using FFT or multi-area SAED to quantify phase transition extent. These supplemental data are crucial for validating the coating's effectiveness, as the current conclusions lack direct structural evidence at the atomic scale. Without such fundamental characterization, the mechanistic claims remain unsubstantiated.

5. The manuscript would significantly benefit from including performance evaluation under industrially relevant high cathode loadings (≥15 mg/cm²), as the current low-loading data (6 mg/cm²) may not adequately represent practical application scenarios. High-loading conditions better reveal critical challenges including interfacial contact resistance, ionic transport limitations, and mechanical stability issues - all essential factors for assessing commercial viability. Specifically, we recommend: (1) rate capability and long-term cycling tests under ≥15 mg/cm² loading to evaluate practical performance metrics; (2) mechanistic analysis of how increased loading affects electrode/electrolyte interface evolution and overall cell behavior; and (3) comparative discussion with established high-loading systems (e.g., DOI:10.1126/sciadv.add5189) to properly contextualize the technological advancement. These additions are crucial as they would more accurately reflect real-world operating conditions and better demonstrate the material's potential for scalable implementation in solid-state batteries

6. In Figure 2f, the cyclic voltammetry (CV) results show improved reversibility of cation redox reactions. For Li-rich Mn-based cathodes in all-solid-state batteries, improving the reversibility of anion redox reactions is more crucial. In Figure 2f, S@LRMO exhibits significantly stronger oxidation peaks at 4.5V and reduction peaks at 3.2V, demonstrating that the sulfur-containing coating on LRMO enhances the reversibility of anion redox reactions. This issue warrants in-depth investigation, with particular focus on elucidating how the sulfur coating stabilizes oxygen redox activity by suppressing oxygen evolution and maintaining structural integrity during cycling.

7. Related to the previous question, while this work demonstrates the effects of sulfur/sulfur-containing coating modification on performance, the underlying mechanisms remain unexplored, limiting the depth of the study. A more thorough investigation into the interaction mechanisms (e.g., chemical bonding, physical adsorption, or electron transfer between sulfur species and the host material) would significantly strengthen the work. Techniques such as Raman spectroscopy, or DFT calculations could help elucidate the sulfur-coating process and its role in enhancing material properties. Additionally, clarifying the thermodynamic/kinetic aspects of sulfur incorporation and its influence on [specific property, e.g., conductivity, stability] would provide valuable insights.

8.. The authors must supplement XPS depth profiling data, including: 1) Chemical state changes of In 3d orbitals before and after cycling; 2) Interface element distribution and valence state evolution; 3) Comparative analysis between coated and uncoated samples. These data are crucial for verifying the mechanism by which the coating layer suppresses electrolyte

oxidation.

Version 1:

Reviewer comments:

Reviewer #1

(Remarks to the Author)
The authors have addressed some of our previous concerns, and the revised manuscript shows improvement. However, several of the earlier comments have not been adequately or correctly addressed. Therefore, the manuscript remains insufficient for publication at this stage.

1. (Comment #3) The authors continue to claim that an almost single atomic layer of sulfur species is coated on the LMRO surface. As evidence, Figure 1g presents a TEM-EDS sulfur mapping image. However, in principle, TEM-EDS mapping is not capable of reliably detecting a single atomic layer of sulfur. Therefore, the presented EDS mapping does not provide convincing evidence to support this claim.

2. (Comments #4, #5, and #7 of Reviewer #1) These comments have not been adequately addressed. The authors did not provide direct experimental evidence but instead proposed plausible mechanisms for the formation of a $LiMn_2O_4$-like spinel phase and for the role of surface modification in bulk diffusivity. Without supporting data, these explanations remain speculative.

3. (Comment #6) The role of surface modification in improving the reversibility of oxygen redox reactions remains unclear. In general, the reversibility of oxygen redox reactions is primarily governed by the bulk crystal structure, such as the degree of cation disorder. This point should be more clearly discussed and supported with appropriate evidence.

Reviewer #2

(Remarks to the Author)
I have reviewed the revised manuscript and the rebuttal letter. The authors have done a good job in addressing the reviewers' comments thoroughly and convincingly. The studies on coating contents, high-mass-loading performances, and structural characterizations further strengthen the work's scientific value and practical relevance. Before final acceptance, I would suggest some minor revisions to further improve clarity and consistency.

1. The authors compare the performance of S@LRMO with other coated LRMO systems in Table S4, but the table seems not specify the testing conditions for each reference. Adding the details would enhance the comparability and contextual relevance of the data.

2. The manuscript mentions that the spinel phase facilitates Li^+ transport, while seems lack direct comparative data on Li^+ diffusivity within the spinel layer versus the bulk LRMO. Adding a brief discussion or supplementary data on the intrinsic diffusivity of the spinel phase would further corroborate this claim.

3. In the discussion of oxygen redox reversibility, the authors attribute the improved stability to the synergistic effect of the sulfur coating and spinel phase. A brief summary of which component plays a more dominant role in suppressing oxygen loss would provide more focused mechanistic insights.

4. I think the response to Reviewer #3's Question #5 remains insufficient. The claim that the LRMO composition can be represented as "$0.5Li_2MnO_3{\cdot}0.5LiMnO_2$" lacks supporting experimental evidence, such as quantitative Rietveld refinement of XRD data. Such analysis is essential to reliably determine the phase fraction of the monoclinic Li_2MnO_3 component. Furthermore, while the authors have included HAADF-STEM images showing spinel phases, transmission electron microscopy is inherently a localized technique. The observation of spinel regions in isolated areas does not demonstrate uniform transformation across the entire particle. More statistically representative analysis, such as multi-particle TEM imaging or XRD-based quantification, is needed to substantiate claims regarding the extent of spinel formation.

5. Regarding practical applications, a key concern raised by Reviewer #4, the authors' high-mass-loading (15.28 mg/cm^2) results are commendable and improve the work's relevance. However, a small-scale pouch cell demonstration would be a valuable addition. Pouch cells better capture the mechanical constraints and scalability challenges encountered in real-world solid-state battery prototypes. Even limited cycling data from a pouch cell would provide compelling evidence that this coating strategy is translatable to more application-relevant formats.

Version 2:

Reviewer comments:

Reviewer #1

(Remarks to the Author)
The authors have addressed essentially all of my previous concerns, and their revisions have substantially improved the manuscript. I have no further comments.

Reviewer #2

(Remarks to the Author)
I appreciate the authors' careful revisions and comprehensive responses to all comments. Most concerns have been well addressed, and the manuscript has been significantly improved. With one minor exception, it would be helpful if the authors could include a pouch cell test (even a small-capacity or single-layer one would suffice) to better demonstrate its practical potential. If this is not feasible at this stage, the current high-mass-loading results are acceptable as an initial effort. Overall, I recommend acceptance of this manuscript.

Open Access This Peer Review File is licensed under a Creative Commons Attribution 4.0 International License, which permits use, sharing, adaptation, distribution and reproduction in any medium or format, as long as you give appropriate credit to the original author(s) and the source, provide a link to the Creative Commons license, and indicate if changes were made.
In cases where reviewers are anonymous, credit should be given to 'Anonymous Referee' and the source.
The images or other third party material in this Peer Review File are included in the article's Creative Commons license, unless indicated otherwise in a credit line to the material. If material is not included in the article's Creative Commons license and your intended use is not permitted by statutory regulation or exceeds the permitted use, you will need to obtain permission directly from the copyright holder.
To view a copy of this license, visit https://creativecommons.org/licenses/by/4.0/

Response to Reviewer

Reviewer #1 (Remarks to the Author):

The manuscript reports a thiourea-derived surface modification strategy for LRMO cathodes, resulting in the formation of a sub-nanometer-thick sulfur-rich coating and a spinel-like structure near the surface. This dual modification is claimed to suppress oxygen-related degradation and facilitate Li^+ transport, leading to enhanced electrochemical performance in solid-state batteries (SSBs). However, the correlation between the surface modification and the observed electrochemical improvements is not convincingly demonstrated. Furthermore, the concept of surface regulation through sulfur-based coatings is not particularly novel. Given these limitations, the manuscript is not suitable for publication in Nature Communications.

1. Incomplete Synthesis Details: The manuscript lacks a description of the synthesis procedure for the bare LRMO powders.

Response:

We thank the reviewer for pointing out this omission. The bare LRMO material used in this study was a commercially available product purchased from Ningbo Lanli Energy Technology Co., Ltd. The material consists of polycrystalline secondary particles with an average particle size of approximately 10-15 μm.

To clarify this point, we have revised the manuscript and Supplementary Information to explicitly state the origin and basic characteristics of the pristine LRMO material. A representative SEM image of a single LRMO secondary particle has been added to support this description (Figure R1 and Figure S3).

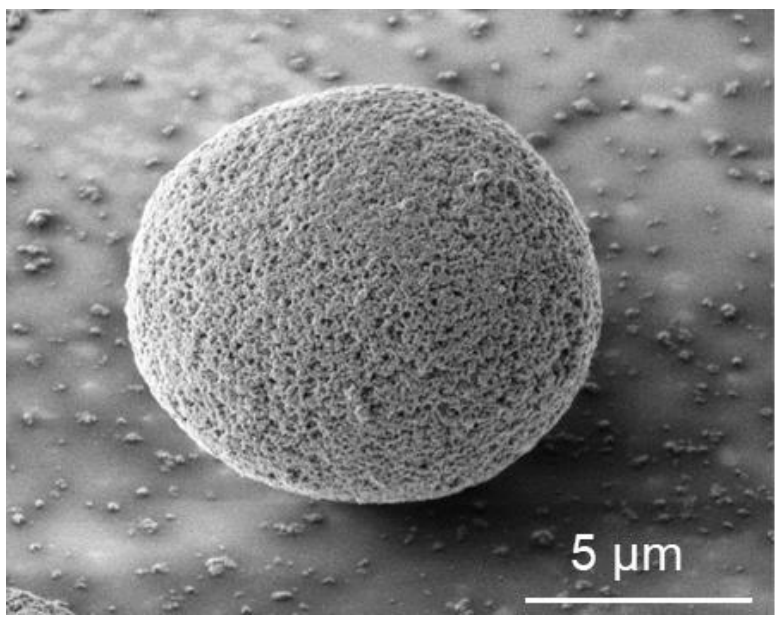

Figure R1. SEM image of a single LRMO secondary particle.

Specifically, the following text has been added to the **Materials synthesis** section of the main manuscript:

"*Commercial LRMO powder (2 g, Ningbo Lanli Energy Technology Co., Ltd.) was used as the starting material and dispersed in thiourea-containing deionized water (0.04 g thiourea in 30 mL).*"

In addition, the Supplementary Information has been updated to include:

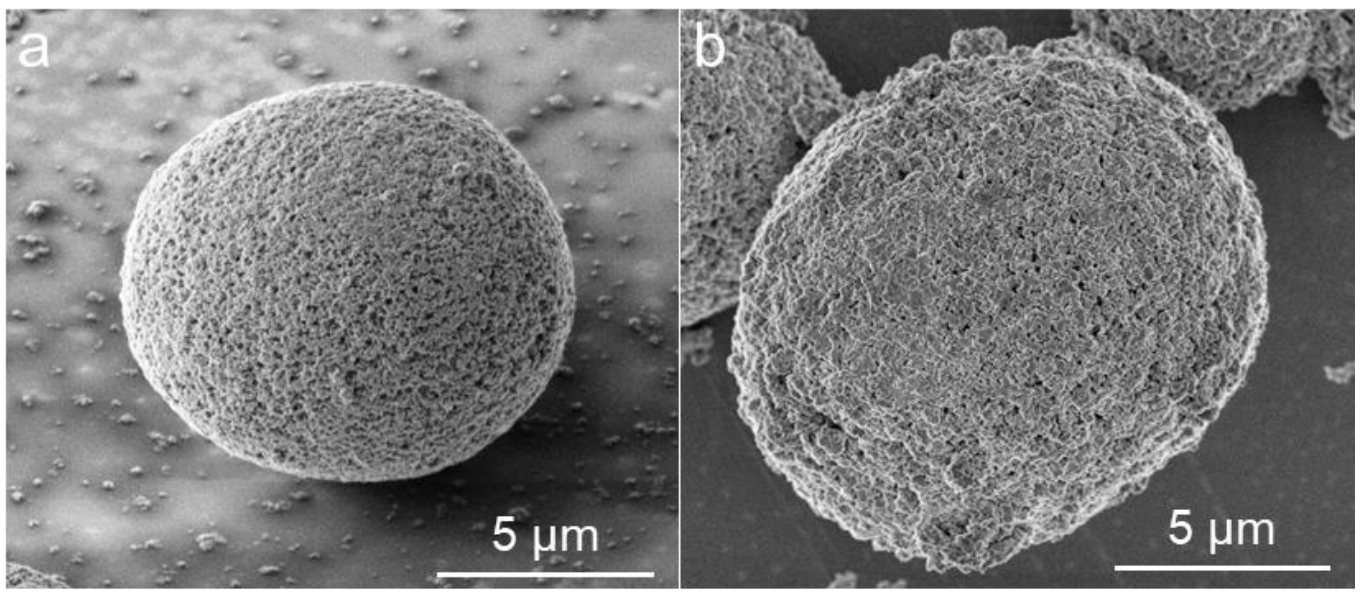

Figure S3. *SEM images of pristine LRMO (a) and sulfur-modified LRMO (S@LRMO) (b).*

2. On page 4, the authors state that "both S@LRMO and pristine LRMO cathodes were tested in cells with the configuration LiIn | Li_6PSCl_5 (LPSCl) | $Li_3InCl_{5.4}F_{0.6}$ (LICF)/Cathode/C," with LICF chosen for its high oxidative stability. However, in the experimental section, it is mentioned that the cathode composite uses LPSCl, not LICF. This discrepancy must be clarified: Which solid electrolyte was used in the cathode composite—LICF or LPSCl?

Response:

We thank the reviewer for carefully identifying this inconsistency. The discrepancy arose from an error in the Experimental section, and we apologize for the confusion. The cathode composite consists of LRMO (or S@LRMO), LICF, and carbon black, not LPSCl. LICF was intentionally selected as the cathode-side solid electrolyte due to its high oxidative stability against the high-voltage LRMO cathode. In the assembled solid-state cell, LPSCl is employed exclusively as an interlayer between the LiIn anode and the LICF electrolyte to prevent unfavorable interfacial reactions between In^0 and In^{3+} species. Thus, the full cell configuration is: LiIn | LPSCl | LICF | (LRMO or S@LRMO + LICF + C). To improve clarity, we have added a schematic illustration of the solid-state cell configuration in the Supplementary Information (Figure R2, now included as Figure S16).

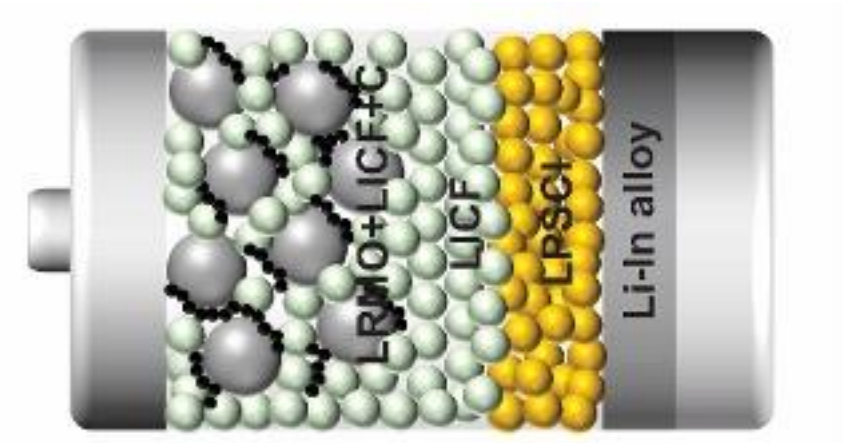

Figure R2 *Schematic illustration of solid-state cells.*

In addition, we have corrected the Experimental section in the main manuscript as follows:

"Then 10 mg of cathode composite powder (mixture of active material (LRMO or S@LRMO), carbon black (CB) and LICF with a mass ratio of 60:5:40) were placed homogeneously on one side of LICF pellet and pressed at 3 tons."

3. Insufficient Coating Layer Characterization: Although the abstract claims a "sub-nm-thick S-rich coating," the only characterization provided includes S 2p and O 1s XPS spectra indicating SO_4^{2-}, SO_3^{2-}, and C–S–C species. This evidence is insufficient to confirm the coating's structure and composition. Additional surface analyses are strongly recommended to support the claim that the coating is responsible for enhanced electrochemical performance.

Response:

We thank the reviewer for this important comment. We agree that XPS data alone are insufficient to fully establish the structure and composition of the surface modification. In the revised manuscript, we therefore provide comprehensive multi-scale surface characterization to substantiate the presence, thickness, and nature of the sulfur-rich coating, as well as the concomitant formation of a spinel-like surface layer.

Based on HAADF-STEM imaging, TEM line-scan analysis combined with EELS, synchrotron X-ray diffraction (sXRD), high-resolution TEM (HRTEM), and Raman spectroscopy, we demonstrate that the surface modification consists of a dual-layer structure:

i) An outer, sub-nanometer sulfur-rich coating, and

ii) An inner Mn-rich spinel-type $LiMn_2O_4$ layer formed via partial surface reconstruction of LRMO.

Specifically, HAADF-STEM images reveal a continuous, ultrathin surface layer on S@LRMO particles (Figure R3a).

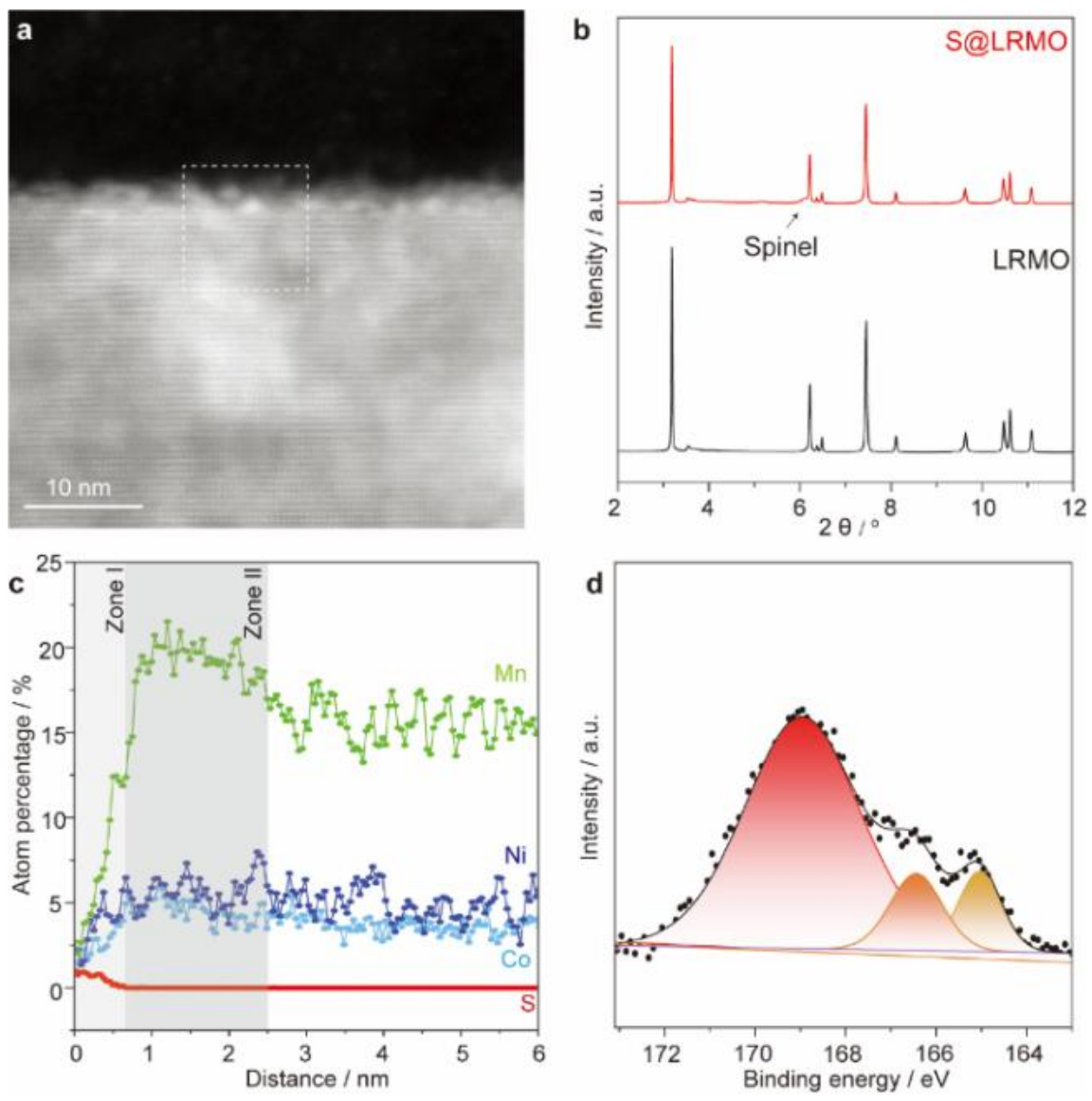

Figure R3 (a) High-Angle Annular Dark-field Scanning Transmission Electron Microscopy (HAADF-STEM) image from a S@LRMO cathode particle; (b) Synchrotron X-Ray Diffraction (SXRD) patterns collected from the bare LRMO and S@LRMO powders; (c) Element line profiles based on the HAADF-STEM image; (d) S 2p XPS spectrum for bare LRMO and S@LRMO particles.

Elemental line-scan profiles and EELS mapping confirm the surface enrichment of sulfur, while Co, Ni, and Mn are retained in the same region, indicating that the sulfur species are transition-metal-based sulfites rather than physically adsorbed sulfur compounds (Figure R3c). The chemical states of sulfur (SO_4^{2-}, SO_3^{2-}, and C-S-C species) are further confirmed by S 2p XPS spectra (Figure R3d). In parallel, sXRD patterns reveal weak additional reflections that can be indexed to a spinel phase in S@LRMO, which are absent in pristine LRMO (Figure R3b). High-resolution TEM directly resolves lattice fringes corresponding to spinel $LiMn_2O_4$ at the particle surface (Figure R4), providing direct structural evidence for surface reconstruction.

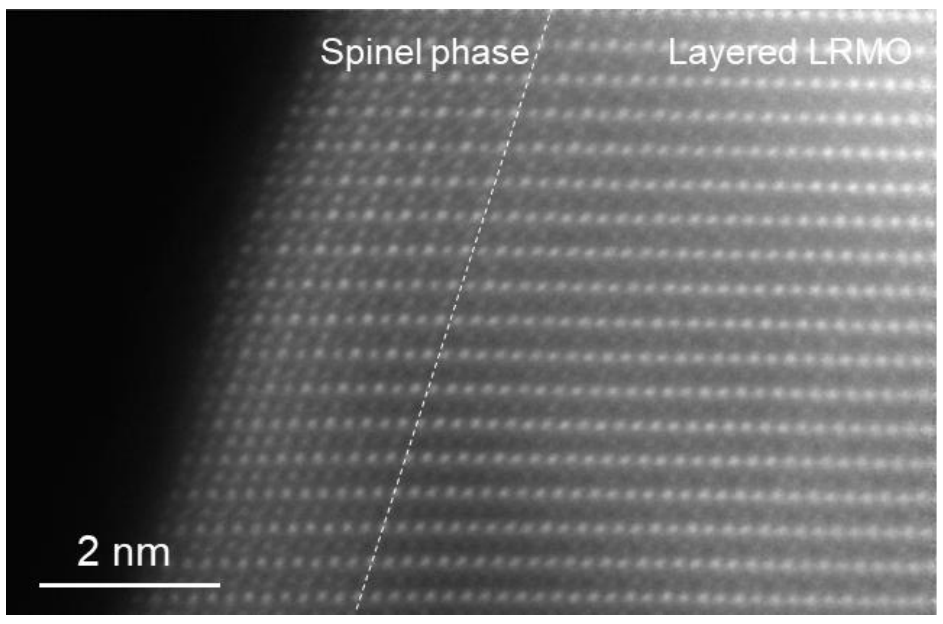

Figure R4 High-resolution TEM images of S@LRMO.

Furthermore, Raman spectroscopy independently corroborates the formation of the spinel phase: while both LRMO and S@LRMO exhibit characteristic Li_2MnO_3-related modes, the S@LRMO sample shows a distinct shoulder peak near **652 cm⁻¹**, attributable to Mn–O stretching vibrations in the spinel structure (Figure R5 / Figure S9).

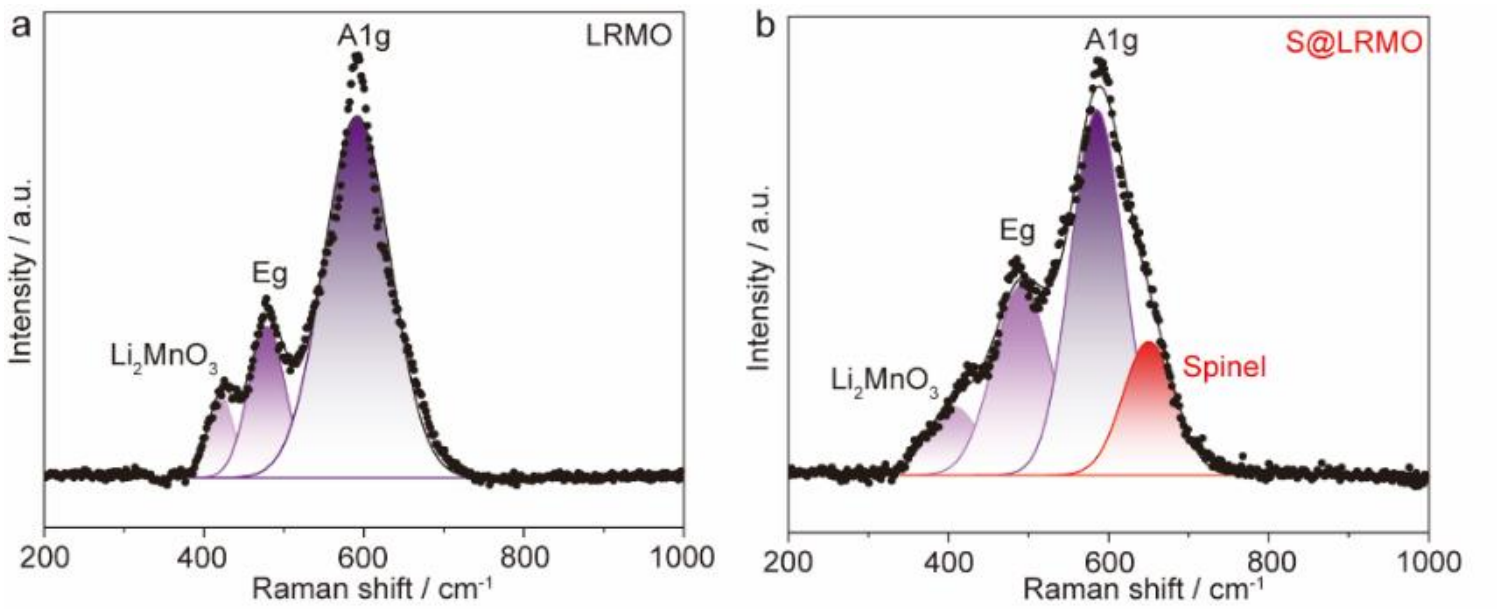

Figure R5 Raman spectroscopy results for both LRMO and S@LRMO.

Taken together, these complementary techniques conclusively demonstrate that the thiourea-derived treatment induces a chemically bonded, sub-nanometer-thick sulfur-rich coating on top of a spinel-like reconstructed surface layer. We believe this dual-layer surface architecture is critical for simultaneously mitigating oxygen-related degradation and facilitating Li^+ transport, thereby accounting for the improved electrochemical performance observed in solid-state batteries.

To address the reviewer's concern, we have substantially expanded the surface characterization section and incorporated the above analyses into the main manuscript and Supplementary Information. The revised figures and text include:

- **Figure 1:** Comprehensive cathode material characterization (sXRD, SEM, HAADF-STEM, HRTEM, EELS, line-scan profiles, and XPS)
- **Figure S9:** Raman spectroscopy of LRMO and S@LRMO

And we added this part related to TEM and Raman spectroscopy in the main discussion in the revised manuscript and revised Supplementary information, as follows:

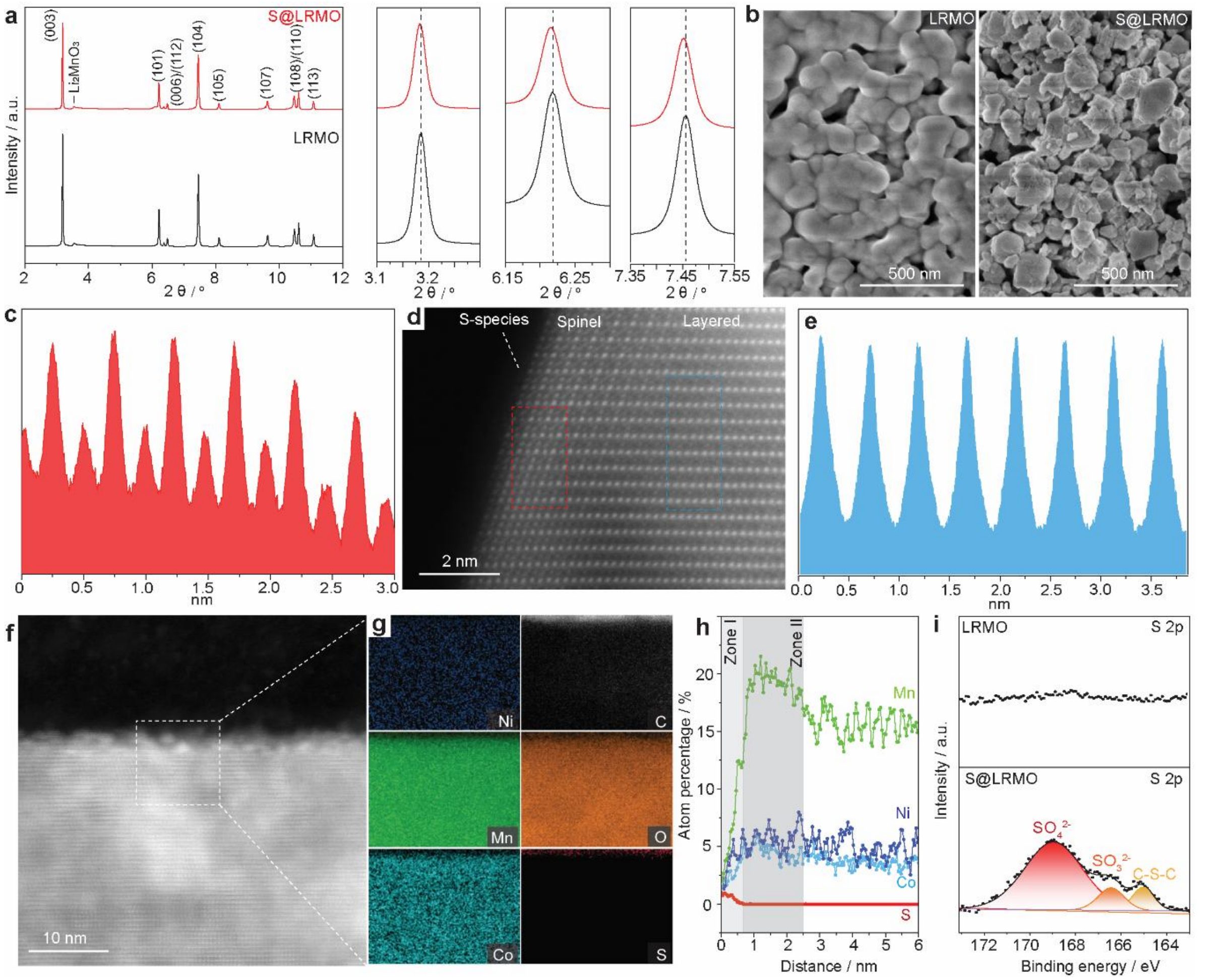

Figure 1*. *Cathode materials characterizations. *(a) Synchrotron X-Ray Diffraction (SXRD) patterns collected from the bare LRMO and S@LRMO powders; (b) SEM images of bare LRMO (left) and S@LRMO (right) particles; (d) High-Angle Annular Dark-field Scanning Transmission Electron Microscopy (HAADF-STEM) image from a S@LRMO cathode particle and the lattice space (c&e) of selected areas from its surface and bulk.(f) HAADF-STEM image from a S@LRMO cathode particle and its corresponding element maps in (g) extracted from the electron energy loss spectroscopy (EELS) data after mapping the region inside the white dashed box in the STEM image. (h)* Element line profiles based on the maps in *(g); (i)* S 2p XPS spectrum for bare LRMO and S@LRMO particles.

"Besides, the existence of spinel phase was also evidenced by Raman spectrum of S@LRMO. Specifically, a small shoulder peak at near 652 cm^{-1} emerged, which is related to the Mn-O stretching vibration in the spinel structure (Figure S9)."

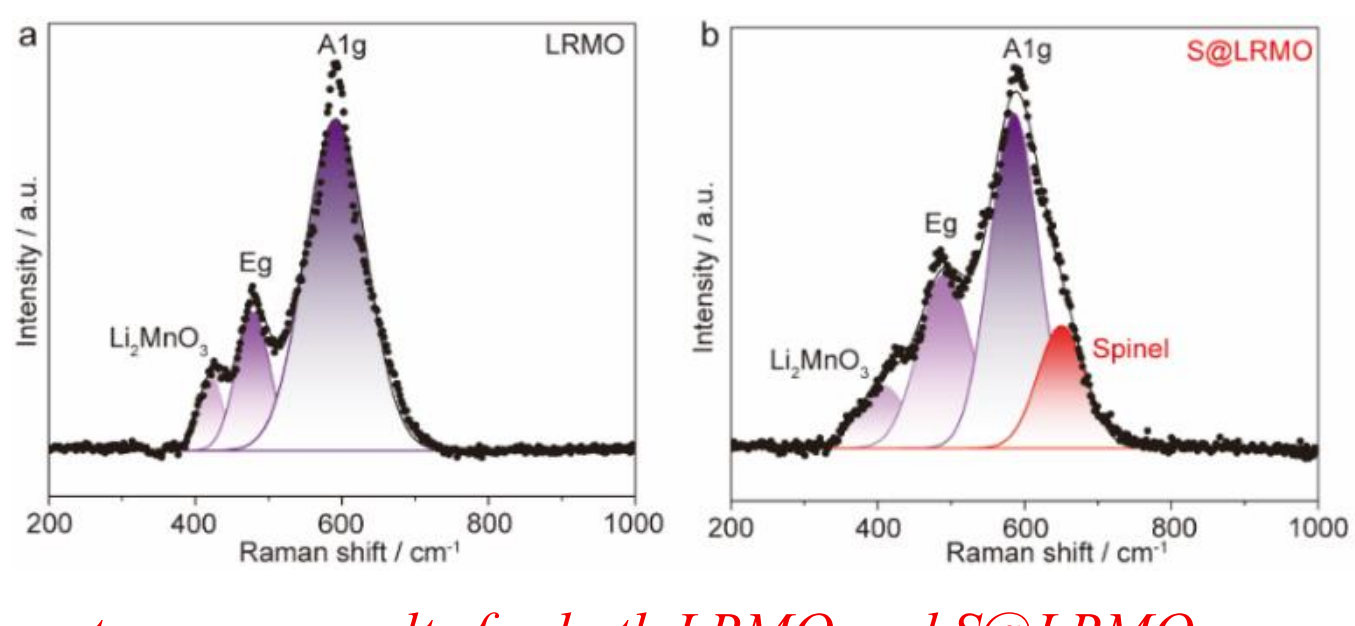

*"**Figure S9.** Raman spectroscopy results for both LRMO and S@LRMO.*

4. Unexplained Spinel Phase Formation: In Figure 2, the manuscript claims that Mn is enriched

near the particle surface, suggesting the formation of a $LiMn_2O_4$-like spinel phase. However, there is no explanation of how the thiourea-derived coating induces this phase transition. A mechanistic discussion is needed to substantiate this claim.

Response:

Thank you for your advice. It is supposed that the spinel phase was formed based on the reactions shown below. When LRMO particles with an alkaline surface are introduced into the solvent, thiourea decomposes according to Reaction (1):

$$SC(NH_2)_2 + 2OH^- \rightarrow S^{2-} + CO(NH_2)_2 + H_2O \quad (1)$$

Subsequently, the generated $CO(NH_2)_2$ further decomposes to produce NH_3, as described by Reaction (2). The absence of a discernible N 1s signal in the XPS spectra (Fig. S13) is consistent with the volatilization of nitrogen-containing species during this process:

$$2CO(NH_2)_2 \rightarrow NH_3 + 2HCNO \quad (2)$$

During the annealing step, the produced S^{2-} species and/or NH_3 act as reducing agents, promoting the partial reduction of Li_2MnO_3 and inducing its transformation into the spinel $LiMn_2O_4$ phase. Concurrently, S^{2-} is oxidized to sulfite and sulfate species, as summarized in Reaction (3):

$$O_2 + S^{2-} + Li_2MnO_3 \rightarrow SO_3^{2-} + SO_4^{2-} + LiMn_2O_4 \quad (3)$$

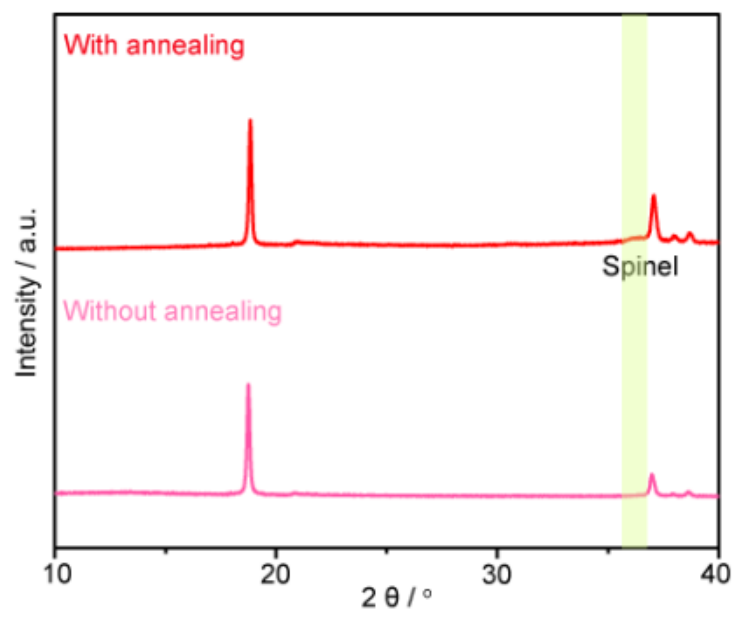

Figure R6 XRD patterns for coated LRMO without and with annealing process.

To make it clear, we integrated this part into main text and Supplementary information in the revised manuscript and revised Supplementary information, as follows:

"Notably, TM ion signals exhibit shifts toward higher oxidation states, likely resulting from oxidation during the annealing process.

Based on the above results and analyses, we propose that the formation of sulfur-containing species and the spinel phase proceeds via the following reactions. When LRMO particles with an alkaline surface are introduced into the solvent, thiourea decomposes according to Reaction (1):

$$SC(NH_2)_2 + 2OH^- \rightarrow S^{2-} + CO(NH_2)_2 + H_2O \quad (1)$$

Subsequently, the generated $CO(NH_2)_2$ further decomposes to produce NH_3, as described by Reaction (2). The absence of a discernible N 1s signal in the XPS spectra (Fig. S13) is consistent with the volatilization of nitrogen-containing species during this process:

$$2CO(NH_2)_2 \rightarrow NH_3 + 2HCNO \quad (2)$$

During the annealing step, the produced S^{2-} species and/or NH_3 act as reducing agents, promoting the partial reduction of Li_2MnO_3 and inducing its transformation into the spinel $LiMn_2O_4$ phase. Concurrently, S^{2-} is oxidized to sulfite and sulfate species, as summarized in Reaction (3):

$$O_2 + S^{2-} + Li_2MnO_3 \rightarrow SO_3^{2-} + SO_4^{2-} + LiMn_2O_4 \quad (3)"$$

And we also put this figure in Supplementary information.

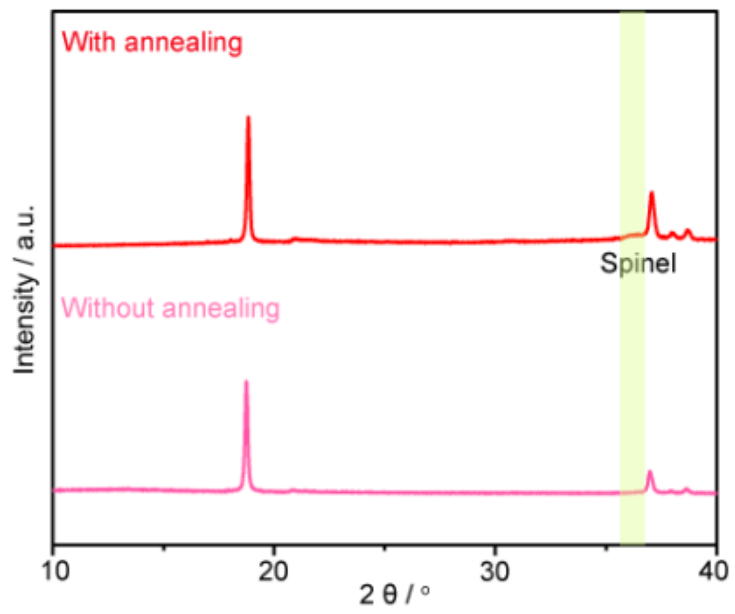

*"**Figure S14.** XRD patterns for coated LRMO without and with annealing process."*

5. Unclear Impact on Bulk Diffusivity: Figure 3c compares Li^+ diffusivity in LRMO and S@LRMO. However, the explanation lacks clarity and depth. Please provide a more rigorous discussion of how surface modification can influence bulk properties like diffusivity.

Response:

We thank the reviewer for this insightful comment regarding the relationship between surface modification and bulk Li^+ diffusivity. Although the sulfate/sulfite species are confined to the outer surface region, the surface treatment induces the formation of a spinel phase in the sub-surface region of S@LRMO.

As illustrated in the newly added schematic (Figure R7), S@LRMO exhibits a hierarchical structure consisting of an outer transition-metal (Co, Ni, Mn)-based sulfate/sulfite layer, an inner spinel phase at the sub-surface region, and the layered LRMO bulk. The spinel phase is known to possess three-dimensional Li^+ diffusion pathways, which can effectively reduce kinetic barriers for Li^+ transport at the surface–bulk interface.

We therefore propose that this sub-surface spinel layer serves as a fast Li^+ transport bridge between the surface and the layered bulk, facilitating Li^+ migration into the bulk lattice. This structural feature leads to a moderate but measurable enhancement in the apparent Li^+ diffusivity of S@LRMO, as evidenced by the GITT-derived diffusion coefficients shown in Fig. 3c. The manuscript has been revised accordingly to clarify this mechanism and to include the schematic illustration for better visualization.

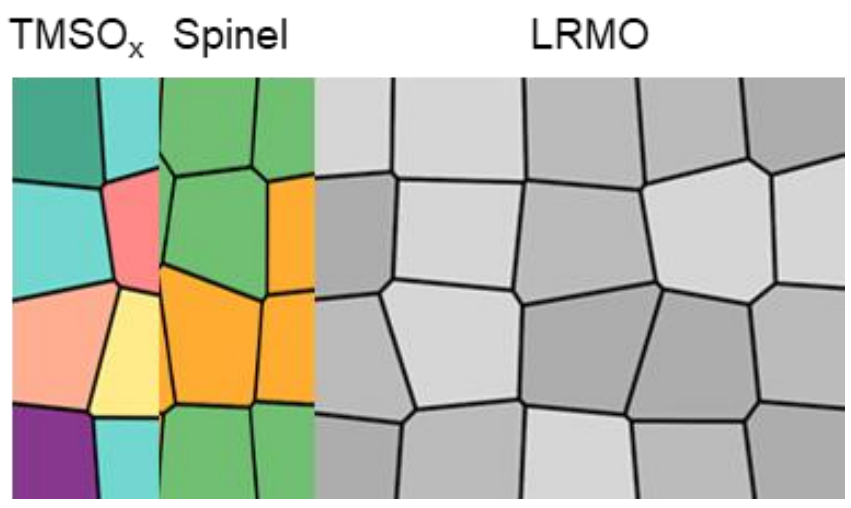

Figure R7 schematic illustration of S@LRMO.

6. Oxygen Redox Reversibility Not Addressed: Reversibility of the oxygen redox reaction is a critical parameter for LRMO performance but is not discussed in the manuscript. Techniques such as XAS, DEMS, or XPS could help clarify whether the thiourea coating improves oxygen redox reversibility. Additionally, the voltage decay during cycling for LRMO and S@LRMO should be discussed to evaluate oxygen redox reversibility.

Response:

We thank the reviewer for highlighting the importance of oxygen redox reversibility in evaluating the electrochemical performance of LRMO-based cathodes. In response to this comment, we have provided additional evidence to assess the reversibility of the oxygen redox reaction in both LRMO and S@LRMO.

Specifically, O 1s XPS spectra were collected for both electrodes at the fully charged state (4.6 V) and the fully discharged state (2.5 V) during the initial cycle, as shown in the newly added Figure R8. Deconvolution of the O 1s spectra reveals that the S@LRMO cathode maintains a higher fraction of oxidized oxygen species (O^-/O_2^{2-}) at both 4.6 V (37.3%) and 2.5 V (26.5%) compared with the pristine LRMO cathode (24.3% and 15.9%, respectively).

The larger retention of oxidized oxygen species upon discharge indicates a higher degree of reversibility of the oxygen redox process in S@LRMO. This improved reversibility is attributed to the synergistic effects of the sulfur-containing surface coating and the formation of a sub-surface spinel phase, which together help stabilize lattice oxygen and mitigate irreversible oxygen loss and surface reconstruction during cycling.

In addition, we have discussed the voltage evolution during cycling for both LRMO and S@LRMO in the revised manuscript. The reduced voltage decay observed for S@LRMO further supports the enhanced reversibility of the oxygen redox reaction, consistent with the XPS results. While advanced techniques such as XAS or DEMS would provide complementary insights, the combined XPS analysis and electrochemical evidence presented here sufficiently demonstrate the improved oxygen redox stability enabled by the thiourea-derived surface modification.

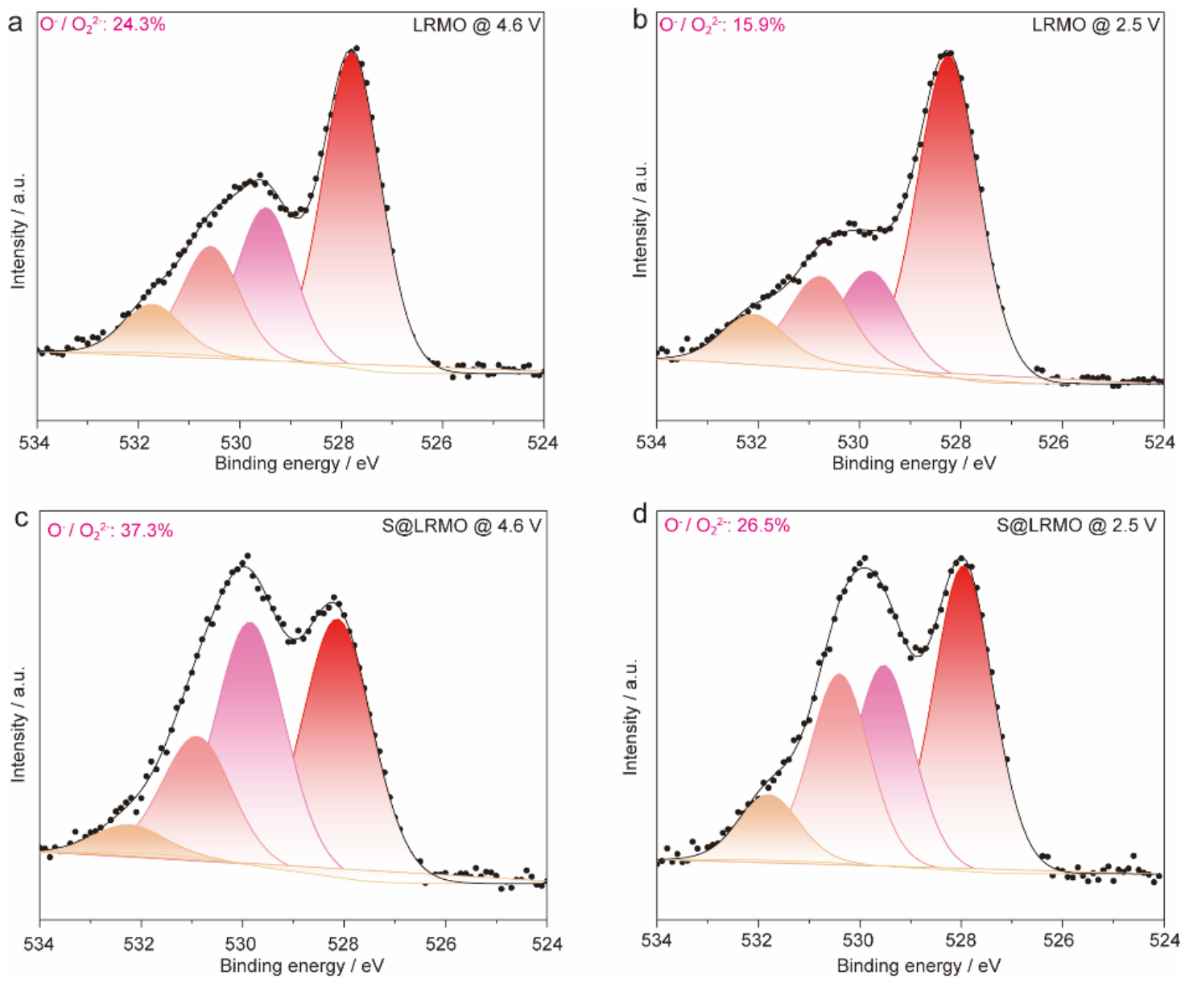

Figure R8 The XPS spectra of O 1s in the LRMO and S@LRMO cathode composite at initial charging state of 4.6 V and discharging state of 2.5 V.

To make it clear, we integrated this part into main text and Supplementary information in the revised manuscript and revised Supplementary information, as follows:

Origin of improved cell performance. *To elucidate the origin of the improved performance of S@LRMO, we employed a comprehensive suite of operando/in-situ and ex-situ characterization techniques, including ex-situ XPS, operando sXRD, and ToF-SIMS.*

The higher specific capacity of LRMO cathodes compared with conventional layered oxide cathodes (e.g., $LiCoO_2$ and $LiNi_xMn_yCo_zO_2$) originates from the additional contribution of oxygen redox reactions. However, the formation of O^-/O_2^{2-} species and, at high voltages, the possible evolution of O_2 can induce structural changes in LRMO, which may oxidize solid electrolytes at the cathode-electrolyte interface, generate Li^+-blocking species, and ultimately lead to sluggish reaction kinetics. Consequently, the reversibility of oxygen redox has generally been considered detrimental to the structural stability and Li^+ transport properties of LRMO cathodes.

To investigate the stability of oxygen redox reactions in LRMO and S@LRMO cathodes, O 1s XPS spectra were collected for both materials in the delithiated and lithiated states. Quantitative analysis reveals that the fraction of O^-/O_2^{2-} species is significantly higher in S@LRMO, reaching 37.3% at 4.6 V and 26.5% at 2.5 V, compared with 24.3% and 15.9%, respectively, for pristine LRMO (Fig. S25). These results indicate enhanced oxygen redox stability in the S@LRMO cathode. This improvement is likely associated with the presence of sulfur-containing surface species and the spinel phase, which stabilize lattice oxygen through a surface phase transformation.

And we also put this figure in Supplementary information.

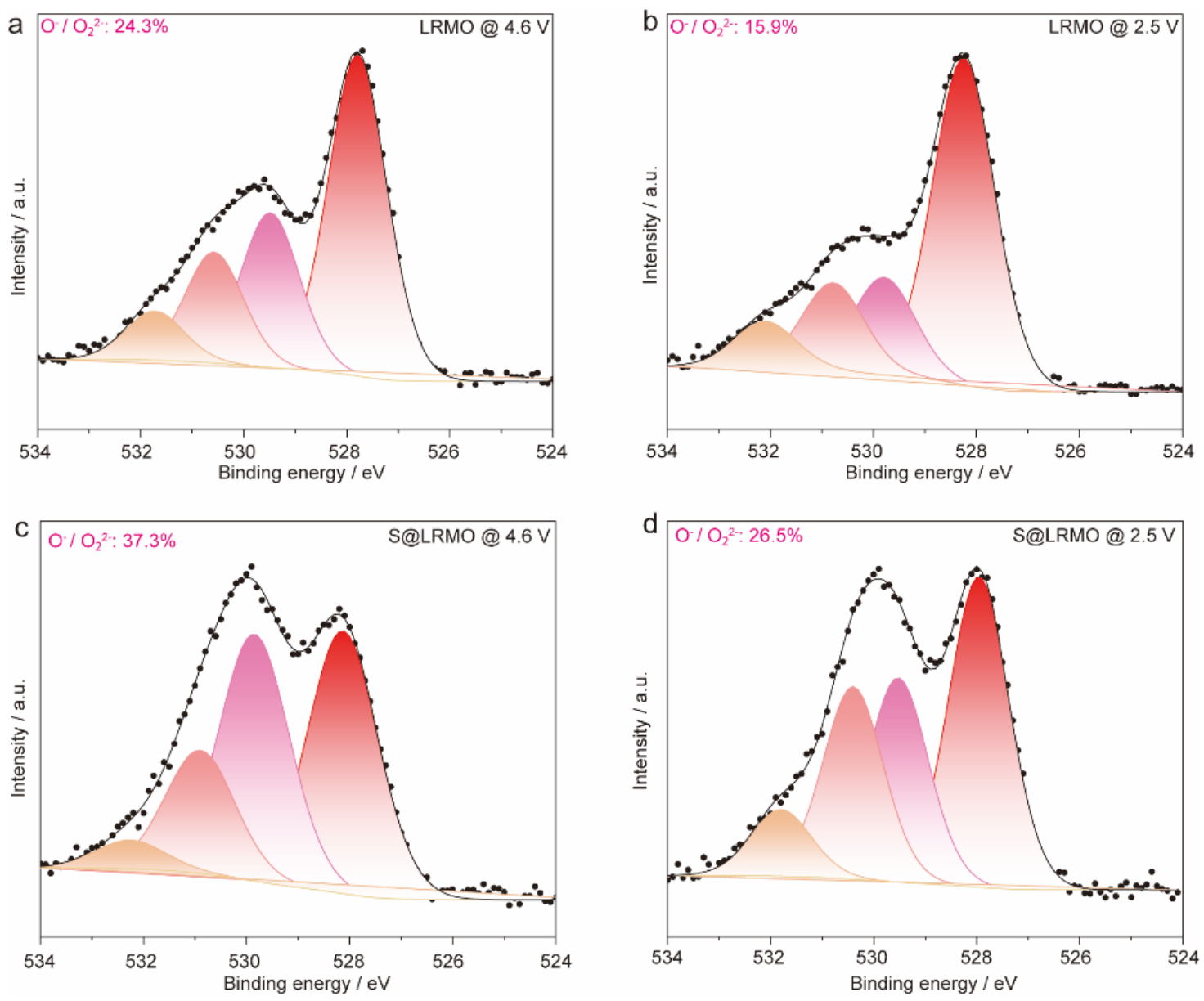

***Figure S25** The XPS spectra of O 1s in the LRMO and S@LRMO cathode composite at initial charging state of 4.6 V and discharging state of 2.5 V.*

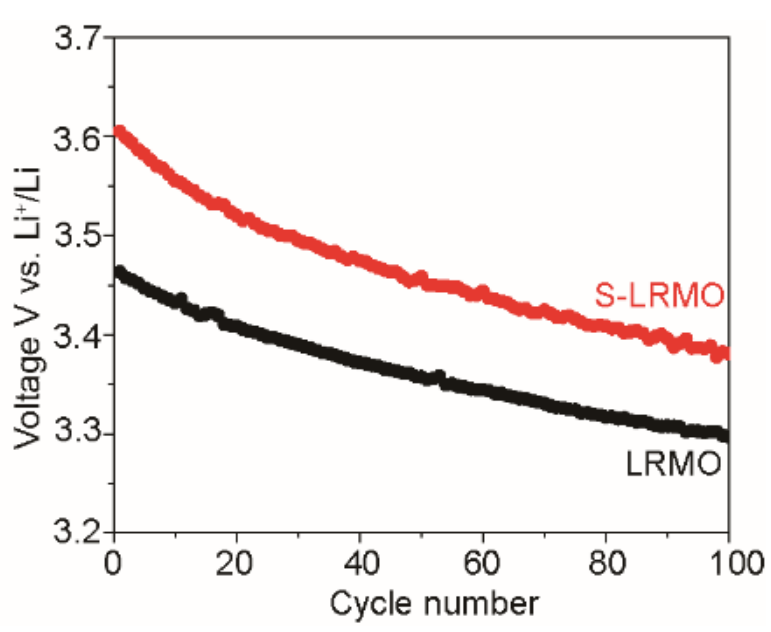

Figure R9 Curves for voltage changes for both LRMO and S@LRMO-based cell during 0.2C long-term cycling.

Compared with SSB-LRMO, SSB-S@LRMO exhibits a higher average discharge voltage in the initial cycles, which can be attributed to suppressed interfacial side reactions and a stabilized surface lattice (Fig. R9). However, such surface stabilization also alters the distribution of electrochemical and

mechanical driving forces associated with high-voltage oxygen redox.

Specifically, stresses that would otherwise be partially accommodated at the surface are more effectively transmitted into the particle interior. This redistribution may promote transition-metal migration and phase evolution within the bulk lattice during prolonged cycling. Consequently, although SSB-S@LRMO shows improved capacity retention, a faster decay of the average discharge voltage is observed, suggesting the emergence of a bulk-dominated degradation pathway.

7. Unsupported Claims in Conclusion: The conclusion asserts that the coating provides a "dual effect": formation of a stable interphase and surface lattice reconstruction, which jointly mitigate degradation and enhance Li-ion transport. However, the manuscript does not provide sufficient experimental evidence to support these claims. It is also unclear which of the two mechanisms plays a dominant role in improved electrochemical behavior. A more evidence-based, quantitatively supported conclusion is necessary.

Response:

We thank the reviewer for this constructive comment regarding the evidence supporting the proposed dual effect of the thiourea-derived coating. In the revised manuscript, we clarify the respective roles of interphase stabilization and surface lattice reconstruction by directly correlating experimental observations with electrochemical metrics.

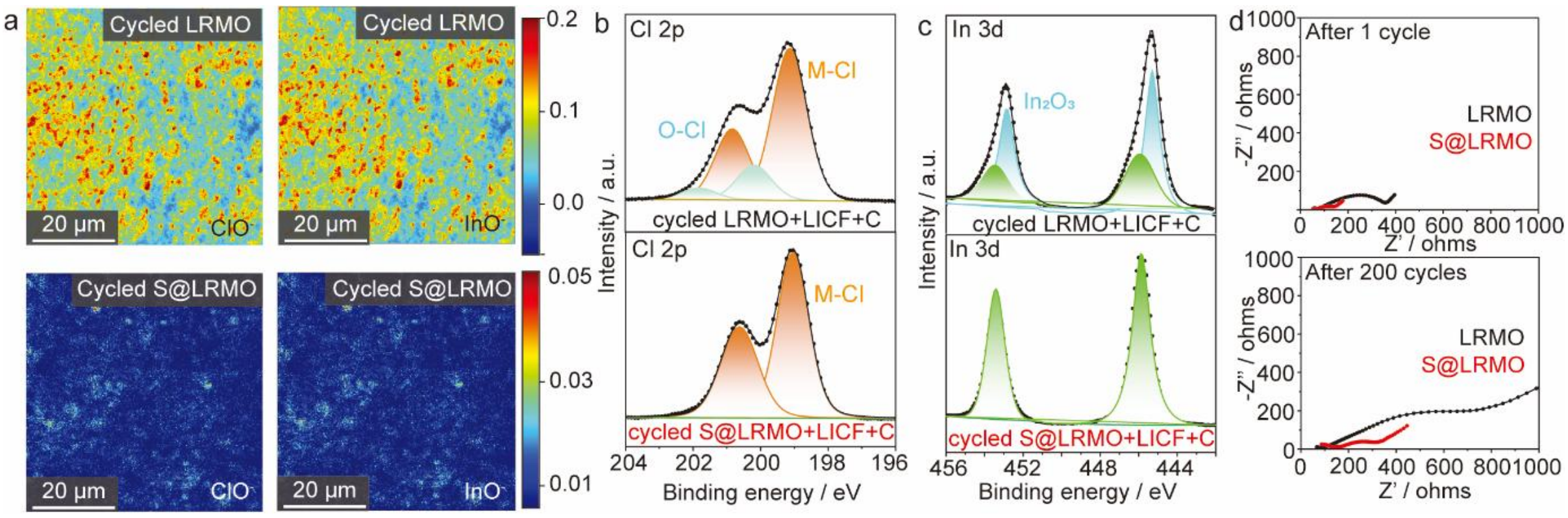

Figure R10 (a) TOF-SIMS graph of ClO⁻ and InO⁻ for cycled LRMO composites and cycled S@LRMO composites. XPS spectra of Cl 2p (b) and In 3d (c) for cycled LRMO composites and cycled S@LRMO composites. (d) The Nyquist plots of SSB-LRMO and SSB-S@LRMO after 1 and 200cycles with 0.2 C.

First, the formation of a chemically stable cathode-electrolyte interphase is strongly supported by TOF-SIMS and XPS analyses. As shown in Fig. R10a–c, the cycled S@LRMO composite exhibits significantly reduced signals associated with oxidized electrolyte decomposition products (InO^- and ClO^-) compared with pristine LRMO, indicating suppressed interfacial reactions with the LICF solid electrolyte. Consistently, electrochemical impedance spectroscopy reveals a substantially lower interfacial resistance for SSB-S@LRMO after prolonged cycling (Fig. R10d). These results identify interphase stabilization as the primary contributor to the mitigated interfacial degradation and improved cycling stability.

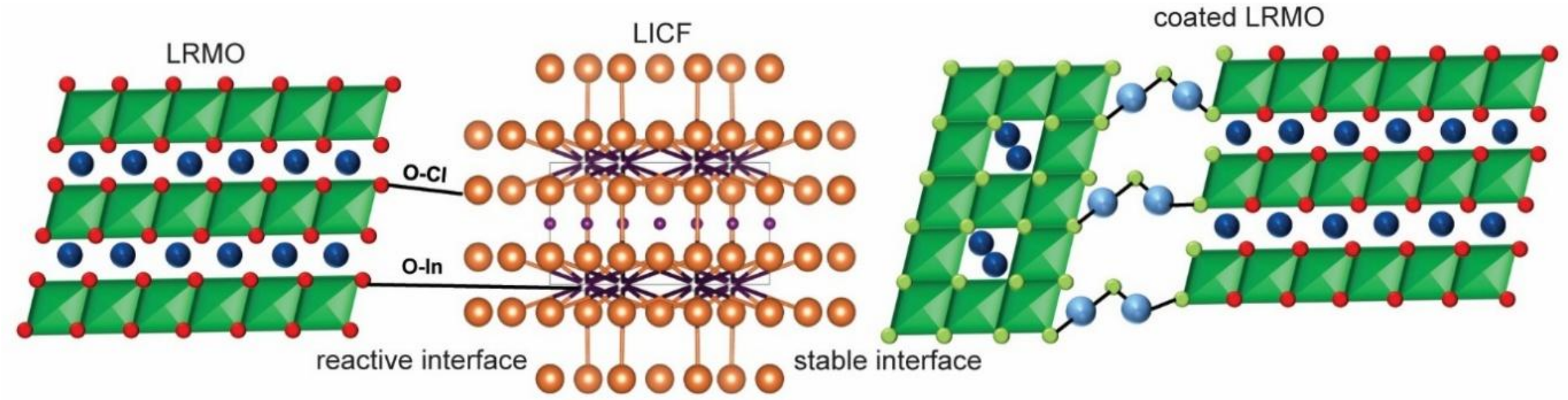

Figure R11 schematic illustration of interfaces of cathode and solid electrolytes for both LRMO | LICF and S@LRMO | LICF.

Second, surface lattice reconstruction into a spinel-like phase provides a complementary kinetic benefit. As schematically illustrated in Fig. R11, the spinel phase introduces three-dimensional Li-ion diffusion pathways at the cathode surface, which facilitate Li-ion transport across the cathode–electrolyte interface. This structural modification contributes to the reduced charge-transfer resistance and enhanced rate capability, but its impact is secondary to that of interfacial chemical stabilization.

Taken together, the experimental results indicate that while both mechanisms operate synergistically, suppression of interfacial reactions and stabilization of the cathode–electrolyte interphase play the dominant role in improving electrochemical performance, with surface lattice reconstruction primarily enhancing Li-ion transport kinetics. The conclusion has been revised accordingly to reflect this evidence-based assessment.

Reviewer #2 (Remarks to the Author)

This is an important study that demonstrates a thiourea-derived surface modification strategy to enhance the performances of lithium-rich manganese oxide (LRMO) cathodes in solid-state batteries (SSBs). The work addresses critical challenges such as oxygen-related degradation and sluggish Li-ion kinetics, offering a dual modification approach that combines sulfur-rich coating and spinel-like surface reconstruction. The electrochemical results are impressive and highlight the practical potential of this strategy, particularly the exceptional capacity retention of 97% after 600 cycles at 1 C. The use of advanced characterization/analysis techniques such as operando SXRD and DRT analysis provides evidence for the proposed mechanisms. While the manuscript is well-structured and the conclusions are largely supported by the data, the following points should be addressed to strengthen the work further.

1. While the SXRD and TEM data suggest the formation of a spinel-like surface structure ($LiMn_2O_4$), the mechanism driving this reconstruction remains unclear. Is the spinel phase formed through Mn migration from the bulk during annealing, or is it a result of sulfur species interacting with the LRMO surface? A deeper discussion is necessary.

Response:

Thank you for your advice. It is supposed that the spinel phase was formed based on the reactions shown below.

When LRMO particles with an alkaline surface are introduced into the solvent, thiourea decomposes according to Reaction (1):

$$SC(NH_2)_2 + 2OH^- \rightarrow S^{2-} + CO(NH_2)_2 + H_2O \quad (1)$$

Subsequently, the generated $CO(NH_2)_2$ further decomposes to produce NH_3, as described by Reaction (2). The absence of a discernible N 1s signal in the XPS spectra (Fig. S13) is consistent with the volatilization of nitrogen-containing species during this process:

$$2CO(NH_2)_2 \rightarrow NH_3 + 2HCNO \quad (2)$$

During the annealing step, the produced S^{2-} species and/or NH_3 act as reducing agents, promoting the partial reduction of Li_2MnO_3 and inducing its transformation into the spinel $LiMn_2O_4$ phase. Concurrently, S^{2-} is oxidized to sulfite and sulfate species, as summarized in Reaction (3):

$$O_2 + S^{2-} + Li_2MnO_3 \rightarrow SO_3^{2-} + SO_4^{2-} + LiMn_2O_4 \quad (3)$$

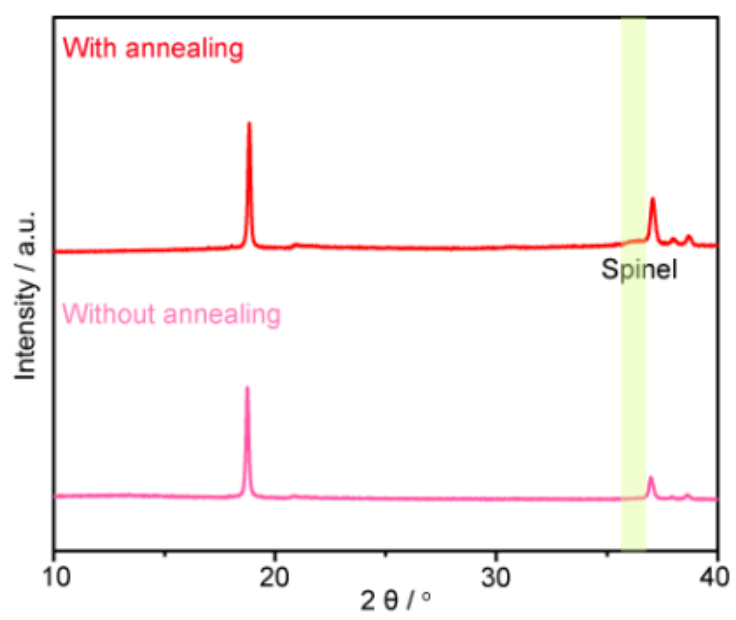

Figure R12 XRD patterns for coated LRMO without and with an annealing process.

To make it clear, we integrated this part into main text and Supplementary information in the revised manuscript and revised Supplementary information, as follows:

"Notably, TM ion signals exhibit shifts toward higher oxidation states, likely resulting from oxidation during the annealing process.

Based on the above results and analyses, we propose that the formation of sulfur-containing species

and the spinel phase proceeds via the following reactions. When LRMO particles with an alkaline surface are introduced into the solvent, thiourea decomposes according to Reaction (1):

$$SC(NH_2)_2 + 2OH^- \rightarrow S^{2-} + CO(NH_2)_2 + H_2O \quad (1)$$

Subsequently, the generated $CO(NH_2)_2$ further decomposes to produce NH_3, as described by Reaction (2). The absence of a discernible N 1s signal in the XPS spectra (Fig. S13) is consistent with the volatilization of nitrogen-containing species during this process:

$$2CO(NH_2)_2 \rightarrow NH_3 + 2HCNO \quad (2)$$

During the annealing step, the produced S^{2-} species and/or NH_3 act as reducing agents, promoting the partial reduction of Li_2MnO_3 and inducing its transformation into the spinel $LiMn_2O_4$ phase. Concurrently, S^{2-} is oxidized to sulfite and sulfate species, as summarized in Reaction (3):

$$O_2 + S^{2-} + Li_2MnO_3 \rightarrow SO_3^{2-} + SO_4^{2-} + LiMn_2O_4 \quad (3)$$ *"*

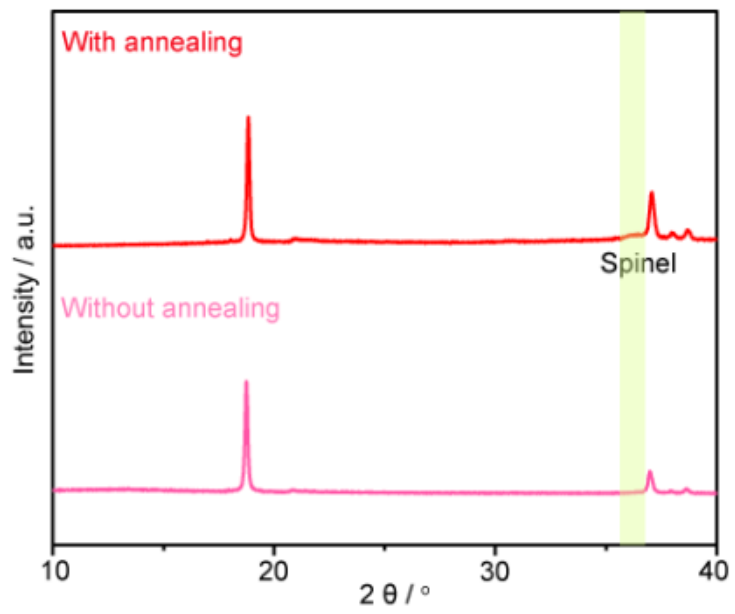

***"Figure S13.** XRD patterns for coated LRMO without and with annealing process."*

2. The GITT-derived Li+ diffusion coefficients (Figure 3c) show improved kinetics for S@LRMO. Including numerical data (e.g., D(Li+) values at different voltages) and comparing them with previous literature reports would quantify the enhancement attributable to this phase. Moreover, could sulfur species react with LICF during cycling, leading to interfacial degradation?

Response: We thank the reviewer for the suggestion to further quantify the enhancement in Li^+ diffusion kinetics and to clarify the interfacial stability of sulfur-containing species. In response, we have included a quantitative comparison of GITT-derived Li^+ diffusion coefficients and additional interfacial analyses in the revised manuscript.

Table R1. The comparison of $D(Li^+)$ values of LRMO shown in the previous literature and this work.

materials	$D(Li^+)$ ($cm^2\ s^{-1}$)	Ref.
Li_2SO_4@LRMO	10^{-9} to 10^{-11}	1
Li_2WO_4@LRMO	10^{-13} to 10^{-16}	2
$Li_2B_4O_7$@LRMO	10^{-10} to 10^{-12}	3
S@LRMO	10^{-9} to 10^{-11}	This work

As summarized in Table R1, the GITT-derived Li^+ diffusion coefficients of S@LRMO in this work fall in the range of ~10^{-9} to 10^{-11} $cm^2\ s^{-1}$, which is comparable to or higher than those reported for various surface-modified LRMO cathodes, including Li_2SO_4@LRMO, $Li_2B_4O_7$@LRMO, and Li_2WO_4@LRMO. We note that direct quantitative comparison across different studies is inherently challenging due to variations in material synthesis, particle morphology, and testing conditions, even for nominally identical compositions. Therefore, we emphasize that the most reliable assessment arises from the direct comparison between pristine LRMO and S@LRMO within the same experimental framework, where a consistent, albeit moderate, increase in $D(Li^+)$ is observed after surface modification (Fig. 3c).

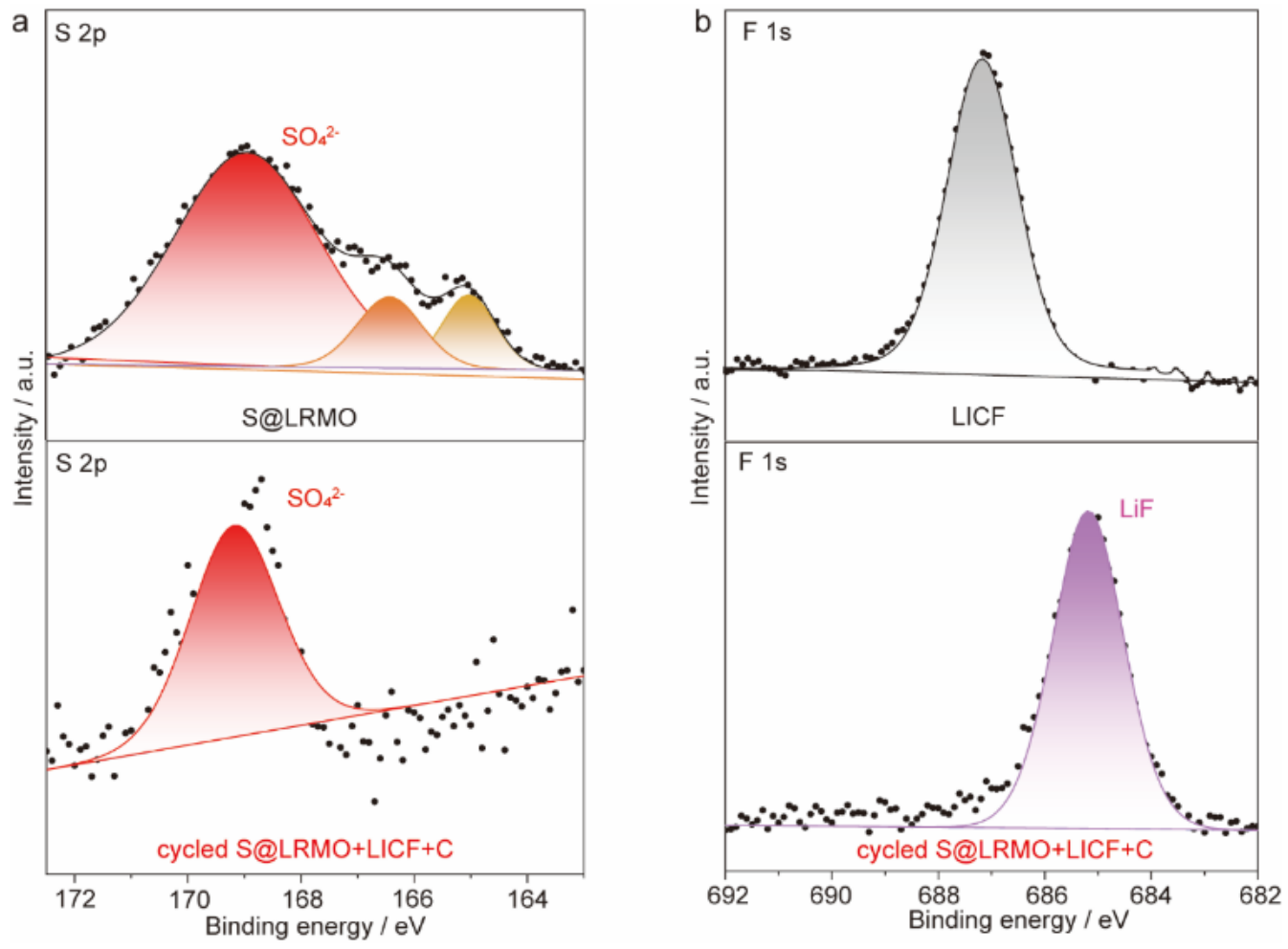

Figure R13 The F 1s (a) and S 2p (b) XPS spectra of fresh cathode components (S@LRMO and LICF) and cycled S@LRMO cathode composite.

Regarding the potential reactivity of sulfur species with the LICF solid electrolyte, XPS analyses were performed on cycled S@LRMO cathode composites. As shown in Fig. R13a, the S 2p spectrum remains dominated by SO_4^{2-} species after cycling, indicating that the sulfur-containing coating is chemically stable at the cathode–electrolyte interface. Meanwhile, the appearance of a peak at 685.1 eV in the F 1s spectrum (Fig. R13b), assigned to LiF, suggests the formation of a LiF-rich interphase during cycling[4,5]. Such LiF-containing interphases are widely recognized to enhance interfacial stability under high-voltage conditions. Together, these results demonstrate that the sulfur species do not induce detrimental interfacial reactions with LICF but instead contribute to the formation of a stable cathode–electrolyte interface.

3. The cycling results at 1 C and 25°C are good, but practical SSBs often operate under a wide range of temperatures or higher current densities. Testing the S@LRMO cathode at different temperatures (e.g., −20 to 60°C) or extreme rates (e.g., 3–5 C) would provide insights into the robustness of the coating under realistic conditions. Additionally, post-cycling structural analysis could confirm whether the spinel phase and sulfur-rich layer remain intact after prolonged operation. Also, scalability considerations, such as particle aggregation during annealing, solvent choice for large-scale processing, should be addressed.

Response:

We thank the reviewer for the constructive suggestions regarding the robustness, structural stability, and scalability of the S@LRMO cathode under practical operating conditions. In response, we have conducted additional experiments and expanded the discussion in the revised manuscript.

i) **Temperature robustness:** Considering that practical solid-state batteries may operate over a broad temperature range, we evaluated the electrochemical performance of S@LRMO at an elevated temperature of 60 °C. As shown in Fig. R14, the S@LRMO-based cell delivers an increased discharge capacity of approximately 250 mAh g^{-1} at 0.1 C during activation, exceeding the capacity obtained at room temperature. At a higher rate of 1 C, however, the long-term cycling stability is reduced, with ~70% capacity retention after 50 cycles. This result suggests that while elevated temperature enhances reaction kinetics, it also accelerates degradation processes at high current densities, highlighting the trade-off between kinetics and stability under harsh operating conditions.

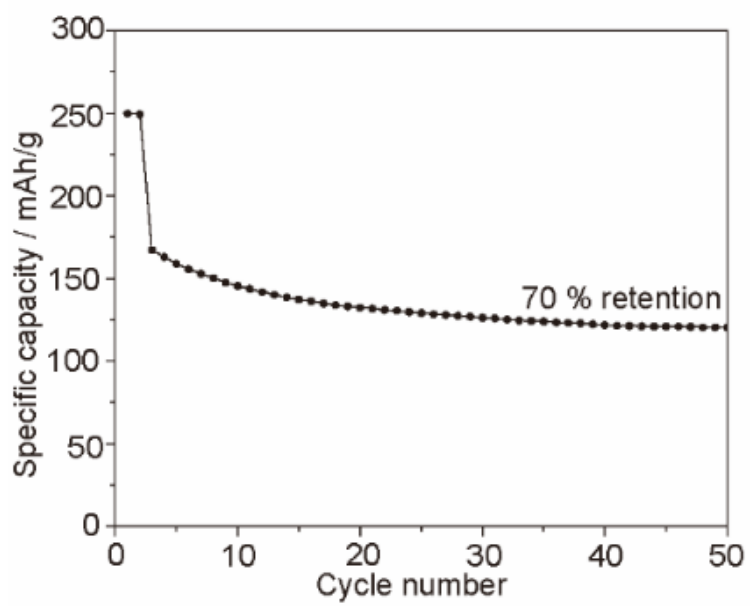

Figure R14 The long-term cycling performance of S@LRMO under 60 °C.

ii) **Post-cycling structural integrity:** To directly assess whether the surface reconstruction and sulfur-rich coating persist during prolonged cycling, post-mortem structural analyses were performed. TEM images of cycled S@LRMO particles (Fig. R15a) reveal that both the coating layer and the sub-surface spinel phase remain clearly identifiable after cycling. In addition, the S 2p XPS spectrum of the cycled cathode (Fig. R15b) exhibits a characteristic peak at ~169 eV, corresponding to SO_4^{2-} species, indicating that the sulfur-containing layer retains its chemical identity. These results confirm that the coating and spinel reconstruction are structurally and chemically stable under electrochemical operation.

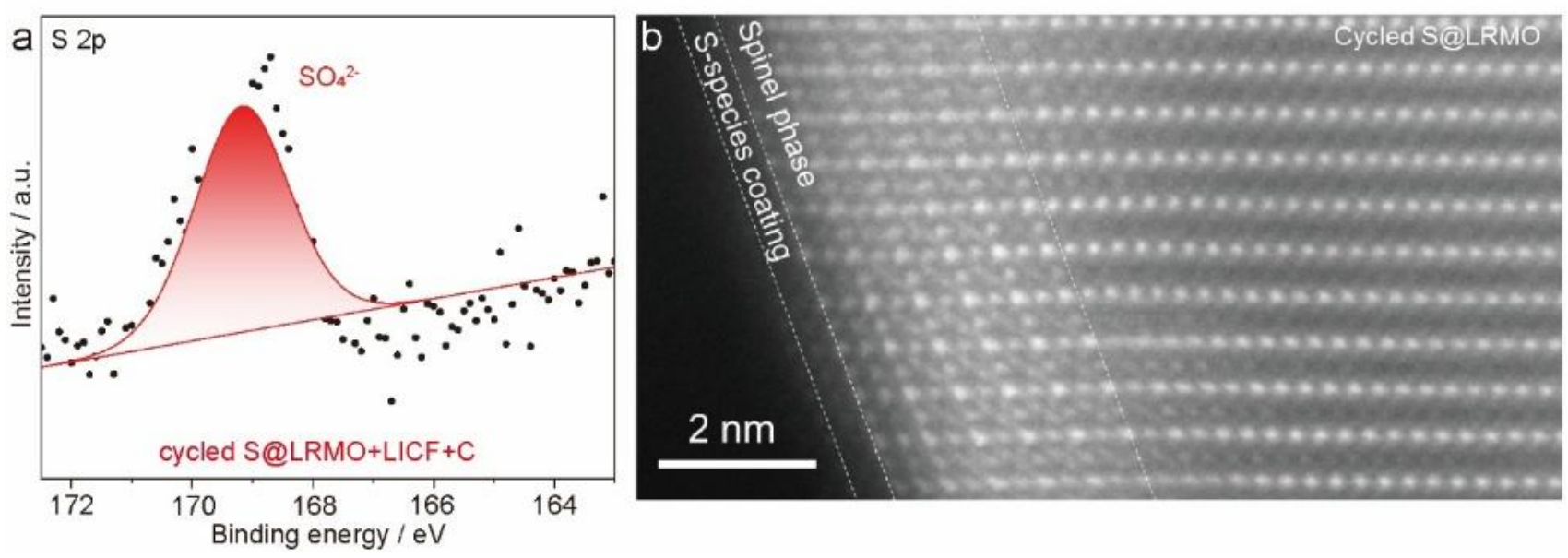

Figure R15. S 2p XPS spectrum of cycled S@LRMO cathode components (a) and TEM image of cycled S@LRMO cathode particle (b).

iii) **Scalability considerations:** From a processing perspective, deionized water was employed as the solvent for the coating process, which is advantageous for large-scale manufacturing due to its low cost, environmental compatibility, and ease of handling. Regarding potential particle aggregation during annealing, SEM observations (Fig. R16) indicate that aggregation is minimal for the micron-sized LRMO secondary particles used in this study. Although partial fragmentation of pristine secondary particles occurs during the coating process, this structural refinement does not adversely affect electrochemical performance. On the contrary, the reduced particle size can shorten Li-ion and electron transport pathways and improve interfacial contact with the solid electrolyte. This interpretation is consistent with previous reports in which intentional fragmentation of secondary LRMO particles via mechanical processing was shown to enhance electrochemical performance[3].

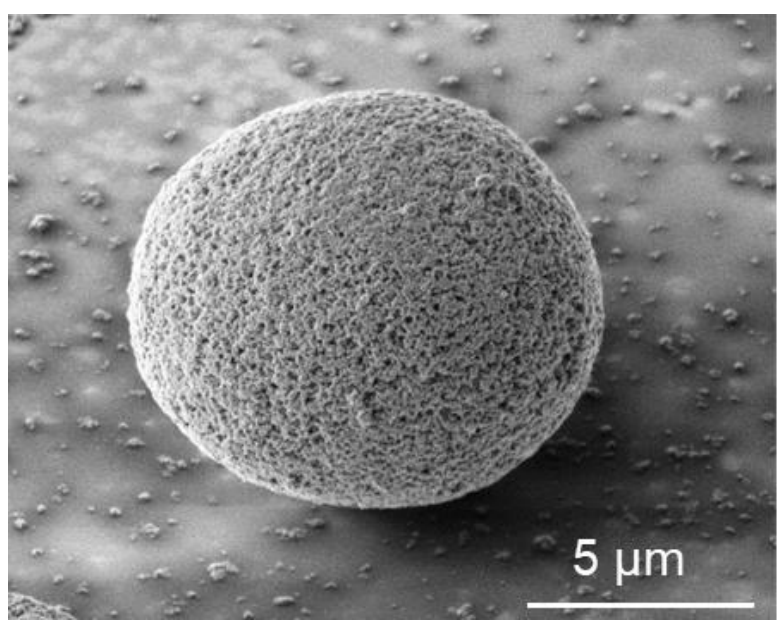

Figure R16. SEM image of a single LRMO secondary particle.

Overall, these additional results demonstrate that the S@LRMO cathode exhibits reasonable robustness under elevated-temperature operation, maintains its designed surface structure after cycling, and is compatible with scalable, water-based processing routes.

And we also integrated them into the main text and Supplementary information, as follows:

In the main text:

***"Coating effectiveness during long-term cycling.** To verify the persistence of the sulfur-containing surface coating and the spinel phase on LRMO particles after prolonged cycling, post-mortem analysis based on XPS and TEM was conducted for S@LRMO cathode. XPS measurements detect sulfur-containing species on the cycled electrodes, with binding energies consistent with SO_4^{2-}(Fig. S29a). This observation is further corroborated by TEM analysis with spinel remaining on the surface of LRMO particle (Fig. S29b). Collectively, these results demonstrate that the ultrathin coating and surface spinel phase maintained their structural integrity during long-term electrochemical cycling".*

In the Supplementary information:

"

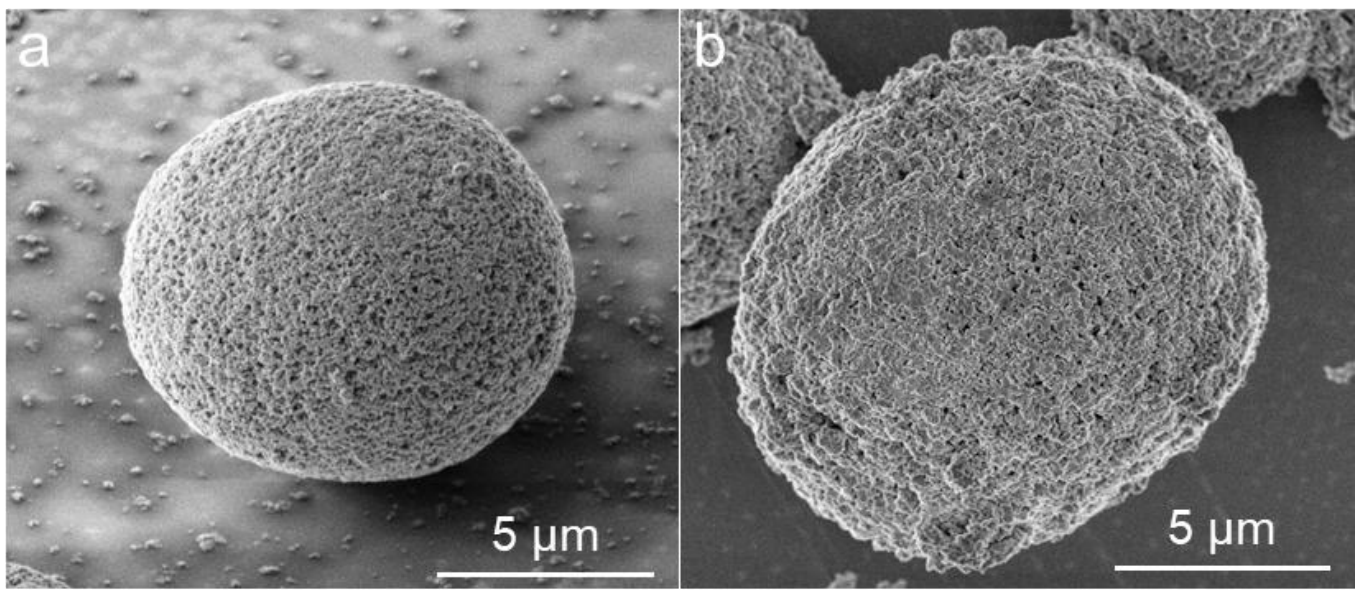

Figure S3. SEM image of LRMO (a) and S@LRMO (b).

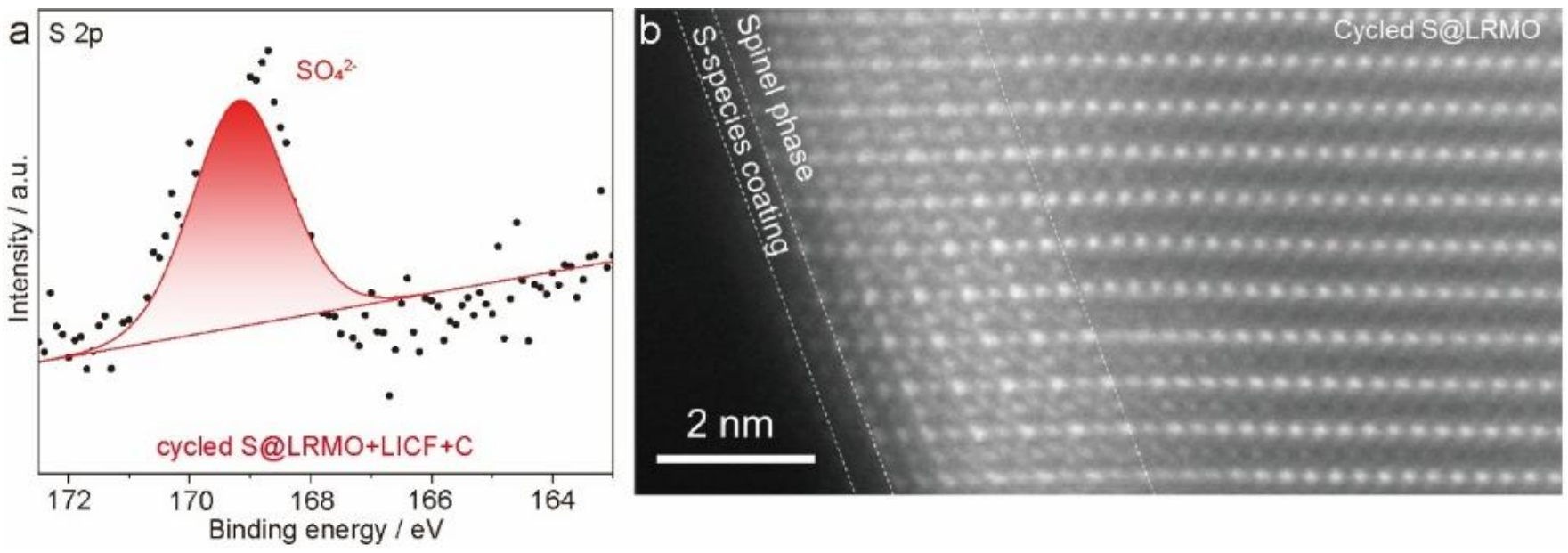

Figure S29. S 2p XPS spectrum of cycled S@LRMO cathode components (a) and TEM image of cycled S@LRMO cathode particle (b)."

4. The authors briefly compare S@LRMO with prior coatings (e.g., Li_3PO_4, Li_2WO_4) in Figure 2e but do not discuss the relative advantages of sulfur-based modification in depth. A table summarizing key metrics (e.g., ICE, capacity retention, coating thickness) for these systems would help contextualize the novelty and superiority of the thiourea-derived approach.

Response: thank you for your questions. A table summarized key metrics sit in Supplementary information (Table 4), and we also put it here to make it clear.

Table R2. Electrochemical performances of LRMO-based all solid-state batteries with different coatings.

LRMO	Coating	$V_{cut\text{-}off}$ (V)	IDC (mAh/g)	ICE (%)	Mass loading (mg/cm^2)	Long-term cycling performances
$Li_{1.2}Mn_{0.54}Co_{0.13}Ni_{0.13}O_2$	Li_2WO_4	4.6	218	74.83 %	7.64	81% retention after 1200 cycles at 1 C
$Li_{1.2}Mn_{0.54}Co_{0.13}Ni_{0.13}O_2$	Li_2SO_4	4.6	248	94 %	4.2	81% retention after 300 cycles at 1 C
$Li_{1.17}Mn_{0.55}Co_{0.05}Ni_{0.24}O_2$	Li_3PO_4	4.8	230	83.0 %	12.7	60 % retention after 431cycles at 0.2 C
$Li_{1.2}Mn_{0.54}Co_{0.13}Ni_{0.13}O_2$	$LiNbO_3$	4.8	221	73.21 %	0.88	67 % retention after 100 cycles at 0.05 C
$Li_{1.2}Mn_{0.54}Co_{0.13}Ni_{0.13}O_2$	$SC(NH_2)_2$	4.6	220	84.83 %	7.13	97 % retention after 600 cycles at 1 C

5. Some expressions are not very rigorous. For example, I do not believe that this strategy will "unlock LRMO's full potential" (introduction part). The phrase "suppressed oxygen-related degradation" is repeated excessively.

Response:

We thank the reviewer for pointing out the overly strong and repetitive expressions in the manuscript. We agree that certain phrases were not sufficiently rigorous and have revised the text accordingly.

Specifically, the statement "unlock LRMO's full potential" in the Introduction has been removed and replaced with more cautious, scientifically appropriate wording that avoids overstatement.

In addition, we acknowledge that the phrase "suppressed oxygen-related degradation" was used excessively throughout the manuscript. To improve clarity and readability, we have revised the text to reduce repetition and employed alternative expressions where appropriate, while maintaining the original scientific meaning.

These changes have been implemented throughout the revised manuscript.

And we also made the corresponding changes in the main text, shown below:

"Building upon these findings, we introduce a novel approach that integrates surface reconstruction in combination with an ultra-thin S-rich coating, addressing interfacial Incompatibility caused by O-involving reactions and limited kinetics in LRMO-based SSBs. This strategy aims to overcome the persistent limitations of previous surface treatments and unlock LRMO's potential. This is realized by immersing LRMO ($Li_{1.2}Mn_{0.56}Ni_{0.13}Co_{0.13}O_2$) in a thiourea ($SC(NH_2)_2$)-based solution followed by annealing."

And to avoid excessive repeating of "**suppressed oxygen-related degradation**", we rephrase the corresponding in the main text, shown below:

"Operando synchrotron X-ray diffraction and ex-situ spectroscopic analyses confirmed that the sulfur modification effectively blocked oxygen-related degradation and stabilized the LRMO structure during high-voltage cycling."

By integrating a thiourea-derived coating, we achieved a dual effect: the formation of a stable interphase that mitigates interfacial degradation and a surface lattice reconstruction that weakens oxygen loss and TM ion migration as well as enhances Li-ion transport (The functionality of the coating is summarized in Figure 5).

"Our findings demonstrate that the synergistic effects of surface reconstruction and sulfur- based functionalization can effectively mitigate oxygen-related degradation while enhancing Li-ion transport in LRMO cathodes."

"This dual modification mitigates oxygen-related degradation and accelerates Li^+ transport through

the surface-near spinel phase.”

“In contrast, S@LRMO exhibits higher D(Li+) values at high SOC compared to LRMO, likely due to suppressed eliminated interfacial reactions at the LICF | LRMO interface and reduced irreversible phase transitions induced by oxygen release at high voltage.”

Reviewer #3 (Remarks to the Author):

The manuscript "NCOMMS-25-19137-T" reported a modified LRMO cathode (S@LRMO), which exhibits an exceptional initial discharge capacity and outstanding capacity retention. And the origin of the improved electrochemical performance is discussed. It can be accepted in nature communication before a major revision, answering the following questions.

1. During the XRD refinement, for the 3a site, why was the site occupancy refined as Li + Mn = 1, but not Li+Ni=1? As the cation mixing is usually suggested to occur between Li^+ and Ni^{2+}.

Response:

We thank the reviewer for this insightful question regarding the site-occupancy constraints used in the Rietveld refinement.

The material studied in this work is a lithium-rich layered oxide with the nominal composition $Li_{1.2}Mn_{0.54}Ni_{0.13}Co_{0.13}O_2$, which can be described as a composite structure consisting of a layered α-$NaFeO_2$ ($\bar{R}3m$) phase and a Li_2MnO_3-type component. Owing to this intrinsic Li_2MnO_3-related ordering, the 3a site (nominally the Li layer in $\bar{R}3m$) is not exclusively occupied by Li but is partially occupied by Mn in an ordered manner.

In conventional Ni-containing layered oxides, cation mixing typically refers to $Ni^{2+} \leftrightarrow Li^+$ antisite defects, and refining the 3a site occupancy as Li + Ni = 1 is therefore appropriate. However, in lithium-rich manganese oxides, the presence of Mn in the Li layer is an inherent structural feature rather than a defect-related cation mixing process. Consequently, constraining the 3a site occupancy as Li + Mn = 1 is necessary to correctly reflect the Li_2MnO_3-type ordering and to satisfy the overall stoichiometry of the compound.

We note that any possible Ni occupation of the 3a site is expected to be minimal compared to the intrinsically ordered Mn population and is difficult to reliably resolve by XRD refinement due to the similar scattering factors of transition metals. Therefore, the Li + Mn = 1 constraint was adopted as a physically meaningful and widely accepted approach for structural refinement of lithium-rich layered oxides.

2. Please present the in-situ XRD cell design. Is there a high pressure offered during the in-situ XRD test? Please provide the rate of the operando cell, and the related capacities of the cells.

Response:

We thank the reviewer for the request to clarify the design and operating conditions of the operando XRD measurements.

i) Operando cell design and pressure conditions: The design of the operando XRD cell is presented in Fig. R17. The setup consists of a stacked solid-state cell assembly integrated with a pressure controller, pressure sensor, and piezoelectric actuator, enabling dynamic control of the applied stack pressure during data acquisition. All operando sXRD measurements were conducted under a constant uniaxial pressure of 150 MPa, which is within the typical pressure range used for solid-state battery operation and ensures sufficient interfacial contact throughout cycling.

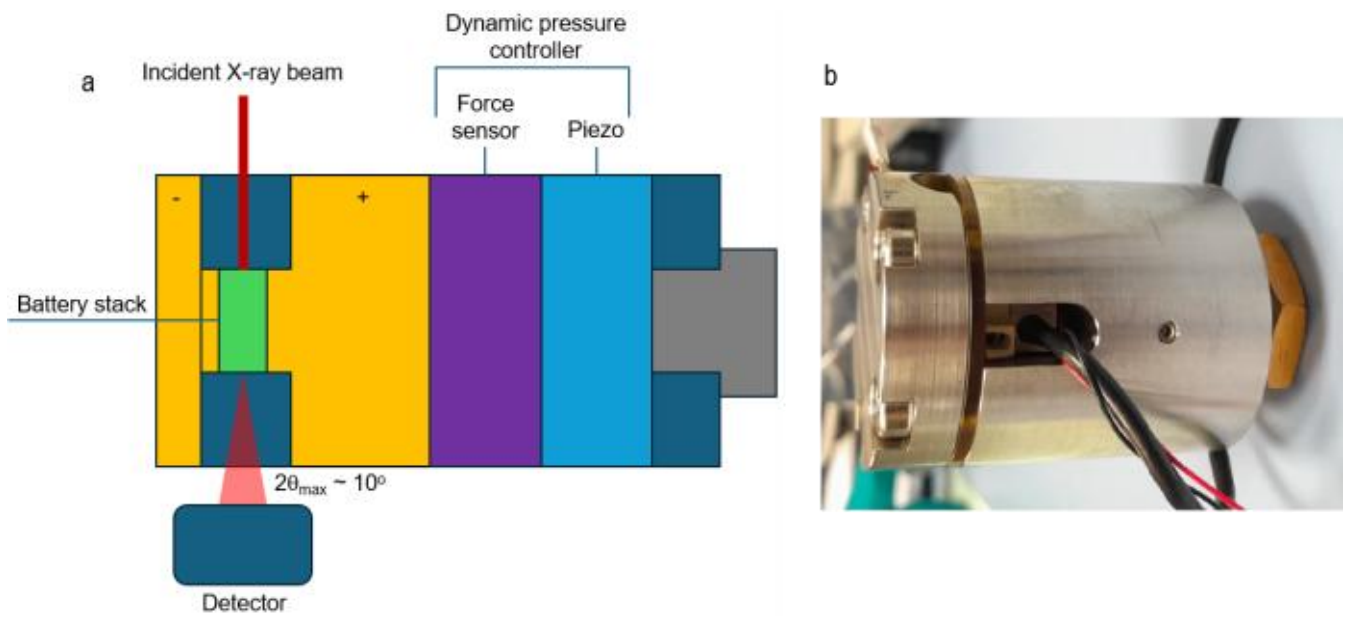

Figure R17 Operando XRD setup. (a) Schematic of the setup, which contains dynamic pressure control using a piezo. (b) Photo of the actual device.

ii) Electrochemical conditions: The operando XRD experiments were carried out at a constant current of 35 μA, corresponding to a low effective C-rate. This relatively small current was selected to accommodate the limited synchrotron beamtime and to ensure sufficient temporal resolution for capturing structural evolution during cycling. The corresponding galvanostatic charge–discharge profiles of both LRMO- and S@LRMO-based operando cells in the initial cycle are shown in Fig. R18.

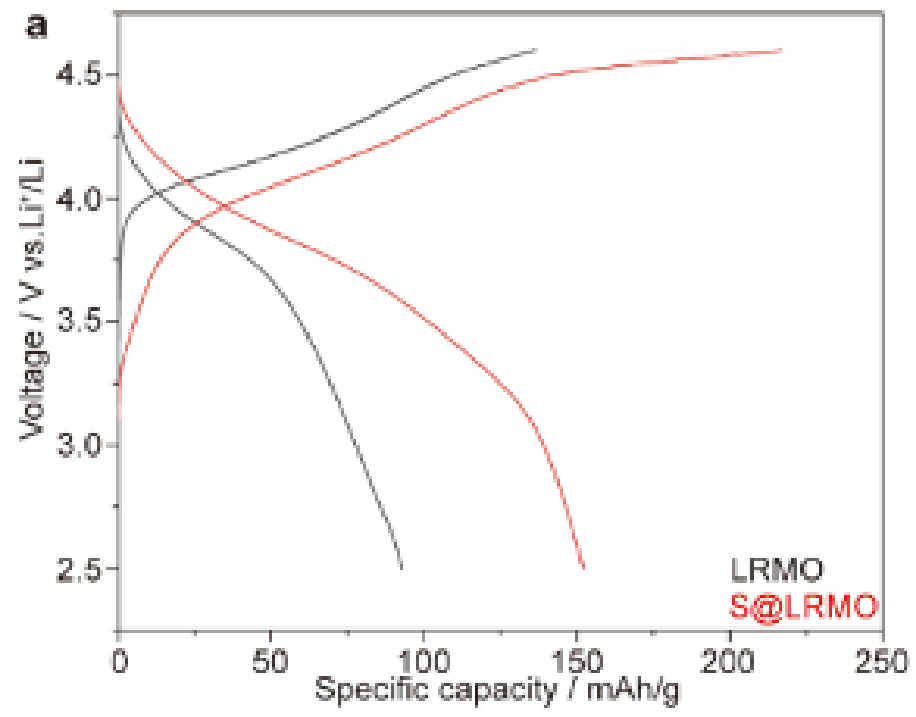

Figure R18 Galvanostatic charge-discharge curves for LRMO and S-LRMO based operando cells in the initial cycle.

iii) Capacity determination: We note that the operando XRD acquisition software did not directly record the specific capacity during the measurement. Therefore, the specific capacities shown in Fig. R18 were estimated based on the applied current, active material mass, and cycling time. This estimation method does not affect the interpretation of the operando XRD results, as the primary objective of these measurements is to track phase evolution as a function of electrochemical state rather than to provide precise electrochemical performance metrics. This clarification has been added to the revised manuscript.

The operando test was conducted with current 35μA due to limited beamtime. And the capacities of both cells are shown in Figure R18. Besides, we also need to mention that the specific capacity data were calculated roughly based on time, mass and current because the software did not record the corresponding specific capacity during operando test.

And we also added this part in Supplementary information, shown below:

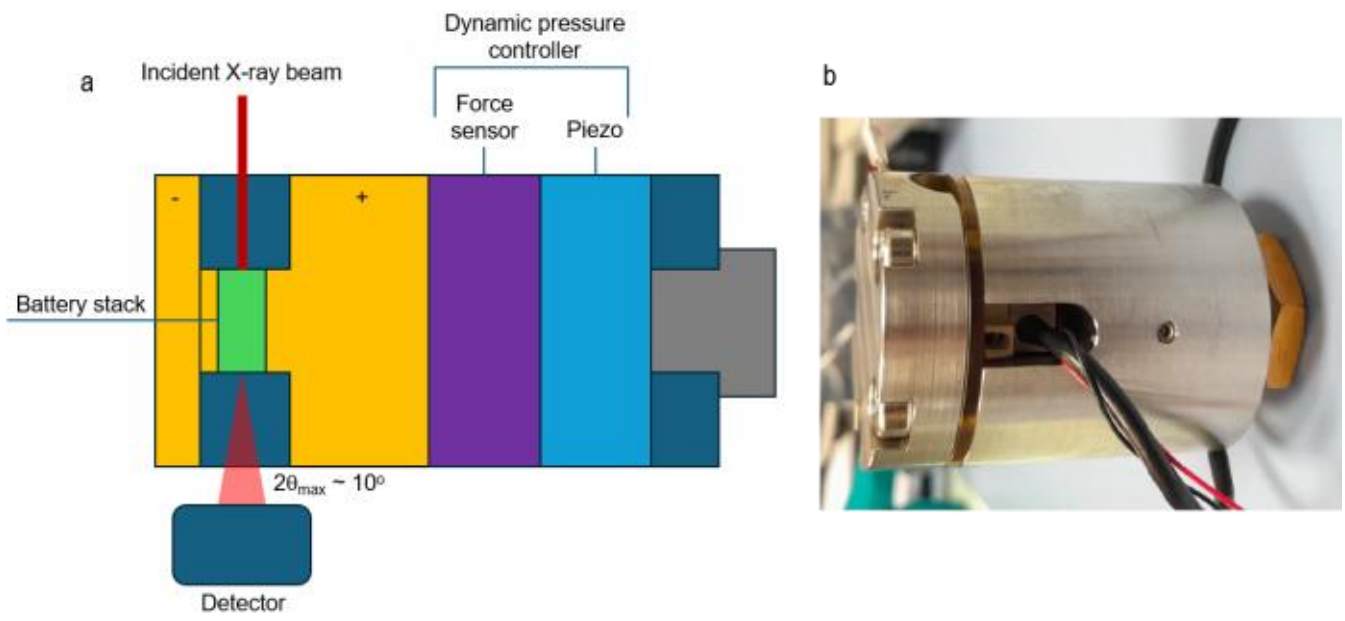

"Figure S26 Operando XRD setup. (a) Schematic of the setup, which contains dynamic pressure control using a piezo. (b) Photo of the actual device

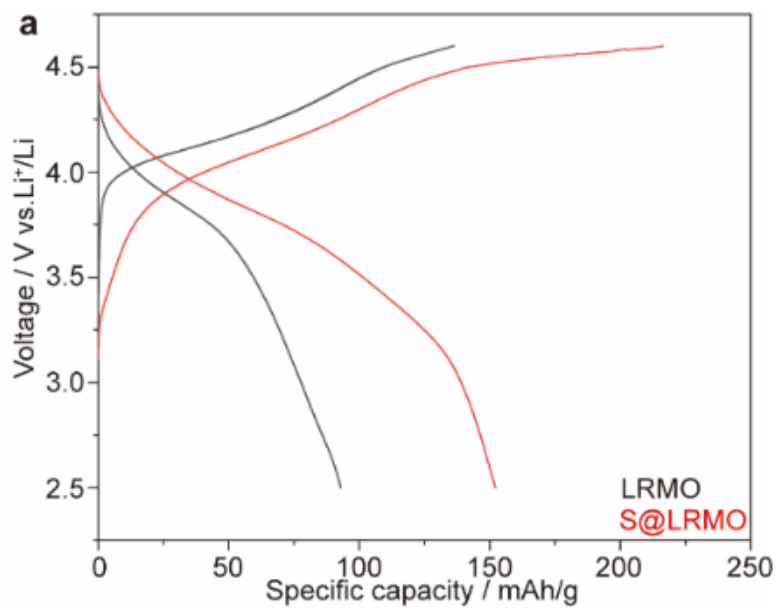

Figure S27 Galvanostatic charge-discharge curves for LRMO and S-LRMO based operando cells in the initial cycle."

3. Are there any errors in Figure 3c? Please check it?

Response: We thank the reviewer for pointing out the inconsistency in Figure 3c. During the initial preparation of the figure, the Li^+ diffusion coefficients for charging and discharging were combined using plotting software, and an inconsistency in the x-axis scaling was inadvertently introduced.

The figure has now been corrected: the charge and discharge data are plotted with consistent x-axis scales, and the revised Figure 3c is shown below. This correction does not affect the interpretation or conclusions of the study. We appreciate the reviewer's careful attention to this detail.

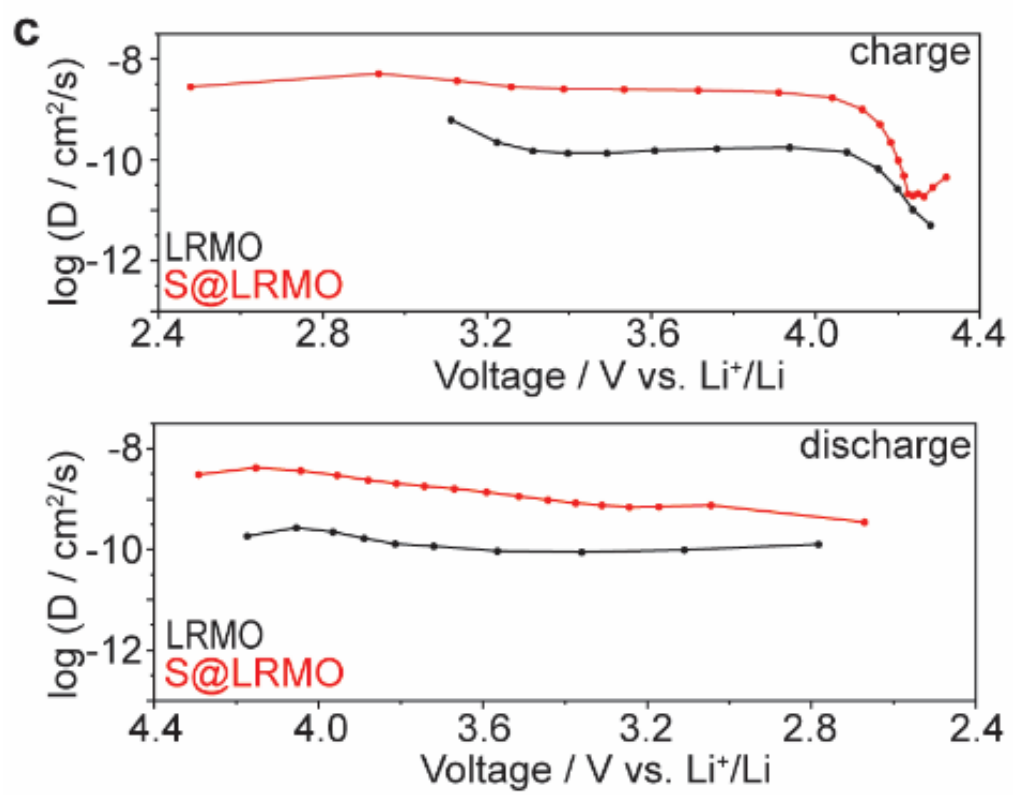

Figure R19 Calculated lithium ion (Li^+) diffusion coefficients obtained via galvanostatic intermittent titration technique (GITT) for LRMO-based and S@LRMO-based cells during charging and discharging.

And we also added this part in Figure 3, shown below:

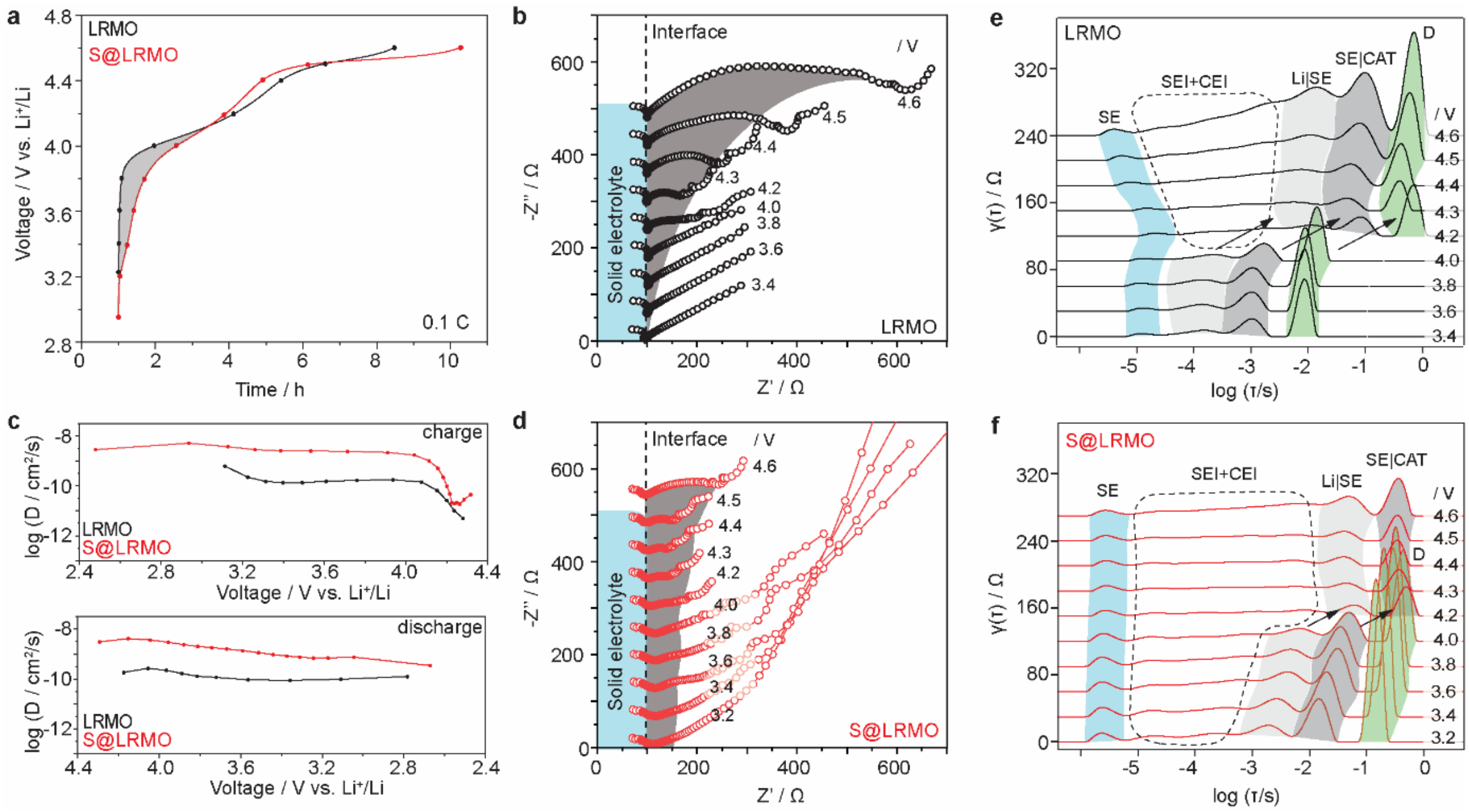

***Figure 3*. Interfacial Li⁺ kinetics evolution between cathode SE during the initial charging process.** *Initial charge voltage curves of the SSB-LRMO and SSB-S@LRMO cells (a); in-situ GEIS spectra collected during initial charging process for SSB-LRMO (b) and SSB-S@LRMO (d) cells and their corresponding DRT profiles (e & f) based on transformation of GEIS results. Calculated lithium ion (Li^+) diffusion coefficients obtained via galvanostatic intermittent titration technique (GITT) for LRMO-based and S@LRMO-based cells during charging and discharging. (g) Solid electrolyte resistance (R_{SE}), (h) interfacial resistance (R_{INTER}) and (i) charge transfer (R_{CT}) resistance changes during initial charging process of SSB-LRMO and SSB-S@LRMO.*

4. The active processes are clearly seen in Figure 2g, please explain it.

Response:

We thank the reviewer for pointing out the activation behavior observed in Fig. 2g. The initial increase in discharge capacity is attributed to progressive improvement and homogenization of the electronic and ionic transport pathways within the cathode composite. During the early cycles, manual mixing of the active material, solid electrolyte, and conductive carbon may result in regions with insufficiently percolated networks, leading to lower utilization of active material, particularly at high current densities.

With repeated charge–discharge cycling, the internal transport network becomes more effective, allowing previously underutilized particles to participate in electrochemical reactions, which manifests as the observed increase in capacity. Such activation phenomena are commonly reported in solid-state batteries, where a fraction of the active material is initially inactive at high C-rates but becomes electrochemically accessible upon cycling [6]. This explanation has been clarified in the revised manuscript.

5. In Figure S2, the Bragg peaks are suggested to be presented. What is the percentage of the monoclinic phase (Li_2MnO_3)? Why are spinel phases not observed in the TEM or HAADF-STEM images?

Response:

Thank you for your questions. For LRMO we used, its formular is $Li_{1.2}Mn_{0.54}Ni_{0.13}Co_{0.13}O_2$ or $0.5Li_2MnO_3 \cdot 0.5LiMO_2$

As for spinel phase, we offer HAADF image shown below. From it, we can clearly see the spinel phases on the surface of the layered LMRO.

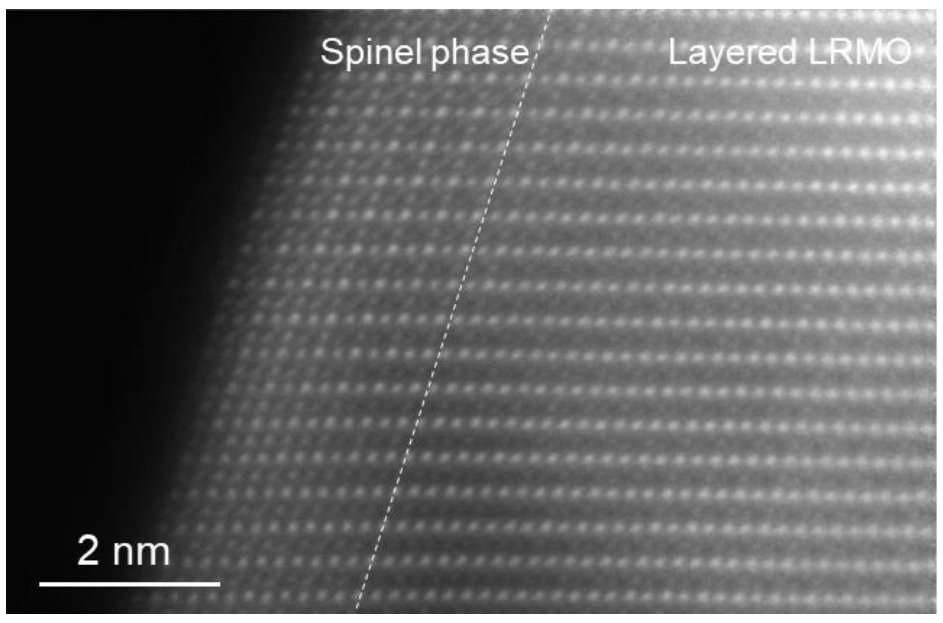

Figure R20. HAADF-TEM image of S@LRMO.

<u>And we added HAADF-TEM image in main discussion in the revised manuscript, as follows:</u>

"

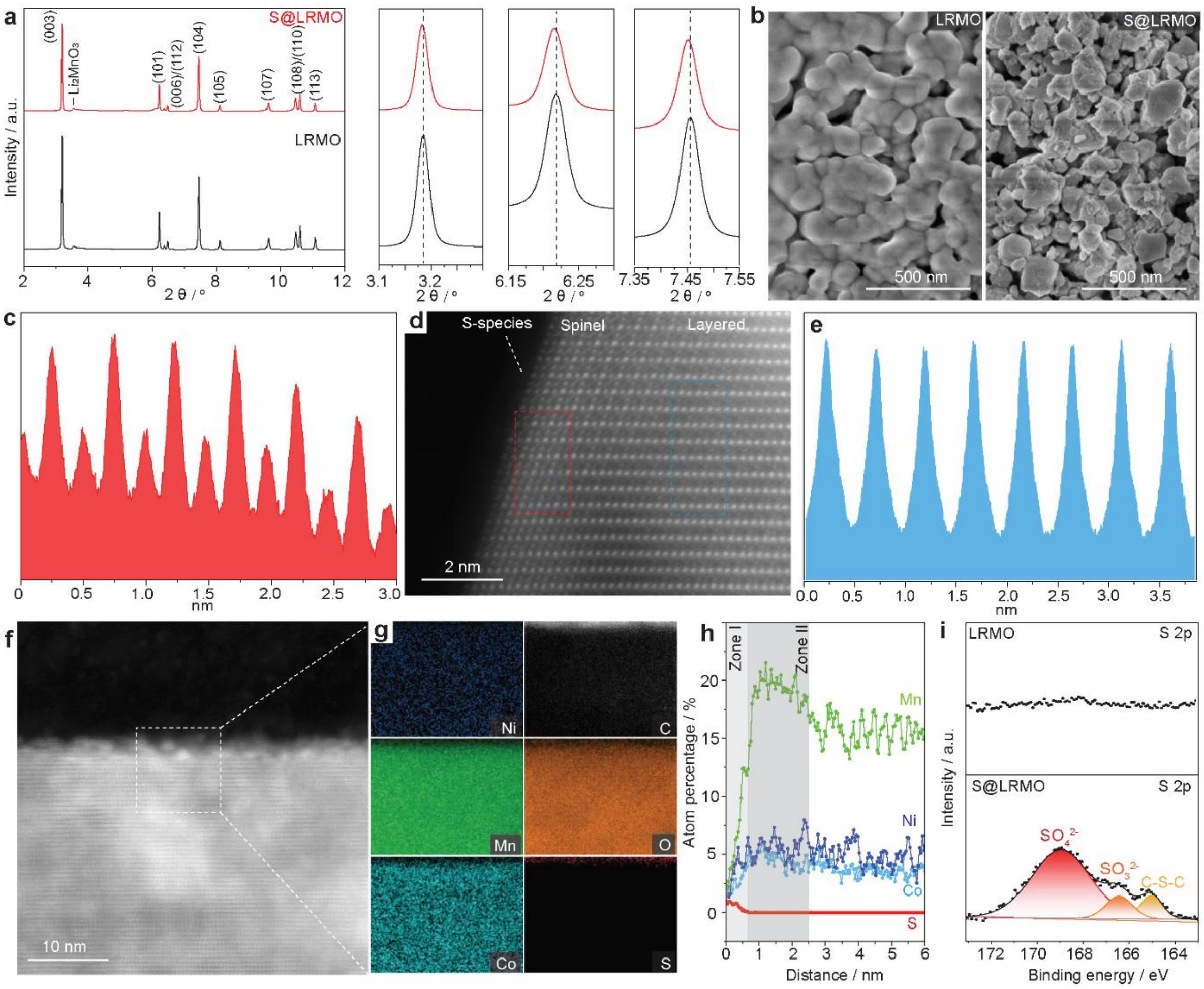

***Figure 1**. **Cathode materials characterizations.** (a) Synchrotron X-Ray Diffraction (SXRD) patterns collected from the bare LRMO and S@LRMO powders; (b) SEM images of bare LRMO (left) and S@LRMO (right) particles; (d) High-Angle Annular Dark-field Scanning Transmission Electron Microscopy (HAADF-STEM) image from a S@LRMO cathode particle and the lattice space (c&e) of selected areas from its surface and bulk. (f) HAADF-STEM image from a S@LRMO cathode particle and its corresponding element maps in (g) extracted from the electron energy loss spectroscopy (EELS) data after mapping the region inside the white dashed box in the STEM image. (h) Element line profiles based on the maps in (g); (i) S 2p XPS spectrum for bare LRMO and S@LRMO particles."*

6. The HAADF-STEM image of LMRO is suggested to be offered.

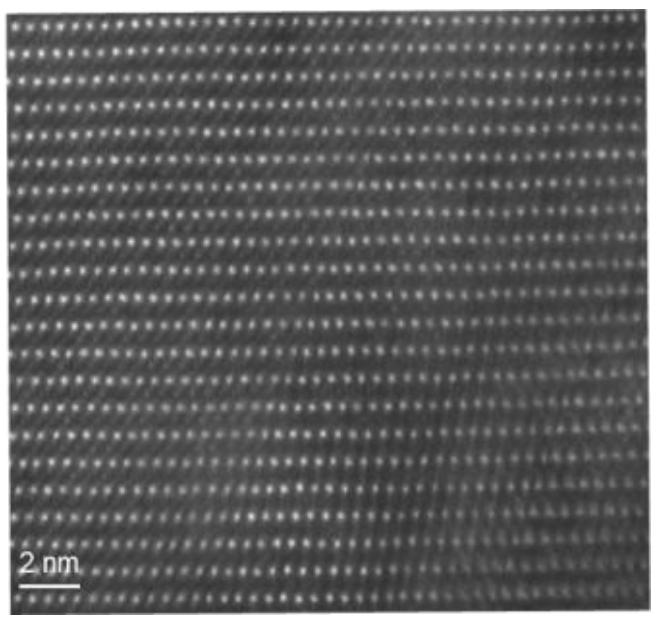

Figure R21 HAADF-STEM image of LRMO.

HAADF-STEM image of LRMO is offered.

And we also added this part in Supplementary information, shown below:

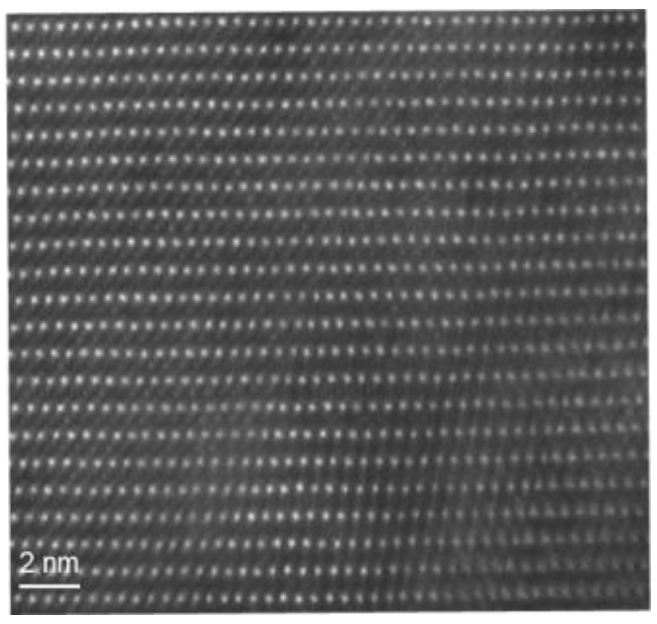

"Figure S7 HAADF-STEM image of LRMO."

7. In Figure S6, why does the peak belonging to -OH increase much after the treatment?

Response:

We thank the reviewer for this question regarding the increase in surface -OH content observed in Fig. S6 after thiourea treatment.

The enhanced -OH signal can be attributed to modifications of the surface chemistry induced by thiourea. First, thermal decomposition of thiourea can generate reducing and mildly alkaline species (e.g., H_2S, NH_3, and S^{2-}), which may partially reduce surface Mn^{4+} to lower-valence states and facilitate the hydroxylation of surface metal-oxygen bonds. Second, sulfur incorporation and/or the formation of surface sulfide or oxysulfide species can create oxygen vacancies, which are highly hydrophilic and tend to absorb hydroxyl groups from the environment.

These surface modifications collectively contribute to the observed increase in the -OH peak intensity after treatment, consistent with the known chemistry of thiourea-treated transition-metal oxides.

8. In Figure S11, the midpoint voltage is suggested to be replaced by the average. In Figure S11, the midpoint voltage is suggested to be replaced by the average voltage, and the charge discharge curves for the 4th, 100th, 200th, 300th, and 500th cycles are suggested to be presented.

Response:

Thank you for your suggestions. We use the average voltage (shown in Fig. R21) to replace the previous midpoint voltage.

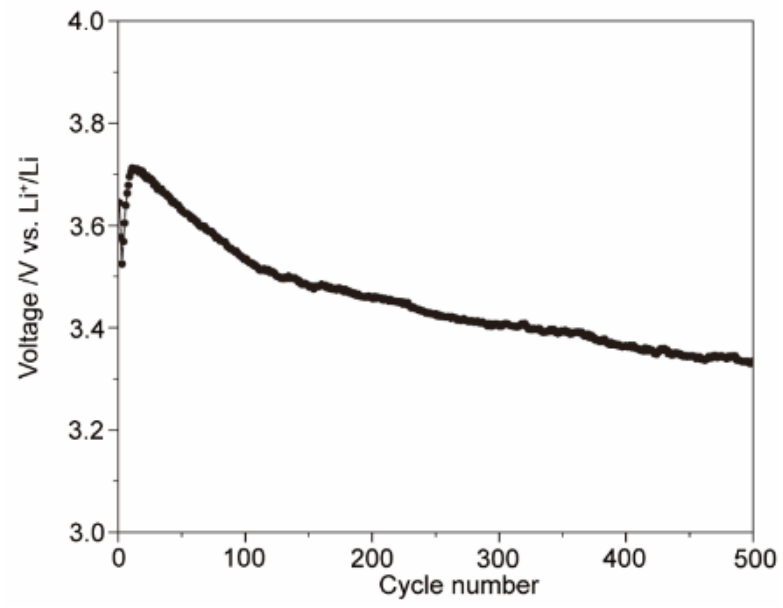

Figure R22 Curves for average voltage changes for S@LRMO-based cell during 1 C long-term cycling.

And for the charge and discharge curves of 4 th, 100th, 200th, 300th, and 500th cycles are plotted as well, shown in Fig

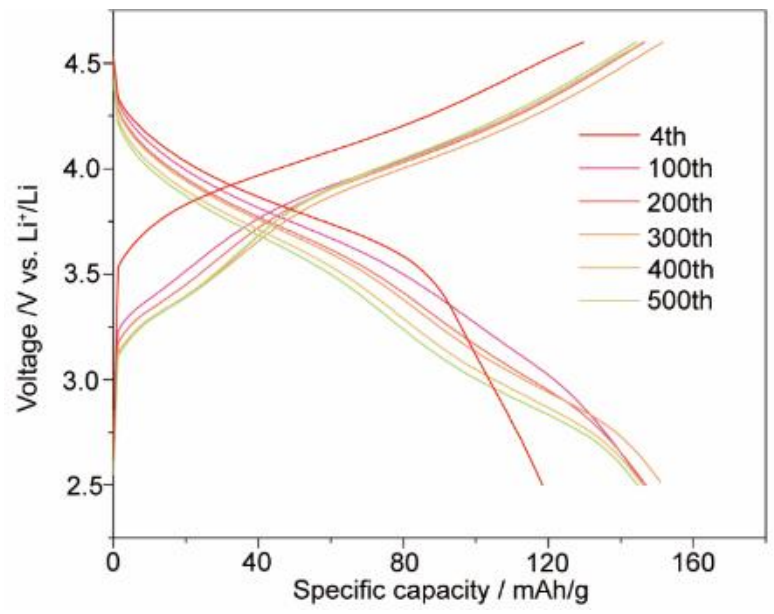

Figure R23 Charge/discharge of different cycles for SSB-S@LRMO with current density of 1C.

And we also added these two graphs in Figure S19 and Figure S18, shown below:

"

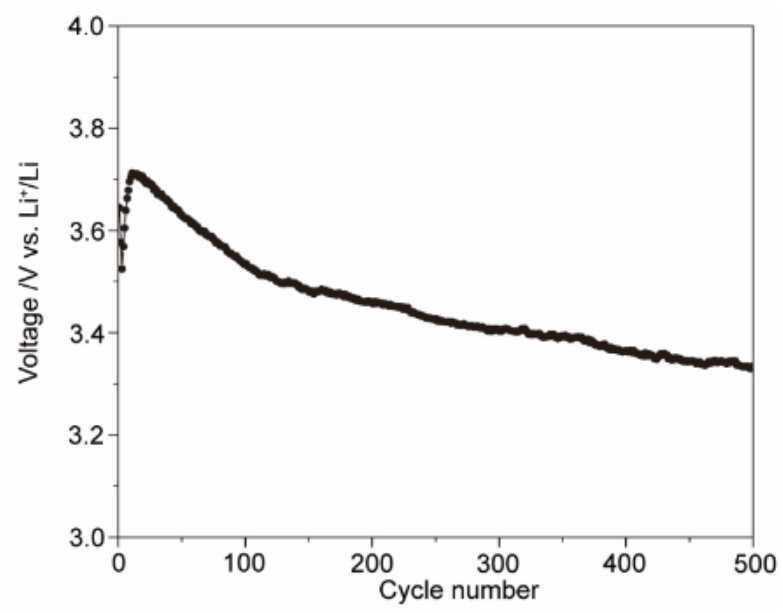

Figure S19. Curves for average voltage changes for S@LRMO-based cell during 1 C long-term cycling.

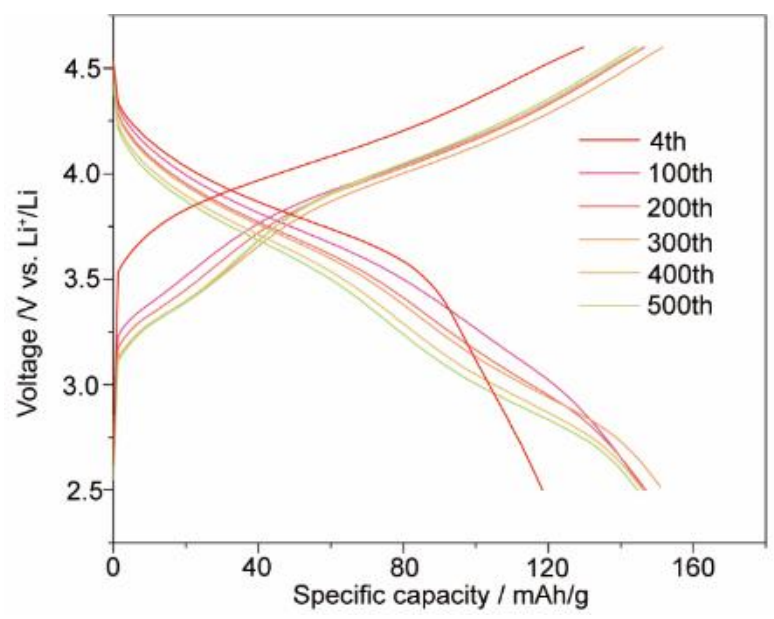

Figure S20. Charge/discharge of different cycles for SSB-S@LRMO with current density of 1C.

"

9. In Figure S12, is the relaxation time of 1h during the GITT test not enough to get a quasi-equilibrium state?

Response:

We thank the reviewer for raising this important point regarding the relaxation time in the GITT measurements. In GITT, the relaxation period is intended to allow the cell to approach a quasi-equilibrium potential, where the measured voltage primarily reflects thermodynamics rather than kinetic polarization.

In our initial measurements, a 1 h relaxation was adopted based on standard protocols for liquid-electrolyte LRMO cells. However, solid-state cells exhibit slower ionic transport and more pronounced solid-solid interfacial kinetics, which can necessitate longer relaxation times.

To evaluate this effect, we conducted additional GITT experiments with extended relaxation. As shown in Fig. R23, a 2 h relaxation period allowed the cell to reach a quasi-equilibrium state. Quantitative comparison of the voltage at three time points—end of the current pulse (A), after 1 h relaxation (B), and after 2 h relaxation (C)—indicates that approximately 90.2% of the total voltage change occurred during the first hour, with only ~9.8% remaining in the subsequent hour. This suggests that a 1 h relaxation captures the majority of the relaxation process, although longer relaxation times can further improve accuracy. We have clarified this point in the revised manuscript and will consider longer relaxation periods in future studies for solid-state systems.

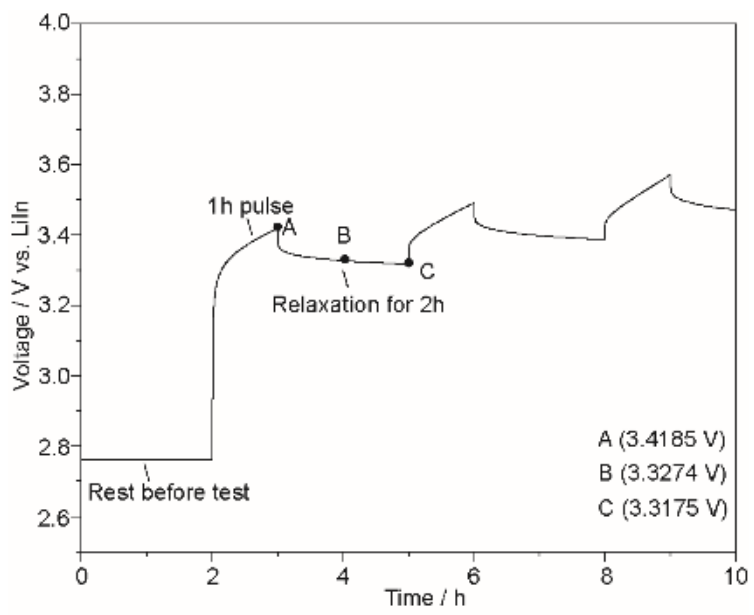

Figure R24 Charge/discharge of different cycles for SSB-S@LRMO with current density of 1C.

10. Why the SEI/CEI resistance (R_{SEI}) and charge transfer (R_{CT}) resistance of SSB-S@LRMO decrease in the high voltage, it is unnormal. Please explain it.

Response: We thank the reviewer for this insightful question regarding the decrease of SEI/CEI resistance (R_SEI) and charge-transfer resistance (R_CT) at high voltage in SSB-S@LRMO cells.

In SSB-LRMO cells, oxygen release and transition-metal migration at high voltage typically lead to growth of interfacial resistance. In contrast, the S@LRMO cathode maintains a chemically cleaner and structurally more ordered interface. The sulfur-derived coating suppresses oxygen evolution and promotes the formation of a stable, thin, and ionically conductive interphase. Moreover, it buffers lattice rearrangements associated with oxygen redox activation, which facilitates Li^+ transport across the interface. These combined effects likely contribute to the observed decrease in both R_SEI and R_CT at elevated voltages.

This interpretation is consistent with previous observations that surface coatings can stabilize the cathode-electrolyte interface and enhance ionic transport during high-voltage cycling in solid-state batteries.

Reviewer #4 (Remarks to the Author):

This work presents a thiourea-derived surface modification strategy for LRMO cathodes, aiming to create a sulfur-rich sub-nanometer coating and induce spinel-like surface reconstruction to enhance solid-state battery performance. The work failed to meet the requirements of this journal for innovation and mechanism depth. The specific reasons are as follows:

1. **The proposed sulfur-rich coating modification for LRMO cathodes lacks sufficient novelty, as similar approaches utilizing sulfate/sulfite-based coatings (e.g., SO_4^{2-}/SO_3^{2-}) to stabilize the interface, suppress oxygen loss/TM migration, and enhance Li^+ transport have been previously demonstrated by Zhang et al. (Sci. Adv. 2022, DOI: 10.1126/sciadv.add5189). While the authors employ thiourea-derived coatings in this work, the fundamental chemistry and functional mechanisms appear analogous to prior article, significantly diminishing the conceptual advancement. To justify publication, the authors must explicitly clarify how their method differs in (1) coating composition or formation mechanism, (2) interfacial stabilization efficacy, or (3) electrochemical performance metrics compared to existing strategies. Without such comparative analysis, the study's incremental contribution remains unclear.**

Response:

Thank you for your valuable comments. We carefully read the paper you mentioned and appreciate the excellent work presented. However, we respectfully disagree that the fundamental chemistry and functional mechanisms in our work are similar to that publication. To clarify, we provide a detailed comparison below.

(i) Coating composition and mechanism: we summarize the differences between Li_2SiO_4-based coatingsand our thiourea-derived coating in Table R3.

Table R3 Comparison on coating details and mechanism of Li_2SiO_4 and $SC(NH_2)_2$ for LRMO cathode.

	Ref	This work
Coating methods	Ball-milling	Liquid phase coating
Precursor	Li_2SO_4	$SC(NH_2)_2$
Coating composition	SO_4^{2-} , SO_3^{2-}	SO_4^{2-} , SO_3^{2-} , spinel-type $LiMn_2O_4$
Coating formation	Acquired from ball milling and annealing.	Phase transition (forming spinel phase at surface) based on thiourea
Working principles	Suppressing O-release through reversible reactions between SO_4^{2-} , SO_3^{2-}	Constructing a stable CAT | SE interface based on S-species coating layer and subsurface spinel-type $LiMn_2O_4$

As shown, while both coatings contain sulfate/sulfite species, our method introduces a **surface spinel-type $LiMn_2O_4$ phase** formed in situ from thiourea, which is absent in the Li_2SiO_4-based system. This results in a fundamentally different mechanism of interfacial stabilization.

To uncover the fundamental chemistry of coating formation, we propose that the formation of sulfur-containing species and the spinel phase proceeds via the following reactions. When LRMO particles with an alkaline surface are introduced into the solvent, thiourea decomposes according to Reaction (1):

$$SC(NH_2)_2 + 2OH^- \rightarrow S^{2-} + CO(NH_2)_2 + H_2O \quad (1)$$

Subsequently, the generated $CO(NH_2)_2$ further decomposes to produce NH_3, as described by Reaction (2). The absence of a discernible N 1s signal in the XPS spectra (Fig. S13) is consistent with the volatilization of nitrogen-containing species during this process:

$$2CO(NH_2)_2 \rightarrow NH_3 + 2HCNO \quad (2)$$

During the annealing step, the produced S^{2-} species and/or NH_3 act as reducing agents, promoting

the partial reduction of Li_2MnO_3 and inducing its transformation into the spinel $LiMn_2O_4$ phase. Concurrently, S^{2-} is oxidized to sulfite and sulfate species, as summarized in Reaction (3):

$$O_2 + S^{2-} + Li_2MnO_3 \rightarrow SO_3^{2-} + SO_4^{2-} + LiMn_2O_4 \quad (3)$$

(ii) Electrochemical performance: A direct comparison of electrochemical metrics is presented in both Figure 2 (main text) and Table S4 (Supporting Information). For clarity, we also provide Table R4:

Table R4. Electrochemical performances of all solid-state batteries based on Li_2SiO_4@LRMO and $SC(NH_2)_2$@LRMO

	Li_2SiO_4@LRMO	$SC(NH_2)_2$@LRMO
Mass loading	4.2 mg/cm^2	7.13 mg/cm^2
Initial discharge specific capacity	248 mAh/g	220 mAh/g
Initial coulombic efficiency	94 %	84.83 %
Discharge specific capacity at 1C	130 mAh/g	153 mAh/g
Capacity retention at 1C	81.2% after 300 cycles	>97 % after 600 cycles

These results demonstrate that our $SC(NH_2)_2$-derived coating enables **higher rate capability and superior cycling stability**, despite the higher mass loading.

(iii) **Clarification of abbreviations**

We note that some abbreviations overlap with those in the Sci. Adv. paper, which may have caused confusion:

- ✧ **S@LRMO:** In our manuscript, S@LRMO refers to the thiourea-derived coating, comprising both S-species (TM sulfates and sulfites) and the spinel phase formed on the LRMO surface. In contrast, in the Sci. Adv. publication, S@LRMO refers solely to Li_2SO_4-coated LRMO.
- ✧ **LICF:** While both works use LICF to denote $Li_3InCl_{6-x}F_x$, the composition differs: x = 0.6 in our manuscript versus x = 1.2 in the Sci. Adv. paper. We have another manuscript focused specifically on Li_3InCl_6–xF_x that will be submitted shortly.

We apologize for any confusion caused by the overlapping abbreviations. Importantly, these distinctions in composition, formation mechanism, and electrochemical performance **clearly demonstrate the novelty and incremental advancement of our work** relative to previous studies.

To make it clear, we integrated this part into main text and Supplementary information in the revised manuscript and revised Supplementary information, as follows:

"Notably, TM ion signals exhibit shifts toward higher oxidation states, likely resulting from oxidation during the annealing process.

Based on the above results and analyses, we propose that the formation of sulfur-containing species and the spinel phase proceeds via the following reactions. When LRMO particles with an alkaline surface are introduced into the solvent, thiourea decomposes according to Reaction (1):

$$SC(NH_2)_2 + 2OH^- \rightarrow S^{2-} + CO(NH_2)_2 + H_2O \quad (1)$$

Subsequently, the generated $CO(NH_2)_2$ further decomposes to produce NH_3, as described by Reaction (2). The absence of a discernible N 1s signal in the XPS spectra (Fig. S13) is consistent with the volatilization of nitrogen-containing species during this process:

$$2CO(NH_2)_2 \rightarrow NH_3 + 2HCNO \quad (2)$$

During the annealing step, the produced S^{2-} species and/or NH_3 act as reducing agents, promoting the partial reduction of Li_2MnO_3 and inducing its transformation into the spinel $LiMn_2O_4$ phase. Concurrently, S^{2-} is oxidized to sulfite and sulfate species, as summarized in Reaction (3):

$$O_2 + S^{2-} + Li_2MnO_3 \rightarrow SO_3^{2-} + SO_4^{2-} + LiMn_2O_4 \quad (3)"$$

2. The manuscript presents a surface coating modification for cathode materials but fails to provide systematic experimental evidence demonstrating how the optimal coating content was determined. To establish scientific rigor and reproducibility, the authors should include: (1) electrochemical performance comparisons (charge/discharge curves, long-term cycling stability, and rate capability tests) across different coating contents (e.g., 1%, 3%, 5%), clearly illustrating the impact on capacity, voltage stability, and kinetics; (2) microstructural characterization (TEM images with thickness measurements and EDS/elemental mapping) to verify coating uniformity and layer integrity at varying coating ratios. Without these critical datasets, the justification for the selected coating content remains unsubstantiated, significantly limiting the study's practical applicability. Providing this information would strengthen the mechanistic understanding and enable readers to evaluate the optimization process objectively.

Response:

Thank you for your insightful comments regarding the determination of the optimal coating content. To address this concern, we conducted systematic studies on LRMO cathodes coated with thiourea at different contents (1%, 2%, and 3%) and evaluated both their electrochemical performance and microstructural features.

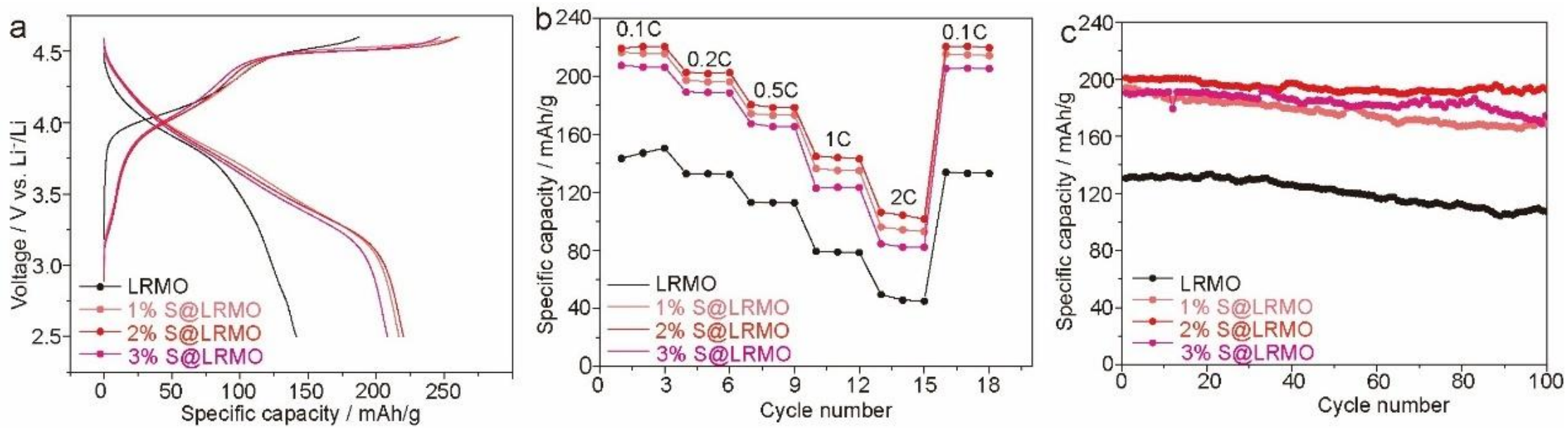

Figure **R25**. Electrochemical performances of LRMO and coated LRMO with various coating contents (1%, 2% and 3%). (a) charge and discharge curves of initial cycle; rate capabilities (b) and long-term cycling stabilities (c).

As shown in Figure R25, the initial charge/discharge curves (Figure R25a), rate capability tests (Figure R25b), and long-term cycling stability (Figure R25c) indicate that 2% thiourea-coated LRMO exhibits the best overall performance. Specifically, 2% S@LRMO provides a balance of high specific capacity, stable voltage profile, and superior cycling retention. In contrast, the 3% sample shows a slightly reduced capacity, likely due to the formation of a thicker coating layer that slightly hinders Li^+ transport. These results support the selection of 2% thiourea as the optimized coating content.

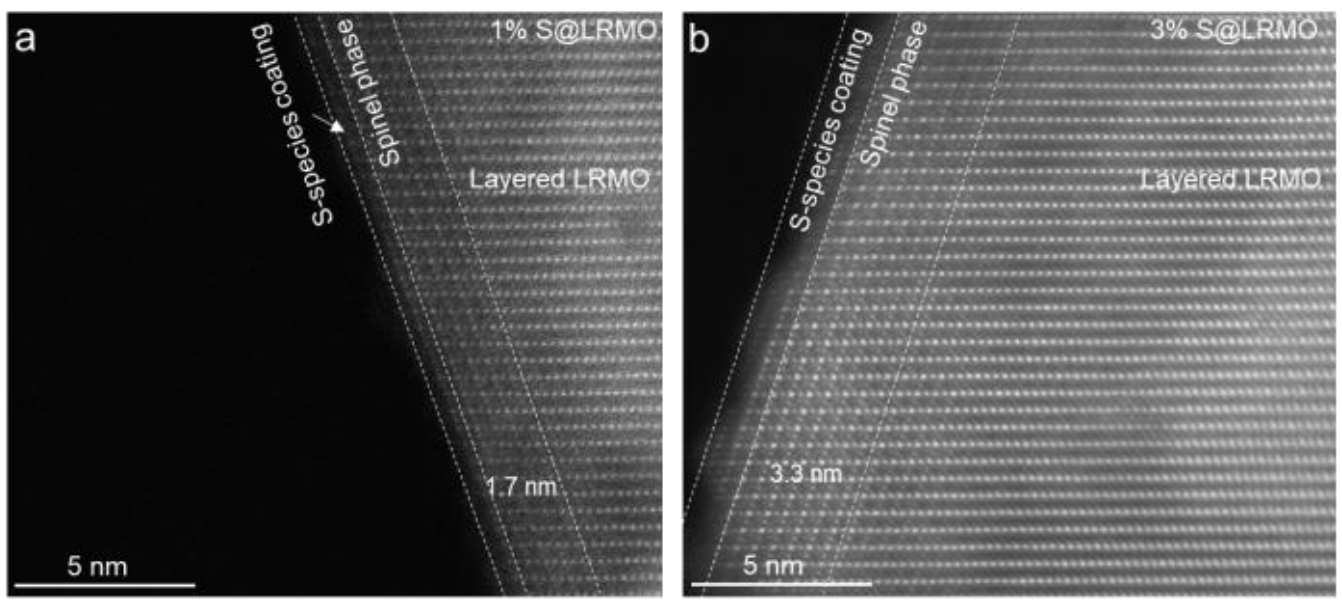

Figure R26. TEM and HAADF-TEM images of coated LRMO with various coating contents.

HAADF-TEM analyses of 1% and 3% S@LRMO (Figure R26) confirm full surface coverage for both coating levels. The measured thickness of the spinel-type surface layer is approximately **1.7 nm** for 1%

S@LRMO and **3.3 nm** for 3% S@LRMO. The thickness varies slightly across particles, indicating conformal but nanoscale heterogeneous deposition.

Due to issues with EELS measurements, SEM-EDS mapping was also performed. Both 1% and 3% S@LRMO samples exhibit clear sulfur signals on the LRMO surface, confirming the presence of S-containing species and successful surface coating.

These systematic electrochemical and structural analyses demonstrate that 2% thiourea provides an optimal coating, achieving sufficient interfacial protection while minimizing impedance to Li^{+} transport. The data provide a reproducible and mechanistically supported basis for coating optimization.

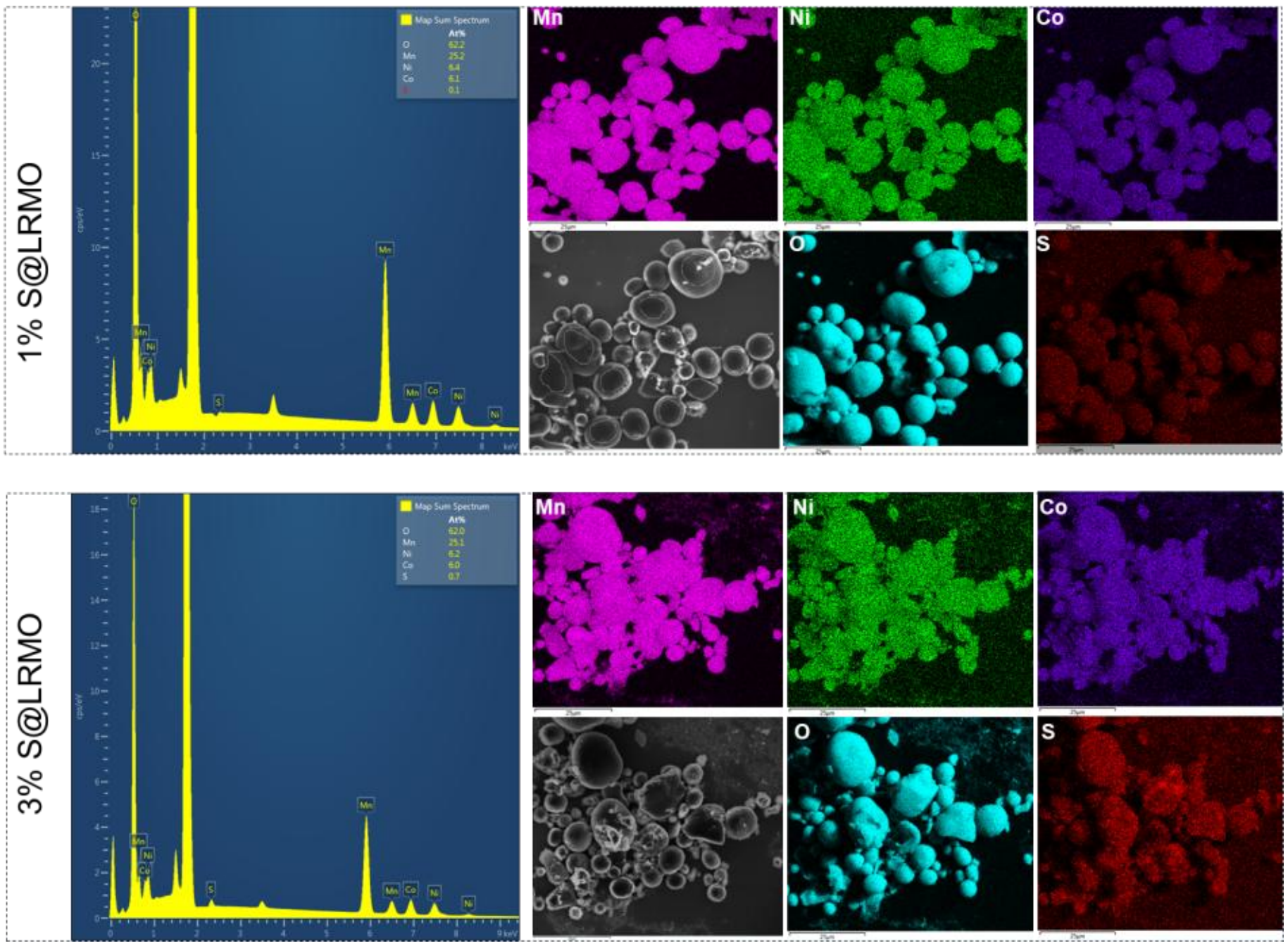

Figure R27. EDS mapping of coated LRMO with various coating contents (1% and 3%)

<u>And we also added these TEM images and cell performances in Supplementary information, shown below:</u>

"

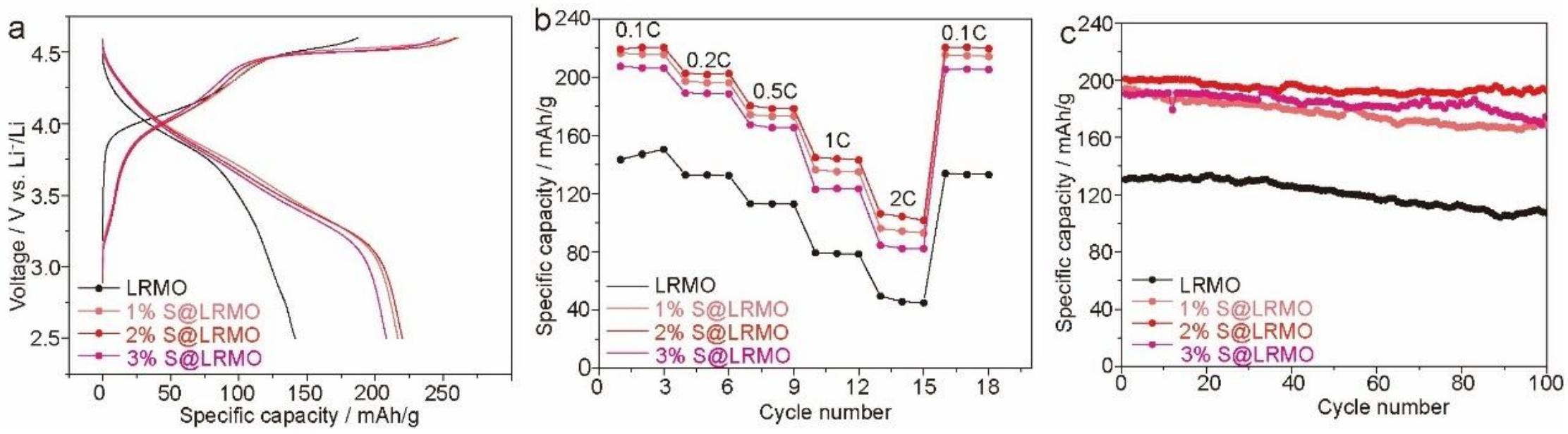

Figure S18. Electrochemical performances of LRMO and coated LRMO with various coating contents (1%, 2% and 3%). (a) charge and discharge curves of initial cycle; rate capabilities (b) and long-term cycling stabilities (c).

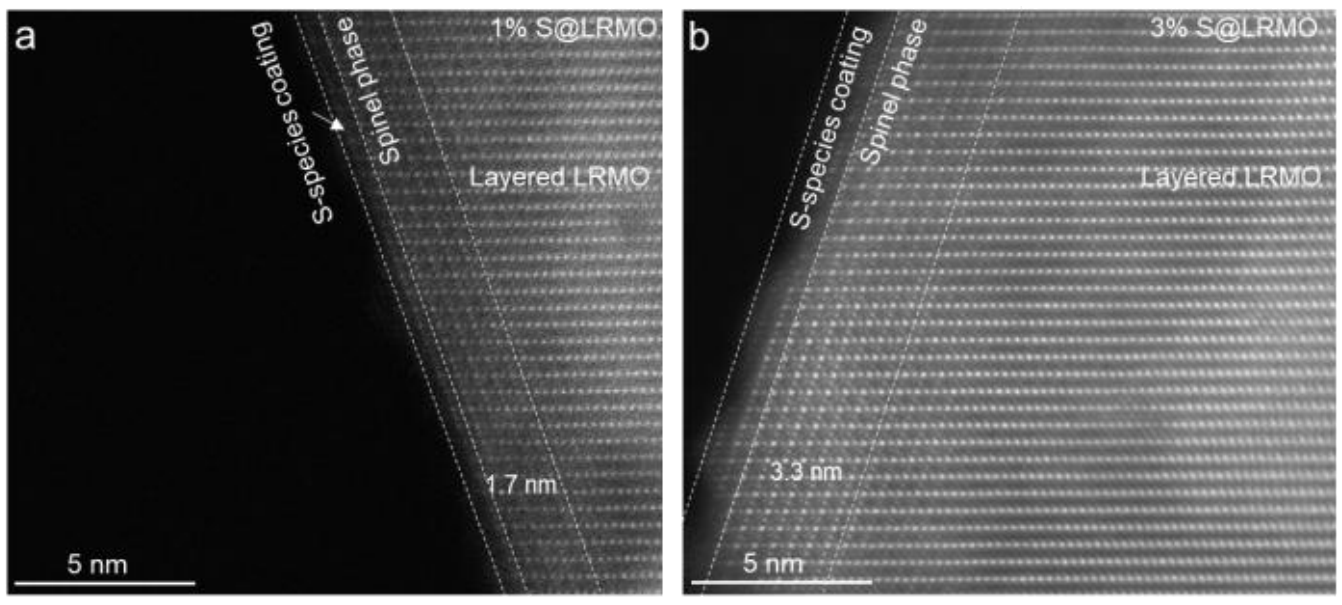

Figure S8. HAADF-STEM image of 1% S@LRMO (a) and 3% S@LRMO (b).
"

3. The authors' claim of achieving a 0.7-nm-thick uniform coating via conventional liquid-phase methods raises significant concerns regarding both methodological feasibility and experimental validation. At this thickness (equivalent to merely 2-3 atomic layers), the coating approaches single-atom precision - a feat typically only achievable through atomic layer deposition (ALD) techniques, as extensively documented in the literature. For liquid-phase approaches like sol-gel or coprecipitation, such extreme thinness would be exceptionally vulnerable to nanoparticle agglomeration and heterogeneous growth. To substantiate this extraordinary claim, the authors must: (1) provide comprehensive full-particle HRTEM images demonstrating cross-scale uniformity, supplemented by statistically rigorous thickness measurements (≥3 independent points per coating condition); (2) offer a detailed mechanistic explanation of how their liquid-phase process overcomes the fundamental limitations of nanoparticle self-assembly; and (3) include post-cycling TEM or TOF-SIMS analysis to verify whether such an ultrathin coating maintains structural integrity under electrochemical cycling, particularly at high-curvature surface features where coverage would be most challenging. Without this critical evidence, the reported coating thickness and its purported uniformity remain scientifically unsubstantiated.

Response: Thank you for your insightful comments regarding the claimed ultrathin coating. We fully agree that achieving a ~0.7 nm coating via conventional liquid-phase methods is challenging. Nevertheless, ultrathin coatings on LRMO using liquid-phase approaches have been reported in previous studies[7].

In our work, thiourea was used as the precursor for the coating layer. During the initial liquid-phase coating, a relatively thicker layer is formed. Upon subsequent annealing and surface reactions, part of this layer reacts with the outermost atomic layers of LRMO, forming a spinel-type phase, while the evaporation of NH_3 and other N-containing gases reduces the overall coating thickness. A schematic illustration of this process is provided in Figure R28.

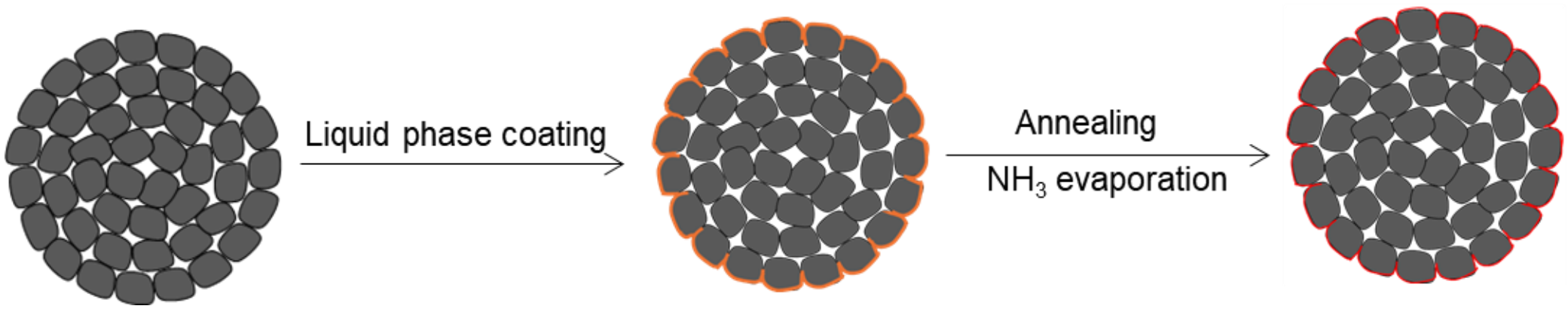

Figure R28. Schematic illustration of coating process.

Interestingly, we observed partial fracture of the pristine secondary LRMO particles during the coating process, leading to smaller particles with increased surface area. As a result, the 2% thiourea

coating formed a thinner and more uniform layer on these smaller particles (Figure R29).

To address the specific points raised:

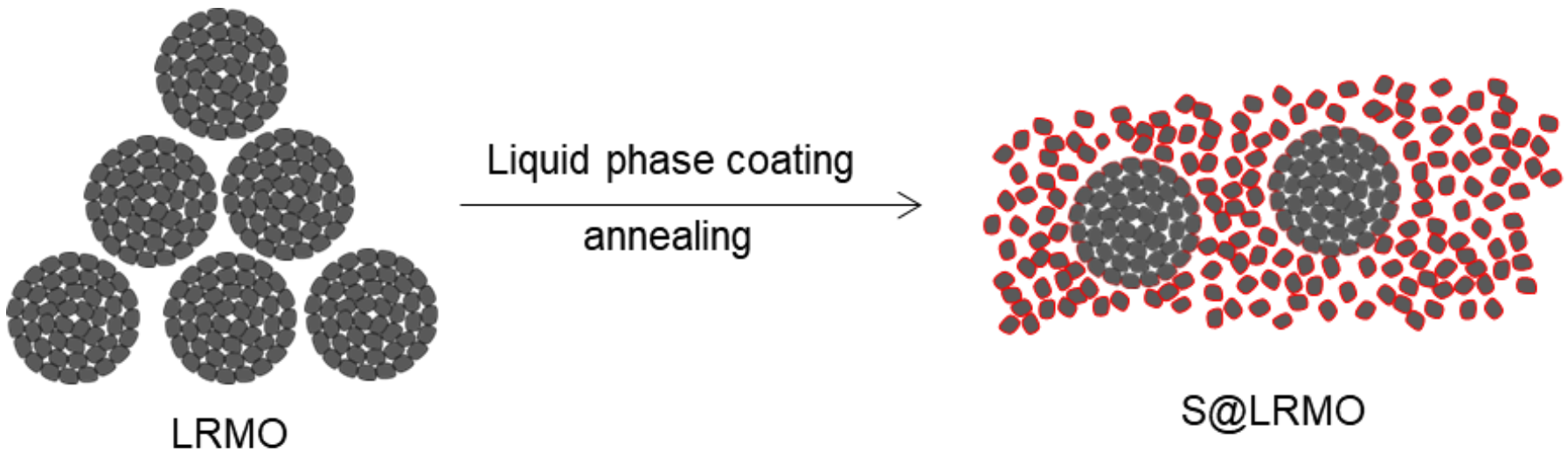

Figure R29. Schematic illustration of secondary particles breaking partially.

(i) Cross-scale TEM evidence: Additional TEM images from three independent particles confirm that the ultrathin coating uniformly covers the LRMO surfaces (Figure R30)

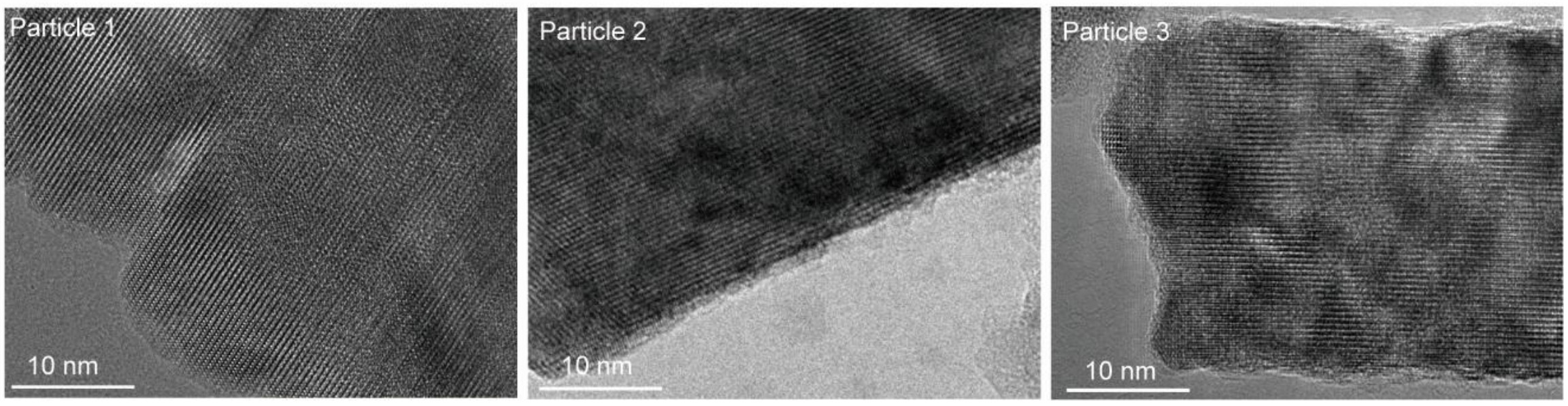

Figure R30. TEM images of 3 independent areas.

(ii) Mitigation of particle agglomeration: The LRMO particles employed are micrometer-sized, minimizing the risk of agglomeration during the liquid-phase coating process. SEM images of a representative secondary particle are shown in Figure R31.

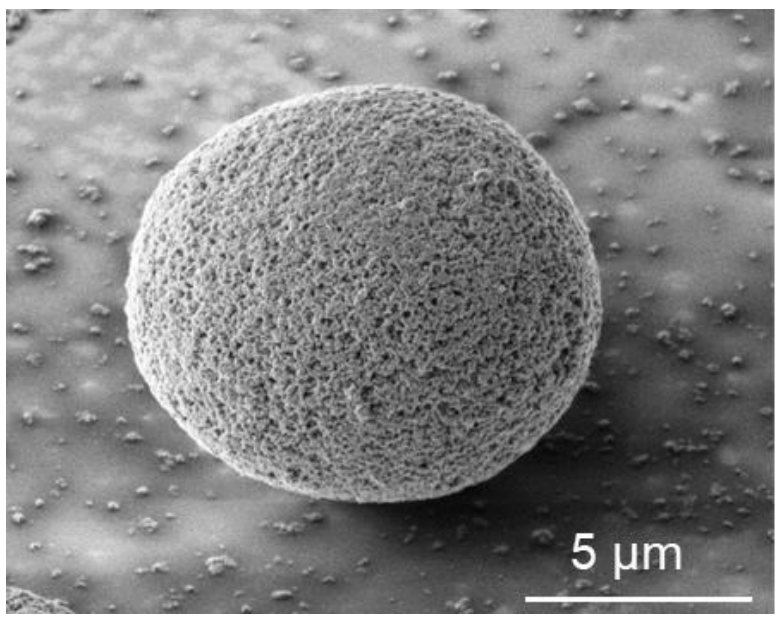

Figure R31. SEM image of a single LRMO secondary particle.

(iii) Structural stability under cycling: Post-cycling TEM analysis demonstrates that the prefabricated spinel phase remains intact on the LRMO particle surface (Figure R32a). Furthermore, XPS measurements of the cycled S@LRMO reveal an S 2p peak at ~169 eV corresponding to SO_4^{2-}, confirming that the coating maintains its chemical and structural integrity during electrochemical

cycling (Figure R32b).

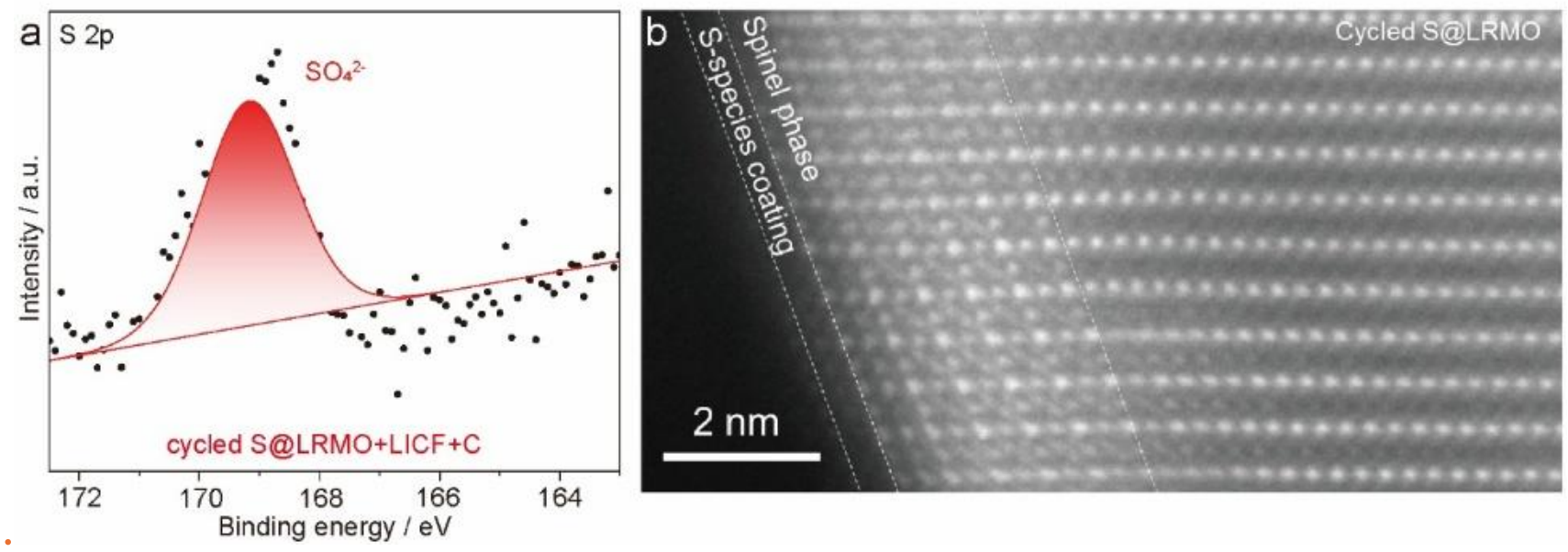

Figure R32. S 2p XPS spectrum of cycled S@LRMO cathode components (a) and TEM image of cycled S@LRMO cathode particle (b).

These results provide robust experimental evidence that the ultrathin (~0.7 nm) thiourea-derived coating is both feasible via liquid-phase processing and structurally stable during cycling, addressing concerns regarding methodological plausibility and coating uniformity.

And we also integrated some of these graphs into Supplementary information, as follows:

"

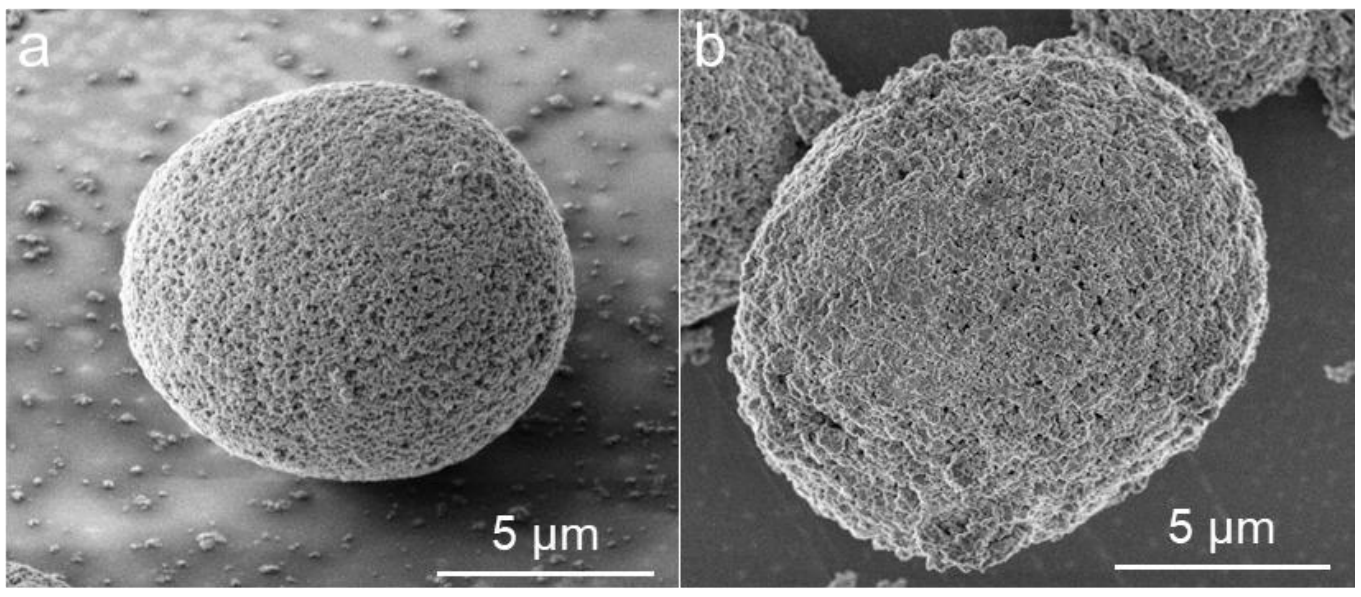

Figure S3. SEM image of LRMO (a) and S@LRMO (b).

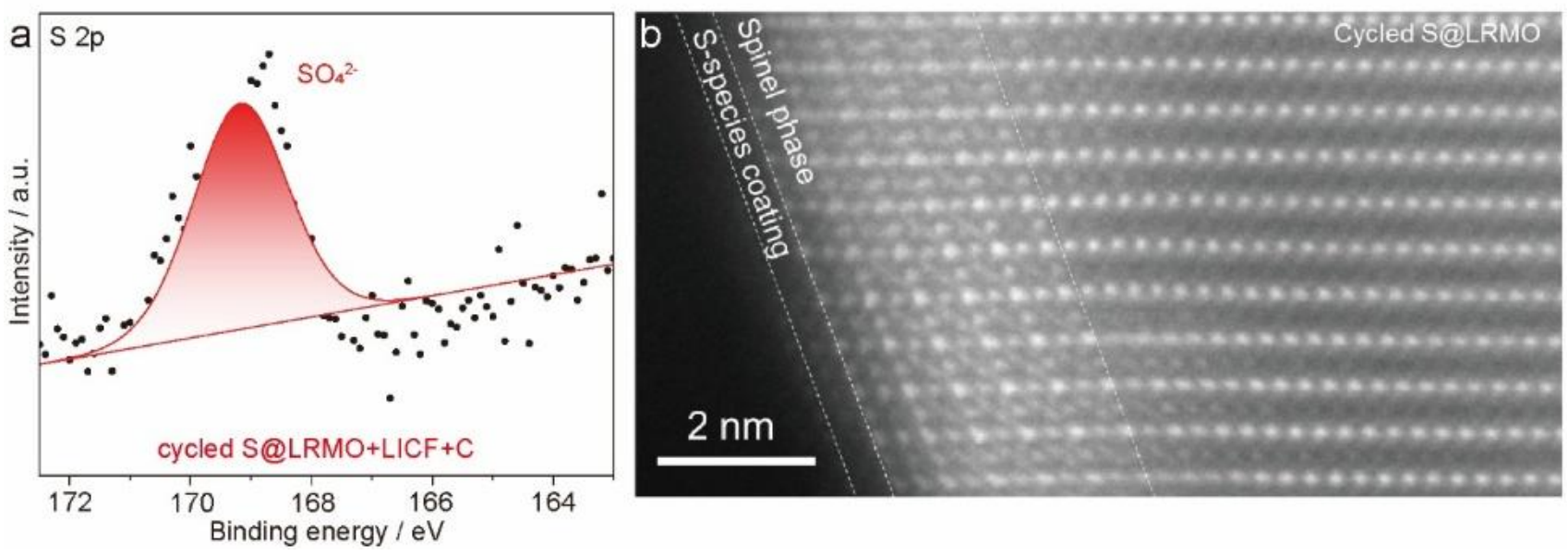

Figure S29. S 2p XPS spectrum of cycled S@LRMO cathode components (a) and TEM image of cycled S@LRMO cathode particle (b).

And we add more text related to these two graphs in the main text:

*"**Materials synthesis***

2 g commercial LRMO (Ningbo Lanli Energy Technology Co., Ltd.) was dispersed in Thiourea-containing deionized water (0.04 g in 30 mL)."

*"**Coating effectiveness during long-term cycling.** To verify the persistence of the sulfur-containing surface coating and the spinel phase on LRMO particles after prolonged cycling, post-mortem analysis based on XPS and TEM was conducted for S@LRMO cathode. XPS measurements detect sulfur-containing species on the cycled electrodes, with binding energies consistent with SO_4^{2-}(Fig. S29a). This observation is further corroborated by TEM analysis with spinel remaining on the surface of*

LRMO particle (Fig. S29b). Collectively, these results demonstrate that the ultrathin coating and surface spinel phase maintained their structural integrity during long-term electrochemical cycling.”

4. The TEM data presented in this study is insufficient to support the structural analysis conclusions. Specifically: (1) The HRTEM images lack critical lattice spacing measurements (e.g., the (003) plane of layered structures), which are essential for direct verification of the crystal structure; (2) The SAED patterns are not indexed with zone axes or diffraction spot assignments, making accurate phase identification impossible; and (3) No comparative HRTEM analysis of cycled vs. uncycled samples is provided to demonstrate the coating's role in suppressing phase transitions. To address these deficiencies, the authors must: (i) clearly label key lattice spacings (e.g., (003), (104), and any spinel/rock-salt phase signatures) in HRTEM images; (ii) index all SAED patterns with zone axes and diffraction spots, including calibration references; and (iii) perform detailed structural evolution analysis of pre- and post-cycling samples using FFT or multi-area SAED to quantify phase transition extent. These supplemental data are crucial for validating the coating's effectiveness, as the current conclusions lack direct structural evidence at the atomic scale. Without such fundamental characterization, the mechanistic claims remain unsubstantiated.

Response:

Thank you for your respond: we have carefully addressed each point as follows:

(i) **Lattice spacing measurements**: HAADF-TEM images of S@LRMO clearly reveal the spinel-phase coating on the layered LRMO surface. Lattice spacings for both the spinel coating and the underlying layered LRMO structure have been measured and are provided in Figure R33. These measurements directly support the coexistence of the layered and spinel phases at the atomic scale.

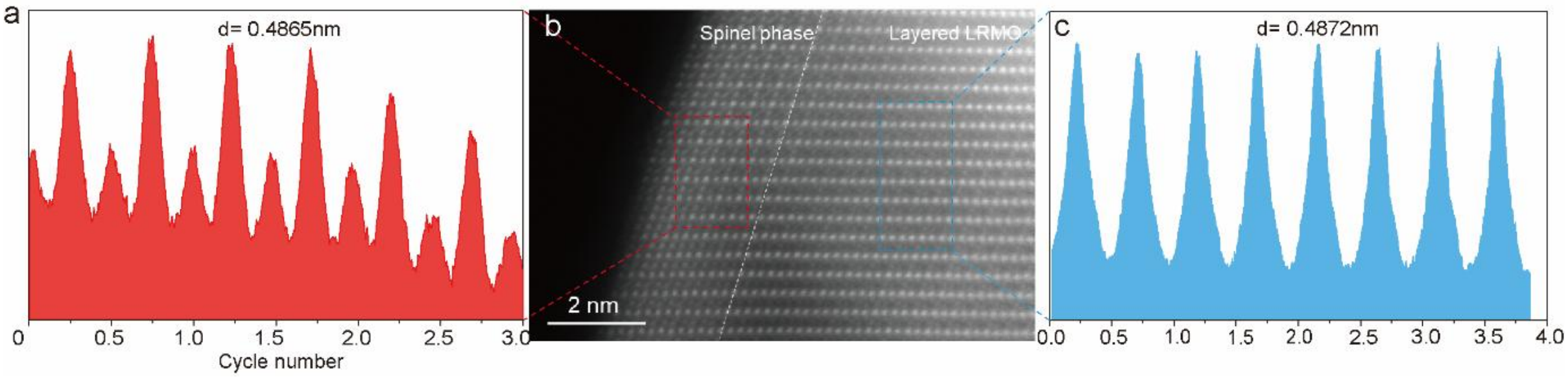

Figure R33. HAADF-TEM image of S@LRMO and corresponding lattice space of both layered structure and spinel phase.

(ii) **SAED indexing and phase identification**: we acknowledge that the SAED patterns in the original manuscript (Figure 1) were not indexed with zone axes or diffraction spot assignments, which limited phase identification. To address this, representative regions from TEM images have been selected, and their corresponding Fourier transforms (FTs) are analyzed for “Domain 1 (D1)”, “Domain 2 (D2)”, and the combined “D1+D2” region.

All three regions exhibit the same rhombohedral crystal structure with identical lattice parameters. The apparent differences arise because D1 and D2 are mirror images of the unit cell across the (100) plane, indicating that the columnar grains are heavily twinned. In the “D1+D2” region, two-dimensional projection overlaps results in the sum of diffraction signals from both domains, along with strong double diffraction due to dynamical effects. Certain spots, such as 001, 002, and 100, appear extinct in the “D1+D2” pattern. Nevertheless, the actual crystal structure remains rhombohedral (space group $R\bar{3}m$) with lattice parameters a = 2.85 Å and c = 14.25 Å (Figure R34).

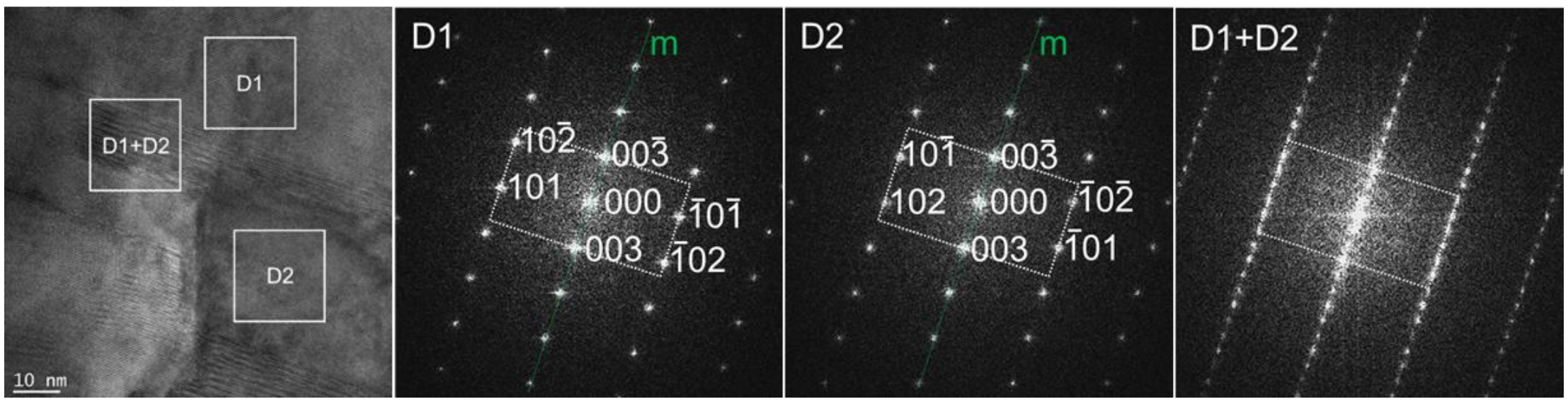

Figure R34. TEM images from the bulk of the bare LRMO particle and SEAD patterns for selected area

(iii) **Structural evolution after cycling**: while TEM and FFT analyses provide local structural information, they are not suitable for quantifying the overall phase transition extent. Therefore, we employed synchrotron XRD (sXRD) to analyze the LRMO cathode after charging to 4.6 V in the first cycle. The phase transition extent was quantified based on the integrated peak areas, yielding a value of approximately 12.6% (Figure R35). These results confirm that the S-species/spinel coating effectively suppresses phase transitions during electrochemical cycling.

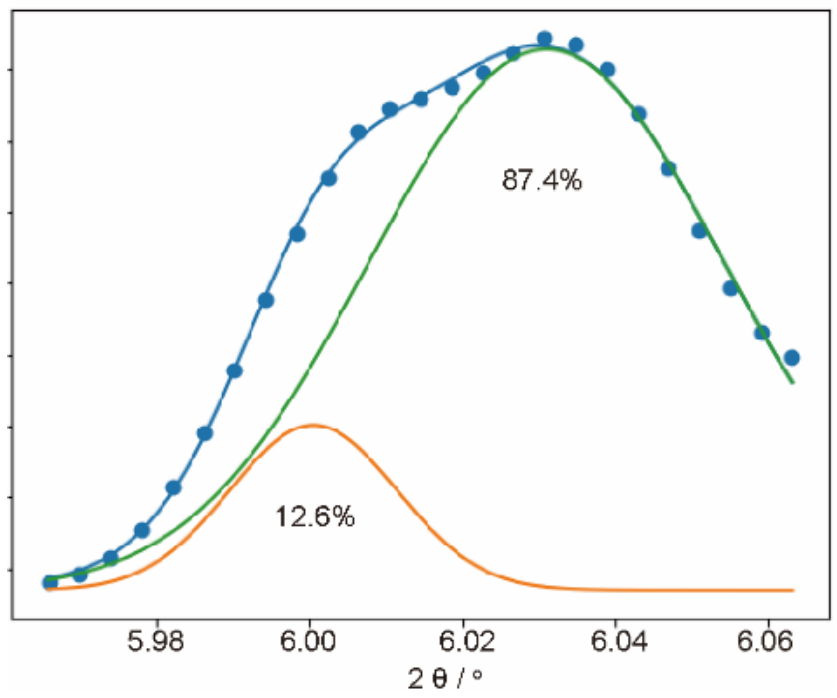

Figure R35. XRD pattern of cycled S@LRMO cathode composite.

And we removed the FFT patterns in Figure 1 before and integrated some of Figure R32 into main text, as follows:

"

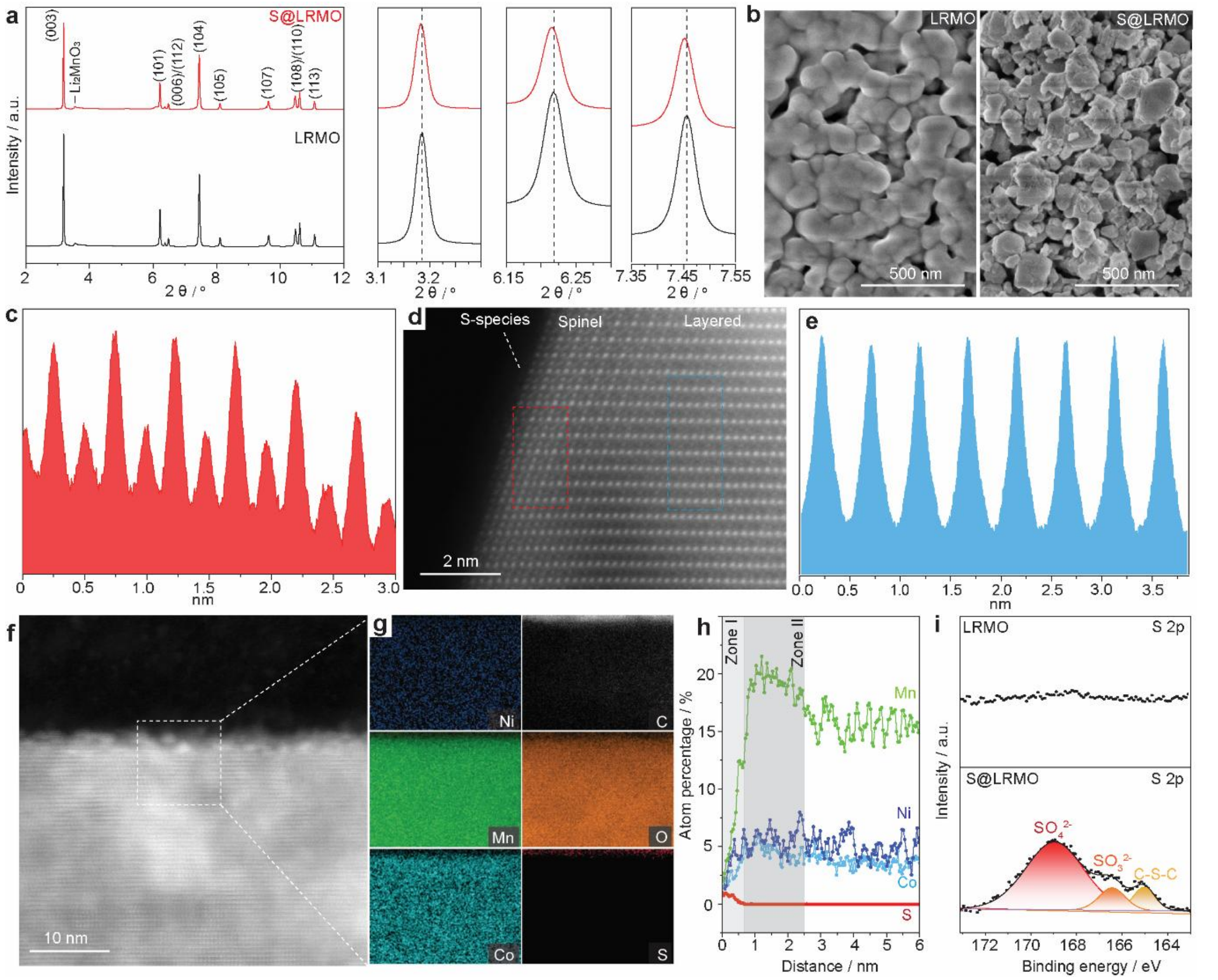

Figure 1. Cathode materials characterizations. *(a) Synchrotron X-Ray Diffraction (SXRD) patterns collected from the bare LRMO and S@LRMO powders; (b) SEM images of bare LRMO (left) and S@LRMO (right) particles; (d) High-Angle Annular Dark-field Scanning Transmission Electron Microscopy (HAADF-STEM) image from a S@LRMO cathode particle and the lattice space (c&e) of selected areas from its surface and bulk. (f) HAADF-STEM image from a S@LRMO cathode particle and its corresponding element maps in (g) extracted from the electron energy loss spectroscopy (EELS) data after mapping the region inside the white dashed box in the STEM image. (h) Element line profiles based on the maps in (g); (i) S 2p XPS spectrum for bare LRMO and S@LRMO particles."*

5. The manuscript would significantly benefit from including performance evaluation under industrially relevant high cathode loadings (≥15 mg/cm²), as the current low-loading data (6 mg/cm²) may not adequately represent practical application scenarios. High-loading conditions reveal critical challenges including interfacial contact resistance, ionic transport limitations, and mechanical stability issues - all essential factors for assessing commercial viability. Specifically, we recommend: (1) rate capability and long-term cycling tests under ≥15 mg/cm² loading to evaluate practical performance metrics; (2) mechanistic analysis of how increased loading affects electrode/electrolyte interface evolution and overall cell behavior; and (3) comparative discussion with established high-loading systems (e.g., DOI:10.1126/sciadv.add5189) to properly contextualize the technological advancement. These additions are crucial as they would more accurately reflect real-world operating conditions and better demonstrate the material's potential for scalable implementation in solid-state batteries.

Response:

Thank you for your suggestion. To address this, we performed electrochemical tests on S@LRMO cathodes with a mass loading of 15.28 mg/cm². The results are shown in Figure R36.

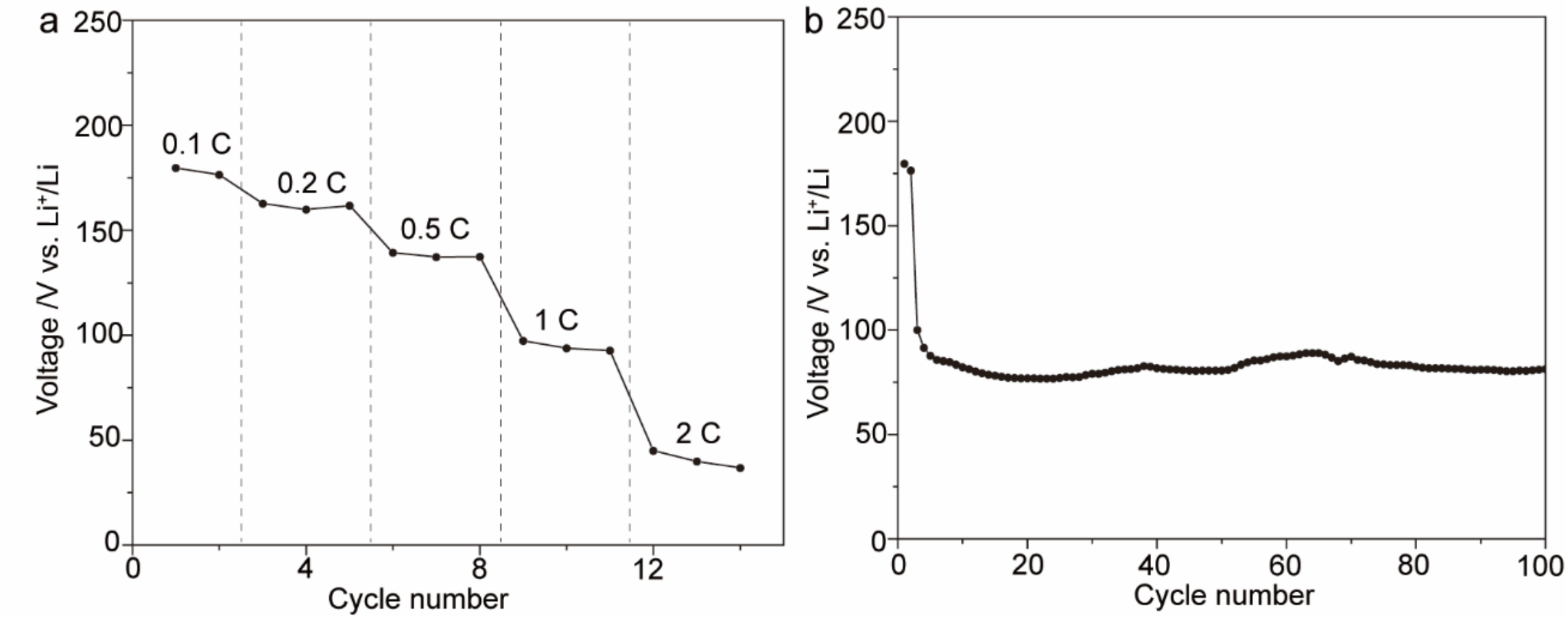

Figure R36. Rate capabilities and long-term cycling performance of S@LRMO with high mass loading of 15.28 mg/cm².

Compared to lower-loading cells (7.13 mg/cm²), the high-loading cells exhibit slightly reduced rate capability, which is likely due to intrinsic limitations of LRMO cathode kinetics and the secondary-particle morphology. Nevertheless, the high-loading cells maintain stable long-term cycling, indicating that the constructed electrode–electrolyte interface remains robust even under industrially relevant conditions (≥15 mg/cm²).

For a direct comparison with previously reported high-loading systems [2], we summarize the data in Table R5. While our $SC(NH_2)_2$@LRMO cathodes show slightly lower initial discharge capacity (~176 mAh/g at 0.1 C) than Li_2SiO_4@LRMO (~194.4 mAh/g at 0.1 C, roughly calculated), they demonstrate superior cycling stability, with no observable capacity decay after 100 cycles at 1 C, compared to 80.3% retention after 60 cycles for Li_2SiO_4@LRMO.

Table R5 Electrochemical performance of high-loading LRMO with Different Coatings

	Li_2SiO_4@LRMO	$SC(NH_2)_2$ @LRMO
Mass loading	12.6 mg/cm^2	15.2 mg/cm^2
Initial discharge specific capacity	~194.4 mAh/g (roughly calculated) at 0.1 C	176 mAh/g at 0.1C
Capacity retention	80.3 after 60 cycles at 0.2C	No obvious decay after 100 cycles at 1C

<u>Add we also put this part in the main text and Supplementary information:</u>

"After 500 cycles at 1 C, SSB-S@LRMO retained 86.8% of its initial mid-point voltage (Figure 2d, Figure S19), likely caused by increased resistance after initial cycles. To further assess the practical applicability of S@LRMO, cells with a high areal mass loading of 15.28 mg/cm² were evaluated. At 0.1 C, these cells exhibited a reduced discharge-specific capacity of approximately 174 mAh/g, which is likely attributable to kinetic limitations of the LRMO cathode and its secondary-particle morphology (Figure S21). Despite the lower capacity, the cells demonstrated stable long-term cycling performance, indicating that the engineered electrode–electrolyte interface remains stable during prolonged cycling even at high mass loading.

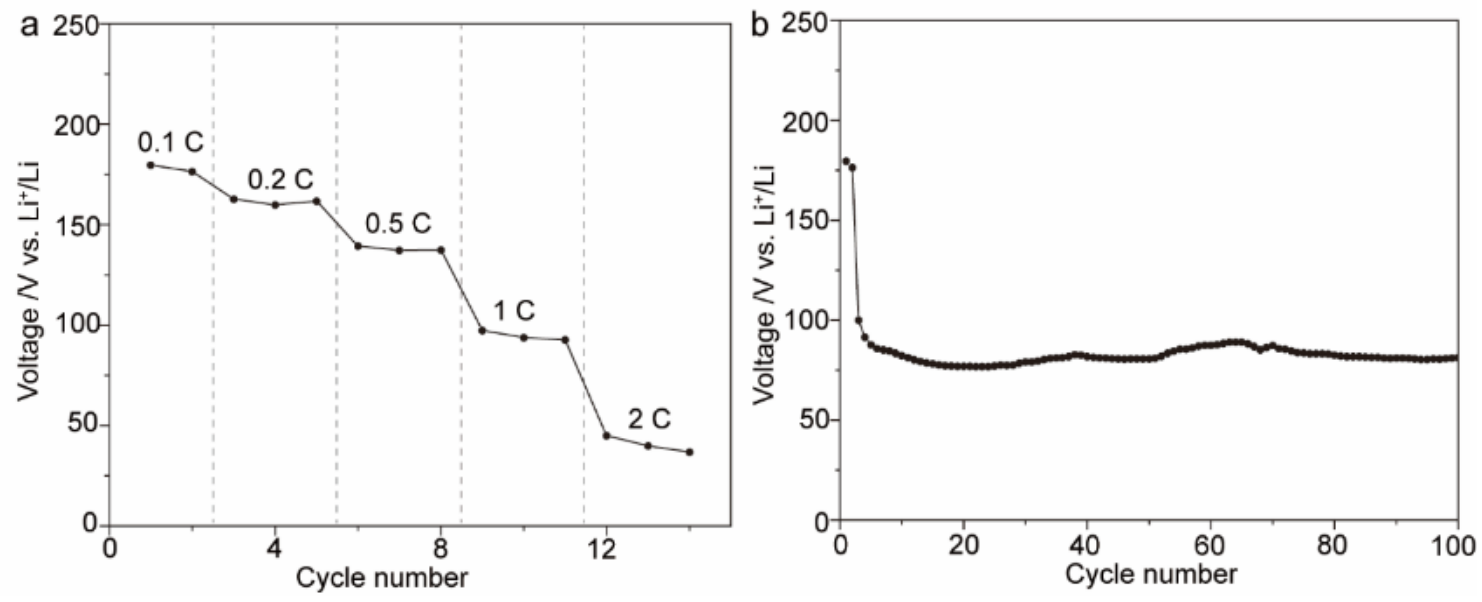

Figure S21. Rate capabilities and long-term cycling performance of S@LRMO with high mass loading of 15.28 mg/cm².”

6. In Figure 2f, the cyclic voltammetry (CV) results show improved reversibility of cation redox reactions. For Li-rich Mn-based cathodes in all-solid-state batteries, improving the reversibility of anion redox reactions is more crucial. In Figure 2f, S@LRMO exhibits significantly stronger oxidation peaks at 4.5V and reduction peaks at 3.2V, demonstrating that the sulfur-containing coating on LRMO enhances the reversibility of anion redox reactions. This issue warrants in-depth investigation, with a particular focus on elucidating how the sulfur coating stabilizes oxygen redox activity by suppressing oxygen evolution and maintaining structural integrity during cycling.

Response:

Thank you for your respond. In Li-rich Mn-based cathodes, limited reversibility of oxygen redox leads to interfacial degradation and Li-ion blocking compounds. In S@LRMO, the thiourea-derived sulfur coating converts reactive Li-O-Li oxygen into stable Mn-O-Mn lattice oxygen, forming a robust cathode-electrolyte interface. This stabilizes oxygen redox activity, suppresses oxygen evolution, and maintains structural integrity during long-term cycling. As evidenced by CV (Figure 2f), S@LRMO exhibits stronger oxidation peaks at ~4.5 V and reduction peaks at ~3.2 V, directly demonstrating enhanced anion redox reversibility. This interfacial engineering underpins the improved cycling stability and high-capacity retention.

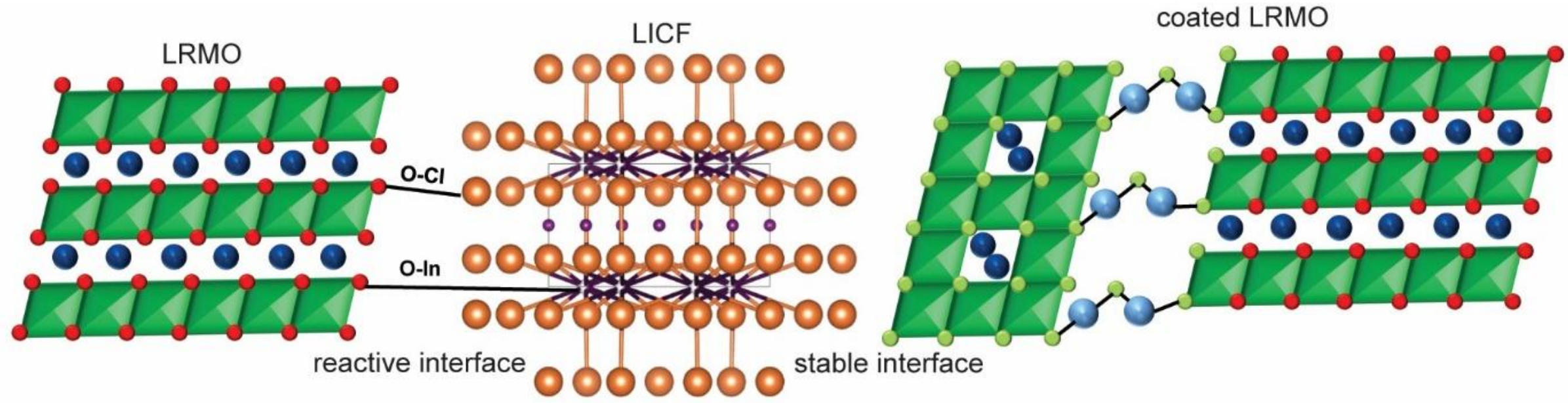

Figure R37 schematic illustration of interfaces of cathode and solid electrolytes for both LRMO | LICF and S@LRMO|LICF.

7. Related to the previous question, while this work demonstrates the effects of sulfur/sulfur-containing coating modification on performance, the underlying mechanisms remain unexplored, limiting the depth of the study. A more thorough investigation into the interaction mechanisms (e.g., chemical bonding, physical adsorption, or electron transfer between sulfur species and the host material) would significantly strengthen the work. Techniques such as Raman spectroscopy,

or DFT calculations could help elucidate the sulfur-coating process and its role in enhancing material properties. Additionally, clarifying the thermodynamic/kinetic aspects of sulfur incorporation and its influence on [specific property, e.g., conductivity, stability] would provide valuable insights.

Response:

Thank you for your suggestions here. To uncover the underlying mechanisms on enhanced performance originating from dual-layer coating modification on LRMO surface, density functional theory (DFT) simulations was conducted.

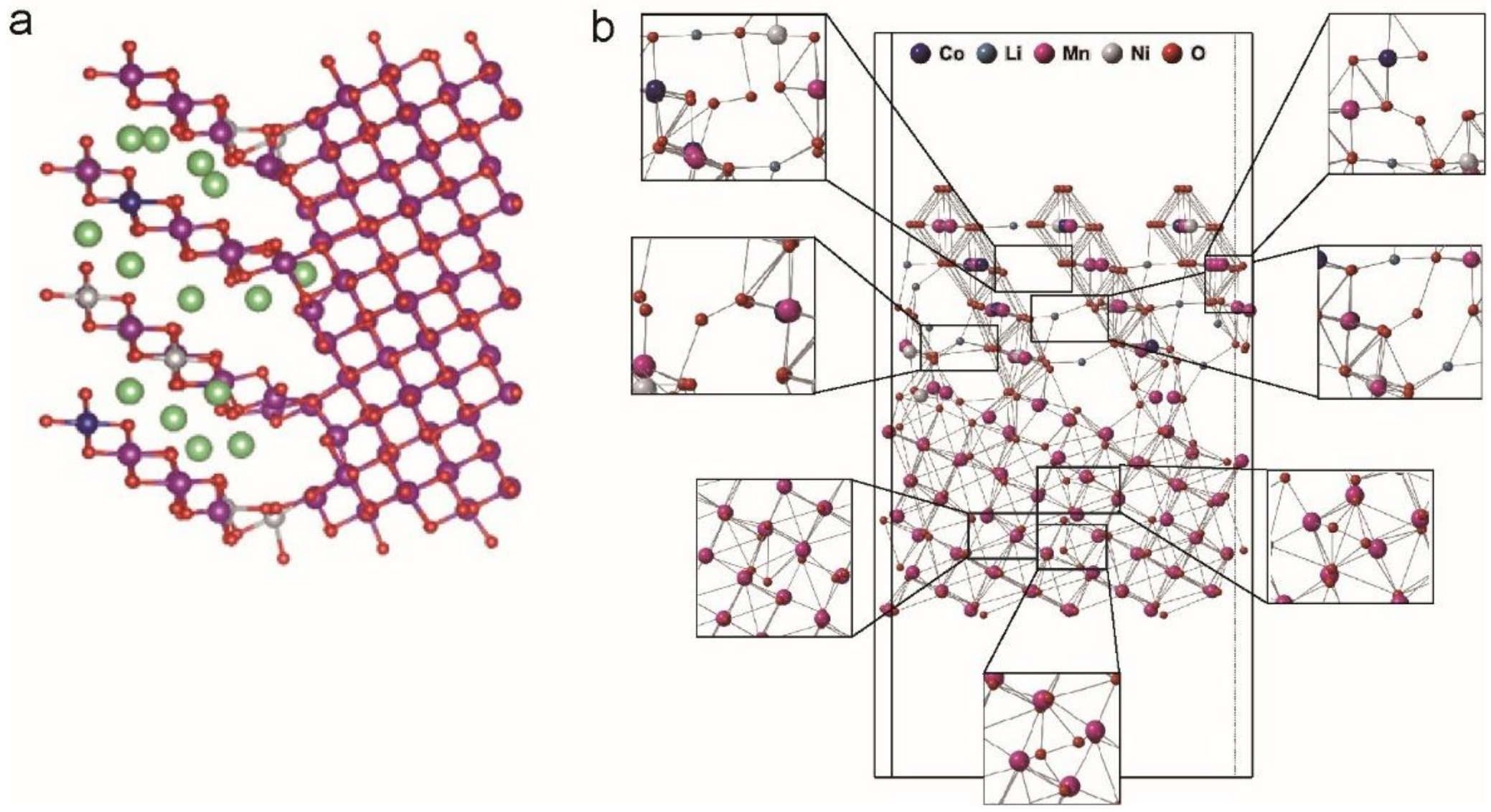

Figure R38. Initial LRMO and spinel $LiMn_2O_4$ interface model (a) and peroxide structures (b).

To further substantiate the hypothesis that the spinel $LiMn_2O_4$ surface layer mitigated degradation by suppressing oxygen loss, density functional theory (DFT) calculations were performed to compare the energetics of oxidized anionic species formation in LRMO and spinel $LiMn_2O_4$. An interface model comprising LRMO in the (104) orientation and spinel $LiMn_2O_4$ in the (111) orientation was constructed to enable a direct comparison of peroxide bond formation energetics (Fig. S30). This interface provides a common thermodynamic reference for delithiation-driven anionic redox processes.

Peroxide bonds were introduced at multiple sites within the bulk-like regions of both the LRMO and spinel slabs, accompanied by the removal of two Li atoms from the LRMO side to initiate anionic redox. Importantly, even when peroxide bonds were introduced in the spinel layer, the delithiation origin remained on the LRMO side, ensuring consistent comparison of total energies between the two phases. Because peroxide bonds were introduced in bulk-like layers rather than at the immediate interface, the calculated relative energetics are not expected to be sensitive to the precise interface structure or surface facet selection. Although Li/vacancy configurations can influence peroxide stability, this effect was isolated by using a common Li/vacancy arrangement derived from a reference LRMO structure containing a peroxide bond. This configuration served as the initial state for peroxide formation in the spinel $LiMn_2O_4$ layer, while allowing full ionic relaxation.

As summarized in Table R6, peroxide formation is significantly less favorable in spinel $LiMn_2O_4$ than in LRMO. The most stable peroxide configuration in the spinel layer is 2.59 eV higher in energy than the lowest-energy configuration in the layered structure. Moreover, peroxide stability exhibits strong dependence on the local chemical environment, with energy spreads of approximately 2.3 eV and 3.9 eV for LRMO and spinel $LiMn_2O_4$, respectively.

These results demonstrate that the formation of oxidized anionic species in the form of peroxides is intrinsically unfavorable in spinel $LiMn_2O_4$, even when interfaced with LRMO and when anionic redox is initiated by over-delithiation of the layered phase. Consequently, the migration of oxidized anionic species through the spinel surface layer would require energetically prohibitive stabilization within the spinel lattice. This energetic barrier effectively suppresses oxygen migration to the surface and prevents oxygen loss, in strong agreement with the experimental observations.

Table R6 Relative energetics of peroxides formed in LRMO and spinel $LiMn_2O_4$ layers of the interface model. The peroxide bond distance was set to 1.4 Å and ΔE was determined by the difference in total energy between the given structure and the structure of peroxide 1.

Peroxide	ΔE	Structure type	Oxygen indices
4	0 eV	LRMO	227, 261
3	1.5 eV	LRMO	257, 259
1	1.5 eV	LRMO	185, 201
2	2.3 eV	LRMO	192, 200
5	2.6 eV	spinel	136, 164
6	3,6 eV	spinel	101, 130
7	6.4 eV	spinel	110, 136

All DFT-related parts are integrated into main text and Supplementary information, shown below:

"To further substantiate the hypothesis that the spinel $LiMn_2O_4$ surface layer mitigated degradation by suppressing oxygen loss, density functional theory (DFT) calculations were performed to compare the energetics of oxidized anionic species formation in LRMO and spinel $LiMn_2O_4$.

…

This energetic barrier effectively suppresses oxygen migration to the surface and prevents oxygen loss, in strong agreement with the experimental observations."

"

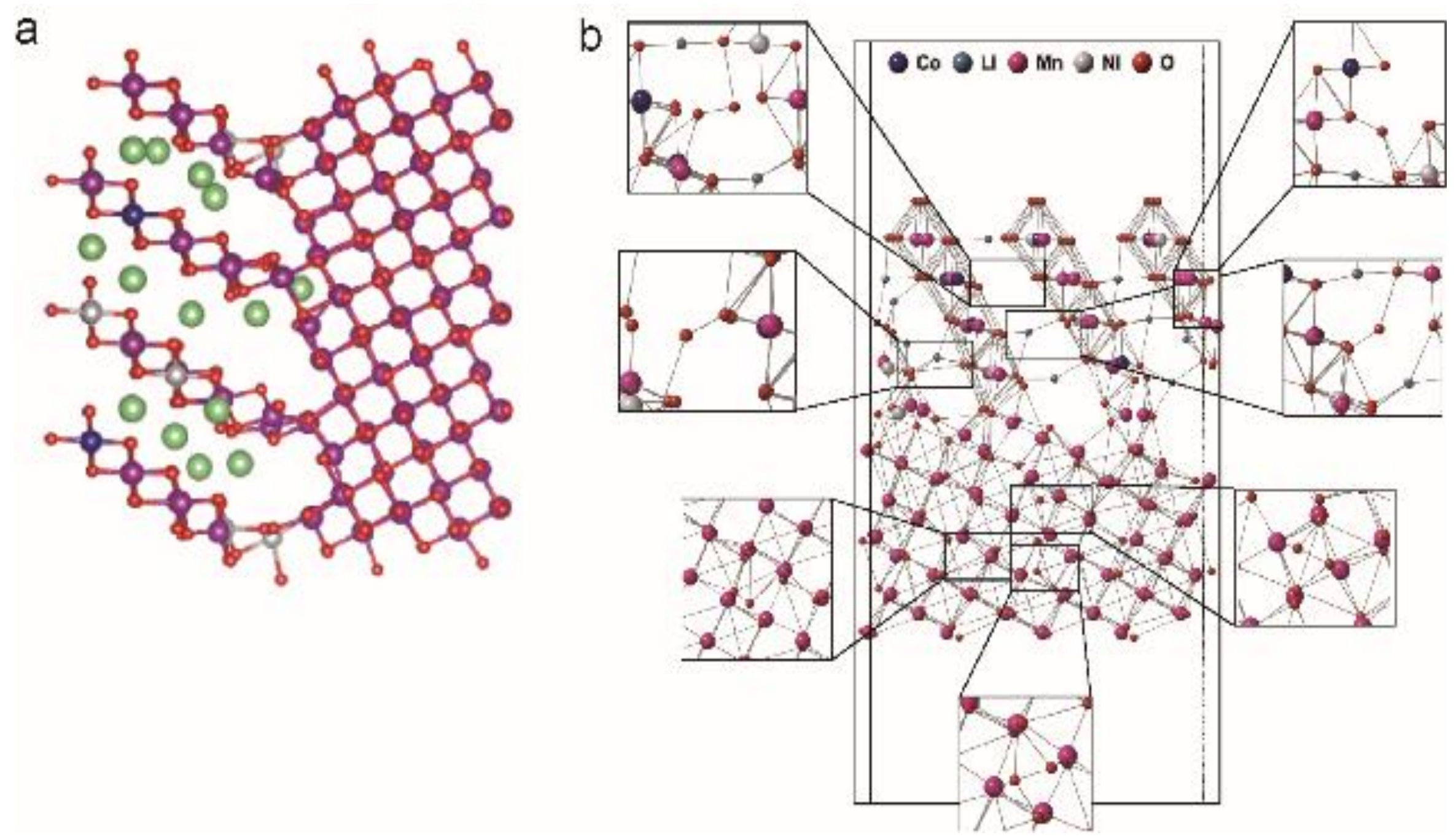

Figure S30. Initial LRMO and spinel $LiMn_2O_4$ interface model (a) and peroxide structure (b).

Supplementary Table S5 Relative energetics of peroxides formed in LRMO and spinel $LiMn_2O_4$ layers of the interface model. The peroxide bond distance was set to 1.4 Å and ΔE was determined by the difference in total energy between the given structure and the structure of peroxide 1.

Peroxide	*ΔE*	*Structure type*	*Oxygen indices*
4	*0 eV*	*LRMO*	*227, 261*
3	*1.5 eV*	*LRMO*	*257, 259*
1	*1.5 eV*	*LRMO*	*185, 201*
2	*2.3 eV*	*LRMO*	*192, 200*
5	*2.6 eV*	*spinel*	*136, 164*
6	*3,6 eV*	*spinel*	*101, 130*
7	*6.4 eV*	*spinel*	*110, 136*

8. The authors must supplement XPS depth profiling data, including: 1) Chemical state changes of In 3d orbitals before and after cycling; 2) Interface element distribution and valence state evolution; 3) Comparative analysis between coated and uncoated samples. These data are crucial for verifying the mechanism by which the coating layer suppresses electrolyte oxidation.

Response:

Thank you for your suggestions, we have performed XPS depth profiling to elucidate the effect of the sulfur coating on the electrode–electrolyte interface, focusing on chemical state evolution and element distribution.

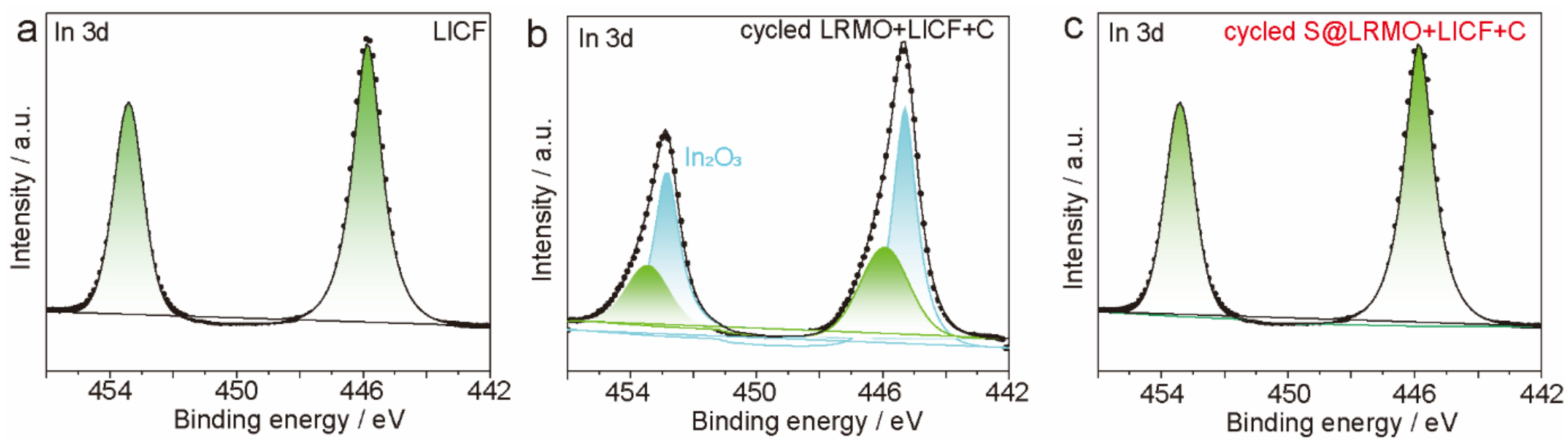

Figure R39 Chemical state changes of In 3d orbitals for LICF (a), both LRMO (b) and coated LRMO (c) cathode composites after cycling.

Figure R39 presents the In 3d spectra for LICF, uncoated LRMO, and S@LRMO cathode composites after long-term cycling. For the uncoated LRMO cathode, two additional peaks appear in the In 3d spectrum, corresponding to the formation of In_2O_3 in LICF, indicating irreversible electrolyte oxidation. In contrast, no such peaks are observed in the S@LRMO composite, demonstrating that the sulfur-containing coating effectively suppresses interfacial oxidation. These findings are consistent with the TOF-SIMS results shown in Figure 4 of the main text.

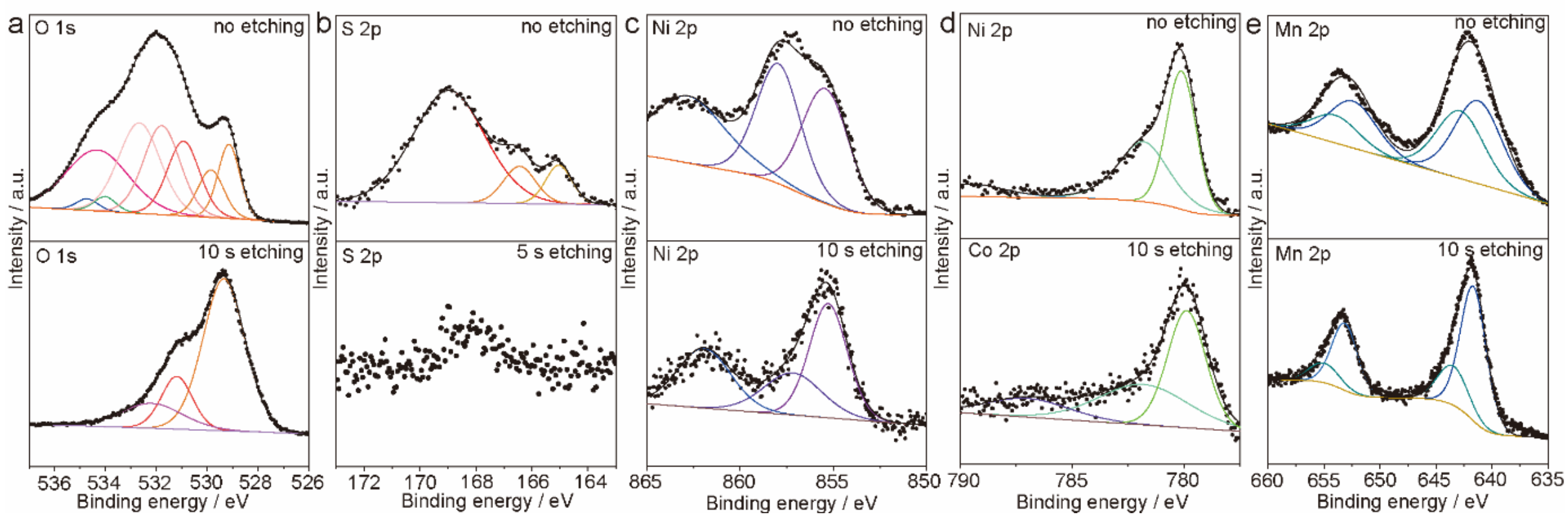

Figure R40 O 1s (a), S 2p (b), Co 2p (c), Ni 2p (d) and Mn 2p (e) XPS spectra for S@LRMO with / without etching, respectively.

Figure R40 shows XPS spectra of O 1s, S 2p, Co 2p, Ni 2p, and Mn 2p for S@LRMO before and after etching. After etching, extra peaks in O 1s disappear and no obvious S 2p signals are detected, confirming that sulfur species are concentrated at the surface. Additionally, transition metal ions exhibit higher chemical states at the surface than in the bulk, reflecting surface oxidation during the annealing process in air.

And we also integrated these graphs into main text and Supplementary information, as follows:

"

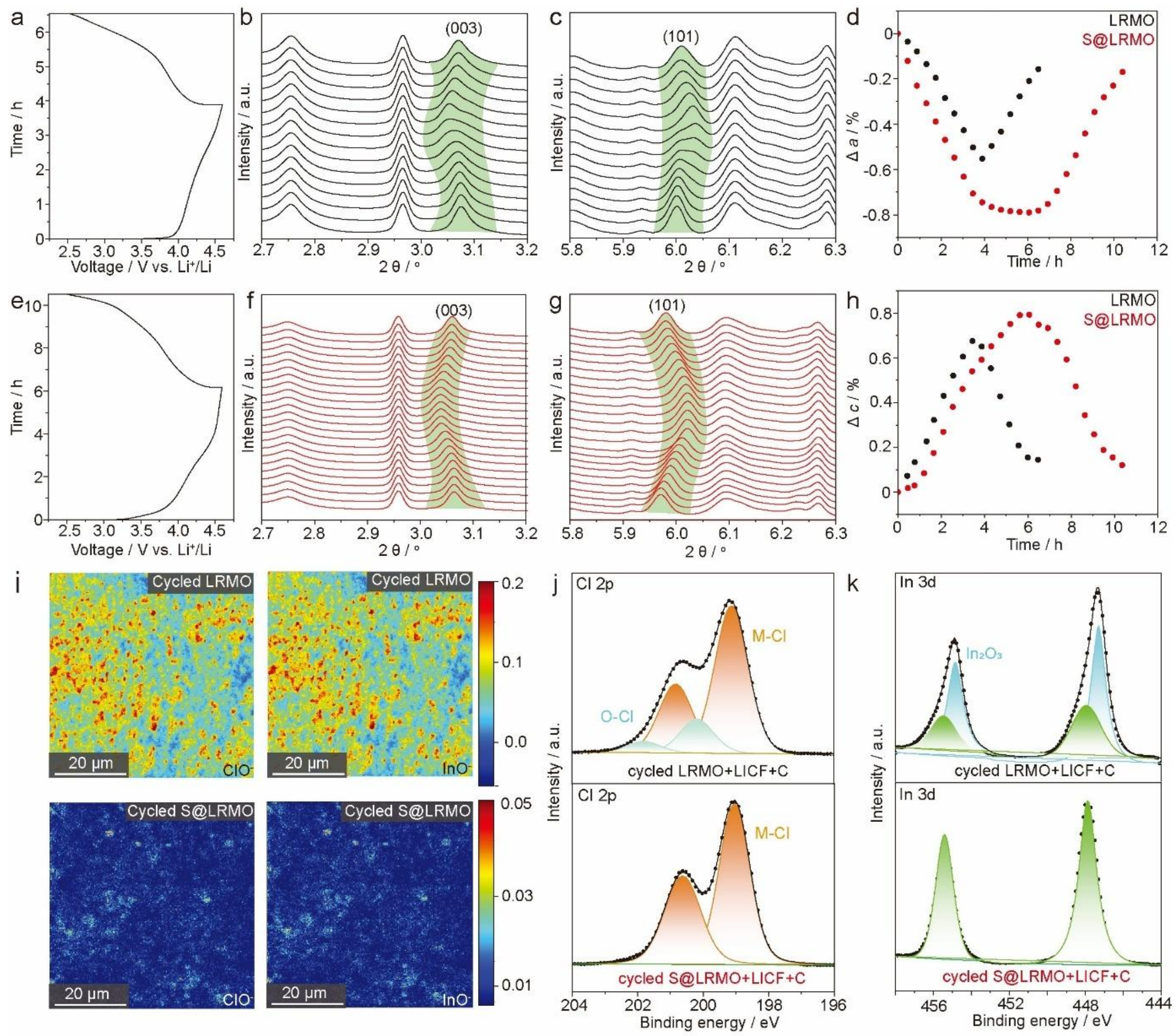

Figure 4. Operando SXRD characterization during the first charge/discharge and post-mortem physicochemical characterizations. Operando synchrotron XRD data was collected from SSB-LRMO and SSB-S@LRMO cells in the initial cycle. Galvanostatic charge-discharge curve for LRMO (a) and S-LRMO (e) based SSBs in the initial cycle. Selected areas operando synchrotron XRD patterns of corresponding to (003) and (101) peaks for LRMO (b-c) and S-LRMO (f-g) based SSBs; Relative changes of lattice parameters containing a (d) and c (h) of SSB-LRMO and SSB-S@LRMO during the initial cycle. Note that values are the statistically averaged results of many particles inside the operando cells. (i) TOF-SIMS graph of ClO$^-$ and InO$^-$ for cycled LRMO composites and cycled S@LRMO composites. (j&k) XPS spectra of Cl 2p and In 3d for cycled LRMO composites and cycled S@LRMO composites.

“

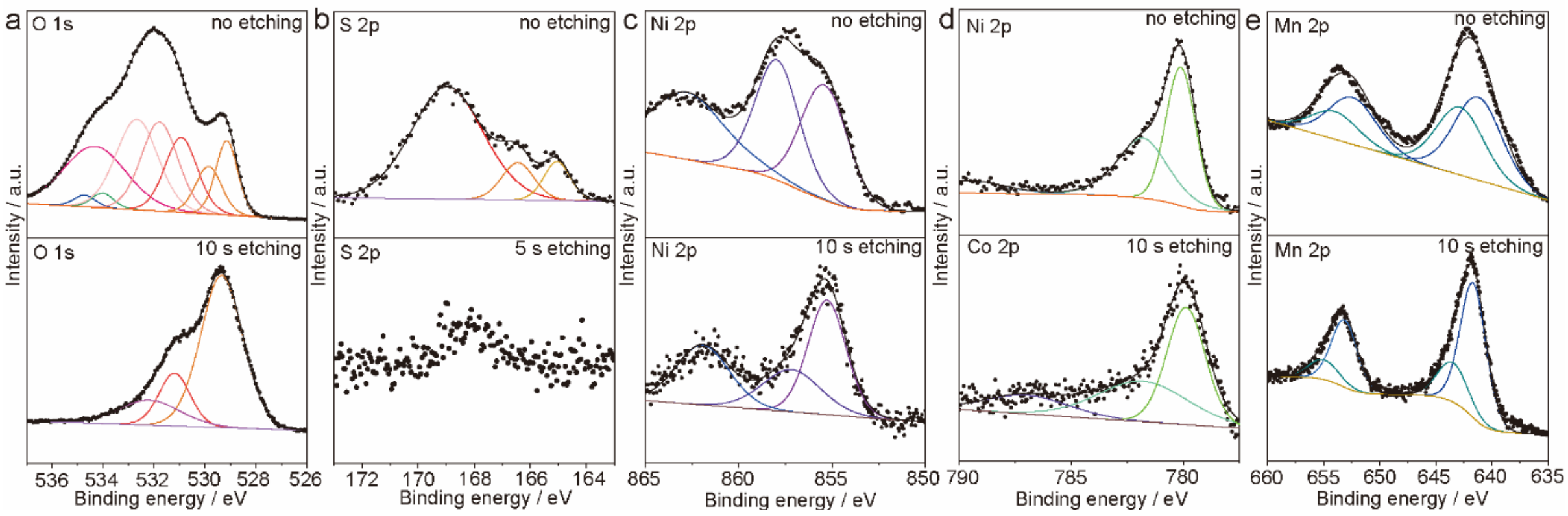

Figure R13 O 1s (a), S 2p (b), Co 2p (c), Ni 2p (d) and Mn 2p (e) XPS spectra for S@LRMO with / without etching, respectively.

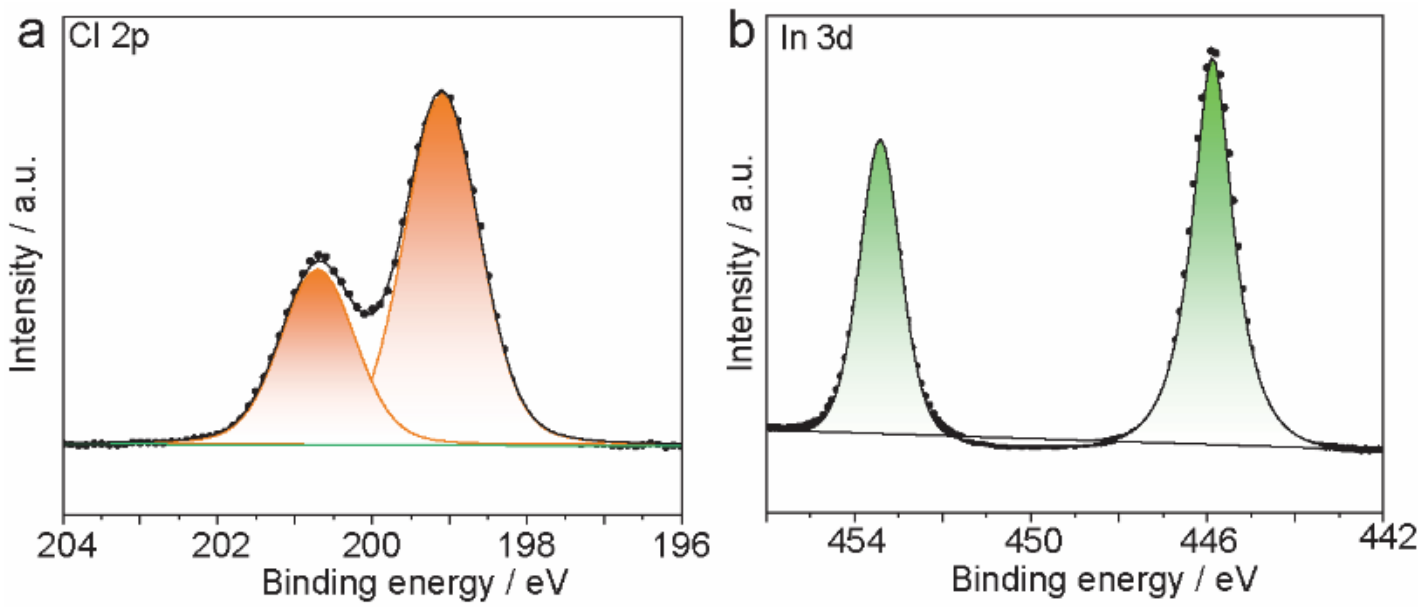

Figure S28. Cl 2p and In 3d XPS spectra of LICF.”

References:

1. Sun, S. *et al.* Eliminating interfacial O-involving degradation in Li-rich Mn-based cathodes for all-solid-state lithium batteries. *Sci. Adv.* **8**, eadd5189 (2022).
2. Kong, W.-J. *et al.* Bulk/Interfacial Structure Design of Li-Rich Mn-Based Cathodes for All-Solid-State Lithium Batteries. *J. Am. Chem. Soc.* 28190−28200 (2024) doi:10.1021/jacs.4c08115.
3. Sun, S. *et al.* Boosting Anionic Redox Reactions of Li-Rich Cathodes through Lattice Oxygen and Li-Ion Kinetics Modulation in Working All-Solid-State Batteries. *Adv. Mater.* **37**, 2414195 (2025).
4. Chen, X. *et al.* Improved stability against moisture and lithium metal by doping F into Li3InCl6. *J. Power Sources* **545**, 231939 (2022).
5. Zhang, S. *et al.* Advanced High-Voltage All-Solid-State Li-Ion Batteries Enabled by a Dual-Halogen Solid Electrolyte. *Adv. Energy Mater.* **11**, 2100836 (2021).
6. Chen, J. *et al.* Long Cycle Life All-Solid-State Batteries Enabled by Medium Nanosized Catholytes. *J. Phys. Chem. Lett.* **16**, 731–737 (2025).
7. Liu, Y. *et al.* Surface-Functionalized Coating for Lithium-Rich Cathode Material To Achieve Ultra-High Rate and Excellent Cycle Performance. *ACS Nano* **13**, 11891–11900 (2019).

Response to Reviewer

Reviewer #1 (Remarks to the Author):

The authors have addressed some of our previous concerns, and the revised manuscript shows improvement. However, several of the earlier comments have not been adequately or correctly addressed. Therefore, the manuscript remains insufficient for publication at this stage.

1. (Comment #3) The authors continue to claim that an almost single atomic layer of sulfur species is coated on the LMRO surface. As evidence, Figure 1g presents a TEM-EDS sulfur mapping image. However, in principle, TEM-EDS mapping is not capable of reliably detecting a single atomic layer of sulfur. Therefore, the presented EDS mapping does not provide convincing evidence to support this claim.

Respond: We thank the Reviewer for this important comment. First, we would like to clarify that the sulfur mapping shown in Figure 1g was obtained by STEM-EELS mapping, rather than TEM-EDS mapping. We apologize for the incorrect description in the previous version of the manuscript and have corrected this throughout the revised manuscript.

Furthermore, we would like to clarify that our results do not suggest the presence of a single atomic layer. Instead, the characterization indicates an "ultrathin" sulfur-containing coating layer with a thickness of approximately 3-5 atomic layers. STEM-EELS provides significantly higher spatial resolution and sensitivity to light elements than conventional TEM-EDS, making it suitable for detecting such ultrathin surface layers.

In addition, the sulfur signal is spatially localized at the particle surface in the STEM-EELS elemental mapping, which is consistent with the formation of a thin surface coating rather than bulk incorporation. The manuscript has been revised to clarify this point and avoid any ambiguity regarding the coating thickness.

To make it clear, we integrated this part into main text in the revised manuscript, as follows:

"Scanning electron microscopy (SEM) images reveal that the LRMO surface became rougher after coating (Figure 1b & Figure S3), hinting towards a successful surface modification. High-angle annular dark-field - scanning transmission electron microscopy (HAADF-STEM) image of S@LRMO showed two-layered coating and the inert layer was spinel phase (Figure 1c-e). and TEM combined with electron energy loss spectroscopy (EELS) along the cross-section of S@LRMO further proved the existence of two-layered coating, which contained 0.7 nm thick S-containing species (shown in EELS mapping of Sulfur in Figure 1g and line scan spectra highlighted in Zone I in Figure 1h) and Mn-rich layer (see Zone II in Figure 1h) on LRMO particles"

2. (Comments #4, #5, and #7 of Reviewer #1) These comments have not been adequately addressed. The authors did not provide direct experimental evidence but instead proposed plausible mechanisms for the formation of a $LiMn_2O_4$-like spinel phase and for the role of surface modification in bulk diffusivity. Without supporting data, these explanations remain speculative.

Respond: We sincerely thank the Reviewer for this important comment. We

acknowledge that direct experimental verification of the individual reactions proposed in the manuscript is challenging under the present experimental conditions. The following reactions were therefore proposed as plausible reaction pathways based on established chemical knowledge and literature reports:

1. $SC(NH_2)_2 + 2OH^- \rightarrow S^{2-} + CO(NH_2)_2 + H_2O$
2. $2CO(NH_2)_2 \rightarrow NH_3 + 2HCNO$
3. $O_2 + S^{2-} + Li_2MnO_3 \rightarrow SO_3^{2-} + SO_4^{2-} + LiMn_2O_4$

Previous studies have shown that under alkaline conditions, thiourea can undergo desulfurization and C=S bond cleavage, particularly in the presence of hydroxide ions or metal species, producing urea-type products while releasing reactive sulfur species (Reaction 1)[1]. The resulting urea can further decompose into ammonia and other small molecules under reaction conditions, as reported in the literature (Reaction 2) [2].

In addition, the XRD patterns shown in Fig. S14 indicate the formation of a spinel-type phase after the high-temperature oxidation treatment. It is also well documented that under oxidative or alkaline environments, reduced sulfur species (e.g., those derived from thiourea decomposition) can be progressively oxidized to sulfite (SO_3^{2-}) and sulfate (SO_4^{2-}) species[3].

Considering these reported reactions together with the known tendency of Li-rich manganese oxides to undergo surface reconstruction toward spinel-like structures during high-temperature treatment, the formation of sulfite/sulfate surface species accompanied by a $LiMn_2O_4$-like spinel surface region represents a chemically reasonable explanation for the observed results.

To avoid overstating the conclusions, we have revised the manuscript to clarify that these reactions represent a plausible mechanistic interpretation rather than directly proven elementary steps, and we have modified the corresponding discussion accordingly.

<u>To make it clear, we integrated related references into main text in the revised manuscript, as follows:</u>

"Based on the above results and analyses, we propose that the formation of sulfur-containing species and the spinel phase proceeds via the following reactions. When LRMO particles with an alkaline surface are introduced into the solvent, thiourea decomposes according to Reaction (1) [20]*:"*

"Subsequently, the generated $CO(NH_2)_2$ further decomposes to produce NH_3, as described by Reaction (2) [21]*. The absence of a discernible N 1s signal in the XPS spectra (Fig. S13) is consistent with the volatilization of nitrogen-containing species during this process:"*

"During the annealing step, the produced S^{2-} species and/or NH_3 act as reducing agents, promoting the partial reduction of Li_2MnO_3 and inducing its transformation into the spinel $LiMn_2O_4$ phase [22]*. Concurrently, S^{2-} is oxidized to sulfite and sulfate species, as summarized in Reaction (3):"*

3. (Comment #6) The role of surface modification in improving the reversibility of oxygen redox reactions remains unclear. In general, the reversibility of oxygen redox reactions is primarily governed by the bulk crystal structure, such as the degree of cation disorder. This point should be more clearly discussed and supported with appropriate evidence.

Respond: Oxygen release in Li-rich Mn-based cathodes originates from the inherent instability of the Li-O-Li, where high-voltage charging induces transition metal migration and collapse of the layered structure, leading to capacity fade. However, oxygen must ultimately diffuse through the particle surface, which is prone to oxidizing solid electrolytes, increasing interfacial resistance and initial capacity loss. Consequently, despite its bulk origin, surface coating remains a highly effective strategy to enhance cycle stability by providing a protective layer or surface reconstruction that mitigates oxygen evolution, suppresses parasitic reactions, and partially stabilizes the underlying bulk structure.

In this work, we introduced S-species containing coating and surface reconstruction into spinel to stabilize improve the reversibility of oxygen redox reactions. Based on our structural and electrochemical characterization, both the sulfur-containing surface layer and the spinel-type surface reconstruction contribute to the enhanced cycling stability and suppression of oxygen loss in S@LRMO. Specifically, the sulfur-derived surface layer primarily stabilizes the electrode-electrolyte interface by avoiding physical contact between S@LRMO and solid electrolyte. And spinel-like reconstruction provides additional structural reinforcement of the surface transition-metal-oxygen framework, which supports interfacial integrity during cycling. Numerous works have been reported on enhancing LRMO stabilities through surface modification, especially surface reconstruction into spinel phase. [4–10]

Reviewer #2 (Remarks to the Author):

I have reviewed the revised manuscript and the rebuttal letter. The authors have done a good job in addressing the reviewers' comments thoroughly and convincingly. The studies on coating contents, high-mass-loading performances, and structural characterizations further strengthen the work's scientific value and practical relevance. Before final acceptance, I would suggest some minor revisions to further improve clarity and consistency.

1. The authors compare the performance of S@LRMO with other coated LRMO systems in Table S4, but the table seems not specify the testing conditions for each reference. Adding the details would enhance the comparability and contextual relevance of the data.

Respond: We thank the Reviewer for this helpful suggestion. We agree that testing conditions can significantly influence electrochemical performance, and providing this information improves the comparability of the reported results.

Accordingly, we have added the relevant testing parameters for each reference in the revised table (Table R1), including voltage window, initial discharge capacity (IDC), initial coulombic efficiency (ICE), mass loading, long-term cycling performance, and test temperature.

The revised comparison table is shown below:

LRMO	Coating	Voltage /V	IDC@0.1 C /mAh/g	ICE	Mass loading/ mg/cm2	C-rate/C	Long-term cycling performances	Test T/ °C	Ref.
$Li_{1.2}Mn_{0.54}Co_{0.13}Ni_{0.13}O_2$	Li_2WO_4	2.2-4.6	218	74.83 %	7.64	1	81% retention after 1200 cycles	25	1
$Li_{1.2}Mn_{0.54}Co_{0.13}Ni_{0.13}O_2$	Li_2SO_4	2.3-4.6	248	94 %	4.2	1	81% retention after 300 cycles	25	2
$Li_{1.17}Mn_{0.55}Co_{0.05}Ni_{0.24}O_2$	Li_3PO_4	2.0-4.8	230	83.0 %	12.7	0.2	60 % retention after 431cycles	25	3
$Li_{1.2}Mn_{0.54}Co_{0.13}Ni_{0.13}O_2$	$LiNbO_3$	4.8	221	73.21 %	0.88	0.05	67 % retention after 100 cycles	25	4
$Li_{1.2}Mn_{0.54}Co_{0.13}Ni_{0.13}O_2$	$SC(NH_2)_2$	2.5-4.6	220	84.83 %	7.13	1	97 % retention after 600 cycles	25	This work

To make it clear, we integrated this part into main text in the revised supporting information, as follows:

Table S4: Comparison advanced coatings on LRMO and corresponding electrochemical performance of all-solid state lithium batteries.

LRMO	Coating	Voltage /V	IDC@0.1 C /mAh/g	ICE	Mass loading/ mg/cm2	C-rate/C	Long-term cycling performances	Test T/ °C	Ref.
$Li_{1.2}Mn_{0.54}Co_{0.13}Ni_{0.13}O_2$	Li_2WO_4	2.2-4.6	218	74.83 %	7.64	1	81% retention after 1200 cycles	25	1
$Li_{1.2}Mn_{0.54}Co_{0.13}Ni_{0.13}O_2$	Li_2SO_4	2.3-4.6	248	94 %	4.2	1	81% retention after 300 cycles	25	2
$Li_{1.17}Mn_{0.55}Co_{0.05}Ni_{0.24}O_2$	Li_3PO_4	2.0-4.8	230	83.0 %	12.7	0.2	60 % retention after 431cycles	25	3
$Li_{1.2}Mn_{0.54}Co_{0.13}Ni_{0.13}O_2$	$LiNbO_3$	4.8	221	73.21 %	0.88	0.05	67 % retention after 100 cycles	25	4
$Li_{1.2}Mn_{0.54}Co_{0.13}Ni_{0.13}O_2$	$SC(NH_2)_2$	2.5-4.6	220	84.83 %	7.13	1	97 % retention after 600 cycles	25	This work

2. The manuscript mentions that the spinel phase facilitates Li+ transport, while it seems to lack direct comparative data on Li^+ diffusivity within the spinel layer versus the bulk LRMO. Adding a brief discussion or supplementary data on the intrinsic diffusivity of the spinel phase would further corroborate this claim.

Respond: We thank the Reviewer for this valuable comment. We note that the spinel-like surface layer in S@LRMO is expected to facilitate Li^+ transport primarily at the particle-electrolyte interface, while the bulk layered LRMO structure remains largely unchanged.

Directly measuring the Li^+ diffusion coefficient specifically within such a thin surface layer is experimentally challenging. The spinel-like region identified in this work is only a few nm thick, and most used electrochemical techniques for determining Li^+ diffusion coefficients (e.g., GITT, EIS, or PITT) probe the overall electrode response, which is dominated by bulk diffusion in the core active material rather than the transport properties of an ultrathin surface reconstruction layer. As a result, isolating the intrinsic Li^+ diffusivity of the surface layer from that of the bulk LRMO is not straightforward.

Nevertheless, spinel $LiMn_2O_4$ exhibits higher intrinsic Li^+ diffusivity than layered Li-rich manganese oxides due to fundamental structural differences. In $LiMn_2O_4$, lithium ions occupy tetrahedral 8a sites and migrate via interconnected 8a-16c-8a pathways, forming a three-dimensional diffusion network with low migration barriers (~0.32-0.4 eV), which enables rapid, isotropic Li^+ transport[11]. In contrast, Li^+ migration in Li-rich layered oxides is largely restricted to two-dimensional pathways within the Li layers, and Li/Mn ordering in the Li_2MnO_3 domains partially blocks diffusion channels, increasing the migration barrier (~0.6-0.9 eV)[12,13]. Consequently, Li^+ diffusion coefficients in layered oxides are typically one to several orders of magnitude lower than in spinel $LiMn_2O_4$. The formation of a thin spinel-like surface layer thus provides faster interfacial Li^+ pathways, mitigating kinetic limitations and enhancing overall electrochemical performance.

To clarify this point, we have added a brief discussion in the revised manuscript and cited relevant literature, emphasizing that the enhanced kinetics are primarily associated with improved interfacial Li^+ transport rather than a modification of bulk Li^+ diffusivity.
In contrast, S@LRMO exhibits higher D(Li^+) values at high SOC compared to LRMO, likely due to eliminated interfacial reactions at the LICF | LRMO interface and reduced irreversible phase transitions induced by oxygen release at high voltage. ***Moreover, the formation of spinel-type Mn-rich species towards the interface is likely to promote Li^+ transport. These spinel domains provide three-dimensional Li^+ diffusion pathways that bypass the intrinsically limited two-dimensional diffusion within the bulk layered structure. As a result, faster interfacial Li^+ transport is enabled, leading to improved kinetic behavior and enhanced overall electrochemical performance.***

3. In the discussion of oxygen redox reversibility, the authors attribute the improved stability to the synergistic effect of the sulfur coating and spinel phase. A brief summary of which component plays a more dominant role in suppressing oxygen loss would provide more focused mechanistic insights.

Respond: We thank the reviewer for this insightful comment. Based on our structural and electrochemical characterization, both the sulfur-containing surface layer and the spinel-like surface reconstruction contribute to the improved cycling stability and enhanced reversibility of oxygen redox in S@LRMO.

The sulfur-derived surface layer mainly stabilizes the electrode–electrolyte interface by reducing parasitic interfacial reactions, which helps mitigate surface oxygen release and suppress electrolyte-induced surface degradation. Meanwhile, the spinel-like surface

reconstruction strengthens the local transition-metal–oxygen framework at the particle surface, providing greater structural robustness during repeated lithium extraction and insertion.

Overall, we consider the spinel-type surface reconstruction to play the dominant role in suppressing oxygen loss, as it stabilizes the surface lattice and helps maintain the integrity of the oxygen framework during cycling. The sulfur-containing surface layer acts synergistically, further improving interfacial stability and protecting the reconstructed surface region.

To improve clarity, we have added a brief summary in the revised manuscript highlighting the respective roles of the spinel-like surface structure and the sulfur-containing coating in enhancing oxygen redox reversibility.

In this study, we demonstrated a Thiourea-derived surface modification strategy for LRMO cathodes, enabling substantial improvements in the electrochemical performance of SSBs. The thiourea-derived coating delivers a dual functionality: (i) the formation of a stable interphase that suppresses interfacial degradation by preventing direct contact between the LRMO cathode and the solid electrolyte, and (ii) the induction of a surface lattice reconstruction into a spinel-type phase. This reconstructed surface effectively mitigates oxygen loss and transition-metal migration due to its enhanced structural stability, while simultaneously facilitating Li^+ transport through three-dimensional diffusion pathways (as summarized in Figure 5).

4. I think the response to Reviewer #3's Question #5 remains insufficient. The claim that the LRMO composition can be represented as "$0.5Li_2MnO_3 \cdot 0.5LiMnO_2$" lacks supporting experimental evidence, such as quantitative Rietveld refinement of XRD data. Such analysis is essential to reliably determine the phase fraction of the monoclinic Li_2MnO_3 component. Furthermore, while the authors have included HAADF-STEM images showing spinel phases, transmission electron microscopy is inherently a localized technique. The observation of spinel regions in isolated areas does not demonstrate uniform transformation across the entire particle. More statistically representative analysis, such as multi-particle TEM imaging or XRD-based quantification, is needed to substantiate claims regarding the extent of spinel formation.

Respond: We thank the reviewer for this insightful comment. First, we would like to clarify that the expression "$0.5Li_2MnO_3 \cdot 0.5LiMO_2$" (M = Mn, Ni, Co) is widely used in the literature to describe the compositional representation of Li-rich layered oxides rather than implying the coexistence of two physically separated crystalline phases. In most Li-rich layered cathodes, the Li_2MnO_3 component does not exist as a distinct long-range monoclinic phase but instead manifests as short-range Li/Mn ordering domains embedded within the layered *R*-3*m* framework. Consequently, conventional X-ray diffraction typically reveals only weak superlattice reflections associated with Li_2MnO_3-type ordering.

In our synchrotron XRD (SXRD) pattern, weak reflections near ~3.4°, which are characteristic of Li_2MnO_3-type superstructure peaks, can indeed be observed (Fig. 1a). However, due to the limited coherence length of these ordered domains and the presence of stacking faults, which are commonly reported in Li-rich layered oxides, quantitative determination of the Li_2MnO_3 fraction using a two-phase Rietveld refinement model (*R*-3*m* + *C*2/*m*) is generally unreliable.

Therefore, we employed a structural model based on the *R*-3*m* framework with superlattice Li occupation. In this model, the Li_2MnO_3 content can be estimated from the refined Li occupancy on the 3b sites (TM layer) according to:

$$Li_2MnO_3\ (mol\%) = (Li\ occupancy\ on\ 3b\ /\ 0.5) \times 100\%.$$

Based on this refinement model, the TM layer (3b) site hosts 0.194 Li, correspondingly, the Li_2MnO_3 fraction in our LRMO sample is estimated to be approximately 40%.

Regarding the extent of spinel formation, we performed additional TEM characterization on multiple particles. The results consistently show that the LRMO particles are coated and that a spinel phase is present on the particle surface. And we also quantified the spinel phase content with SXRD refinements, and the calculated spinel content on the surface is around 5.2%.

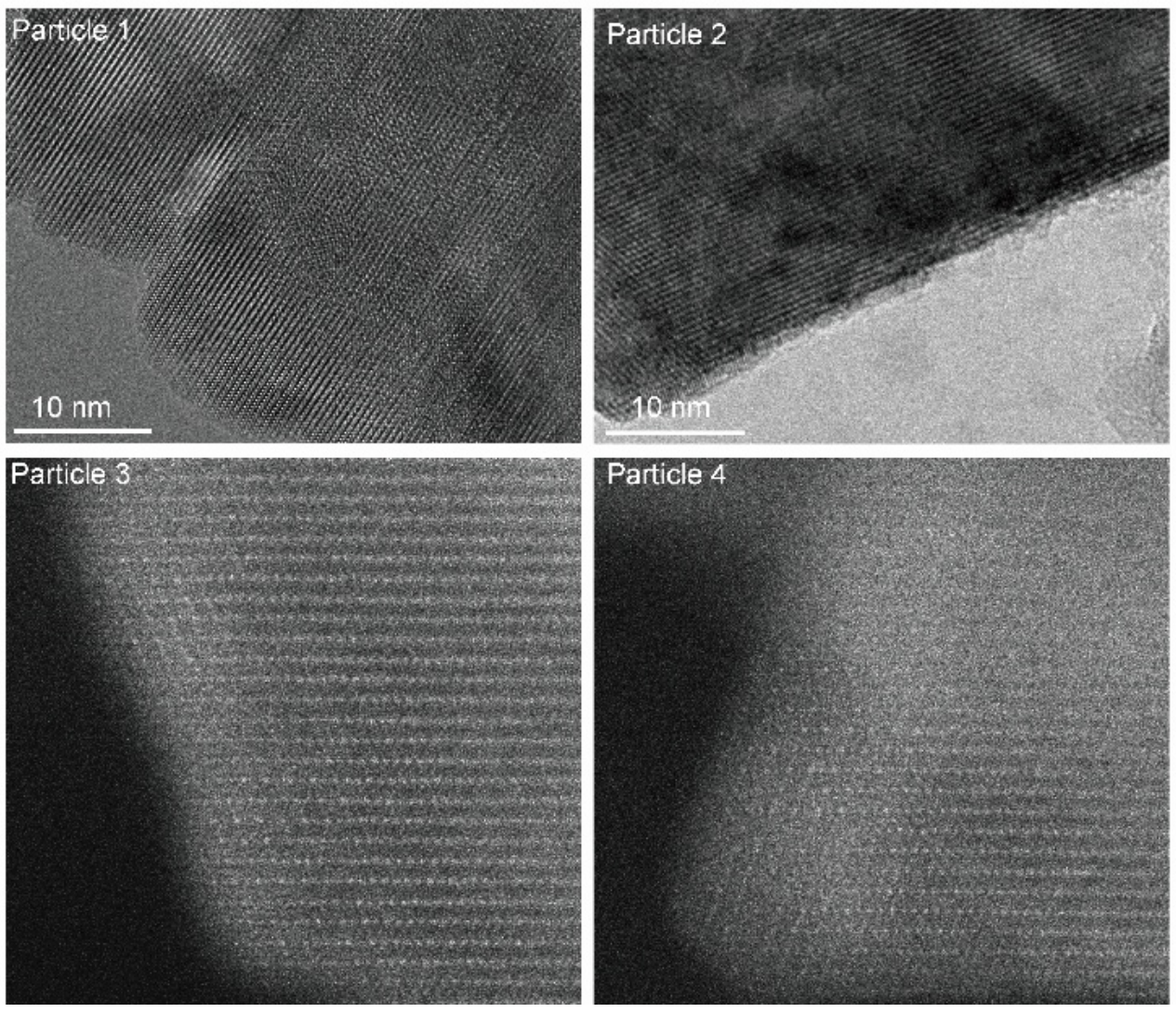

Figure R1. TEM and HR-TEM images of several independent particles.

5. Regarding practical applications, a key concern raised by Reviewer #4, the authors' high-mass-loading (15.28 mg/cm2) results are commendable and improve the work's relevance. However, a small-scale pouch cell demonstration would be a valuable addition. Pouch cells better capture the mechanical constraints and scalability challenges encountered in real-world solid-state battery prototypes. Even limited cycling data from a pouch cell would provide compelling evidence that this coating strategy is translatable to more application-relevant formats.

Respond: We thank the Reviewer for highlighting the importance of evaluating the practical applicability of the material in pouch cell configurations. We fully agree that pouch cells can provide valuable insights into mechanical constraints and scalability challenges encountered in realistic solid-state battery prototypes.

In the present study, we focused on demonstrating electrochemical performance under high-mass-loading conditions (up to 15.28 mg cm^{-2}) using PEEK cells. These experiments already capture key practical challenges, including high areal capacity and interfacial stability under elevated active material loading, and therefore provide strong evidence that the sulfur-derived coating strategy effectively improves interfacial kinetics and

cycling stability under application-relevant conditions.

We believe that the high-mass-loading results presented here already represent a meaningful step toward practical implementation of this material system. Nevertheless, we agree that pouch cell validation would further strengthen the demonstration of scalability. However, the fabrication and optimization of reliable pouch cells for solid-state batteries require extensive additional engineering to obtain meaningful and reproducible results, which is beyond the scope of the current study. We therefore consider pouch cell evaluation as an important next step, and we plan to pursue this direction in future work aimed to integrate the develop cathode materials in prototype solid-state battery formats.

References:

1. Zhou, W. *et al.* 5.1% efficiency of Si photoanodes for photoelectrochemical water splitting catalyzed by porous NiFe (oxy)hydroxide converted from NiFe oxysulfide. *Chem. Commun.* 55, 12627–12630 (2019).
2. Kröcher, O. & Elsener, M. Materials for thermohydrolysis of urea in a fluidized bed. *Chem. Eng. J.* 152, 167–176 (2009).
3. Wang, Y., Liu, Z., Lv, Y., Wang, S. & Du, H. Oxidation of sulfide with the CuO catalyst assisted oxygen microbubbles in alkaline wastewater: Efficiency, sulfur conversion, and mechanisms. *Chem. Eng. J. Adv.* 12, 100408 (2022).
4. Hui, T. *et al.* A dual-coating strategy based on primary and secondary particle structures of Li-rich Mn-based materials. *J. Energy Storage* 127, 117148 (2025).
5. Wei, Y. *et al.* A Structure Self-Healing Li-Rich Cathode Achieved by Lithium Supplement of Li-Rich LLZO Coating. *Adv. Funct. Mater.* 33, 2214775 (2023).
6. Yang, M. *et al.* A surface-to-interface boronation engineering strategy stabilizing the O/Mn redox chemistry of lithium-rich manganese based oxides towards high energy-density cathodes. *Energy Environ. Sci.* 18, 6168–6179 (2025).
7. Yang, P. *et al.* Layered-Spinel Heterogeneous Structure and Oxygen Vacancies Enable Superior Electrochemical Performance for Li-Rich Cathodes. *Angew. Chem. Int. Ed*. 137, e202501539 (2025).
8. Yao, Y. *et al.* Recent advances in coating strategies for lithium-rich manganese-based layered oxide cathodes. *J. Energy Storage* 134, 118132 (2025).
9. Zhao, S. *et al.* Towards high-energy-density lithium-ion batteries: Strategies for developing high-capacity lithium-rich cathode materials. *Energy Storage Mater.* 34, 716–734 (2021).
10. Zhao, T. *et al.* Exploring the Mechanism of Surface Cationic Vacancy Induces High Activity of Metastable Lattice Oxygen in Li- and Mn-Rich Cathode Materials. *Angew. Chem. Int. Ed.* 64, e202419664 (2025)
11. Xu, B. *et al.* Factors affecting Li mobility in spinel LiMn2O4—A first-principles study by GGA and GGA+U methods. *J. Power Sources* 195, 4971–4976 (2010).
12. Xiao, R. *et al.* Density Functional Investigation on Li_2 MnO_3. *Chem. Mater.* 24, 4242–4251 (2012).
13. Xu, J. *et al.* The basic physical properties of Li2MnO3 and LiMn2O4 cathode materials. *Comp. Mater. Sci.* 229, 112426 (2023).
